# A modular high-throughput approach for advancing synthetic biology in the chloroplast of *Chlamydomonas*

**DOI:** 10.1038/s41477-025-02126-2

**Published:** 2025-11-03

**Authors:** René M. Inckemann, Tanguy Chotel, Michael Burgis, Cedric K. Brinkmann, Laura Andreas, Jessica Baumann, Priyati Sharma, Melanie Klose, James Barrett, Fabian Ries, Nicole Paczia, Timo Glatter, Luke C. M. Mackinder, Felix Willmund, Tobias J. Erb

**Affiliations:** 1 Department of Biochemistry & Synthetic Metabolism, Max Planck Institute for Terrestrial Microbiology, Marburg, Germany; 2 SYNMIKRO Center of Synthetic Microbiology, Marburg, Germany; 3 Department of Biology, University of Marburg, Marburg, Germany; 4 Centre for Novel Agricultural Products, Department of Biology, University of York, York, UK; 5 Group Genetics of Eukaryotes, TU Kaiserslautern, Kaiserslautern, Germany

**Keywords:** Molecular engineering in plants, High-throughput screening

## Abstract

Chloroplast synthetic biology holds promise for advancing photosynthetic organisms through improving the function of plastids. However, chloroplast engineering efforts face limitations due to the scarcity of genetic tools and the low throughput of plant-based systems. To address these challenges, we here established *Chlamydomonas reinhardtii* as a prototyping chassis for chloroplast synthetic biology. To that end, we developed an automation workflow that enables the generation, handling and analysis of thousands of transplastomic strains in parallel. Furthermore, we expanded the repertoire of effective selection markers and reporter genes, and we characterized over 140 regulatory parts, including native and synthetic promoters, 5′ and 3′ untranslated regions, and intercistronic expression elements. We integrated the system with existing molecular cloning standards and demonstrated several applications, including a library-based approach to develop synthetic promoter designs in plastids. Finally, we provide a proof of concept for prototyping metabolic pathways in plastids by introducing a chloroplast-based synthetic photorespiration pathway, resulting in a threefold increase in biomass production. Overall, our study advances current chloroplast engineering efforts by providing a high-throughput platform and standardized genetic parts for the rapid prototyping and characterization of plastid manipulations with the prospect of high transferability between different chloroplasts, including those of higher plants and crops.

## Abstract (web summary)

This study reports a modular-cloning-based platform for high-throughput chloroplast engineering in *Chlamydomonas reinhardtii* that allows large-scale characterization of genetic parts and prototyping of new traits, as demonstrated for the photorespiration pathway.

## Main

Synthetic biology offers promising prospects for developing photosynthetic organisms and crop varieties with improved traits^1^. These include enhanced resilience to environmental challenges, such as heat and drought^2^; superior nutrient content, such as omega fatty acids^3^ and vitamins^4^; and improved yield, for instance through more efficient photorespiratory bypasses^5–10^ or entirely new-to-nature carbon fixation cycles^11–15^. However, realizing the full potential of synthetic biology in photosynthetic eukaryotes will require substantial advancements in genetic engineering capabilities that go beyond traditional breeding and gene-editing techniques^16^.

In particular, chloroplasts have become an increasing target for advanced engineering efforts^7,17–19^. These organelles harbour the photosynthetic apparatus and house several metabolic processes of high interest for the engineering of metabolic traits. For example, chloroplast genome (plastome)-based engineering of plastid isoprenoid metabolism has recently proved a highly effective strategy for the production of high-value compounds, such as astaxanthin and artemisinin^20,21^. Moreover, several subunits of the light-harvesting and carbon fixation machineries are encoded by the plastome, which makes the direct manipulation of the chloroplast genome central for any efforts that aim at improving photosynthetic yield. Current examples are efforts to integrate carboxysome- and pyrenoid-based CO_2_-concentrating mechanisms into chloroplasts to boost photosynthetic efficiency^22–30^. These examples emphasize the growing need to develop advanced genetic tools for the direct manipulation of the plastome. Chloroplast genome engineering offers additional advantages over nuclear-genome-based engineering strategies of chloroplast functions. The expression of transgenes from the chloroplast genome benefits from precise genomic integration, the absence of (nuclear) silencing mechanisms, the potential for significantly higher protein levels and improved genetic containment, as transgenes are not transmitted by pollen due to the strict maternal inheritance of chloroplasts^31^.

Despite all these advantages, genetic engineering of the plastid is still constrained to only a handful of available genetic elements and tools^32,33^. For example, only a small number of natural gene expression elements (such as 5′ and 3′ untranslated regions (UTRs) and promoters) are available, which limits the number of genes that can be stacked and provides little control over their expression strength^32^. This limited tool set is even more constrained, as gene expression elements cannot be simply reused because of the high homologous recombination frequency of the plastome, which is observed for sequences as short as 50 base pairs^34^. There is thus a pressing need to bring the applications of synthetic biology in plastids to the level of versatility and complexity that is available for other organisms. Moreover, methods are required for the systematic assembly and large-scale characterization of gene expression elements in chloroplasts^19,35,36^. However, the long generation times of photosynthetic eukaryotes and the absence of suitable model systems that are compatible with high-throughput handling routines have impeded the development of genetic tools for the chloroplast thus far^37^. Consequently, the systematic development of plastid transgene expression strategies and the prototyping of complex genetic designs in chloroplasts have remained beyond reach.

Over many decades, *Chlamydomonas reinhardtii* has proved to be a valuable model system to study basic physiological processes in photosynthetic organisms, including plant cells. For example, an impressive share of our current knowledge about the function of the conserved photosynthesis processes was gained by work with *C. reinhardtii*^38^. The alga was also one of the first photoautotrophic organisms that allowed the transformation and engineering of plastidic genes. While individual players of plastid gene expression differ between *C. reinhardtii* and land plants, several plastomic elements of *C. reinhardtii* have already been successfully implemented in transplastomic studies with model plants such as tobacco^18^, demonstrating the potential to transfer knowledge between the organisms.

Even though *C. reinhardtii* offers several advantages that make it an interesting chassis to advance synthetic biology efforts in plastids, such as a single chloroplast per cell, fast growth and the possibility to cultivate strains in high-throughput formats^39–42^, plastomic transgene expression in *C. reinhardtii* still suffers from limitations. These include low expression rates of transgenes and only a handful of genetic elements that are available for basic genetic engineering^43^, including regulatory parts, selection markers and reporters. Moreover, methods for time- and cost-efficient cloning and high-throughput characterization of genetic parts currently limit the capacity to realize advanced genetic designs in plastids.

In this study, we aimed to overcome the above shortcomings by developing technological and genetic resources to establish *C. reinhardtii* as a chassis for chloroplast synthetic biology. To generate, handle and analyse thousands of transplastomic *C. reinhardtii* strains in parallel, we designed and implemented an automation workflow, expanded the number of selection markers for chloroplast transformation, and established several new reporter genes for fluorescence and luminescence-based read-outs and cell sorting.

Building on these resources, we systematically characterized a collection of more than 140 regulatory parts, including 35 different 5′UTRs, 36 3′UTRs, 59 promoters and 16 intercistronic expression elements (IEEs) for advanced gene stacking. We embedded these genetic parts within a Phytobrick/modular cloning (MoClo) framework^19^ for the standardized and automated assembly of genetic constructs that is compatible with existing *C. reinhardtii* and plant MoClo resources. Using this MoClo routine allowed us to rapidly assemble and characterize gene element combinations at multiple insertion loci to establish multi-transgene constructs that range across more than three orders of magnitude in expression strength. Finally, we showcase the utility of our tools by developing more than 30 synthetic promoters through a pooled library-based approach in chloroplasts and providing a real-world application case by introducing a recently reported synthetic photorespiration pathway into the chloroplast^7^.

Overall, our work provides a high-throughput workflow for chloroplast synthetic biology in *C. reinhardtii* and a foundational set of genetic elements for the convenient engineering of the chloroplast that is freely available to the community. Because of the compatibility and transferability of results between the chloroplast of *C. reinhardtii* and those of other organisms^44–46^, we note that our platform might offer a promising test bed beyond *C. reinhardtii* for prototyping advanced genetic designs prior to their implementation in plant and crop plastids.

## Results

### Automation workflow for high-throughput characterization of transplastomic *C. reinhardtii* strains

**Fig. 1:**
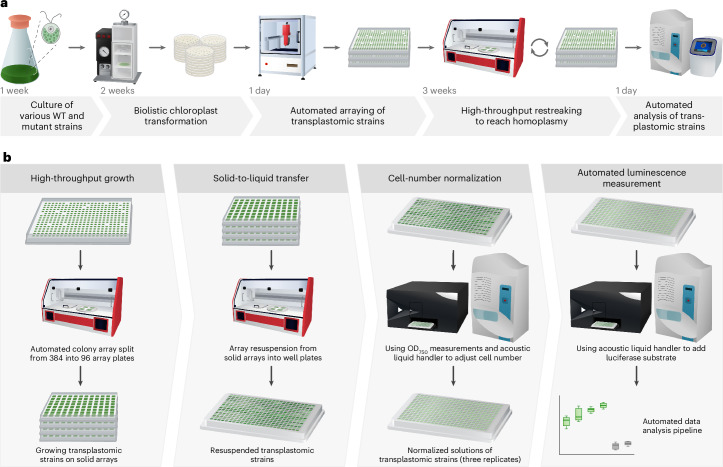
Workflow for generation and high-throughput characterization of transplastomic *C. reinhardtii* strains. **a**, Automated transplastomic strain generation workflow. Chloroplast transformation is performed via biolistic transformation using a particle gun, and subsequent automated arraying of transformants is done via a PIXL picking robot. Transplastomic strains are restreaked three times to reach homoplasmy using a Rotor screening robot. Automated genotyping of transplastomic strains is performed via an acoustic liquid handler and cPCR analysis to confirm integration and homoplasmy. **b**, High-throughput characterization of transplastomic strains. Colonies are transferred to 96-well formats for biomass cultivation, preparing for reporter gene measurement. The Rotor robot is then used to transfer biomass from agar (96 strains per plate) to multi-well plates for resuspension. OD_750_ is measured, and the Echo liquid handler is used for cell normalization and NanoLuc luciferase substrate addition.

To facilitate the systematic characterization of genetic parts across thousands of transplastomic *C. reinhardtii* strains, we developed an automated workflow dedicated to modular and synthetic engineering of the chloroplast genome. This workflow is inspired by recent efforts of handling large collections of nuclear transformants in *C. reinhardtii*^39^. However, our workflow shows several key differences and advances the level of automation. Most notably, our platform is based on solid-medium cultivation, which is more reproducible than liquid-medium cultivation, and leverages a contactless liquid-handling robot to enhance the capacity for managing an increased number of strains (Fig. 1). In brief, our workflow relies on the automated picking of transformants into a standardized 384 format and subsequent restreaking to achieve homoplasy, using a Rotor screening robot. These colonies are then organized into a 96-array format for high-throughput biomass growth, liquid-medium transfer and/or additional screens such as reporter gene analysis. The latter involves using the Rotor screening robot for the transfer of biomass from 96-array agar plates into multi-well plates filled with water. Following resuspension, the optical density is measured at 750 nm (OD_750_), and the contact-free liquid handler is used for cell number normalization, medium transfer and the supplementation of additional compounds (for example, the substrate for luciferase assays; see below).

Switching to a (partially) solid-medium-based workflow proved to be more time- and cost-efficient than individual screening of transplastomic lines in liquid medium, which inherently limited the number of strains that could be handled in parallel. For example, it was possible to easily drive 80% of the transformants into homoplasmy by simultaneously screening 16 replicate colonies per construct on plates over the course of three weeks, with only minor losses observed (~2% total losses—that is, less than one replicate out of 3 × 16 replicates). Using this workflow reduced the time needed for picking and restreaking by about eightfold (from 16 h weekly for 384 strains to 2 h weekly when using the pipeline), while yearly maintenance spending was reduced by twofold (comparing running costs prior to the establishment of the platform). Overall, this automation workflow was capable of generating and managing the 3,156 individual transplastomic strains that were used in this study.

### Establishing a foundational set of genetic parts for chloroplast engineering

**Fig. 2:**
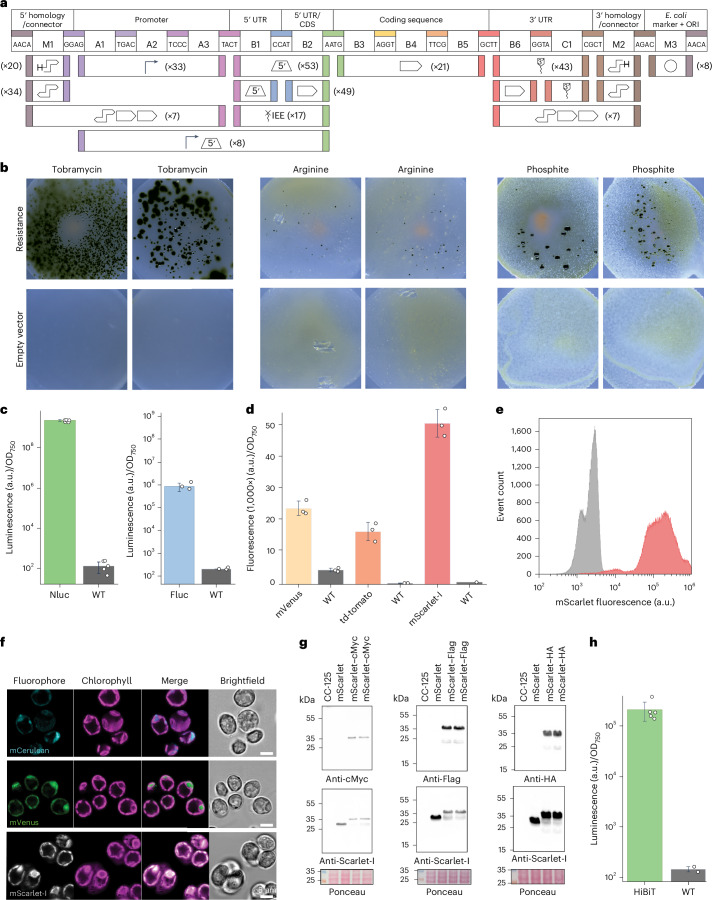
Establishing basic tools for chloroplast engineering. **a**, Architecture of a chloroplast MoClo system and overview of the created genetic parts. The architecture follows the Phytobrick standard^47^ and conserves the cloning overhangs of this system, as well as the nomenclature for the part types (A1–C1). The positions M1–M3 allow for higher modularity as reported by Stukenberg et al.^83^. The number of parts is indicated for each position. **b**, Selection markers for chloroplast transformation. Three different selection marker systems are shown, with two replicates and two negative controls for each one. Successful selection after chloroplast transformation can be observed for all three of them, notably with a high efficiency for tobramycin-based selection (100 µg ml^−1^). **c**, NanoLuc (Nluc) and firefly luciferase (Fluc) as luminescence reporters for the chloroplast. The means and standard deviations of luminescence signal of transplastomic strains containing a NanoLuc or Fluc expression cassette (green and blue, respectively) are plotted as arbitrary units (a.u.) normalized to OD and compared with the WT strain (grey). *n*_biological_ = 5, *n*_technical_ = 3 for NanoLuc, and *n*_biological_ = 3, *n*_technical_ = 3 for Fluc. **d**, Means and standard deviations of adapted fluorescent reporters for the chloroplast. Fluorescent signals of transplastomic strains containing different fluorescent reporter expression cassettes, measured via a plate reader, are plotted as arbitrary units normalized to OD_750_ and compared with the WT strain (grey). *n*_biological_ = 3, *n*_technical_ = 3. **e**, Flow cytometry data of the transplastomic strain containing an mScarlet-I expression cassette (red) compared with the WT strain (grey). **f**, Fluorescence microscopy images of the transplastomic strains containing different fluorophores (mCerulean, mVenus and mScarlet-I). **g**, Western blot analysis of transplastomic strains producing mScarlet FLAG-tagged, HA-tagged and cMyc-tagged in the carboxy-terminal position. Each strain is also tested for the presence of mScarlet using mScarlet-I-specific probes. Ponceau gels are shown for each gel, and each experiment was performed twice to confirm reproducibility. **h**, NanoLuc assay for the HiBiT tag in the C-terminal position. Means and standard deviations are plotted as arbitrary units normalized to OD_750_ and compared with the WT strain (grey). *n*_biological_ = 5, *n*_technical_ = 3. Source Data

**Extended Data Fig. 1:**
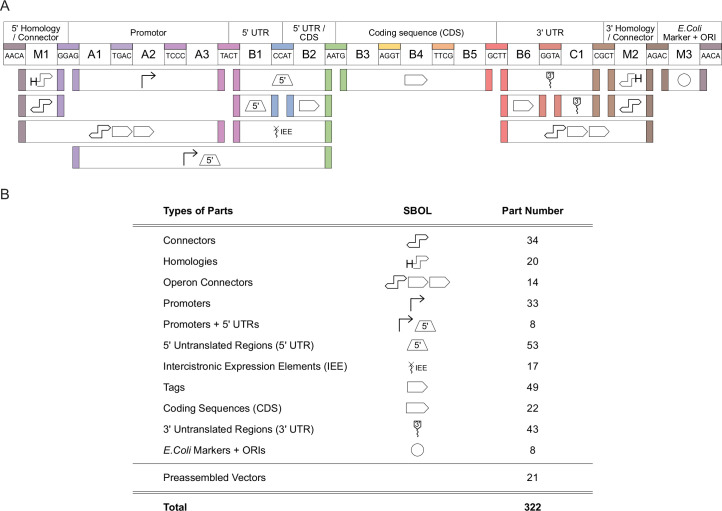
Architecture of the chloroplast MoClo system. Architecture of the chloroplast modular cloning (MoClo) system and overview of the created genetic parts. **a**, The architecture follows the Phytobrick standard^47^ and conserves the cloning overhangs of this system, as well as the nomenclature for the part types (A1-C1). The position M1-3 allow for higher modularity as reported by Stukenberg et al. Number of parts are indicated for each position. **b**, List of parts. Each type of parts is listed, their SBOL depicted and the number of parts associated indicated.

We next established a foundational set of >300 genetic parts for plastome manipulation of *C. reinhardtii* (wild-type (WT) strain CC-125), which we embedded in a standardized MoClo framework^47,48^ (Fig. 2a and Extended Data Fig. 1). MoClo uses a standardized syntax for the modular assembly of transcription units and multiple gene constructs by combining defined genetic elements, including selection markers, promoters, UTRs, terminators, affinity tags, reporter genes and intergenic regions, through Golden Gate cloning. Golden Gate cloning is based on Type IIS restriction enzymes that cut DNA sequences outside their recognition sequence and generate defined four-nucleotide overhangs, allowing the assembly of genetic elements according to a predefined standard, which enables quick combinatorial assembly and exchange of individual genetic elements.

**Extended Data Fig. 2:**
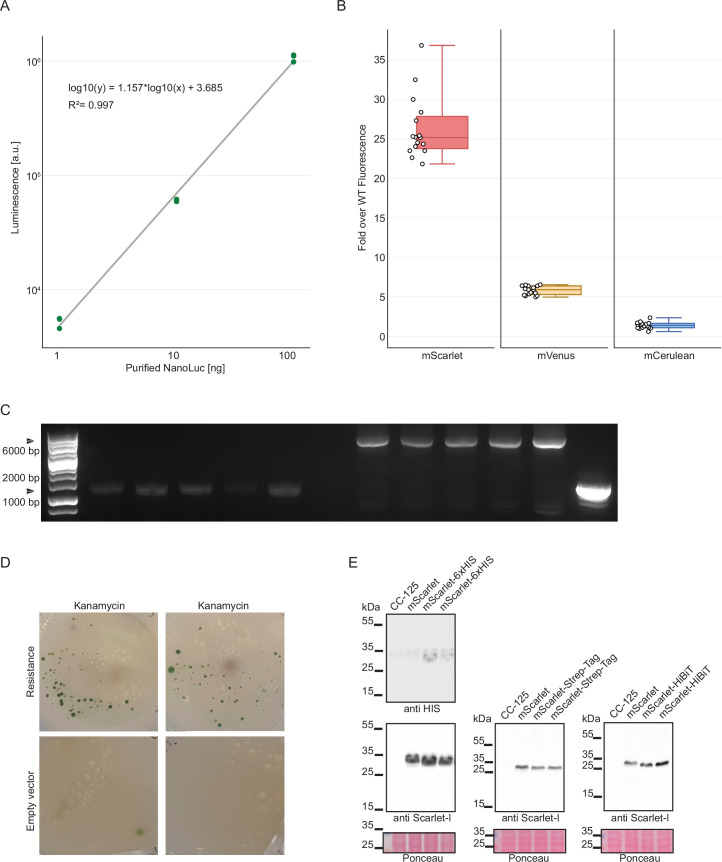
Additional Reporters and selection markers. **a**, Calibration curve for purified NanoLuc concentration (ng) versus luminescence intensity (a.u.). The relationship between nanoluciferase abundance and luminescence is linear. **b**, Fluorescent reporter characterization. Fold over WT fluorescence is displayed for each strain, normalized by chlorophyll content, each expressing a different fluorescent reporter. n_biological_ = 16. **c**, PCR analysis of tobramycin-resistant strains. The first section of the gel illustrates PCR products of the tobramycin resistance gene, exclusive to the five genetically modified strains, with ~1500 bp bands, absent in WT. The latter section shows PCR products from the insertion site, with ~8000 bp bands in engineered strains and a 2000 bp band in WT, indicating homoplasmy in the modified strains. **d**, Selection marker for chloroplast transformation, with two replicates and two negative controls for each one. Successful selection after chloroplast transformation can be observed. **e**, Western blot analysis of transplastomic strains producing mScarlet HIS-tagged, Strep-tagged, Hibit-tagged in the Cterm position. Each strain is also tested for the presence of mScarlet using mScarlet specific probes. Ponceau gels are shown for each gel, each experiment was performed twice to confirm reproducibility. All box plots show median as solid line, the box with lower and upper quartile, and the whiskers representing the data points that fall within 1.5 times the interquartile range (IQR) from the lower and upper quartiles. Any data point outside this range is considered as outlier.ss. Source Data

Our library of >300 parts encompasses native regulatory elements, such as 5′UTRs, 3′UTRs and IEEs, that we derived from the chloroplast genome of *C. reinhardtii* and tobacco, as well as some synthetic designs. Our library also contains parts for integration into various loci in the chloroplast genome (Extended Data Fig. 1). In addition to spectinomycin (the *aadA* gene), which is the predominantly used selection marker for *C. reinhardtii* chloroplast transformations, our repository includes a comprehensive set of codon- and sequence-optimized selection makers that had been reported in different setups in the past. For example, we adopted tobramycin, which was previously demonstrated only for *Nicotiana tabacum* chloroplast transformation, as a marker for *C. reinhardtii*^49^ (Fig. 2b and Extended Data Fig. 2c). When using tobramycin, we observed 1,200 to 2,500 colonies per plate (~10 µg DNA biolistic transformation, *n* = 1), which was tenfold higher than spectinomycin-based selections (Supplementary Fig. 4), with no cross-resistance between the two antibiotics (Supplementary Fig 5). We also codon-optimized, validated and integrated kanamycin^50^ and phosphite^51,52^ selection markers in our library, as well as an arginine-based selection system^53^ that complements arginine auxotrophy in a *C. reinhardtii Arg9* deletion background (Fig. 2b and Extended Data Fig. 2d).

To characterize and quantify regulatory elements, we codon-optimized and validated different luciferase-based and fluorescent reporters for their use in the chloroplast of *C. reinhardtii*, which had proved difficult in the past^43^. As the primary reporter, we established NanoLuc luciferase (Extended Data Fig. 2a), which did not interfere with chloroplast autofluorescence and showed a high signal-to-noise ratio that was seven orders of magnitude higher than the WT background and superior to other fluorescent reporters we tested (Fig. 2c,d). As an alternative to NanoLuc, we integrated the previously reported firefly luciferase^54^ into our library. Moreover, we tested four different fluorescent reporters (mVenus, mScarlet-I, td tomato and mCerulean), of which mScarlet-I performed best. This reporter showed little interference with chloroplast autofluorescence (Fig. 2d) and could be successfully applied in flow cytometry and microscopy (Fig. 2e). While the fluorescence of mCerulean measured in the plate reader was only 1.5 times higher (Extended Data Fig. 2b) than the average WT signal, it was still sufficient for microscopic analysis (Fig. 2f).

Finally, we encompassed additional parts into our library, which included modular tags (FLAG, HA and cMyc tags) for immunoblots and a HiBiT–luciferase complementation assay to tag and detect proteins of interest (Fig. 2g,h and Extended Data Fig. 2e). This set of selection markers, reporter genes and regulatory parts provided the basis for all high-throughput characterization efforts and engineering applications of this study.

### High-throughput characterization of regulatory elements

Having established the initial library of parts, we proceeded to systematically quantify the different regulatory parts using the NanoLuc-based read-out. To that end, we tested various 5′UTRs, as well as 3′UTRs, both of which are known to strongly influence and regulate gene expression in the chloroplast^55–58^. Despite their cyanobacterial origins, chloroplasts show distinct differences in the regulation of gene expression compared with their prokaryotic counterparts^59^. While prokaryotic gene expression is predominantly controlled at the transcriptional level, chloroplast gene expression is mainly regulated post-transcriptionally via RNA elements in the 5′ and 3′UTRs^60^. In particular, 5′UTRs are known to interact with chloroplast mRNA binding factors that regulate transcript maturation, stability and translation efficiency, thus determining final gene expression levels^61–65^.

**Extended Data Fig. 3:**
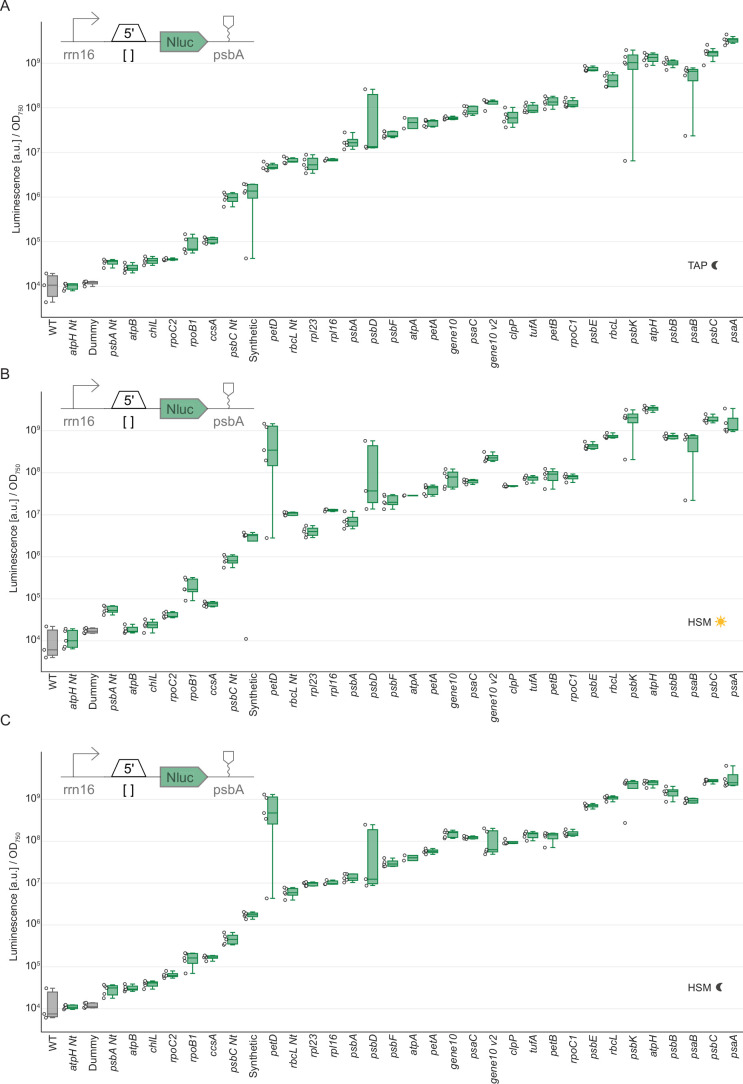
Characterization of 5′ UTR under different light and medium conditions. 5’UTRs were characterized by measuring NanoLuc activity of transplastomic strains containing different constructs, which differ in their 5’UTR. Nanoluc signal of a transplastomic strain containing a NanoLuc expression cassette (green) is plotted as arbitrary units [a.u.] normalized to OD and compared to WT strain (gray). For all measurements, n_biological_ = 5, n_technical_ = 3. **a**, NanoLuc assay under TAP dark conditions, **b**, under HSM light conditions and **c**, under HSM dark conditions. PetD stands out under HSM conditions. All box plots show median as solid line, the box with lower and upper quartile, and the whiskers representing the data points that fall within 1.5 times the interquartile range (IQR) from the lower and upper quartiles. Any data point outside this range is considered as outlier. Source Data

To test the influence of 5′UTRs on gene expression, we constructed 35 different genetic constructs, each composed of the *rrn16* promoter, followed by a variable 5′UTR, the NanoLuc luciferase coding sequence, the *psbA* 3′UTR and homology sequences for integration at the *psbH* locus^66^, which is the most widely used integration site in *C. reinhardtii*. As the 5′UTR, we used natural sequences sourced from *C. reinhardtii* and tobacco, as well as synthetic sequences. For each of the 35 designs, we picked 16 biological replicates, which we tested under mixotrophic and photoautotrophic growth conditions (Tris–acetate–phosphate (TAP) medium and high-salt medium (HSM), respectively) and dark versus light conditions (Extended Data Fig. 3). In total, this experiment involved the handling and analysis of 2,240 transplastomic strains.

**Fig. 3:**
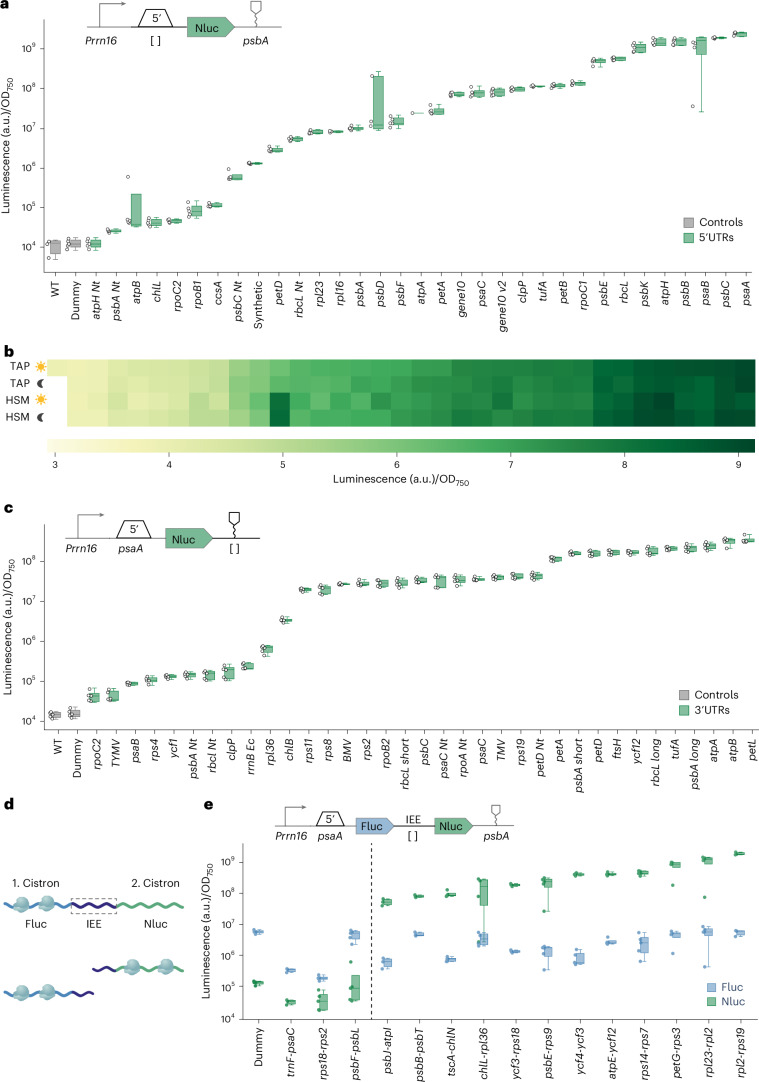
Characterization of regulatory parts for chloroplast gene expression. **a**, 5′UTR characterization. 5′UTRs were characterized by measuring NanoLuc activity of transplastomic strains containing different constructs, which differ in their 5′UTR. The NanoLuc signals of transplastomic strains containing a NanoLuc expression cassette (green) are plotted as arbitrary units normalized to OD_750_ and compared with the WT strains (grey). For all measurements, *n*_biological_ = 5, *n*_technical_ = 3. **b**, 5′ characterization under four different conditions. The heat map shows the NanoLuc signal of transplastomic strains under light, dark, TAP and HSM conditions. Similar expression patterns can be observed under all four conditions. **c**, 3′UTR characterization. 3′UTRs were characterized by measuring NanoLuc activity of transplastomic strains containing different constructs, which differ in their 3′UTR. The NanoLuc signals of transplastomic strains containing a NanoLuc expression cassette (green) are plotted as arbitrary units normalized to OD_750_ and compared with the WT strains (grey). For all measurements, *n*_biological_ = 5, *n*_technical_ = 3. **d**, Function of IEEs. Two cistrons are connected via an IEE. After processing via imported *cis*-factors, both cistrons are translated separately. **e**, IEE characterization. IEEs were characterized by measuring NanoLuc and Fluc activity of transplastomic strains containing different constructs, which differ in their IEE. The luciferase signals of transplastomic strains containing a NanoLuc expression cassette (green) are plotted as arbitrary units normalized to OD_750_ and compared with Fluc luminescence (blue). The dashed line differentiates IEEs with insufficient support for robust expression (left) from those facilitating strong expression (right). For all measurements, *n*_biological_ = 5, *n*_technical_ = 3. For each measurement, we included a dummy part that lacks any secondary structure, promoter elements, ribosome-binding motifs or RNA-binding protein sites, serving as a negative control. In each box plot, the central line indicates the median, the box indicates the lower and upper quartiles, and the whiskers represent the data points that fall within 1.5 times the interquartile range from the lower and upper quartiles. Any data point outside this range is considered an outlier. Source Data

Overall, the different transplastomic strains showed a broad luminescence, which spanned more than three orders of magnitude (Fig. 3a). Strains carrying 5′UTRs of RNA polymerase and ribosomal proteins (*rpoC2*, *rlp16* and *rpl23*) showed lower expression levels on average, while strains carrying 5′UTRs of highly abundant proteins in photosynthesis (*rbcL*, *psaA* and *psbC*) showed high expression levels, aligning well with recently reported Ribo-seq data for *Chlamydomonas*^67^. These expression patterns proved to be consistent across the four different growth conditions, except for the *petD* 5′UTR, which somehow displayed divergent expression patterns across the two different media (Fig. 3b and Extended Data Fig. 3a–c).

We next focused on 3′UTRs. We constructed 36 different genetic constructs targeting the *psbH* locus, each carrying the *rrn16* promoter, the *psaA* 5′UTR, the NanoLuc luciferase coding sequence and a variable 3′UTR, which we derived from *C. reinhardtii*, tobacco or viral origin. In these experiments, the luminescence of the different transplastomic strains showed three levels of variability spanning three orders of magnitude (Fig. 3c), low, medium and high, illustrating the relevance of the 3′UTR for gene expression activity in chloroplasts, although to a much lesser extent than the 5′UTR.

Finally, we investigated IEEs, which facilitate polycistronic gene expression in the chloroplast. IEEs promote the processing of polycistronic mRNAs into stable monocistronic transcripts and protect these mRNAs against degradation^68,69^ (Fig. 3d). Using small RNA profiling data from the chloroplast of *Chlamydomonas*^70^, we identified 15 potential IEE candidates, which we tested between firefly luciferase and NanoLuc luciferase as reporter genes (Fig. 3e). All sequences tested showed successful expression of the downstream gene, except for three sequences (*trnF-psaC*, *rps18-rps2* and *psbF-psbL*). Since polycistronic chloroplast transcripts are thought to be trimmed into monocistronic units prior to translation^70^, we speculate that these three IEEs might be less efficiently processed in the tested transgenes. Interestingly, the functional IEEs showed up to tenfold variation in luciferase activity levels, highlighting the potential of these elements in modulating the expression of stacked transgenes within the chloroplast (Fig. 3e). In summary, these experiments established and quantified the behaviour of more than 80 genetic elements in the chloroplast.

### Developing synthetic chloroplast expression elements through rational and library-based approaches

Most of the genetic elements tested thus far were of natural origin. We therefore sought to extend our design efforts to fully synthetic (that is, non-native) genetic parts, particularly promoters. Inspired by the architecture of the *C. reinhardtii rrn16* promoter, we developed a minimal scaffold, consisting of only 46 base pairs carrying the −35 and −10 motifs of *rrnS*. We preserved these −35 and −10 motifs across the majority of these promoters and modified the rest of the sequence to modulate expression strength, while reducing the risk of unintended homologous recombination for multi-gene constructs.

**Fig. 4:**
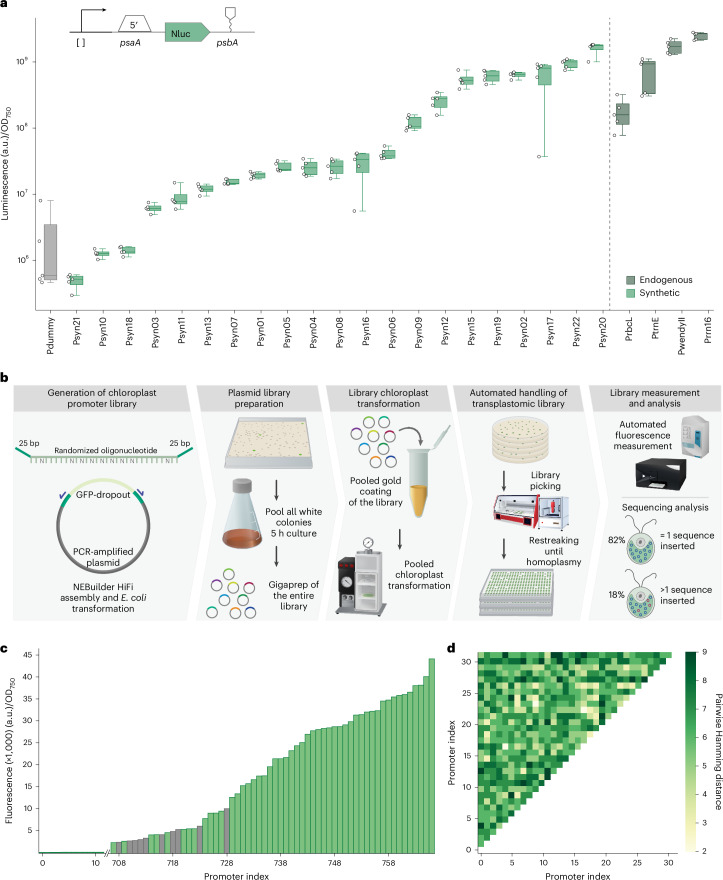
Designing and testing synthetic chloroplast promoters and library chloroplast transformation. **a**, Synthetic promoter characterization. Promoters were characterized by measuring the NanoLuc activity of transplastomic strains containing different constructs, which differ in their promoters. The NanoLuc signals of transplastomic strains containing a NanoLuc expression cassette (green) are plotted as arbitrary units normalized to OD_750_ and compared with a dummy sequence (grey). The dashed line demarcates synthetic promoters (left, light green) from endogenous ones (right, dark green). For all measurements, *n*_biological_ = 5, *n*_technical_ = 3. In each box plot, the central line indicates the median, the box indicates the lower and upper quartiles, and the whiskers represent the data points that fall within 1.5 times the interquartile range from the lower and upper quartiles. Any data point outside this range is considered an outlier. **b**, Conceptual workflow for transforming construct libraries into the chloroplast. Sequencing analysis showed that 82% of transformants contained a single promoter variant, and for 18% multiple sequence variants could be detected, **c**, Fluorescence measurement of the transplastomic promoter library. mScarlet-I fluorescence of the transplastomic strains containing different promoter variants was measured (excitation, 569 nm; emission, 605 nm). Fluorescence is plotted as arbitrary units normalized to OD _750_. A total of 768 transplastomic strains were measured, and a range of different mScarlet-I signals were detected. The grey bars represent examples that were selected in **d** for sequencing. **d**, Sequence analysis of sequenced transplastomic strains. Pairwise Hamming distance is plotted for the promoter sequence variant, which was found in a set of 32 sequenced transplastomic strains. Source Data

In total, we designed 21 different promoters (for the specific design rules, see Supplementary Text 5) and tested them in a standardized construct that was integrated at the *psbH* locus and included the different promoter sequences upstream of the *psaA* 5′UTR, the coding sequence of NanoLuc luciferase and the *psbA* 3′UTR. Indeed, the luminescence signals of the different transplastomic strains varied across three orders of magnitude, suggesting that our synthetic promoters covered a wide range of expression strengths (Fig. 4a).

Even though the above experiments successfully identified synthetic promoters, we note that the biolistic transformation remained a bottleneck in our automated workflow. This is because each of the 21 promoters that we tested required an individual transformation, limiting the total number of promoter designs that could be assessed. To address this constraint, we explored the feasibility of a pooled library approach, as opposed to transforming individual constructs. As a proof of concept, we sought to transform and screen a library of degenerated promoter sequences. This library was created by cloning a degenerated single-strand oligonucleotide into a pre-assembled vector containing a spectinomycin selection marker and an mScarlet expression cassette (Fig. 4b). After the cloning step, *Escherichia coli* colonies were pooled, and the DNA of the entire plasmid library was prepped and used in 20 parallel biolistic transformations, resulting in approximately 10,000 colonies. Of these, a subset of 1,536 random spectinomycin-resistant colonies were screened in a 1,536 format on a single agar plate. A total of 768 transformants were subsequently assayed via a fluorescent read-out, in three technical replicates per colony (without confirming homoplasmy for all 768 transformants). While 90% of the strains showed only little or no fluorescence, 10% showed a broad spectrum of mScarlet signal. We continued to characterize selected transformant strains in more depth (Fig. 4c).

From the 10% of strains showing an mScarlet signal, we randomly selected 32 individual strains and subjected them to sequencing. Using the individual promoter sequences of the library as barcodes enabled us to analyse whether individual sequence variants had been inserted or whether a mixture of sequences persisted across the genome copies. Of these 32 strains, 26 (82%) contained one unique sequence, while the remaining 6 (18%) exhibited a mixture of sequences (Fig. 4d). Overall, these findings not only provide more than 20 additional synthetic promoter sequences for the chloroplast but also demonstrate the power of pooled library transformation in chloroplasts, opening new possibilities for plastid synthetic biology and engineering.

### Prototyping the effects of different genetic contexts on transgene expression

In all prior experiments, the different genetic elements were always assessed in the same genetic context—that is, within the same transcription unit (and at one specific integration site), varying only one parameter (for example, promoter, 5′UTR or 3′UTR). While this setup was helpful for studying the individual behaviour of the respective elements that were tested, for more advanced engineering approaches a collection of orthogonal part combinations are required that are unique in their respective sequences and can be used together in multi-transgene expression without the risk of unintended homologous recombination.

**Fig. 5:**
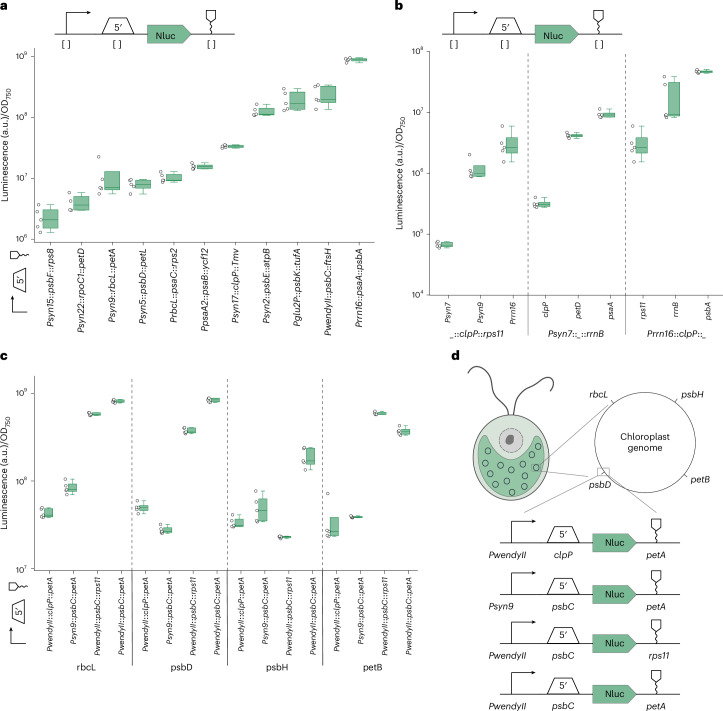
Characterizing the effects of different genetic contexts. **a**, Characterizing various combinations of parts. Combinations were characterized by measuring the NanoLuc activity of transplastomic strains containing different constructs, which differ in their promoters, 5′UTRs and 3′UTRs. The NanoLuc signals of transplastomic strains containing a NanoLuc expression cassette (green) are plotted as arbitrary units normalized to OD_750_. For all measurements, *n*_biological_ = 5, *n*_technical_ = 3. A range of three orders of magnitude of NanoLuc signal can be observed. **b**, Characterizing parts in weak expression backgrounds. Combinations were characterized by measuring the NanoLuc activity of transplastomic strains containing different constructs, which differ in their promoters, 5′UTRs and 3′UTRs. The NanoLuc signals of transplastomic strains containing a NanoLuc expression cassette (green) are plotted as arbitrary units normalized to OD_750_. For all measurements, *n*_biological_ = 5, *n*_technical_ = 3. A range of two orders of magnitude of NanoLuc signal can be observed. **c**, The effects of different sites of integration across the chloroplast genome were characterized by measuring the NanoLuc activity of transplastomic strains containing different constructs, with four different combinations of parts integrated in four different locations in the chloroplast genome. **d**, Conceptual design of the characterization of different integration sites. Four different constructs, which vary in the promoter, 5′UTR and 3′UTR, are integrated in four different locations of the chloroplast genome. In each box plot, the central line indicates the median, the box indicates the lower and upper quartiles, and the whiskers represent the data points that fall within 1.5 times the interquartile range from the lower and upper quartiles. Any data point outside this range is considered an outlier. Source Data

To prototype such orthogonal part combinations, we devised 11 unique combinations, all expressing the NanoLuc reporter, but each varying in the promoter, 5′UTR and 3′UTR (Fig. 5a). In these experiments, the luminescence of the different combinations spanned three orders of magnitude, indicating a broad range of expression strength. In almost all cases, expression strength showed a similar trend that followed the expected 5′UTR activity (and promoter activity), except the *SynP9*::*rbcL*::*petA* and *SynP17*::*clpP*::*ycf12* constructs, whose expression strength seemed to match expected promoter activity instead.

To study the context dependency of individual genetic elements in more detail, we next devised a series of combinatorial constructs. This series included constructs in which three different promoters of low, medium and high activity were tested in combination with the low-activity elements *clpP* (5′UTR) and *rps11* (3′UTR); three different 5′UTRs of low, medium and high activity were tested in combination with the low-activity promoter *Psyn7* and the low-activity 3′UTR *rps11*; and three different 3′UTRs of low, medium and high activity were tested together with the high-activity promoter *Prrn16* and the low-activity 5′UTR *clpP*. In all cases, the elements behaved as expected by increasing expression strength by approximately 10-fold and 50-fold for the medium- and high-activity elements, confirming their functionality across different genetic contexts (Fig. 5b).

**Extended Data Fig. 4:**
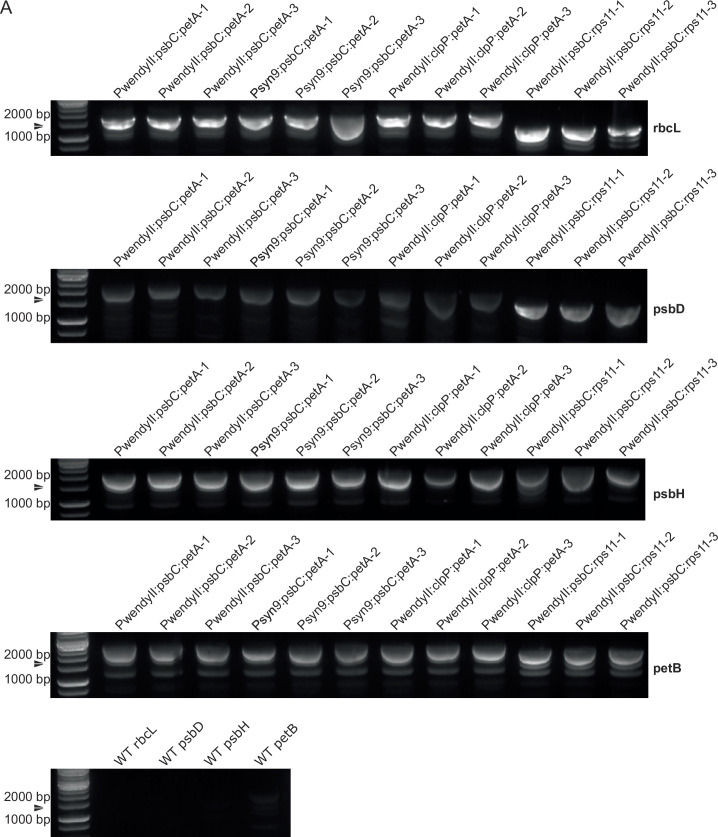
Genotyping of transplastomic strains. **a**, Gel depiction of cPCR amplification for the NanoLuc reporter gene across transplastomic strains shown in Fig. 5c, with four distinct constructs integrated at separate sites in the genome. For each construct, n_biological_ = 3. A unique band, ranging from ~1500-2000 bp, is visible in genetically modified strains across all four constructs and integration sites, but is not present in the WT. Source Data

Finally, we studied the impact of four different integration sites in the chloroplast genome (see Supplementary Text 4 for the design rules of the different integration sites). To this end, we created four different transcriptional units (two of low and two of high transgene expression strength) that we integrated into four distinct locations (Fig. 5d and Extended Data Fig. 4) in the chloroplast genome (*psbH*, *rbcL*, *psbD* and *petB*). For the *rbcL*, *psbD* and *petB* integration sites, we observed comparable expression levels among the four constructs (Fig. 5c). However, for the *psbH* integration site, we noted some differences, particularly for the construct containing the *rps11* 3′UTR. Testing for potential recombination events between the regulatory elements of our construct and adjacent native sequences (*WendyI* and *WendyII*) ruled out any rearrangements (Supplementary Figs. 6 and 7) but confirmed a recent observation that the 3′UTR at the *psbH* site is important in controlling read-through activity of neighbouring genes, which in turn can modulate transgene expression^71^. Overall, these experiments showed that most constructs behaved similarly independent of their genomic position, but they also highlighted that validating individual constructs at the *psbH* site, which is one of the most frequently used integration sites in *C. reinhardtii*, is important for quantifying actual transgene expression strength.

### Applying the tools to prototype synthetic chloroplast pathways in *C. reinhardtii*

**Fig. 6:**
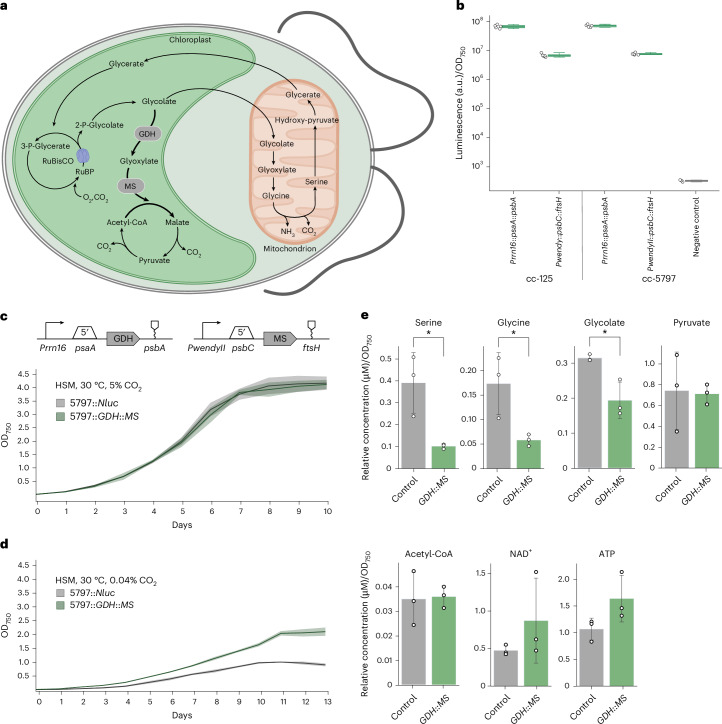
Introducing a synthetic photorespiratory bypass in the chloroplast of *C. reinhardtii.* **a**, Metabolic scheme for the previously reported synthetic photorespiration bypass. The pathway relies on the introduction of two genes (in grey): *GDH* and *MS*. **b**, NanoLuc assay for the characterization of part combinations in the cc-125 WT and cc-5797 mutant. NanoLuc signal is plotted as arbitrary units normalized to OD_750_ and compared with the control-lacking reporter (grey). For all measurements, *n*_biological_ = 5. **c**,**d**, Growth curves of engineered strains and controls at 5% CO_2_ (**c**) and ambient CO_2_ (**d**), shown as OD_750_ over time (days). The parts tested in **b** were used to express both GDH and MS. The engineered strains (5797::*GDH*::*MS*, green) reached a twofold higher final OD than the control strains (5797::*Nluc*) in ambient CO_2_ but behaved similarly in 5% CO_2_. *n*_biological_ = 3 for each curve. **e**, Intracellular metabolome analysis of engineered and control strains during the exponential growth phase. The relative concentrations (μM normalized to OD_750_) of serine, glycine, glycolate, pyruvate, acetyl-CoA, NAD^+^ and ATP are displayed for 5797::*GDH*::*MS* (green) and 5797::*Nluc* (grey). *n*_biological_ = 3 for each experiment. Significant changes according to unpaired *t*-tests are indicated by an asterisk for *P* < 0.05 (for serine, *P* = 0.023; for glycine, *P* = 0.037; and for glycolate, *P* = 0.017). Source Data

We aimed to employ our plastome engineering toolkit to prototype a multi-gene metabolic pathway in the chloroplast of *C. reinhardtii*. As a test case, we sought to introduce a recently reported synthetic photorespiratory bypass^7^ into the chloroplast that is based on two enzymes: glycolate dehydrogenase (GDH) and malate synthase (MS) (Fig. 6a). These two enzymes cause the direct decarboxylation of the photorespiration product glycolate into CO_2_, which increases CO_2_ concentrations in the chloroplast and has been reported to improve photosynthetic yield in different crops^7,72^.

**Extended Data Fig. 5:**
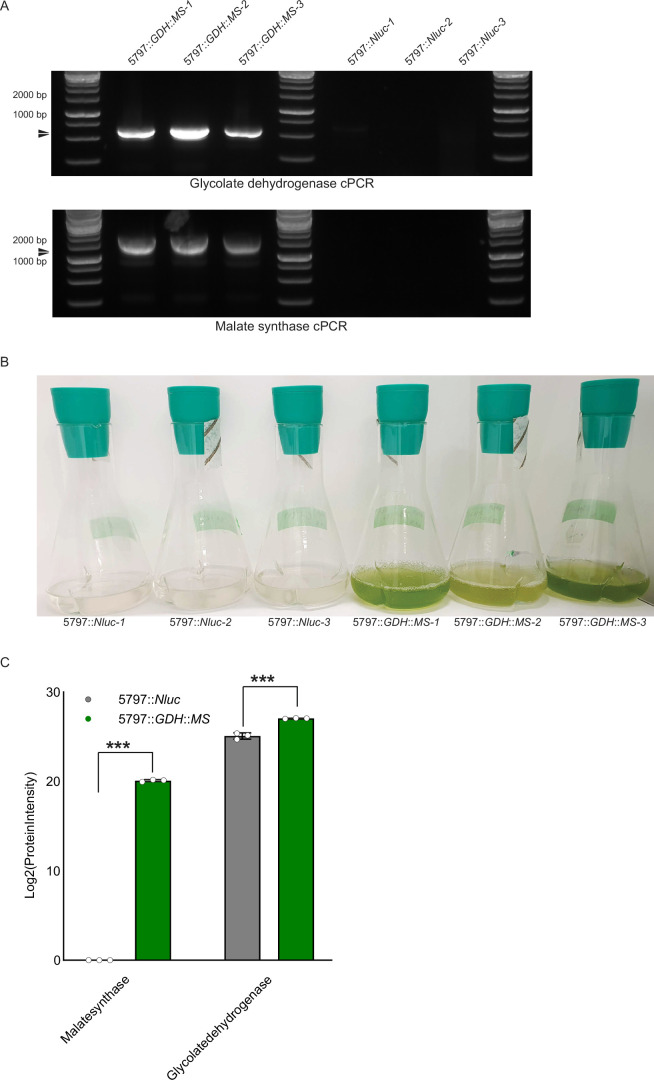
Genotyping and phenotyping of transplastomic strains containing the synthetic photorespiratory bypass. **a**, Gel depiction of cPCR amplification for the two heterologous genes glycolate dehydrogenase (GDH) and Malate synthase (MS) gene across transplastomic strains shown in Fig. 6c–k. A unique band of ~500 bp and 1500 bp, for GDH and MS respectively, is visible in genetically modified strains but is not present in the control (5797::*Nluc*). For each experiment, n_biological_ = 3. **b**, Phenotypic comparison of strains expressing GDH and MS after 15 days. The image presents three biological replicates of the 5797::*GDH*::*MS* strain, demonstrating robust and dense growth. In contrast, control strains (5797::*Nluc*) exhibit no further growth. **c**. Proteomic analysis of the modified strains. The chart compares means and standard deviations of protein levels of MS and GDH in both the 5797::*GDH*::*MS* and 5797::*Nluc* strains, revealing significantly higher protein intensities in the 5797::*GDH*::*MS* strain for both proteins. Statistical significance of unpaired t-tests is denoted by *** for a p-value < 0.001 (MS p-value < 0.000001, GDH p-value = 0.000623). Source Data

As the host for our engineering efforts, we chose a CO_2_-concentrating-mechanism-deficient and GDH-deficient *C. reinhardtii* mutant (cc-5797; genotype confirmation is shown in Supplementary Fig. 2c,d) that shows a severe growth phenotype under atmospheric CO_2_ concentrations due to increased photorespiration^73^. To express GDH and MS from the chloroplast genome, we chose four regulatory elements (5′UTR of *psaA* and 3′UTR of *psbA* for GDH; 5′UTR of *psbC* and 3′UTR of *ftsH* for MS) and validated their activity at the *psbH* integration site in cc-5797 using the NanoLuc reporter (Fig. 6b). We assembled the transcriptional units for the expression of GDH and MS, integrated these units into the *psbH* locus, confirmed integration via colony PCR (cPCR) (Extended Data Fig. 5a) and verified the presence of MS and GDH through proteomics (Extended Data Fig. 5c). During our analysis, we detected functional expression of GDH, indicating that the 5′UTR knockout of GDH was not completely tight in the cc-5797 strain; this suggests that the growth phenotype was mainly caused by deficiency of the CO_2_-concentrating mechanism.

At 5% CO_2_ concentration, the *C. reinhardtii* strain expressing MS and GDH and the negative control (that is, the cc-5797 strain expressing NanoLuc luciferase) behaved identically (Fig. 6d). However, at ambient CO_2_ concentrations, the strain with the functional photorespiration bypass showed a clear growth benefit (Fig. 6c and Extended Data Fig. 5b). While both strains had comparable doubling times (~43 h), the strain expressing MS and GDH reached almost twofold higher final ODs and threefold higher dry weight (Supplementary Fig. 3d), indicating that the pathway was functionally expressed from the chloroplast (Fig. 6c and Extended Data Fig. 5b). Moreover, intracellular metabolomics unravelled distinct differences between the engineered strain and the control strain. In the engineered strain, intracellular glycolate concentrations (as well as those of the derived amino acids glycine and serine) were significantly lower than in the control strain (Fig. 6e). In contrast, the levels of pyruvate and energy metabolites, such as ATP, were not significantly different between the two strains (Fig. 6e), supporting the hypothesis that the expression of MS and GDH had rechannelled photorespiration flux towards the synthetic glycolate metabolism. Together, these results demonstrate the possibility of prototyping metabolic pathways—in particular, new-to-nature photorespiration pathways that have been shown to be relevant in higher plants—with our platform, re-emphasizing the transferability of results between the chloroplast of *C. reinhardtii* and those of higher plants.

## Discussion

Harnessing the full potential of synthetic biology in photosynthetic eukaryotes necessitates considerable improvements in genetic engineering capabilities, with chloroplasts emerging as a prime target for such efforts. Traditionally, chloroplast engineering has been limited to a relatively small number of transplastomic strains or plant lines, which has challenged a systematic characterization of genetic parts and more complex constructs in chloroplasts^32^.

In this study, we leveraged chloroplast synthetic biology in *C. reinhardtii* by implementing an automated workflow for the generation and handling of thousands of transplastomic strains in parallel and introducing an extensive array of genetic tools to advance the possibilities in chloroplast synthetic biology. By substantially expanding the number of selection markers, reporter genes and regulatory elements for *C. reinhardtii*, our study paves the way for more ambitious algal chloroplast engineering projects. To facilitate a broader application and further development within the community, all tools, including the 300 genetic parts, are freely available through repositories and are already in use by other laboratories^27^. This set of parts provides a reference set that can be systematically extended in the future via more high-throughput screens, including pooled library approaches, which we demonstrated in our study.

Our comprehensive toolkit enables multi-transgene expression through verified part combinations and offers the possibility of more advanced transgene stacking in the plastid of *C. reinhardtii*. Chloroplast-based strategies have thus become a viable alternative for applications targeting the chloroplast, as opposed to nuclear-genome-based efforts, which have been used so far but suffer from several disadvantages, such as low transgene expression and gene silencing^74,75^. We demonstrate such an application by establishing a recently reported synthetic photorespiration pathway^7^ in *C. reinhardtii* chloroplasts that leads to a threefold higher biomass yield under selection. This proof of concept provides new possibilities for ongoing efforts to engineer plastid metabolism for the production of high-value compounds in microalgae, realize synthetic photorespiration pathways (for example, the TaCo pathway^5^ or BHAC cycle^8,9^) or even replace the Calvin cycle with new-to-nature carbon fixation pathways (for example, the Theta cycle^12^ or reverse glyoxylate shunt^76^).

While *C. reinhardtii* has a general potential to serve as a test bed for designing synthetic metabolic pathways in plastids, there are distinct differences between chloroplast gene expression in the alga and in higher evolved plants that need to be accounted for when considering knowledge transfer between the systems. For example, *C. reinhardtii* employs one multi-subunit, plastid-encoded RNA polymerase with one identified sigma factor, whereas land plants such as *Arabidopsis thaliana* possesses a plastid-encoded and a nuclear-encoded RNA polymerase with up to six sigma factors. Furthermore, mRNA processing differs between the systems. mRNA editing is absent in the alga, and *C. reinhardtii* seems (unlike tobacco) to always cleave polycistronic transcripts prior to translation^77^. Nevertheless, the plastome structure and many genetic elements show a high degree of conservation across photosynthetic eukaryotes, and the translation apparatus with its final protein synthesis output is remarkably conserved^78,79^. Genetic elements from the chloroplast of *C. reinhardtii* have been repeatedly and successfully used in various land plants, including tobacco^20^, *Arabidopsis*^46^ and poplar^44,45^, and vice versa (for example, as shown in this study). This high degree of transferability indicates the potential of our system for the rapid testing and optimization of genetic designs before a transfer of results to land plants and crops. Especially when using high-throughput routines combined with pooled library strategies, these efforts could dramatically expand the search for new phenotypes and improved chloroplast functions, which could in turn remarkably enhance the development of more efficient and more resilient chloroplast functions to cater to the needs of biotechnology and agriculture.

## Methods

### *Escherichia coli* strains, transformation and growth conditions

Bacterial cultures during the cloning phase were incubated at 37 °C in Luria–Bertani broth under shaking conditions for liquid cultures and on Luria–Bertani agar plates with 1.5% (m/v) agar for solid cultures. Antibiotic selection was applied according to the construction level: chloramphenicol (34 µg ml^−1^) for level 0 constructs (pME_Cp_UAV_GFP), ampicillin (100 µg ml^−1^) for level 1 (pME_Cp_0_7-8_003) and kanamycin (50 µg ml^−1^) for level 2 (pME_CP_0_7-8_006). Two chemically competent *E. coli* strains, DH10B and NEB Turbo, both sourced from New England Biolabs, were used for transformations, which were performed via heat shock as per the manufacturer’s instructions. Plasmid DNA was isolated using Macherey-Nagel kits designed for both mini and midi preparations, the latter being essential for chloroplast transformation due to the supercoiled nature of the DNA obtained. Terrific Broth was employed to maximize yields in midi preparations.

### *Chlamydomonas reinhardtii* strains and growth conditions

WT strains of *C. reinhardtii* were cultured on TAP medium at ~100 μmol photons per m^2^ per s, following the Chlamydomonas Resource Center’s recipe, which included Hutner’s trace elements^80^. The medium was supplemented with ampicillin (100–500 µg ml^−1^) and Difco agar (Beckton Dickson, 0.8% m/v) for solid cultures. Transformations were performed on TAP agar plates supplemented with spectinomycin (100 µg ml^−1^), tobramycin (100 µg ml^−1^) or kanamycin (200 µg ml^−1^) as required. Tobramycin antibiotic stocks were prepared freshly every time. Transformed strains were propagated on TAP medium supplemented with ampicillin (500 µg ml^−1^) and an increasing concentration of either spectinomycin (100, 300, 500 and 1,000 µg ml^−1^) or tobramycin (100, 150 and 300 µg ml^−1^) during the restreaking phase. Culture flasks were meticulously cleaned with detergent, rinsed thrice with deionized water and autoclaved with Milli-Q water to eliminate any residual detergent (SOMAT). The study predominantly used the cc-125 mt+ mutant strain from the Chlamydomonas Resource Center, as well as cc-5797, a double mutant for *GDH* and *cia5*, which is a global regulator for the CO_2_ concentrating mechanism in *C. reinhardtii*.

For growth curves of cc-5797, pre-cultures were grown for nine days in HSM, according to the Chlamydomonas Resource Center (HSM + Spec_100_, 5% CO_2_, 30 °C), and used for inoculation of cultures at OD_750_ = 0.05. Growth curves were carried in HSM supplemented with Spec_100_ at 0.04% CO_2_ and 30 °C, or HSM + Spec_100_ at 5% CO_2_ and 30 °C for the control. Cultures for Supplementary Fig. 3 were grown in similar conditions without antibiotic supplementation. Each day, 1-ml samples were taken, and OD_750_ was measured.

Following these growth curves, average dry weight was measured. Cells were harvested via centrifugation at 3,000 *g* for 10 min, the supernatant was discarded and the pellets were dried for 48 h at 80 °C. The 15-ml Falcon tubes used for harvesting were weighed before and after harvest to account for their mass. The difference was used to estimate average dry weight (*n*_biological_ = 3).

### Chloroplast transformation

Chloroplast transformation in *Chlamydomonas* was executed using a Bio-Rad PDS/1000 He (Bio-Rad) gene gun. Briefly, cells were grown in 50 ml of TAP medium for two days before inoculation into two 400-ml TAP cultures with 10–15 ml of the pre-culture. These cultures were grown to a density of 10^7^ cells per ml and harvested via centrifugation at 3,000 *g* for 10 min, and the pellets were resuspended in 50 ml of fresh TAP medium to disperse clumps before a second centrifugation at 3,000 *g* for 10 min. The pellets were resuspended in 10 ml of TAP medium, and 500 µl was used for plating on TAP agar plates supplemented with the appropriate selection antibiotics. The plates were dried, uncovered, for at least one hour. 60 mg of gold particles (0.6 µm, Bio-Rad) was weighed and washed with 100 µl of 100% ethanol. The gold particles were then washed twice (1 ml of water) and resuspended in 1 ml of water. 50 µl of washed gold particles were coated with ~10 μg of supercoiled plasmid DNA prepared via midi preparation for each construct, using 50 µl of 2.5 M CaCl_2_ and 10 µl of 0.1 M spermidine. All reagents were kept on ice throughout the protocol to prevent particle clumping. A pure ethanol wash was carried out, and DNA-bound gold beads were resuspended in 60 µl of pure ethanol and used for biolistics, following Bio-Rad’s instructions. 1,100-psi rupture disks were used. The plates were incubated in the dark for recovery (~5–10 μmol photons per m^2^ per s) for 24 h and moved to high light for selection (~100 μmol photons per m^2^ per s).

Chloroplast transformation experiments were conducted as previously described with minor modifications. Transformed cells were incubated in the dark at approximately 5–10 μmol photons per m^2^ per s for a recovery period of 24 h. The following day, 1 ml of the recovery culture was used to flush each cell lawn, and 400 μl of the transformed cells was plated on TAP media supplemented with either tobramycin (100 μg ml^−1^) or spectinomycin (100 μg ml^−1^).

After two weeks of incubation, photographs were captured using a PIXL camera, and colony-forming units were quantified to assess transformation efficiency.

### Automated picking, restreaking and re-arraying

For automated propagation and selection, growth on solid media versus liquid media was compared. Growth on solid media proved more robust and reproducible than liquid growth in multi-well plates. A PIXL robot (Singer Instruments) facilitated semi-automatic colony picking across various array formats, including 96, 384 and 1,536 configurations, with optimized parameters ensuring consistent selection of *C. reinhardtii* colonies as outlined in Supplementary Text 2. For each construct, 16 colonies were selected when possible. Approximately 3,780 colonies were initially picked to generate the strain collection, from which a total of 3,150 strains were selected using the PIXL methodology. The Rotor (Singer Instruments) played a crucial role in managing arrays of transformants, adjusting antibiotic concentrations and preparing assay plates. It was employed for restreaking tasks and for dividing larger 384-well plates into smaller 96-well measurement arrays, using pin formats compatible with both plate sizes. A total of 19 plates were generated and maintained in either 96 or 384 format. Each plate was restreaked once a week for three weeks to achieve homoplasmy, followed by biweekly restreaking for maintenance to prevent colony cross-contamination. By the end of the study, each plate had been restreaked an average of 50 times.

### Colony-forming unit quantification

For all pictures obtained after the transformation for selection marker efficiency assessment, image processing and analysis were performed using Fiji v.2.16 (ref. ^81^). The particle areas were determined from standard agar plate pictures using a Fiji macro (https://github.com/ChlamyMarburg/ChloroplastTools/blob/main/pipeline/Colony_count.ijm). This approach was used for the analysis of the 24 plates analysed in Supplementary Fig. 4 (2 controls, 11 spectinomycin plates and 11 tobramycin plates).

### Part design

For our *Chlamydomonas* Chloroplast Modular Assembly System (CHLOROMODAS), we employed the Phytobrick standard for the design of level 0 parts^48^, as previously used in other plant and *Chlamydomonas* MoClo toolkits^36,47,82^. To assemble multiple transcription units, we adapted the standard of the Marburg collection^83^, except for exchanging the 3′ Connector/Backbone Part overhang from AGCT to AGAC to enhance assembly efficiency as determined by the NEB ligase fidelity tool^84^. All level 0 parts were cloned into a standard universal acceptor vector.

For the rational design of 5′UTR and 3′UTR level 0 parts, we used previously reported RNA-seq and small RNA data^62^ from *C. reinhardtii* chloroplasts for the annotation of transcription start sites and RNA-binding sites of trans-factors. Additionally, RNA secondary structures of 3′UTRs were analysed using ViennaRNA Web Services to identify necessary stabilizing hairpin structures. The design of endogenous promoter parts involved examining RNA-seq data to identify consensus −35 and −10 promoter sequences in the plastome prior to the transcription start site. Suitable candidates for IEEs were identified by analysing co-transcription data from a list of genes in the chloroplast and searching for RNA-binding protein footprints within these sequences, indicating potential post-transcriptional processing success.

Several parts in our toolkit underwent multiple design–build–test cycles using reporter gene assays to determine the minimal sequence required for optimal function, such as for the *rrn16* promoter and *rbcL* 3′UTR. Codon usage for coding sequences was optimized using the Geneious codon optimization tool on the basis of the chloroplast codon usage table for *C. reinhardtii* (available at https://www.kazusa.or.jp). Original sequences for *GDH* and *MS* were sourced from South et al.^7^ and subsequently optimized.

Level 0 parts were either amplified from the cc-125 genome or synthesized as fragments by Twist Bioscience. Smaller parts (<100 bp) were cloned using annealed oligonucleotides.

A list of all sequences designed and tested in this study can be found in Supplementary Table 5.

### Part assembly

Vectors and inserts were standardized to concentrations of 20 fmol. The assembly of level 0 and level 2 constructs used the type IIS restriction enzyme BsmBI, while BsaI was employed for the assembly of level 1 constructs. These assembly reactions were facilitated using the NEBridge Golden Gate Assembly Kit from New England Biolabs for both BsaI and BsmBI enzymes (NEB #E1601 and NEB #E1602). The reaction mixtures, prepared without specifying volumes, included a vector at 10 fmol, inserts at 20 fmol, the respective NEBridge Golden Gate Enzyme Mix (BsaI or BsmBI), T4 DNA Ligase Reaction Buffer and nuclease-free water to complete the mixture. BsaI-based assembly reactions underwent 50 cycles of temperature alternations, with periods at 37 °C followed by cooling to 16 °C. In contrast, BsmBI-based reactions alternated between 42 °C and 16 °C. The final steps for both methods involved a brief incubation at 60 °C and a final heat inactivation at 80 °C before the transformation into competent bacterial cells (5 µl of the Golden Gate mixture). A manual on how to use our system can be found in Supplementary Text 6.

### Fluorescence assays and fluorescence-activated cell sorting

For chloroplast fluorescence analysis in *C. reinhardtii*, transformed and WT colonies from 384-well arrays were resuspended in nuclease-free water in black 384-well Greiner Bio-One plates. OD was measured at 750 nm for normalization. Fluorescence quantification used a microplate reader (Tecan Infinite 200 Pro) with specific settings for mScarlet-I (excitation, 569 nm; emission, 605 nm), mVenus (excitation, 515 nm; emission, 545 nm) and tdTomato (excitation, 554 nm; emission, 585 nm).

Fluorescence-activated cell sorting analysis was performed using a SONY SH800S Cell Sorter to assess fluorescent protein expression. WT and mScarlet-I-expressing strains underwent a two-day pre-culture and a two-day culture in TAP medium, with spectinomycin (100 µg ml^−1^) added for transformants. The samples were diluted tenfold in TAP for optimal sorting. The SONY SH800S settings were adjusted to accurately detect the fluorescent markers according to the manufacturer’s instructions, which can be found in Supplementary Text 2.

### High-throughput pipeline for luminescence measurements

To facilitate the high-throughput analysis of regulatory elements, we established a detailed pipeline, as illustrated in Fig. 1. This approach incorporated NanoLuc luciferase assays from Promega for the quantitative assessment of regulatory element functionality. Initially, transformants were propagated on 384-well agar plates. These plates were then segmented into four 96-well solid-agar arrays with the aid of the Rotor robot, enhancing the biomass by replicating each transformant into 5 × 5 squares. This cultivation extended over a five-day period under continuous light, followed by a three-day period in either continuous light or dark conditions depending on the experimental requirements. Post-cultivation, colonies were carefully resuspended in 30 µl of water in 384-well ECHO 525 plates (LABCYTE). OD measurements at 750 nm were conducted to ascertain cell density. To ensure standardized measurements across seven replicates per construct, a custom Python pipeline was created. This pipeline leverages a configuration file to set the liquid handling parameters for the ECHO 525 and define experimental variables, including the numbers of technical and biological replicates. The objective was to achieve an OD_750_ of 0.01 for each replicate. To this end, the script uses the previously measured absorbance data to generate pipetting instructions for the ECHO 525 liquid handler, which include automated modifications to the plate layout and the selection of source wells. The dilutions were created in white 384-well plates, with alternate wells left empty to minimize light interference between replicates in the subsequent luminescence measurements. The luminescence assay was initiated by adding 10 µl of NanoLuc substrate solution to each well (PROMEGA, N1110). Following a five-minute incubation at room temperature, luminescence was measured using a plate reader. Afterwards, the data processing pipeline used a JSON mapping file to link each measurement with the corresponding construct. By integrating the ECHO pipetting instructions with the absorbance and luminescence data, a comprehensive experiment overview was automatically compiled. This document includes normalized luminescence values for each construct, corrected on the basis of the OD_750_ measurements and dilution factors to precisely reflect the activity of regulatory elements. This overview was subsequently used for further analysis and data visualization.

### Selection marker cross-resistance assay

SBS plates for cross-resistance assays between tobramycin and spectinomycin were generated from a TAP + tobramycin (200 µg ml^−1^) plate and restreaked on TAP + tobramycin (200 µg ml^−1^) and TAP + spectinomycin (500 µg ml^−1^), respectively. The plates were grown for four days before a picture was taken.

### Metabolome extraction

Daily 1-ml samples from the growth curve were mixed with an equal volume of 70% methanol pre-cooled at −70 °C and then centrifuged, and the supernatant was discarded before the pellets were frozen at −70 °C. In a chemical fume hood and with proper PPE (gloves and glasses), 200 µl of 50% methanol in TE buffer (10 mM Tris-HCl, 1 mM EDTA, pH 7.0) was added to each pellet. Mechanical disruption was performed in a bead mill (Bead Bug) using 0.375 g of acid-washed glass beads (500 µm) for two 30-s cycles at maximum power, with the samples cooled on ice between cycles. Following disruption, 200 µl of 100% chloroform was added, and the mixture was vortexed for 30 s. After centrifugation at 13,000 *g* for 10 min at −10 °C, the upper phase was extracted with 1-ml syringes, slowly filtered through PTFE filters into pre-cooled Eppendorf tubes and transferred into metabolomics vials for analysis, with careful handling to avoid warming and contamination.

### Metabolome analysis

Quantitative determination of the targets was performed using a liquid chromatography–tandem mass spectrometry system, integrating chromatographic separation and mass spectrometry for precise analysis. Chromatographic separation was achieved on an Agilent Infinity II 1290 HPLC system, employing columns of varying specificities (Kinetex EVO C18, ZicHILIC SeQuant and SeQuant ZIC-pHILIC) with particle sizes ranging from 3 to 5 μm and pore sizes of 100 Å (Phenomenex), connected to guard columns of similar specificity. The flow rates varied between 0.1 and 0.3 ml min^−1^, with mobile phases tailored to each experiment: CoA esters (1) 50 mM ammonium formate in water and 100% methanol, organic acids (2) 0.1% formic acid in water and methanol, amino acids (3) 0.1% formic acid in 99:1 water:acetonitrile and acetonitrile:water, and ATP and NAD^+^ (4) 10 mM ammonium acetate in water and acetonitrile, all adjusted to specific pH levels and temperatures. Injection volumes were set at 1 or 2 µl, with mobile phase profiles designed to optimize separation over varying times and gradients.

Mass spectrometry analysis was conducted using an Agilent 6495 ion funnel mass spectrometer, operating in both positive and negative ionization modes with specific settings for ESI spray voltage, nozzle voltage, sheath gas temperature, nebulizer pressure and drying gas temperature to ensure optimal detection and quantification. Compounds were identified and quantified on the basis of their mass transition, retention time and peak area compared to standards, using MassHunter software. Relative abundance and absolute concentrations were determined on the basis of peak areas and external standard curves, respectively. The optimization of mass transitions, collision energies, cell accelerator voltages and dwell times was achieved using chemically pure standards, with the parameter settings detailed in Supplementary Tables 1–4 for each experimental variation.

### Proteome extraction

Cells were cultured in HSM under a high CO_2_ concentration (5%) for four days until reaching a density of 10^7^ cells per ml or the exponential phase in a 50 ml pre-culture. The cultures were centrifuged at 3,000 *g* for 10 min at room temperature. The supernatant was removed, and the cell pellet was washed with 30 mM Tris-HCl (pH 7.9), followed by a second centrifugation. The pellet was then either used immediately or stored at −80 °C. Cell pellets were lysed by adding 500 µl of Tris-HCl with protease inhibitors and 0.5 µg µl^−1^ lysozyme (Sigma-Aldrich), incubating at 25 °C for 1 h. Then, 400 µl of 2% sodium deoxycholate with 10 mM dithiothreitol was added. After three freeze–thaw cycles, the pellet was sonicated, heated at 90 °C for 30–45 min and sonicated again. The lysate was centrifuged at 14,000 *g* for 30 min at 4 °C, and the supernatant was collected for further processing. The supernatant was mixed with six volumes of cold (−20 °C) acetone and incubated overnight at −20 °C. After centrifugation at 5,000 *g* for 20 min at 4 °C, the supernatant was discarded. The pellet was washed twice with cold methanol, dried and reconstituted in 200 µl of 0.5% sodium deoxycholate. Protein concentration was determined using BCA and pre-diluted BSA (Pierce BCA, Thermo Fischer Scientific) following the manufacturer’s protocol, and 50 µg of total protein was used for further protein purification via SP3 (ref. ^85^) as described previously with minor modifications. In short, proteins were bound to 4 µl of SP3 beads (40% v/v) in the presence of 70% acetonitrile for 15 min at room temperature, followed by two washes of the beads with 70% ethanol and an additional wash with acetonitrile. After removal of the supernatant, 1 µg of trypsin in 100 mM NH_4_HCO_3_ was added to the beads and digested with shaking overnight at 30 °C. Following digestion, the beads were separated, and peptide-containing supernatant was collected and further purified and desalted via C18-solid-phase extraction using Chromabound spin columns (Macherey-Nagel). Cartridges were prepared by adding acetonitrile, followed by equilibration with 0.1% TFA. Acidified peptides were loaded on equilibrated cartridges, washed with buffer containing 5% ACN and 0.1% TFA and finally eluted with 50% ACN and 0.1% TFA.

### Proteome analysis

Dried peptides were reconstituted in 0.1% trifluoroacetic acid and then analysed using liquid chromatography–mass spectrometry carried out on an Exploris 480 instrument connected to an Ultimate 3000 RSLC nano and a nanospray flex ion source (all Thermo Scientific). Peptide separation was performed on a reverse phase HPLC column (75 µm × 42 cm) packed in-house with C18 resin (2.4 µm; Dr. Maisch). The following separating gradient was used: 94% solvent A (0.15% formic acid) and 6% solvent B (99.85% acetonitrile, 0.15% formic acid) to 25% solvent B over 97 min, and an additional increase of solvent B to 35% for 35 min at a flow rate of 300 nl min^−1^. Raw mass spectrometry data were acquired on an Exploris 480 (Thermo Scientific) in data independent acquisition (DIA) mode. Peptides were ionized at a spray voltage of 2.3 kV, with the ion transfer tube temperature set at 275 °C. The funnel RF level was set to 45. For DIA experiments, full mass spectrometry resolutions were set to 120.000 at *m*/*z* 200 and full mass spectrometry, and the automatic gain control target was 300% with an IT of 50 ms. The mass range was set to 350–1,200. The automatic gain control target value for fragment spectra was set at 3,000%. 60 windows of 4 Da were used with an overlap of 1 Da (*m*/*z* range, 550–678). Resolution was set to 15,000 and IT to 50 ms. Stepped HCD collision energy of 27%, 30% and 32% was used. MS1 data were acquired in profile, MS2 DIA data in centroid mode. Analysis of DIA data was performed using DIA-NN v.1.8 (ref. ^86^), a UniProt protein database from *C. reinhardtii* with added sequences to generate a dataset-specific spectral library for the DIA analysis. The neural-network-based DIA-NN suite performed noise interference correction (mass correction, RT prediction and precursor/fragment co-elution correlation) and peptide precursor signal extraction of the DIA-NN raw data. The following parameters were used: full tryptic digest was allowed with two missed cleavage sites, and oxidized methionines and carbamidomethylated cysteins. ‘Match between runs’ and ‘remove likely interferences’ were enabled. The precursor FDR was set to 1%. The neural network classifier was set to the single-pass mode, and protein inference was based on genes. Quantification strategy was set to any LC (high accuracy). Cross-run normalization was set to RT-dependent. Library generation was set to smart profiling. DIA-NN outputs were further evaluated using the SafeQuant^87,88^ script modified to process DIA-NN outputs.

### Immunoprecipitation analysis

For protein analysis via SDS–PAGE, samples were prepared by centrifuging 8 ml of culture with 4.10^7^ cells at 4,500 rpm and 4 °C for 2 min, decanting the supernatant and resuspending the cells in the remaining medium for transfer to 1.5-ml Eppendorf tubes. After a subsequent centrifugation for 30 s at 4 °C, the supernatant was removed, and the cells were resuspended in 120 µl of 0.1 M Na_2_CO_3_ carbonate buffer, followed by the addition of 110 µl of 5% SDS solution. The samples were then mixed vigorously, heated at 95 °C for 45 s, snap-frozen in liquid nitrogen and stored at −20 °C.

Protein concentrations were determined using the Lowry assay. Solutions A and B for the Lowry assay were prepared and mixed and were then stored at 4 °C. Protein standards were prepared by diluting 1 mg ml^−1^ BSA in ddH_2_O, and samples were prepared from the −20 °C freezer, with triplicates of 90 µl water and 10 µl supernatant. After adding 1 ml of Lowry’s solution and incubating for 15 min at 25 °C, 100 µl of 1× Folin reagent was added, and the samples were incubated for an additional 20 min. Absorbance was measured at *λ* = 750 nm to determine protein concentrations.

SDS–gel electrophoresis was performed using prepared solutions for stacking (3%) and separating (10%) gels. Gels were assembled, polymerized and loaded with samples prepared with 30 µg of protein in a final volume of 60 µl, including 4× loading buffer. Electrophoresis was conducted at 160 V until the chlorophyll was about to exit the gel. Subsequently, the samples were prepared for western blotting using Whatman paper and nitrocellulose membranes. The membranes were blocked with 5% non-fat dry milk in TBST (20 mM Tris, 150 mM NaCl, 0.1% Tween-20) for 1 h at room temperature and incubated overnight at 4 °C with primary antibodies specific to the target tag: mouse monoclonal antibody (Sigma) for the HA tag, mouse monoclonal antibody (Sigma) for FLAG and mouse monoclonal antibody (Invitrogen) for Myc. Following primary antibody incubation, the membranes were washed three times with TBST and secondary antibodies for 1 h at room temperature, and proteins were analysed using a gel imager.

### Data analysis, plotting and statistical analysis

Data analysis and visualization were performed using Python v.3.10.5. For parsing and processing, the pandas library (v.1.4.3)^89^ was used. Visualization of the data was conducted using the Plotly library (v.5.9.0)^90^. Some data are displayed as box plots and adjacent individual data points on a decadic logarithm scale. The midlines of the box plots represent the median, and the boxes’ upper and lower limits represent the first and third quartiles. The whiskers correspond to the boxes’ edges ±1.5 times the interquartile range.

Bar graphs, specifically in Figs. 2 and 6, depict mean values with error bars indicating standard deviation, providing a clear visual summary of the data’s central tendency and variability. For the analysis of fluorescence-activated cell sorting data, the FlowCal library (v.1.3.0)^91^ was employed, presenting data on a logicle scale, a method detailed in ref. ^92^.

Data analysis and visualization for Supplementary Figs. 3 and 4 were performed using R v.4.4.2. Visualization of the data was conducted using the ggplot2 package^93^. The data are displayed as bar plots or box plots and adjacent individual data points on a linear scale.

Metabolomics data underwent statistical examination using the scipy library (v.1.8.1)^94^. The significance of these analyses was based on unpaired *t*-tests. Growth curve data were visualized using mean values with shading to represent the standard error. Doubling times for each strain were calculated using GraphPad Prism v.10 and its Exponential (Malthusian) Growth function, providing the doubling time over the first seven days (exponential phase).

### Fluorescence microscopy

For imaging, cells were grown to the exponential phase (2–8 × 10^6^ cells per ml) in TAP media. Then, 10 μl of cell suspension was overlaid with 30 μl of 1% TP-low-melting point agarose in µ-Slide 18-well chambered coverslips (ibidi). All fluorescence images were captured using a Zeiss LSM880 confocal microscope in Airyscan mode using a ×63 1.4 numerical aperture Plan-Apo oil-immersion lens (Carl Zeiss). Bright-field images were captured using the microscope T-PMT output channel. The specific settings are given in Supplementary Text 1.

### Colony PCR analysis

*Chlamydomonas* strains were subjected to cPCR analysis. The cells were processed in 384 Greiner PCR plates, each well containing 25 μl of an extraction buffer composed of 10 mM Tris, 5 mM EDTA and 0.01% Triton100, adjusted to pH 8. The plates underwent vortexing, followed by a boiling step at 100 °C for 10 min. Subsequently, the plates were vortexed again and then cooled on ice for 5 min before centrifugation. The supernatant obtained from this process was diluted in a 1:5 ratio with the extraction buffer and used as the DNA template for PCR reactions.

For the PCR amplification, the 384 Greiner PCR plates were prepared by adding 0.5 μl of primer (10 μM) to each well, followed by 8 μl of Phusion High Fidelity Master Mix (New England Biolabs, M0531L) or OneTaq 2X Master Mix (New England Biolabs, M0482L) according to the manufacturer’s instructions. These steps were facilitated using the ECHO 525 liquid handling system. To this mixture, 1 μl of the 1:5 diluted DNA template was added. The PCR reaction was then performed as per the manufacturer’s guidelines, with the annealing temperature being adjusted according to the specific primer combination used. GeneRuler 1 kb DNA ladder (Thermo Fischer Scientific) was used for every gel shown in the study.

cPCR confirming NanoLuc insertion in each strain used for part characterization can be found in Supplementary Figs. 1 and 2. The primers used for these cPCRs were designed to have one primer binding in the NanoLuc cassette (oRI343) and one primer binding in the *psbH* homology (oRI643), ensuring the presence of the coding sequence in the correct locus (Supplementary Figs. 1 and 2). cPCR confirming the integration of tobramycin can be found in Extended Data Fig. 2c. The primers used for that cPCR were designed to have one primer binding in the tobramycin cassette (tobra_rv_58_56_2) and one in the *psbH* homology (oRI643).

cPCR performed to assess the presence of recombination between the *Wendy* regulatory elements with the genome were performed using the primer pairs WendyI_fwd/WendyI_rv, WendyI_fwd2/WendyI_rv2 and WendyII_fwd/WendyII_rv, WendyII_fwd2/WendyII_rv2 (Supplementary Figs. 6 and 7).

Homoplasmy was tested via cPCR only on some strains, as on average 80% of strains reached homoplasmy after a minimum of three weeks. Cells were processed in the same way as described before; however, the primers were designed to bind outside of the homology regions, to allow a comparison between transplastomic and WT strains. For the *psbH* locus, the primers psbH_cPCR_homology_fwd and psbH_rv_58_56 were used.

A list of all primers can be found in Supplementary Table 6, including all versions of the primers tested.

### Design, construction and chloroplast transformation of a degenerated promoter library

A 90-bp single-stranded degenerated oligonucleotide was designed and ordered. This oligonucleotide has 25-bp homologies at the 5′ and 3′ ends to an already pre-assembled vector. The oligonucleotide also contains 40 bp of the core *rrn16* promoter, but with nine sites that are changed to a degenerated base pair. The pre-assembled vector contains a spectinomycin resistance and a mScarlet-I expression cassette, consisting of a *psaA* 5′UTR, the mScarlet-I coding sequence, the *psbA* 3′UTR and 5′ and 3′ homology flanks for the integration in the *psbH* locus. The vector was then PCR amplified and subsequently used for the NEB HiFi assembly together with the degenerated oligonucleotide. Afterwards, all red colonies were pooled in 500 ml of Terrific Broth. After 5 h of incubation, the DNA of the entire library was isolated via the NucleoBond Xtra Maxi Kit (Macherey Nagel). This pooled DNA isolation was used for the subsequent chloroplast by coating the plasmid mixture on the gold nanoparticles. After the chloroplast transformation, 768 colonies were picked and restreaked four times (once a week) to reach homoplasmy.

### Reporting summary

Further information on research design is available in the Nature Portfolio Reporting Summary linked to this article.

## Supplementary information

Supplementary Information Supplementary Figs. 1–14, Texts 1–6 and Tables 1–7.

Reporting Summary

## Source data

Source Data Figs. 2–6 and Extended Data Figs. 2–5 Unprocessed images of DNA and protein gels for Fig. 2 and Extended Data Fig. 2, and statistical source data for Figs. 2–6 and Extended Data Figs. 2–5.

## Data Availability

The raw mass spectrometry data have been deposited in the ProteomeXchange database under accession code PXD051642. All plasmid sequence information has been deposited in GenBank, and the accession numbers are given in Supplementary Table 7. Source data are provided with this paper. The plasmids used in this study are available through Addgene (https://www.addgene.org/kits/erb-chloromodas/).

## Code availability

The automation and data analysis scripts as well as the GenBank files of all the plasmids used in this study (including the entire chloroplast MoClo collection) are available via GitHub at https://github.com/ChlamyMarburg/ChloroplastTools.
