# Supplementary material for: A modular high-throughput approach for advancing synthetic biology in the chloroplast of Chlamydomonas
Source: Nat Plants. 2025 Nov 3;11(11):2332–49. doi: 10.1038/s41477-025-02126-2 (PMC12626891; doi:10.1038/s41477-025-02126-2)
Supplement: Supplementary file 1 — Supplementary Figs. 1–14, Texts 1–6 and Tables 1–7. [file 41477_2025_2126_MOESM1_ESM.pdf]

nature plants

Supplementary information https://doi.org/10.1038/s41477-025-02126-2

A modular high-throughput approach for advancing synthetic biology in the chloroplast of *Chlamydomonas*

In the format provided by the authors and unedited

Inventory of Supplementary Information

Supplementary Figures

Supplementary Figure 1: Gel analysis of cPCR amplification for NanoLuc

Supplementary Figure 2: cPCR and sequencing for strain genomic characterization

Supplementary Figure 3: Growth and biomass production analyses of transplastomic strains containing the synthetic photorespiration bypass

Supplementary Figure 4: Comparison of the selection efficiency for the Spectinomycin and Tobramycin antibiotic resistance markers

Supplementary Figure 5: Cross-resistance assessment between Tobramycin and Spectinomcin markers

Supplementary Figure 6: Gel analysis of cPCR amplification of the WendyI locus

Supplementary Figure 7: Gel analysis of cPCR amplification of the WendyII locus

Supplementary Figure 8: Homoplasmy cPCR amplification of multiple mScarlet-I and Nanoluc transformants and isolated clones

Supplementary Figure 9: Source data for Supplementary Figure 1

Supplementary Figure 10: Source data for Supplementary Figure 2

Supplementary Figure 11: Source data for Supplementary Figure 4

Supplementary Figure 12: Source data for Supplementary Figure 6

Supplementary Figure 13: Source data for Supplementary Figure 7

Supplementary Figure 14: Source data for Supplementary Figure 8

Supplementary Texts

Supplementary text 1: Fluorescence microscopy settings for each fluorophore, chlorophyll and brightfield

Supplementary text 2: PIXL colony detection parameters

Supplementary text 3: FACS analysis settings

Supplementary text 4: criteria for selecting integration sites

Supplementary text 5: Design and in-depth analysis of synthetic chloroplast promoters

Supplementary text 6: Manual for the Chloroplast Modular Cloning System

Supplementary Tables

Supplementary table 1: Mass Spectrometry parameters for CoA

Supplementary table 2: Mass Spectrometry parameters for organic acids

Supplementary table 3: Mass Spectrometry parameters for amino acids

Supplementary table 4: Mass Spectrometry parameters for energy metabolites

Supplementary table 5: Sequences of all the parts of interest

Supplementary table 6: Primers used in this study

Supplementary table 7: List of Genbank Accession code per construct

Supplementary Figures

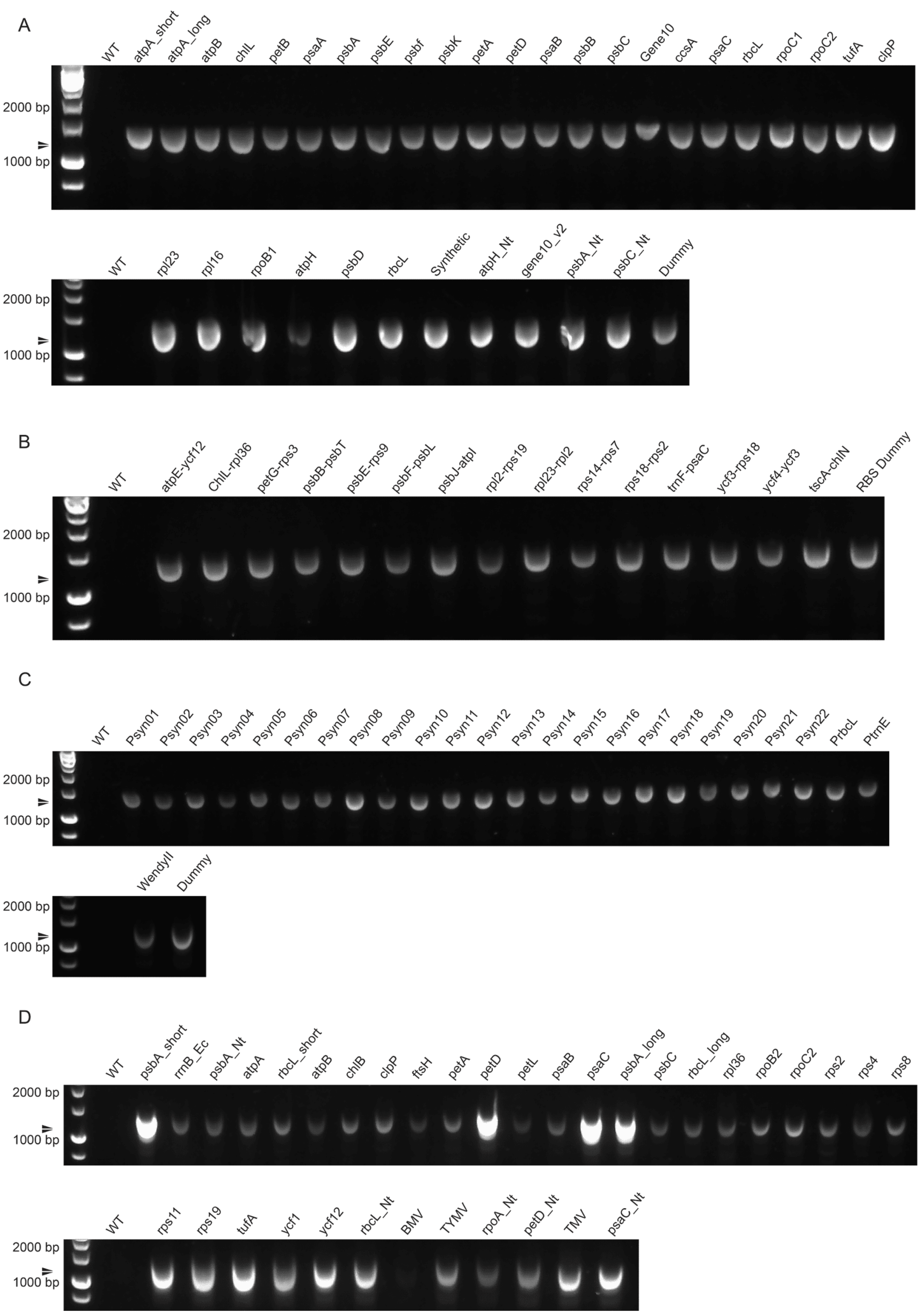

Supplementary figure 1: Gel analysis of cPCR amplification for the NanoLuc reporter gene in transplastomic strains, characterized in Figures 3-4, featuring one representative colony for each construct. A distinct band, approximately 1100-1300 bp in size, is observed in the genetically modified strains, absent in the WT. **a-d,** cPCR for NanoLuc in strains containing various 5'UTRs, IEEs, Promoters, and 3'UTRs respectively.

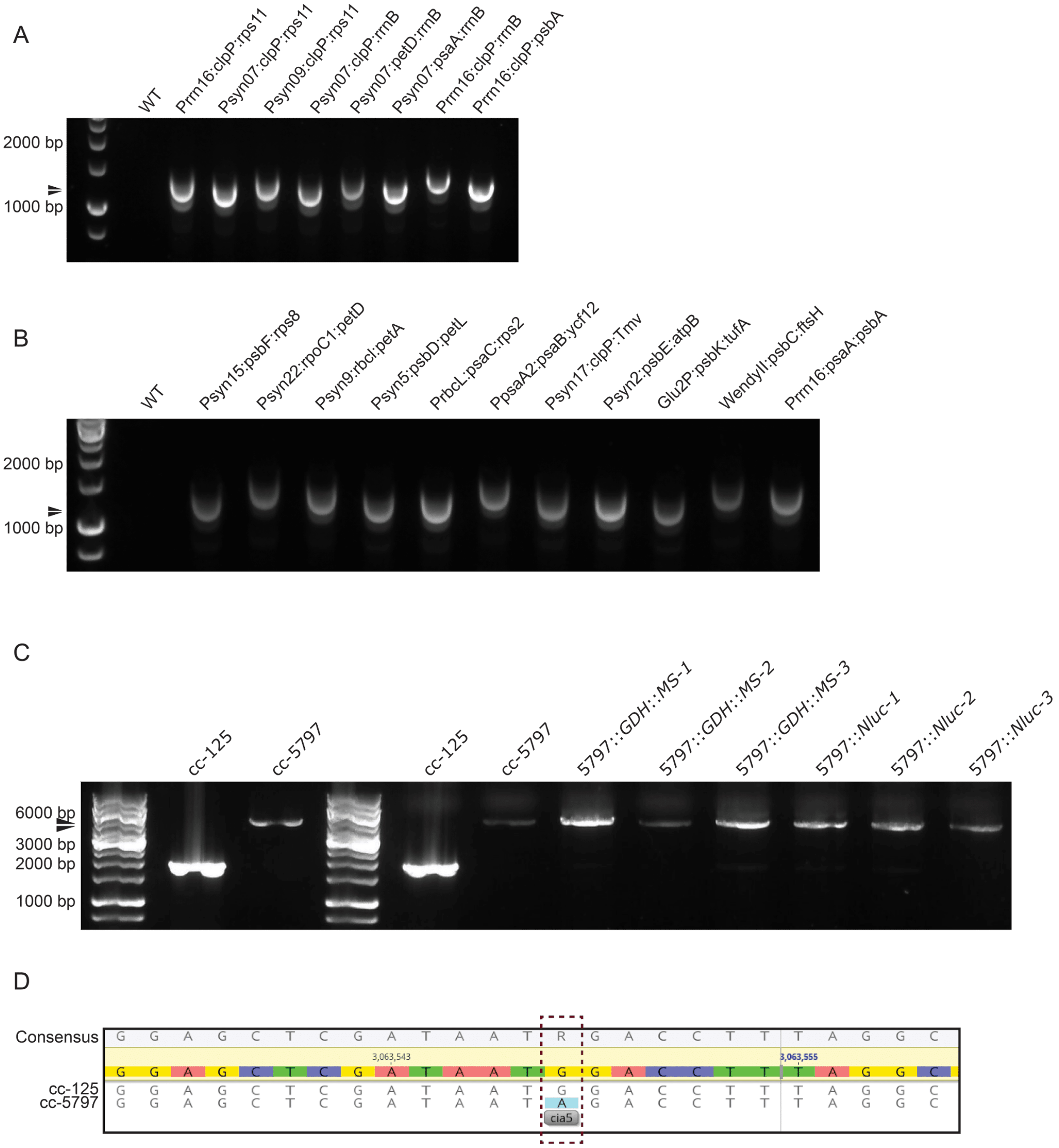

Supplementary figure 2: **cPCR and sequencing for strain genomic characterization**. **a-b,** Gel analysis of cPCR amplification for the NanoLuc reporter gene in transplastomic strains, characterized in Figures 5, featuring one representative colony for each construct. A distinct band, approximately 1100-1300 bp in size, is observed in the genetically modified strains, absent in the WT. **c** cPCR analysis of the glycolate dehydrogenase gene in cc-125 WT, cc-5797 mutant, and all engineered strains depicted in Figure 6. The transition from a 2000 bp band in cc-125 to a 6000 bp band in cc-5797, attributable to an insertion cassette. This band shift is consistently present in all engineered cc-5797 strains, verifying the insertion cassette's presence across these variants. **d,** Sequencing analysis reveals a point mutation at the cia5 locus in the nuclear genome of the cc-5797 mutant, absent in the cc-125 WT. A dark red dotted-lined box highlights this mutation, confirming the previously reported genotype**.**

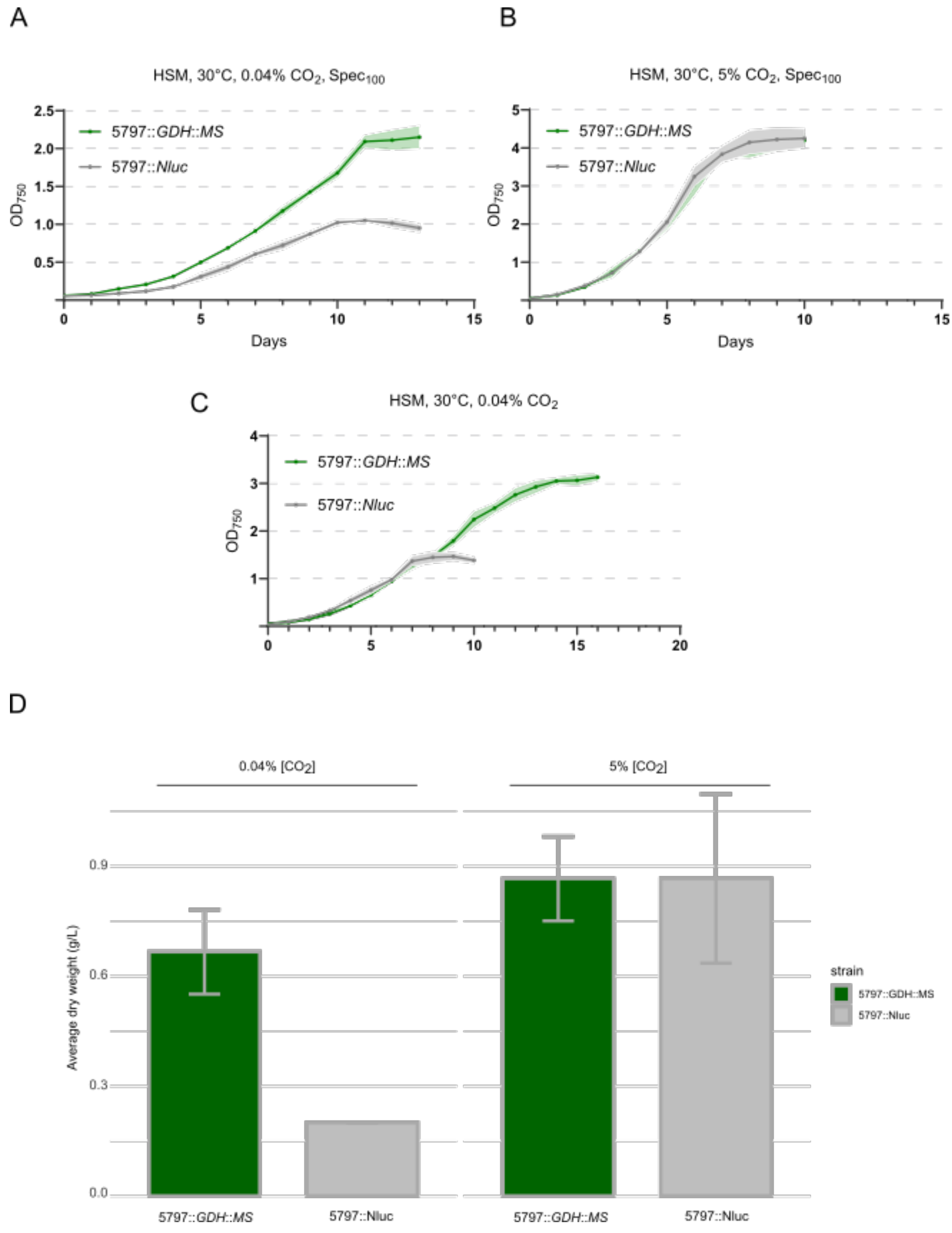

Supplementary figure 3: **Growth and biomass production analyses of transplastomic strains containing the synthetic photorespiration bypass. a-c,** Growth curve of engineered strains and control at ambient CO_2 (**a**) and 5% CO_2 (**b**) both with selection pressure (Spectinomycin 100 µg/mL) and ambient CO_2 without selection pressure (**c**), all shown as OD_{750} over time (days). Engineered strains (5797::*GDH*::*MS*,in green) reach a two-fold final OD over the control strains (5797::*Nluc* in grey) in ambient CO2 but behave similarly in 5% CO2. The same phenotype is observed when removing the selection pressure in c. $n_{biological}$=3 for each curve. **d,** Mean and standard deviations of measured dry weight of the south engineered strains after 13 days of growth for $n_{biological}$=3. The average dry weight is comparable for both control strains (5797::*Nluc* in grey) and engineered strains (5797::*GDH*::*MS* in green) in high CO_2 concentration (5%), while a three-fold increase can be observed in the engineered strains in ambient CO_2 concentrations (0.04%).

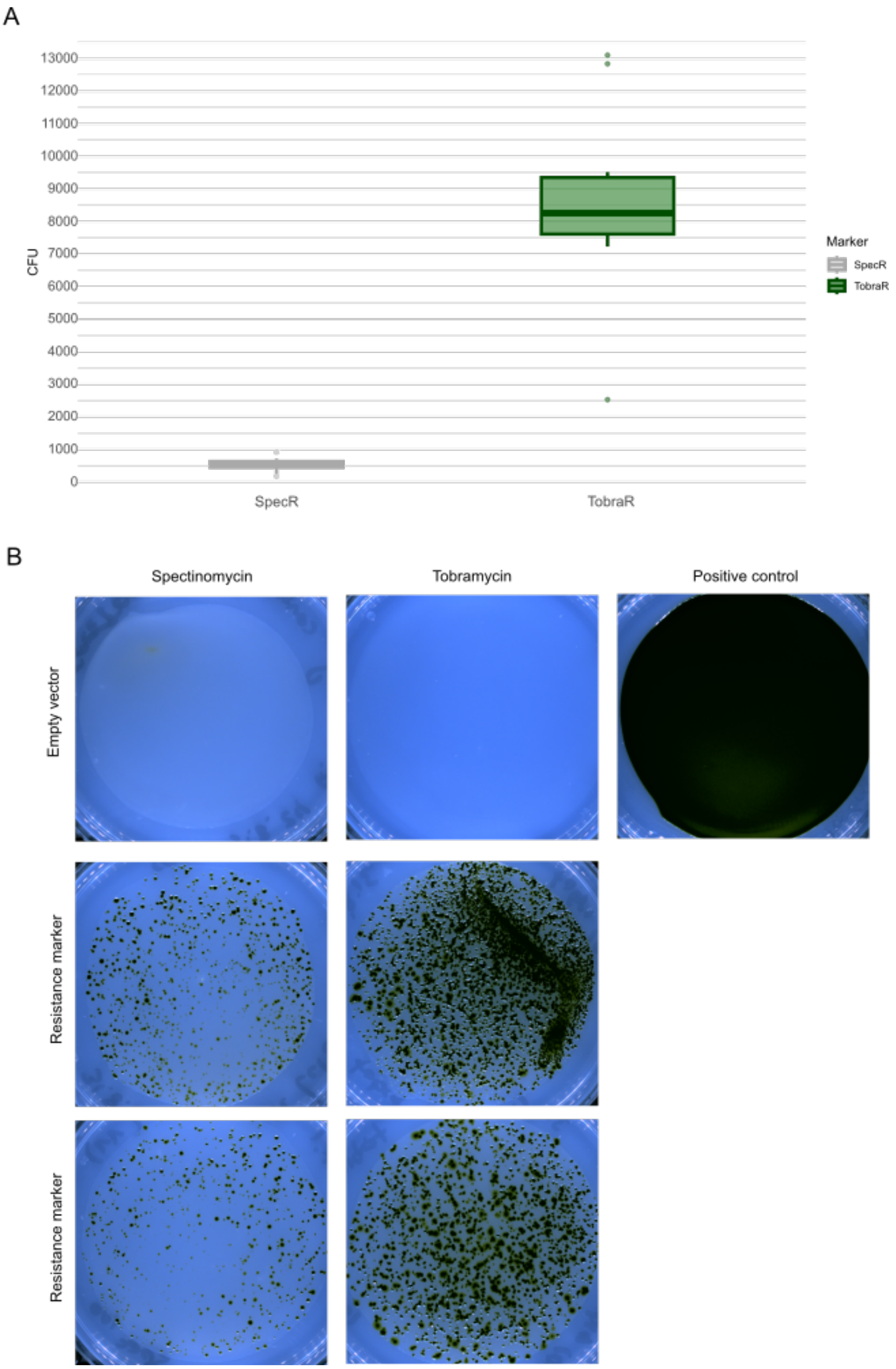

Supplementary figure 4: **Comparison of the selection efficiency for the Spectinomycin and Tobramycin antibiotic resistance markers. a,** Mean and standard deviations of Colony Forming Unit (CFU) count per plate after transformation of both markers in the WT strain, n=11. The Tobramycin marker leads to an order of magnitude increase in the number of colonies obtained after transformation. Box plot shows median as solid line, the box with lower and upper quartile, and the whiskers representing the data points that fall within 1.5 times the interquartile range (IQR) from the lower and upper quartiles. Any data point outside this range is considered as outlier. **b,** Example of a transformation plate for the selection markers. Two different selection marker systems are shown with two replicates and one negative controls for each one, as well as a positive control showing cell viability. Successful selection after chloroplast transformation can be observed,

notably with a high efficiency for tobramycin-based selection (100 µg/mL) over Spectinomycin-based selection (100 µg/mL).

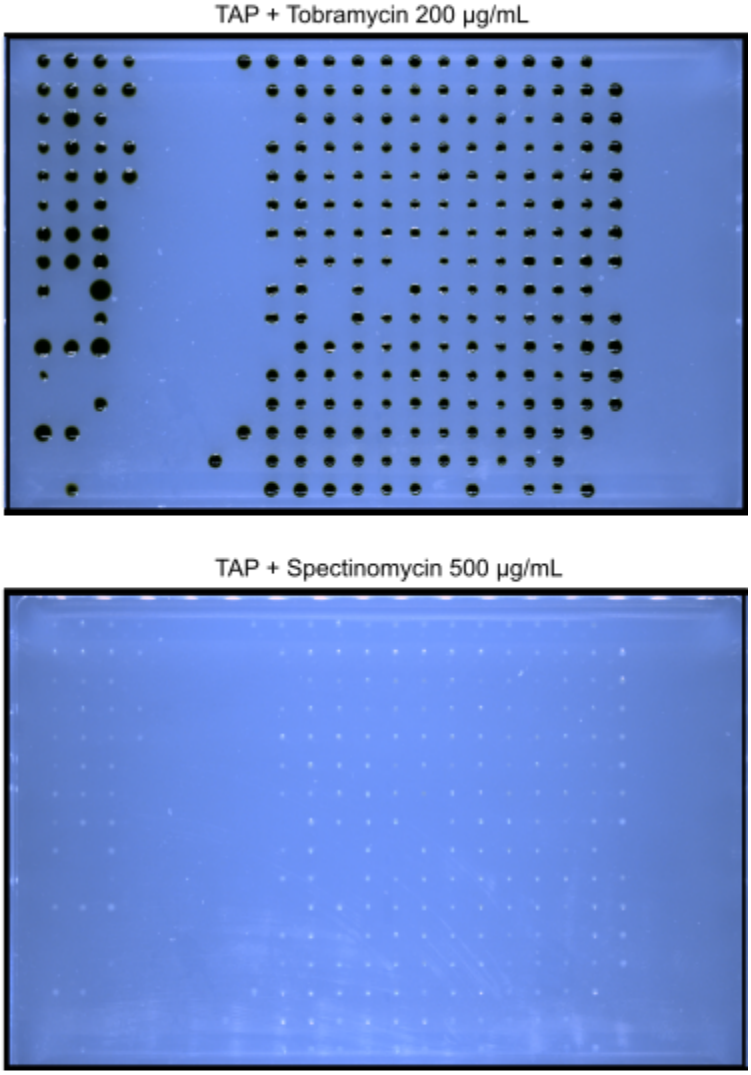

Supplementary figure 5: **Cross-resistance assessment between Tobramycin and Spectinomcin markers. a,** Tobramycin resistant transformants restreaked on two different media: TAP supplemented with Tobramycin (200 µg/mL, top plate) and TAP supplemented with Spectinomycin (500 µg/mL, bottom plate). Concentrations of antibiotic were chosen according to standard concentrations used for restreaks after confirmed integration and homoplasmy.

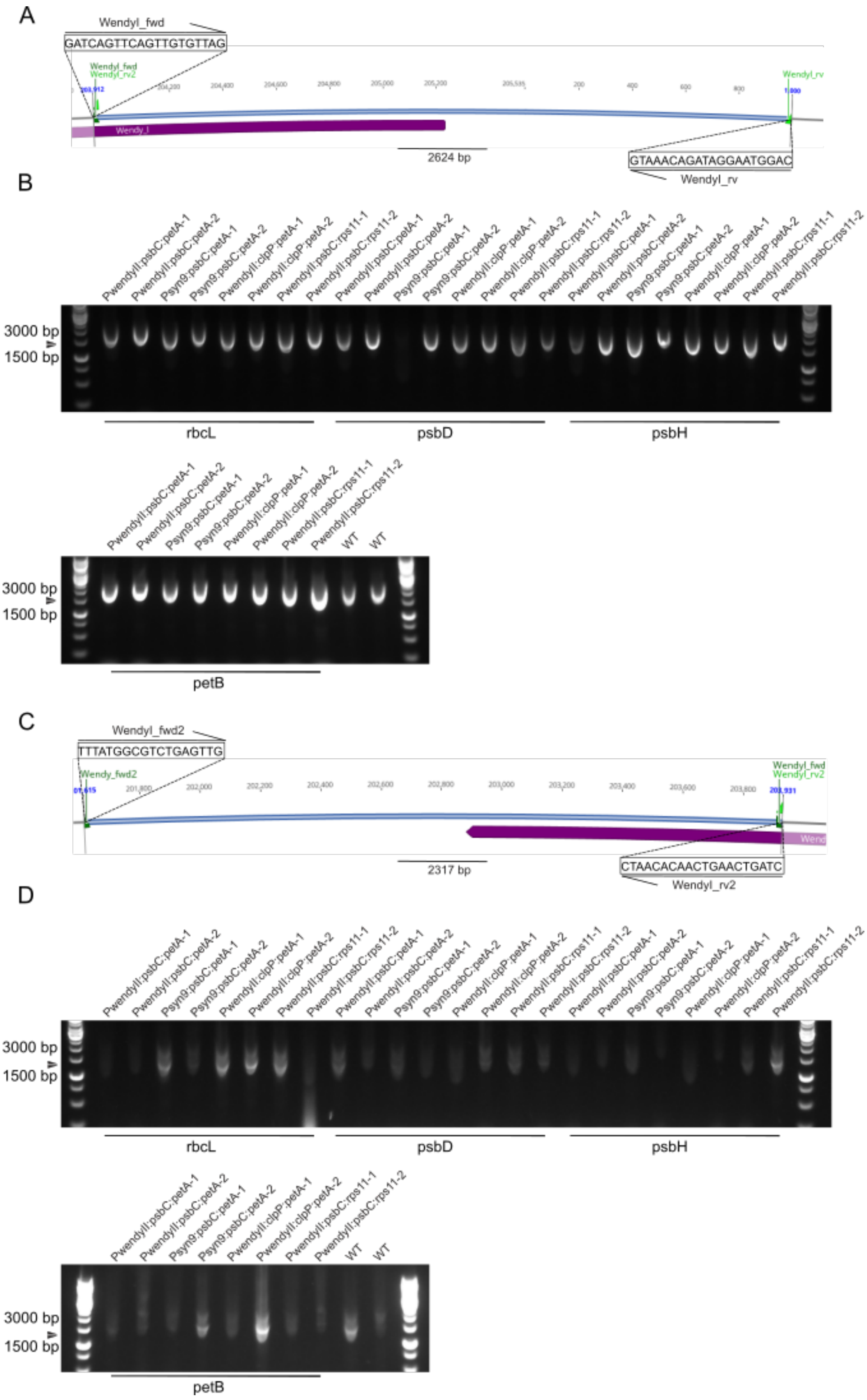

A
Wendyl_fwd
GATCAGTTCAGTTGTGTTAG
Wendyl_fwd
Wendyl_rv2
Wendyl_rv
Wendy_I
2624 bp
GTAAACAGATAGGAATGGAC
Wendyl_rv
B
Pwendyll:psbC:petA-1
Pwendyll:psbC:petA-2
Psyn9:psbC:petA-1
Psyn9:psbC:petA-2
Pwendyll:clpP:petA-1
Pwendyll:clpP:petA-2
Pwendyll:psbC:rps11-1
Pwendyll:psbC:rps11-2
3000 bp
1500 bp
rbcL
psbD
psbH
WT
petB
C
Wendyl_fwd2
TTTATGGCGTCTGAGTTG
Wendy_fwd2
Wendyl_fwd
Wendyl_rv2
2317 bp
CTAACACAACTGAACTGATC
Wendyl_rv2
D
Pwendyll:psbC:petA-1
Pwendyll:psbC:petA-2
Psyn9:psbC:petA-1
Psyn9:psbC:petA-2
Pwendyll:clpP:petA-1
Pwendyll:clpP:petA-2
Pwendyll:psbC:rps11-1
Pwendyll:psbC:rps11-2
3000 bp
1500 bp
rbcL
psbD
psbH
WT
petB

Supplementary figure 6: Gel analysis of cPCR amplification of the WendyI locus, in transplastomic strains characterized in Figure 5c, featuring two representative colonies for each construct, including four constructs per integration sites. Schematics for the first and second PCRs are shown in panels **a** and **c** respectively. **b and d,** PCR 1 and 2, respectively covering the right and left border of the WendyI gene. A distinct band, approximately 2100-2300 bp and 2500-2600 bp in size (for **b** and **d** respectively), is observed in all strains, including the WT, indicating that no recombination event took place for that locus. GeneRuler 1kb Plus (Thermo Fischer Scientific) was used as ladder for these gels.

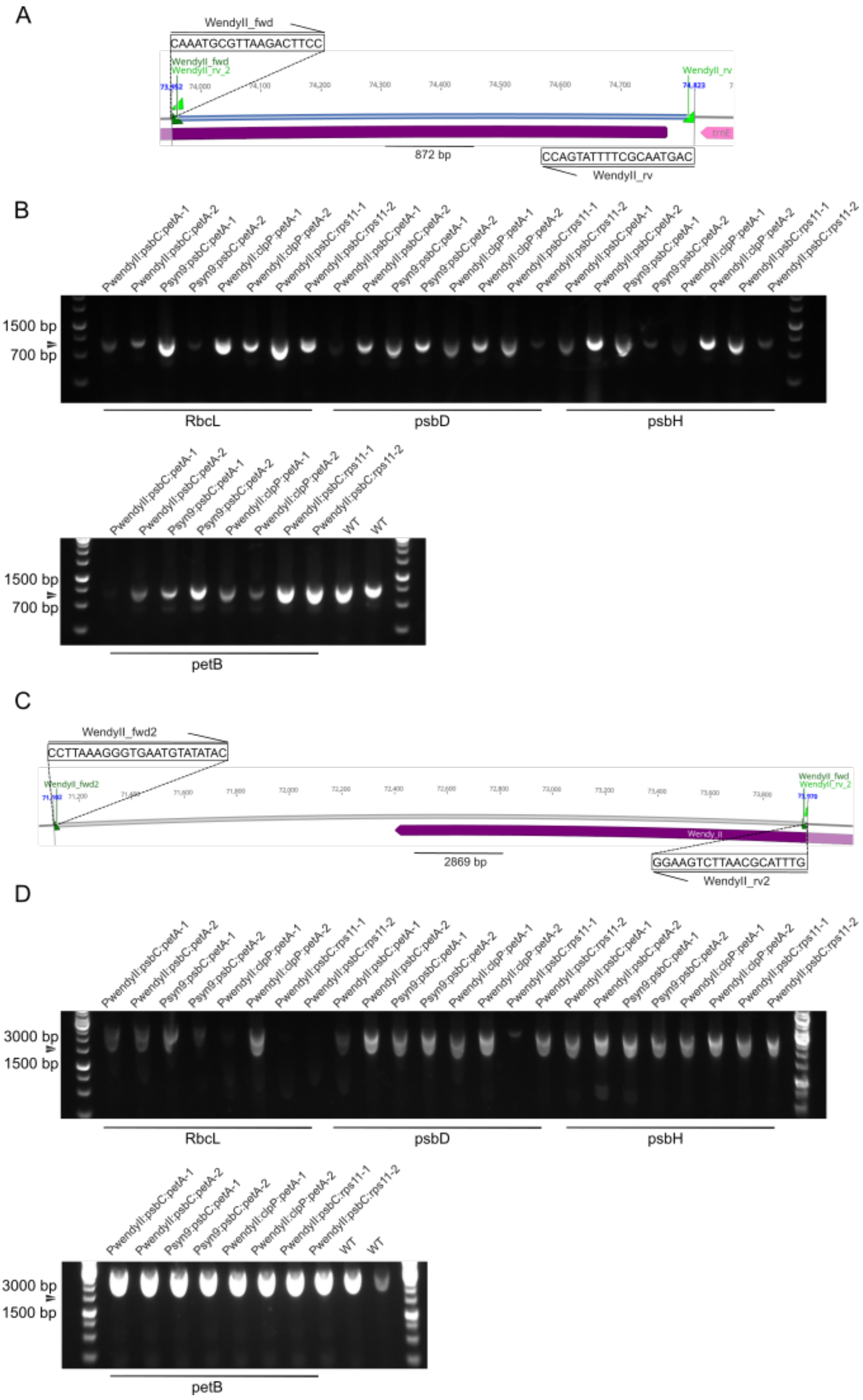
A
WendyII_fwd
CAAATGCGTTAAGACTTCC
WendyII_fwd
WendyII_rv_2
WendyII_rv
872 bp
CCAGTATTTTCGCAATGAC
WendyII_rv
B
Pwendyll:psbC:petA-1
Pwendyll:psbC:petA-2
Psyn9:psbC:petA-1
Psyn9:psbC:petA-2
Pwendyll:clpP:petA-1
Pwendyll:clpP:petA-2
Pwendyll:psbC:rps11-1
Pwendyll:psbC:rps11-2
Pwendyll:psbC:petA-1
Pwendyll:psbC:petA-2
Psyn9:psbC:petA-1
Psyn9:psbC:petA-2
Pwendyll:clpP:petA-1
Pwendyll:clpP:petA-2
Pwendyll:psbC:rps11-1
Pwendyll:psbC:rps11-2
Pwendyll:psbC:petA-1
Pwendyll:psbC:petA-2
Psyn9:psbC:petA-1
Psyn9:psbC:petA-2
Pwendyll:clpP:petA-1
Pwendyll:clpP:petA-2
Pwendyll:psbC:rps11-1
Pwendyll:psbC:rps11-2
1500 bp
700 bp
RbcL
psbD
psbH
Pwendyll:psbC:petA-1
Pwendyll:psbC:petA-2
Psyn9:psbC:petA-1
Psyn9:psbC:petA-2
Pwendyll:clpP:petA-1
Pwendyll:clpP:petA-2
Pwendyll:psbC:rps11-1
Pwendyll:psbC:rps11-2
WT
WT
1500 bp
700 bp
petB
C
WendyII_fwd2
CCTTAAAGGGTGAATGTATATAC
2869 bp
GGAAGTCTTAACGCATTTG
WendyII_rv2
D
Pwendyll:psbC:petA-1
Pwendyll:psbC:petA-2
Psyn9:psbC:petA-1
Psyn9:psbC:petA-2
Pwendyll:clpP:petA-1
Pwendyll:clpP:petA-2
Pwendyll:psbC:rps11-1
Pwendyll:psbC:rps11-2
Pwendyll:psbC:petA-1
Pwendyll:psbC:petA-2
Psyn9:psbC:petA-1
Psyn9:psbC:petA-2
Pwendyll:clpP:petA-1
Pwendyll:clpP:petA-2
Pwendyll:psbC:rps11-1
Pwendyll:psbC:rps11-2
Pwendyll:psbC:petA-1
Pwendyll:psbC:petA-2
Psyn9:psbC:petA-1
Psyn9:psbC:petA-2
Pwendyll:clpP:petA-1
Pwendyll:clpP:petA-2
Pwendyll:psbC:rps11-1
Pwendyll:psbC:rps11-2
3000 bp
1500 bp
RbcL
psbD
psbH
Pwendyll:psbC:petA-1
Pwendyll:psbC:petA-2
Psyn9:psbC:petA-1
Psyn9:psbC:petA-2
Pwendyll:clpP:petA-1
Pwendyll:clpP:petA-2
Pwendyll:psbC:rps11-1
Pwendyll:psbC:rps11-2
WT
WT
3000 bp
1500 bp
petB

Supplementary figure 7: Gel analysis of cPCR amplification of the WendyII locus, in transplastomic strains characterized in Figure 5c, featuring two representative colonies for each construct, including four constructs per integration sites. Schematics for the first and second PCRs are shown in panels **a** and **c** respectively. **b and d,** PCR 1 and 2, respectively covering the right and left border of the WendyII gene. A distinct band, approximately 700-800 bp and 2500-2700 bp in size (for **b** and **d** respectively), is observed in all strains, including the WT, indicating that no recombination event took place for that locus. GeneRuler 1kb Plus (Thermo Fischer Scientific) was used as ladder for these gels.

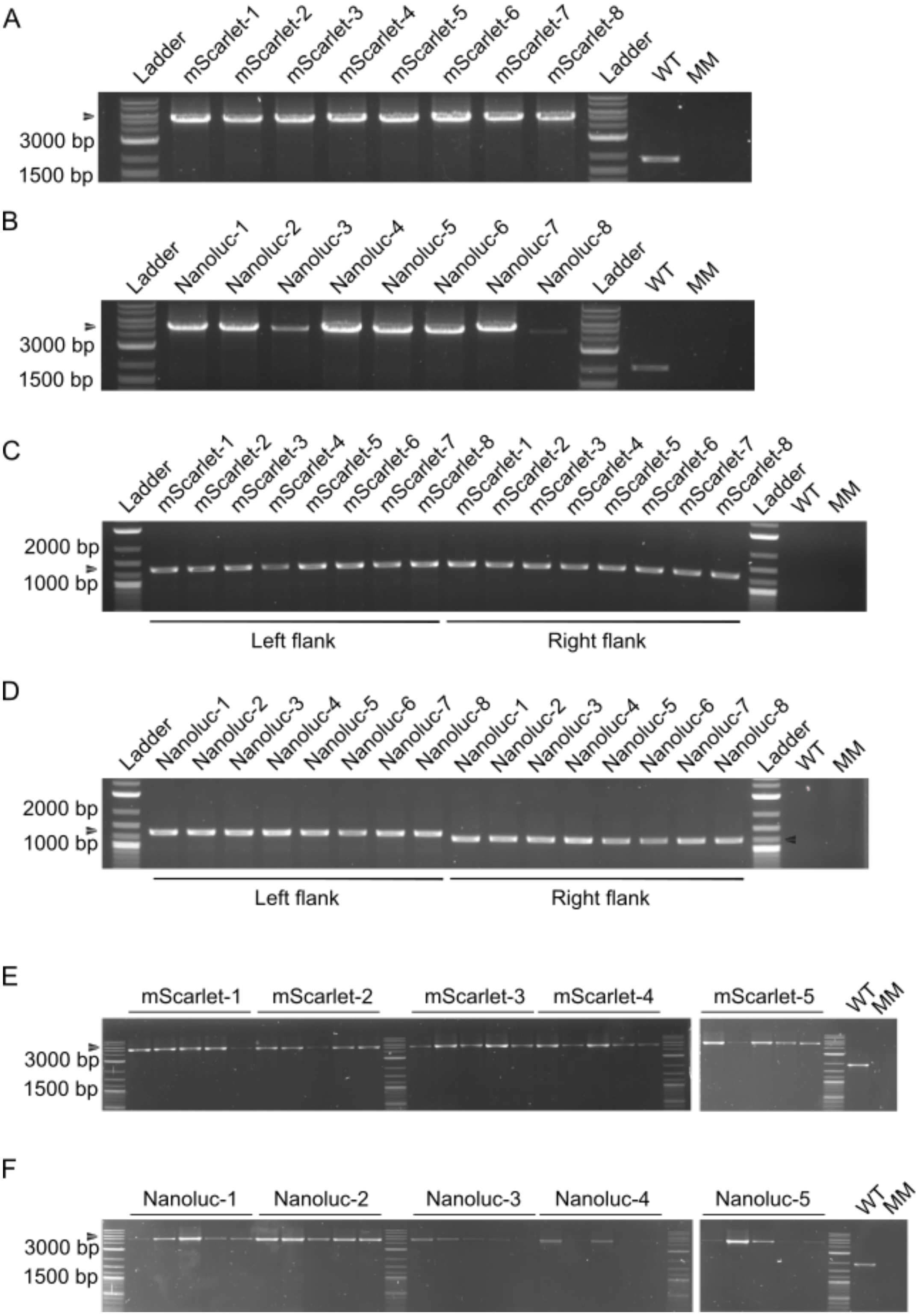

Supplementary figure 8: Gel analysis of Homoplasmy cPCR amplification of mScarlet-I and Nanoluc transplastomic strains. mScarlet strains analyzed in Figure2d-e and Nanoluc strains analyzed in Figures 2c and 3a-c were selected for confirming homoplasmy in colonies selected through the high-throughput pipeline. Panels **a-d** feature 8 clones per construct. The 1kb plus NEB ladder was used for these gels. **a-b**, cPCR covering the entire integrated fragment, homologies included, using the HomPCR_Confirm_01/ 02. Expected bands of 5400 bp can be observed for mScarlet strains in **a**, and of 5217 bp for Nanoluc strains in **b**. A smaller band around 1980 bp is observed for WT, characteristic of the amplification of the WT locus. No bands appear in the MasterMix control (MM). **c-d**, cPCR confirming left and right flank integration of the cassette, binding in each homology and reporter gene, using the HomPCR_Confirm_03/04 for the left flank, and HomPCR_Confirm_05/06 and 07/08 for the right flank of mScarlet-I and Nanoluc respectively. Expected bands of 1360 and 1318 bp are present in mScarlet strains left and right flanks in **a**. Similarly, expected bands of 1360 and 1192 bp are present in Nanoluc strains in **b**. **e-f**, After restreaking of colonies 1 to 5 for both mScarlet (mScarlet-1 to mScarlet-5) and Nanoluc (Nanoluc-1 to Nanoluc-5) transplastomic strains, 5 individual colonies were picked (each individual lane) and subjected to the homology cPCR shown in **a-b.** Each individual colonies for both mScarlet and Nanoluc clones displays the expected bands of 5400 and 5217 bp respectively, supporting homoplasmy for the population. The characteristic WT band of 1980 bp was observed, while no band appeared for the MM control.

A

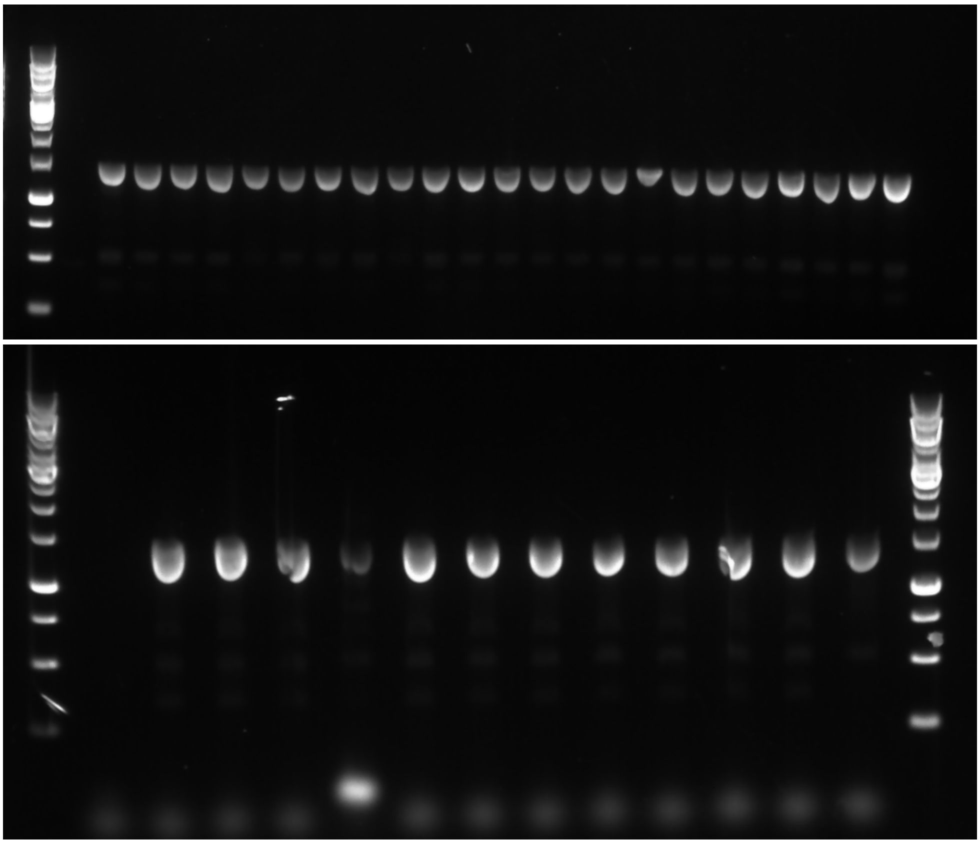

B

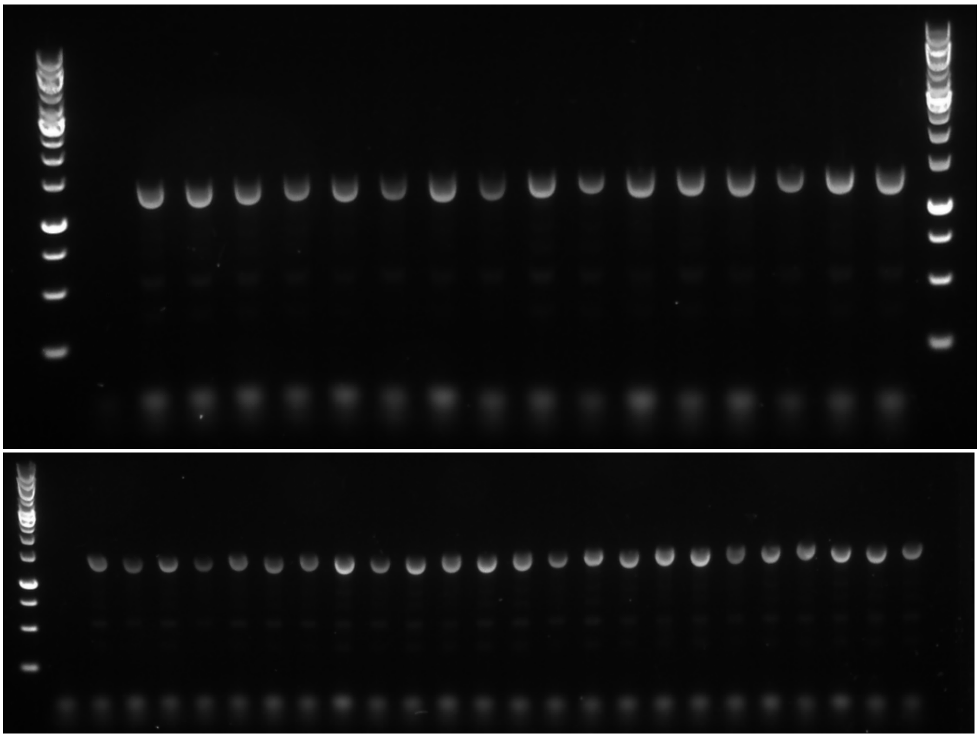

C

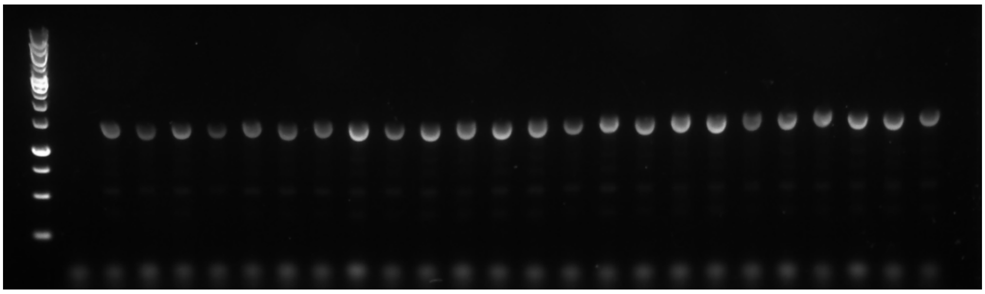

D

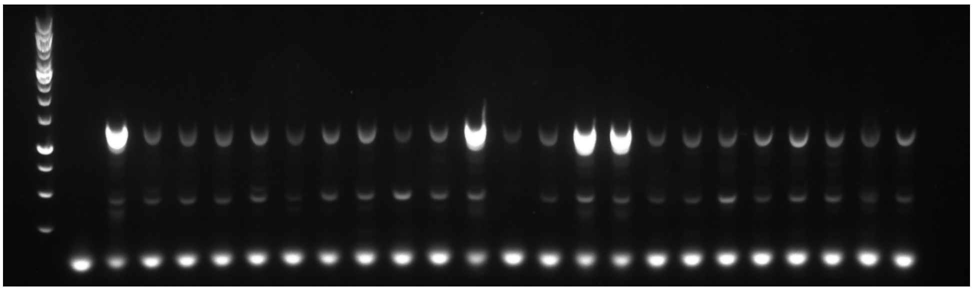

Supplementary figure 9: Unprocessed gels of Supplementary figure 1 depicted cPCR confirming Nanoluc integration in transplastomic strains.

A

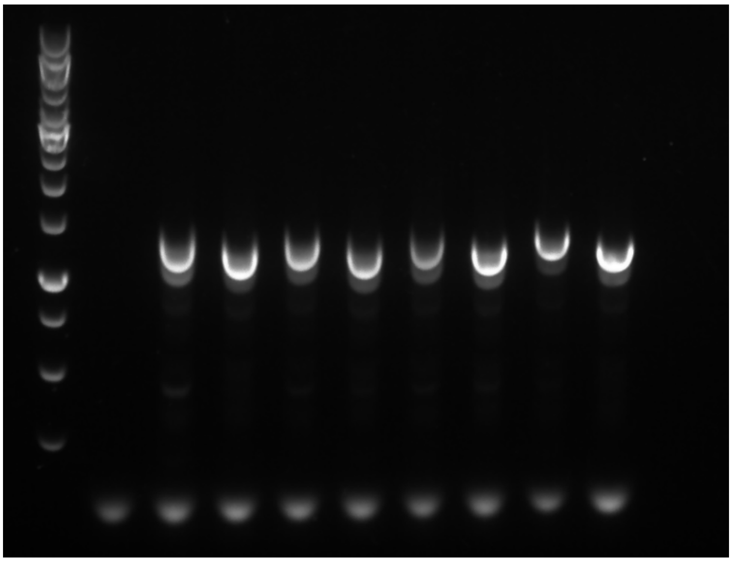

B

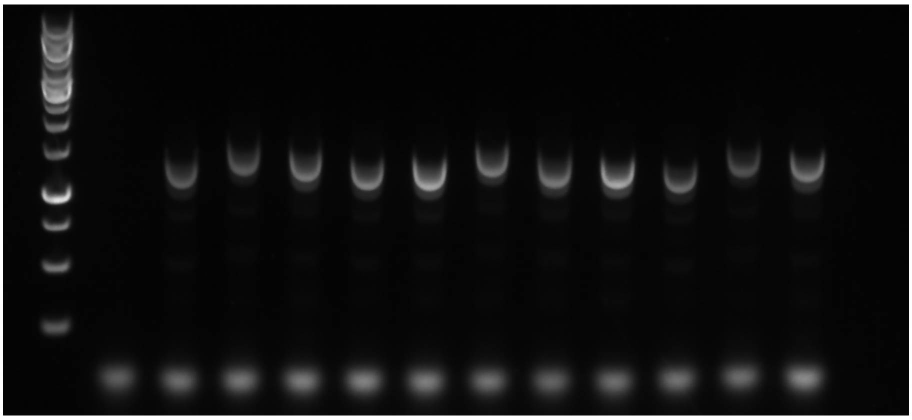

C

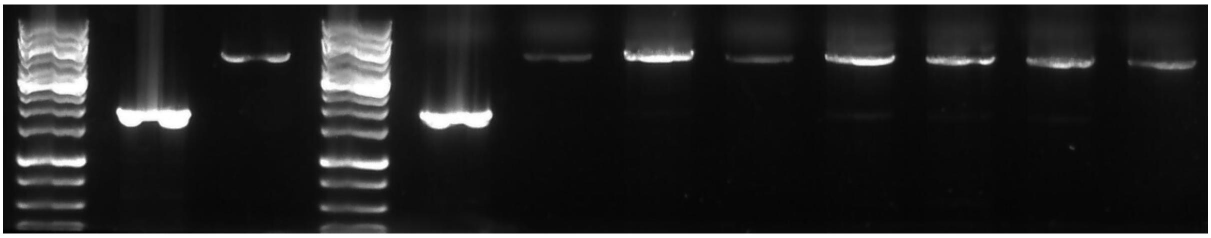

Supplementary figure 10: Unprocessed gels of Supplementary figure 2 depicted cPCR confirming Nanoluc integration in transplastomic strains, alongside cPCR confirming that the mutant strain cc-5797 contains a cassette in the Glycolate Dehydrogenase locus.

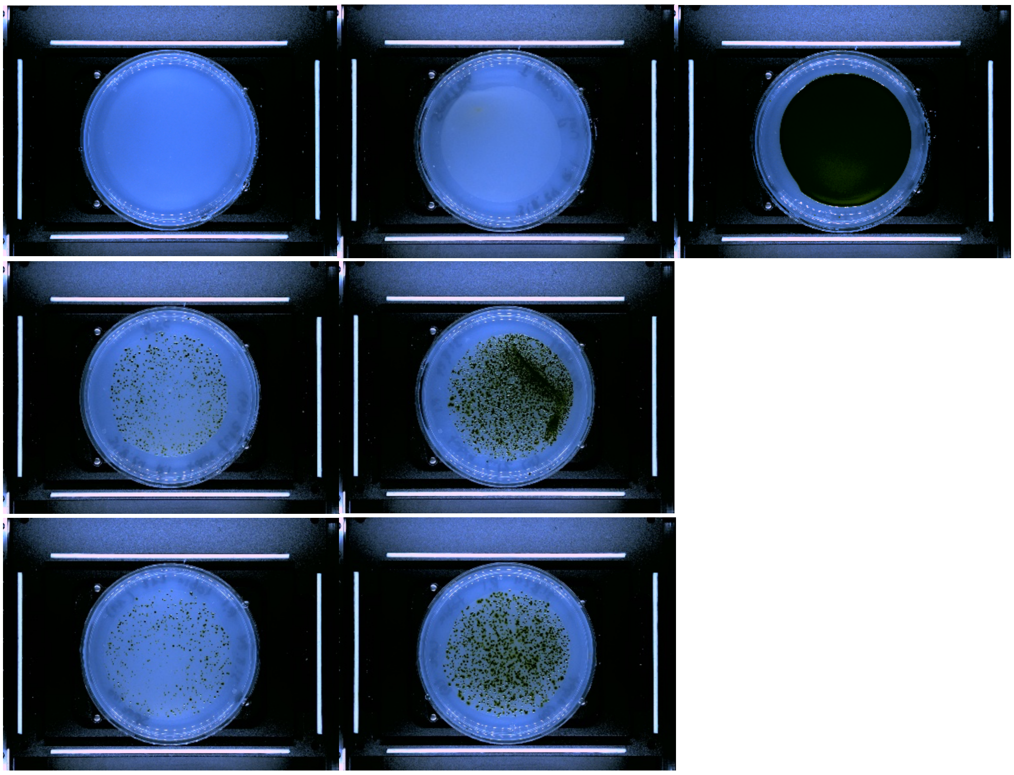

Supplementary figure 11: Unprocessed plate pictures of Tobramycin chloroplast transformation showed in Supplementary figure 4 panel B.

A

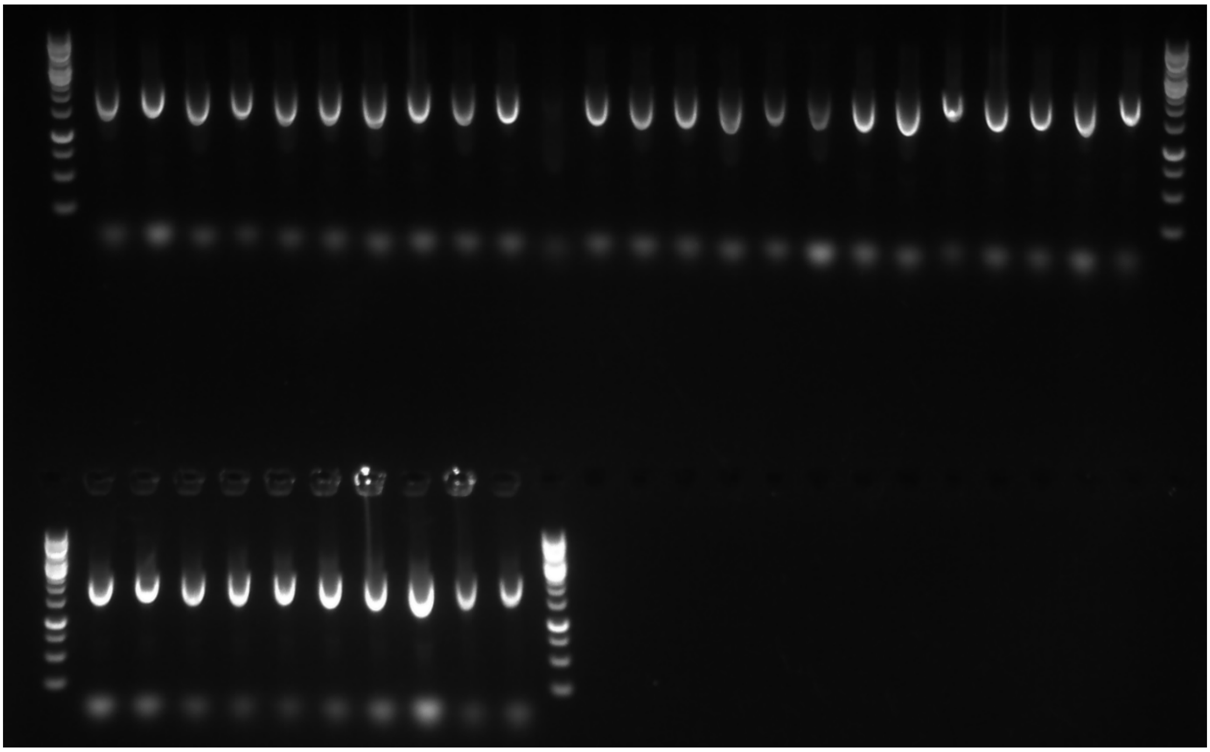

B

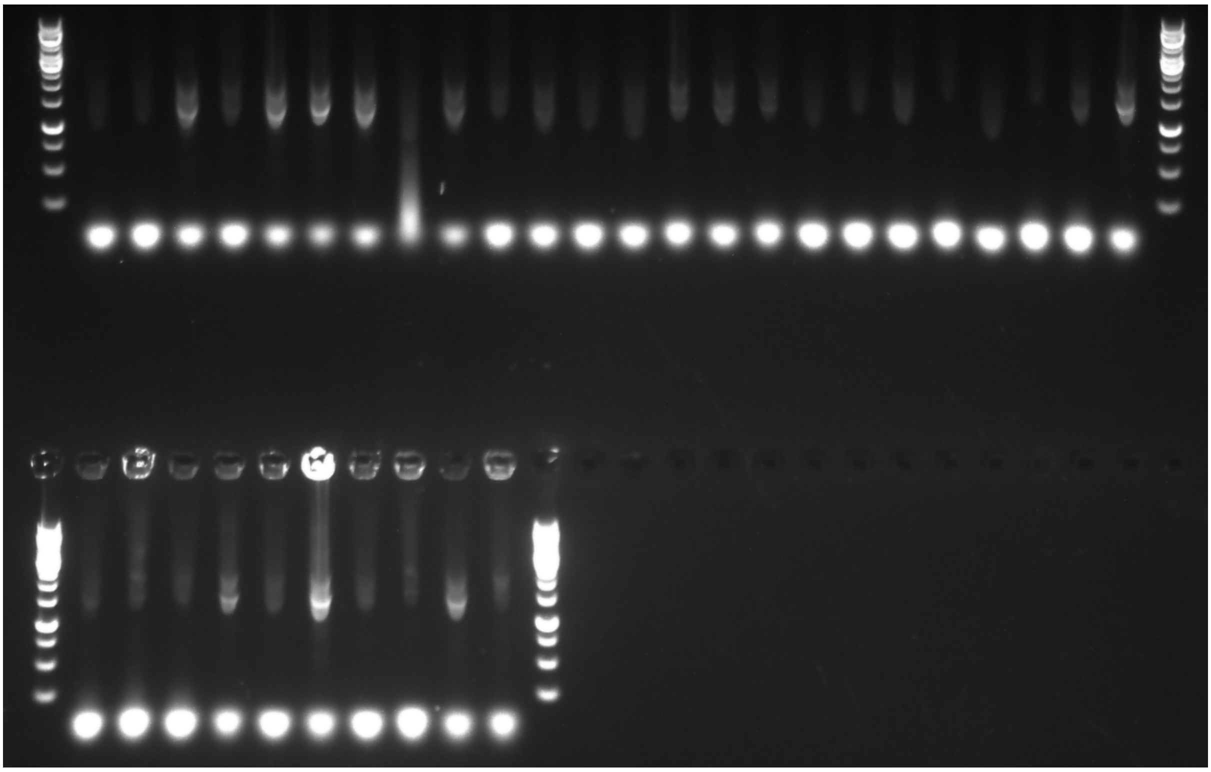

Supplementary figure 12: Unprocessed gels of Supplementary figure 6, panels b and d, depicted cPCR confirming Nanoluc integration in transplastomic strains, in WendyI locus right and left borders, to identify any homologous recombination event.

A

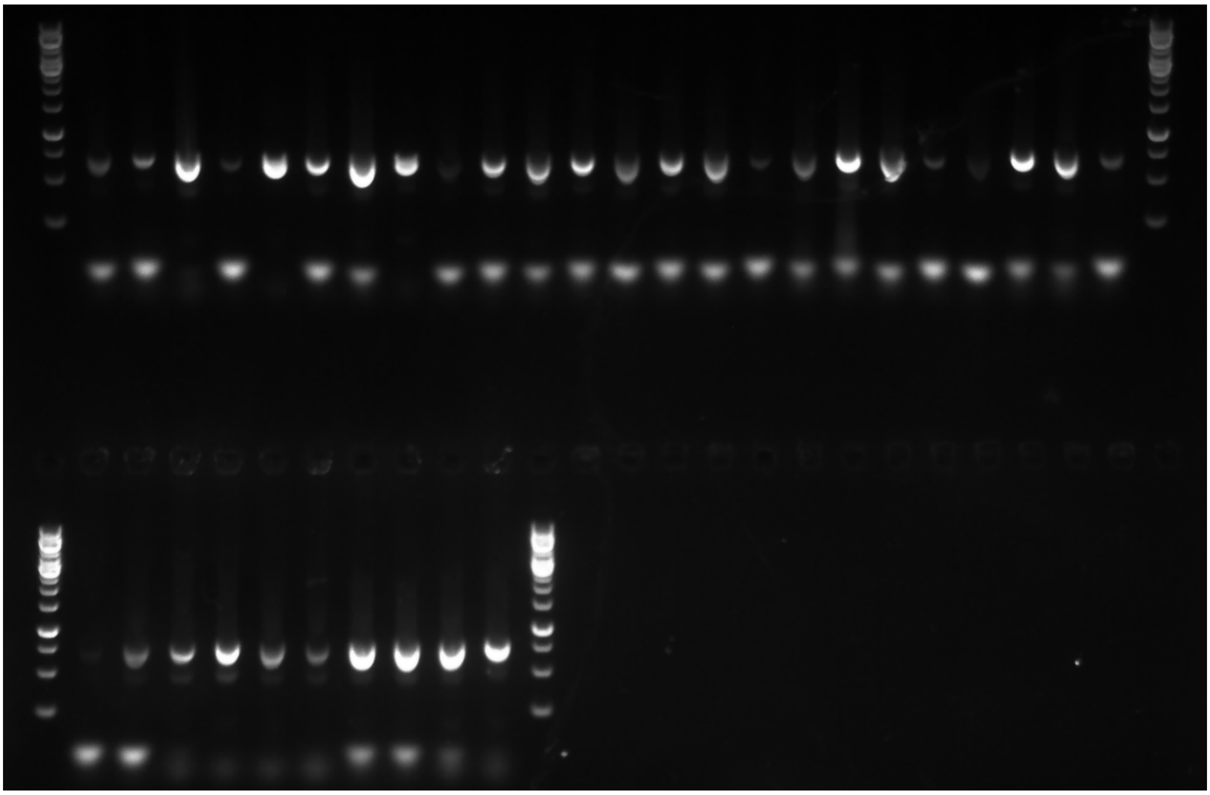

B

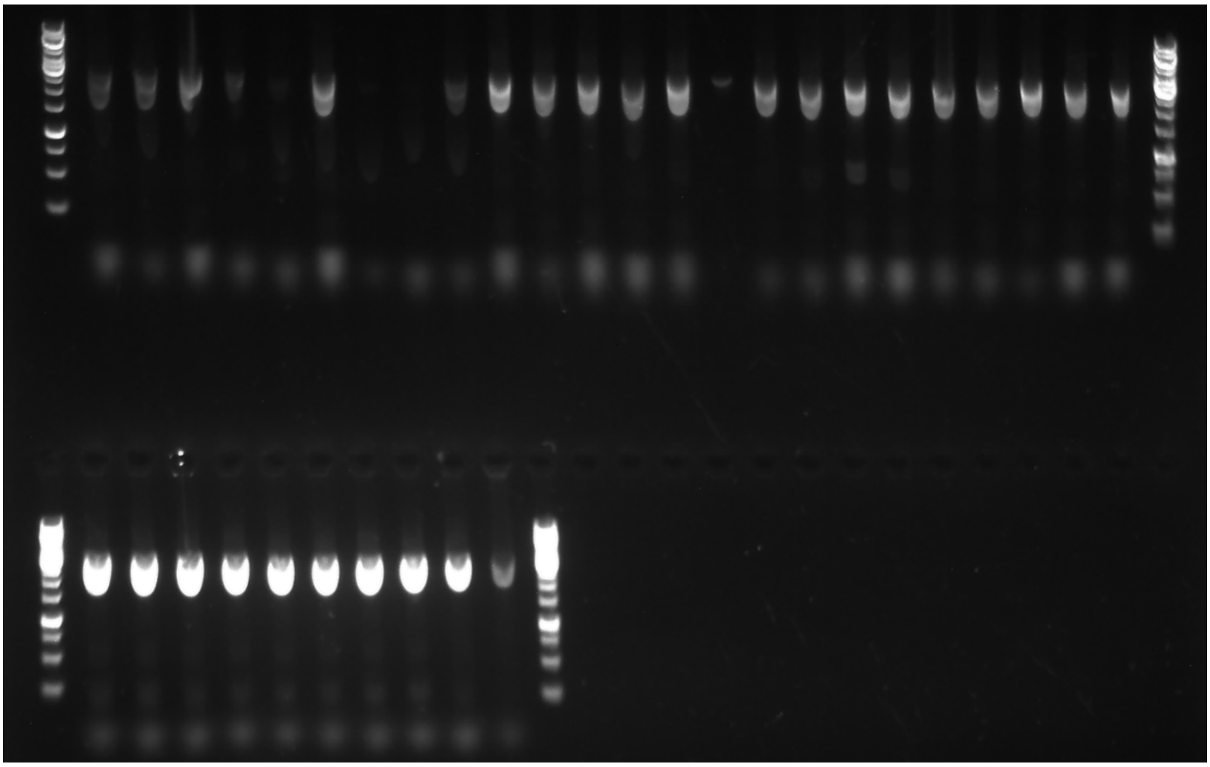

Supplementary figure 13: Unprocessed gels of Supplementary figure 7, panels b and d, depicted cPCR confirming Nanoluc integration in transplastomic strains, in WendyII locus right and left borders, to identify any homologous recombination event.

A

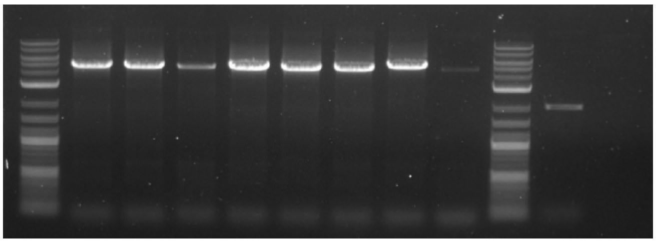

B

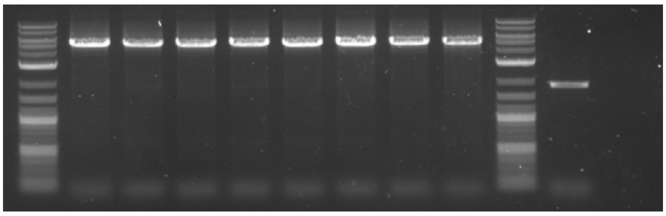

C

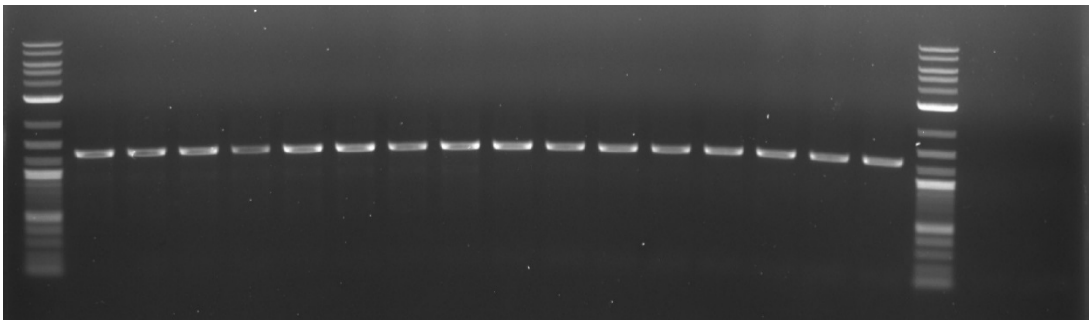

D

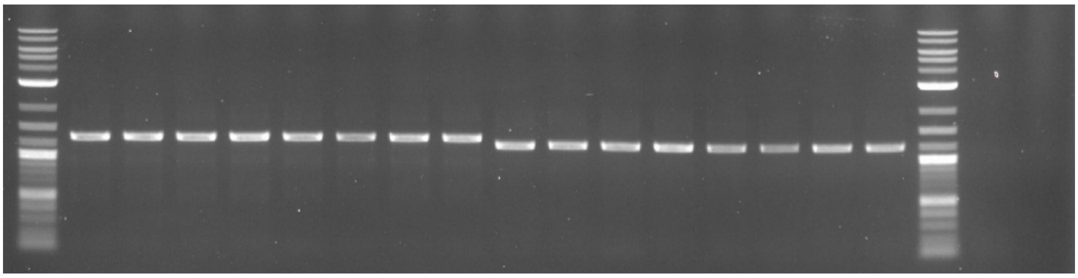

E

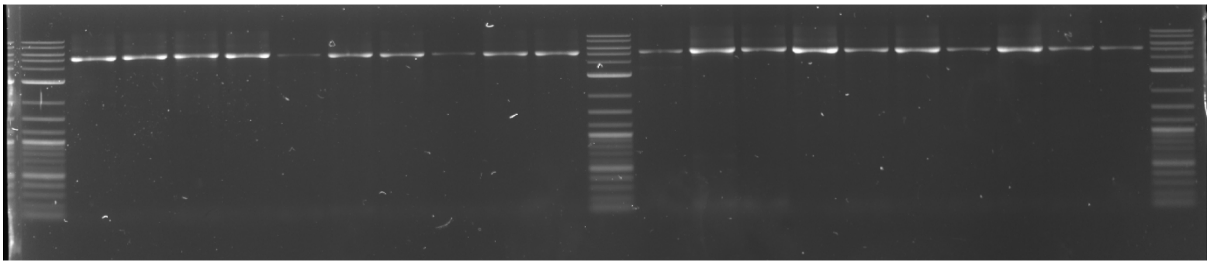

F

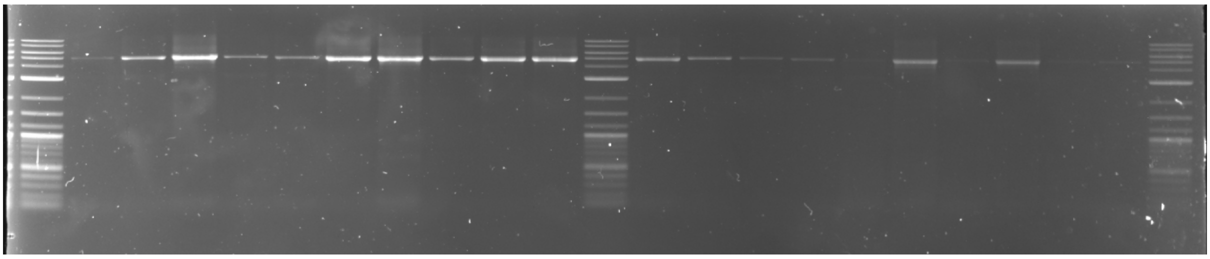

G

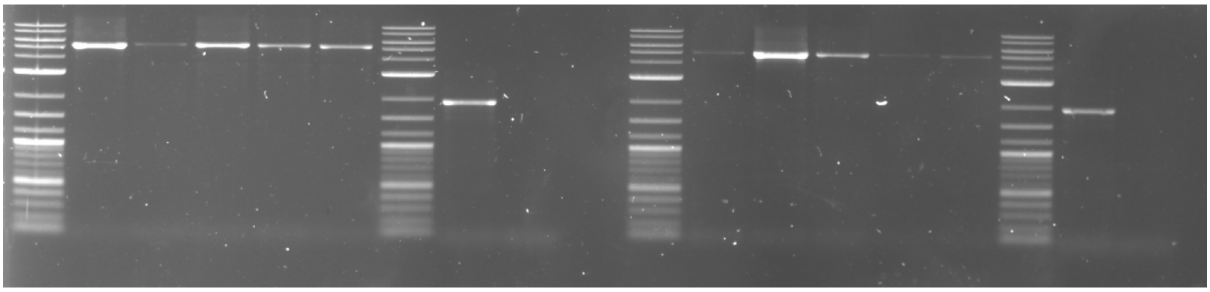

Supplementary figure 14: Unprocessed gels of Supplementary figure 8. a-b cPCR to assess homoplasmy in transplastomic strains, for Nanoluc and mScarlet constructs respectively. c-d, cPCR confirming left and right flank integration of the constructs in the chosen locus, for nanoluc and mScarlet constructs respectively. e-g, cPCR to assess homoplasmy in transplastomic strains after two rounds of restreaking to isolate single clones, for mScarlet and Nanoluc respectively constructs respectively.

Supplementary Text

Supplementary text 1: Fluorescence microscopy settings for each fluorophore, chlorophyll and brightfield.

<u>mCherry</u>

Excitation laser:	561 nm
Laser power:	2.5%
Pinhole:	103
Imaging mode:	SUPERRES
Master gain:	750
Digital gain:	1.5
Main beam splitter (MBS):	488/561/633
Second beam splitter (SBS):	LP 570
Emission dual filters:	BP 420-480 + BP 495-620

<u>mScarlet-I</u>

Excitation laser:	561 nm
Laser power:	2.5%
Pinhole:	109
Imaging mode:	SUPERRES
Master gain:	750
Digital gain:	1.5
Main beam splitter (MBS):	488/561/633
Second beam splitter (SBS):	LP 570
Emission dual filters:	BP 420-480 + BP 495-620

<u>mVenus</u>

Excitation laser:	514 nm
Laser power:	5%
Pinhole:	103
Imaging mode:	SUPERRES

Master gain:	775
Digital gain:	1.0
Main beam splitter (MBS):	458/514
Second beam splitter (SBS):	LP 525
Emission dual filters:	BP 420-480 + BP 495-550

<u>mCerulean</u>

Excitation laser:	458 nm
Laser power:	5%
Pinhole:	103
Imaging mode:	SUPERRES
Master gain:	775
Digital gain:	1.0
Main beam splitter (MBS):	458/561
Second beam splitter (SBS):	SP 615
Emission dual filters:	BP 465-505 + LP 525

<u>Chlorophyll</u>

Excitation laser:	633 nm
Laser power:	1%
Pinhole:	103
Imaging mode:	SUPERRES
Master gain:	720
Digital gain:	1.0
Main beam splitter (MBS):	488/561/633
Second beam splitter (SBS):	BP 570-625
Emission dual filters:	BP 570-620 + LP 645

<u>Brightfield</u>

Excitation laser:	561 nm
Laser power:	2%
Pinhole:	76.4
Imaging mode:	T-PMT
Master gain:	150

Digital gain:	1.0

Supplementary text 2: PIXL colony detection parameters. Imaging settings and algorithm selection.

Mode:	White
Gain:	39.4%
Gamma:	39.4%
Exposure:	10ms
Saturation:	34.4
White Balance - Red:	34.4%
White Balance - Green:	25%
White Balance - Blue:	74.3
Focus:	Auto
Lighting power:	70%
Algorithm:	Colony Separation
Organism:	S. cerevisiae (dark colonies)
Blueness Filter:	0 - 1
Circularity Filter:	0 - 1
Greenness Filter:	0 - 1
Intensity Filter:	0 - 1
Proximity Filter:	0 - 100
Radius Filter:	0.25 – 2.5
Redness Filter:	0 - 1

Supplementary text 3: FACS analysis settings.

Laser (mScarlet-I):	561 nm
Filter (mScarlet-I):	570-630 nm
Filter Gain (mScarlet-I):	40%
Laser (Chlorophyll):	638 nm
Filter (Chlorophyll):	690-750 nm
Filter Gain (Chlorophyll):	40%

Supplemental text 4: criteria for selecting integration sites

We evaluated four distinct chloroplast integration sites in this study. We began with the well-established *psbH* locus, widely used in *C. reinhardtii* chloroplast engineering[1].

To expand upon previously reported sites, we designed three additional loci (*psbD*, *rbcL*, and *petB*) using similar criteria:

1. We placed each insertion near a photosynthetic gene to allow for photosynthetic selection in knockout strains, similiar like what has been reported for the psbH integration site.
2. We aimed to minimize interference with neighboring genes by reviewing RNA-seq data to avoid genome regions of high or antisense transcription[2,3].
3. We searched for a specific orientation of neighboring genes. For instance, at the *rbcL* and *psbD* loci, flanking genes are oriented away from the insertion site (divergent orientation), thereby reducing the likelihood of antisense transcription. In the case of *petB*, the gene faces te integrated cassette, but low transcriptional activity at that locus, based on RNA-seq evidence, made it a worthwhile comparison for evaluating different genomic contexts.
4. We ensured compatibility with our modular cloning system by removing internal restriction sites without disrupting adjacent genes.

These criteria would also allow for integration at next to the *psbF* site in the chloroplast genome of C. reinhardtii, which we have not explored yet. Additionally, futures work could compare our sites to integration in the inverted repeats, which have been demonstrated to increase the gene expression strength due to the higher gene dosage.

Supplemental text 5: Design and in-depth analysis of synthetic chloroplast promoters

To develop a minimal chloroplast promoter scaffold in *C. reinhardtii*, we began with the commonly used *rrn16* promoter, which is 217 base pairs (bp) in length. By annotating the -35 and -10 sequence motifs, located near the midpoint of this 217 bp region, we hypothesized that the promoter could be shortened without significantly reducing its activity. Through iterative truncations, we arrived at a minimal scaffold of only 46 bp.
Building on this minimal promoter, we designed 22 synthetic variants to achieve a range of expression strengths and to explore fundamental design principles. The variants can be broadly grouped as follows:

Ten variants preserving the original -35 and -10 boxes and randomizing the other base pairs
Maintained -35 box (TTGACA) and -10 box (TAAATT), while randomizing all other positions.

Twelve variants with targeted changes relative to the *rrn16* promoter

Psyn11: Modified only upstream (5′) of the -10 box. Decrease in expression.
Psyn12: Modified only downstream (3′) of the -35 box. Relatively high expression.
Psyn13: Modified all bp between the -35 and -10 motifs. Low expression.
Psyn14: Modified the region immediately downstream of the -10 box. Data currently unavailable.
Psyn15: Modified only upstream (5′) of the -35 box. Very high expression.
Psyn16: Incorporated scattered base-pair changes across the entire scaffold.
Psyn17: Replaced 17 bp between the -35 and -10 boxes with sequence from the *psaA* promoter. Very high expression.
Psyn18: Replaced 23 bp between the -35 and -10 boxes with *psaA* sequence. Low expression.
Psyn19: Added the RUA-box 5′ of the -35 box (derived from the tobacco *rrn16* promoter). High expression, but not exceeding other strong promoters.
Psyn20: Substituted the native -10 box (TAAATT) with the canonical bacterial consensus (TATAAT). One of the highest expression levels observed.
Psyn21: Changed every base outside the -35 and -10 motifs to its complementary nucleotide (A→T, T→A, G→C, C→G). Very low expression.
Psyn22: Modified every base 5′ of the -35 box and 3′ of the -10 box. Very high expression.

For benchmarking, we also included two endogenous reference promoters (*Pwendy II* and *PtrnE*). Both share the same -35 box, the same distance between the -35 and -10 motifs, and an even more bacterial-like -10 box, leading to robust expression in *C. reinhardtii*.
These experiments revealed several important trends:

Proximal region to the -10 box: Positions immediately upstream (5′) of the -10 motif play a key role in maintaining high expression. In particular, a conserved G two nucleotides 5′ of the -10 box was shared by many strong promoters.
Sequence upstream of -35 and downstream of -10: Modifications in these regions did not generally affect expression (e.g., the region 5′ of the -35 box, which is crucial in tobacco, appeared nonessential in *C. reinhardtii*).
Spacing between -35 and -10 motifs: Psyn17 and Psyn18 showed that changing this distance strongly impacts expression.

Substituting the native -10 motif: Replacing TAAATT with the bacterial consensus TATAAT (Psyn20) yielded especially high expression.

Collectively, these findings highlight the utility of synthetic promoter libraries in uncovering critical regulatory elements and informing more advanced synthetic biology efforts in chloroplast engineering.

Supplemental text 6: Manual for the Chloroplast Modular Cloning System

Type IIs restriction enzymes

Unlike conventional Type II restriction enzymes, which cleave DNA within their recognition sequences, Type IIS restriction enzymes cut outside their recognition sequences at defined distances (Figure 1) This property allows for the flexible design of overhangs, enabling a single enzyme to generate up to 256 distinct 4-base pair overhangs. This capability has led to the development of the Golden Gate cloning technique, now one of the most widely used cloning methods. Commonly used Type IIS enzymes include BsaI, BsmBI, BpiI (BbsI), PaqCI (AarI), and SapI.

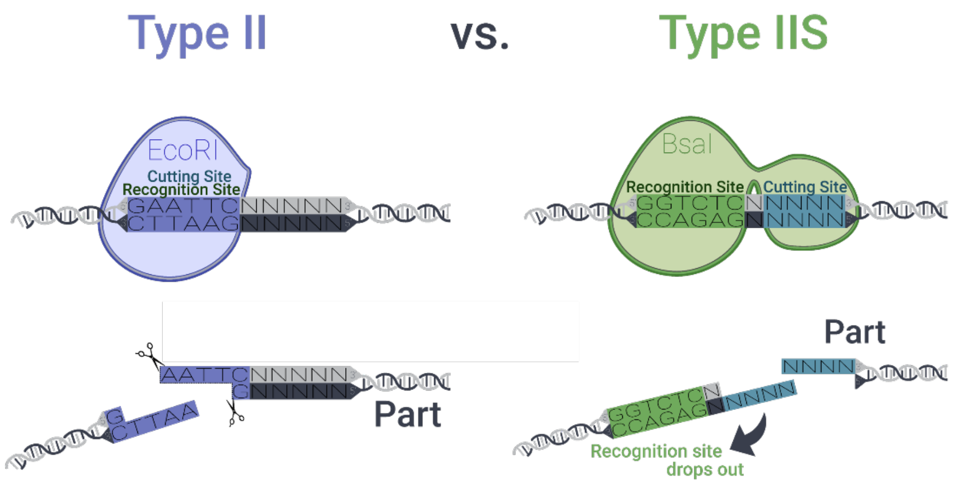

Figure 1 General principle of Type IIs restriction enzymes
While Type II restriction enzymes cleave DNA within their recognition sequence, Type IIS restriction enzymes cut DNA adjacent to their recognition sites, allowing the 4-base pair cut site to be freely chosen.

Golden Gate cloning allows these enzymes to be combined in a single "one-pot" reaction with T4 ligase, eliminating the need for separate restriction and ligation steps. Because the recognition sites for Type IIS enzymes are cleaved off during digestion and are not included in the final assembly (Figure 1), correctly assembled constructs are not cleaved further. This feature enables the cycling of restriction enzyme digestion and ligation steps, which increases the efficiency of obtaining correctly assembled constructs.

General introduction

Following the initial development of Golden Gate cloning and the subsequent creation of the widely adopted Modular Cloning (MoClo) standard syntax, which employs standardized overhangs generated by Type IIS restriction enzymes such as BsaI, BpiI, and BsmBI, a consensus has emerged within the plant synthetic biology community.
The widely adopted Phytobrick standard in the plant synthetic biology community has been successfully adapted for a range of photosynthetic organisms, including cyanobacteria, plants, mosses, plant chloroplasts, and even for nuclear engineering in Chlamydomonas.
To apply synthetic biology techniques to chloroplast engineering in Chlamydomonas reinhardtii, standardizing and characterizing genetic parts is essential. Although different cloning systems employ varying strategies for assembling multigene constructs, most are

compatible with the assembly of single transcription units from standardized parts. Our system, utilizes a hierarchical assembly approach, similar to the common Plant MoClo and the Chlamydomonas MoClo system, rather than the iterative cloning scheme used in the GoldenBraid/Loop assembly standard.
The following sections provide a detailed explanation of our cloning system.

The general Modular cloning scheme
In our hierarchical cloning scheme, individual Level 0 parts are first cloned into a universal acceptor vector. A Level 0 part represents the DNA sequence of a fundamental genetic element—such as a promoter, 5'UTR, coding sequence, or 3'UTR—flanked by a standardized 4-bp overhang and BsaI recognition sites.

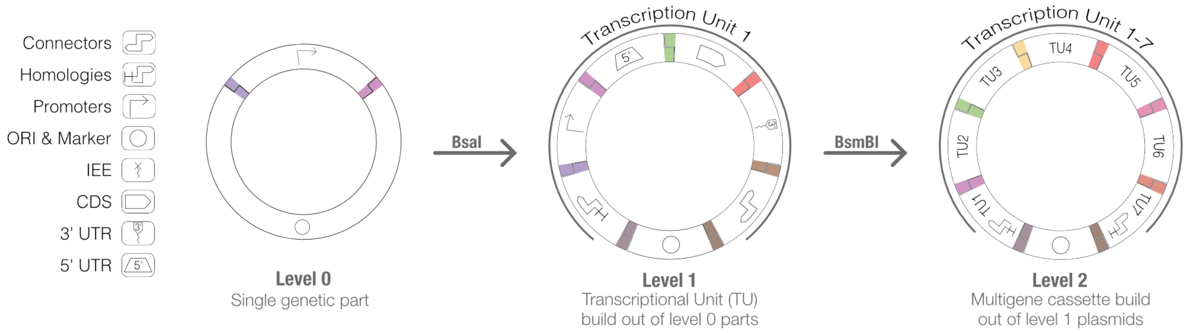

Figure 2 The hierarchical modular cloning scheme
New parts are first domesticated and assembled into a universal acceptor vector (Level 0). These parts can then be combined in a one-pot reaction to create Level 1 plasmid containing a single transcriptional unit. Optionally, these single transcriptional units can be further assembled into a multigene cassette. (Level 2).

These parts are then assembled into a single transcriptional unit using a one-pot Golden Gate reaction with BsaI, combining 5–9 Level 0 parts into a Level 1 assembly.
Next, 2–7 transcriptional units are integrated into a Level 2 multigene cassette via another one-pot Golden Gate reaction using BsmBI. In our system, the arrangement and order of transcription units are defined by the chosen connectors (detailed explanation below), rather than by the acceptor vectors used in the Level 1 assembly.
To enable selection of successfully assembled plasmids, different antibiotic markers are used at each assembly stage. In our system, Level 0 employs chloramphenicol, Level 1 uses carbenicillin (or ampicillin), and Level 2 utilizes kanamycin selection.

Domesticating new Level 0 parts
New Level 0 parts are generated using PCR products or synthesized DNA. These linear sequences are then assembled into a single standardized universal acceptor vector. In contrast to other methods that require multiple acceptor vectors, only one is needed because standardized overhangs are incorporated into the PCR primers or synthesized sequence. The specific overhangs are detailed in Table 1.

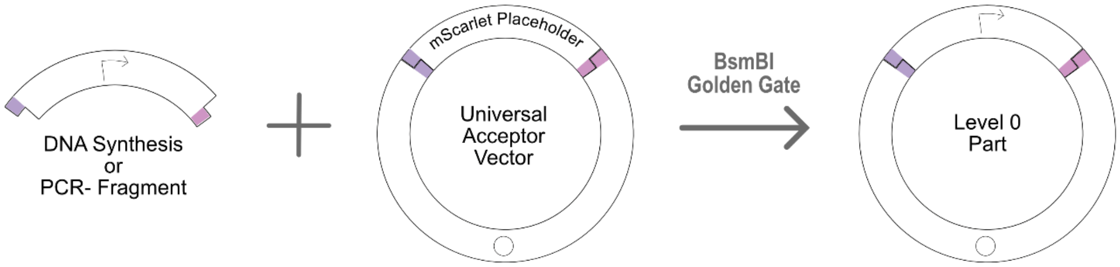

Figure 3 Domesticating new Level 0 Parts
PCR or DNA synthesis fragments that include standardized overhangs and BsmBI recognition sites are assembled into the Universal Acceptor vector. This vector features an mScarlet placeholder, enabling pink/white colony screening.

The unique overhangs assign the position within the Level 1 assembly for the subsequent cloning steps. For some overhangs, additional nucleotides are appended. For example, overhangs in the 3'UTR position (GCTTAA, GGTAA) incorporate stop codons, while those at the tag positions (CCATG, GGAATG, GCTTTA) include extra bases to ensure that fusion tags remain in frame.
For the domestication of new coding sequences, the original start and stop codons are removed. The start codon is reintroduced as part of the Golden Gate overhang for the CDS, which prevents double start codons, while the stop codon is incorporated into the 3'UTR/terminator sequence. This design allows for the modular addition of C-terminal tags.
For most Level 0 assemblies, the BsmBI enzyme is used. However, for new Connector/Integration site homology parts, the enzyme PaqCI is required along with the alternative Universal acceptor vector. This is because these parts contain BsmBI recognition sites, which are necessary for the subsequent assembly of multi-gene constructs.

Table 1 Overhangs for creating new Level 0 parts via DNA synthesis/PCR
Level 1 overhangs are indicated in blue. BsmBI sites (**CGTCTC/GAGACG)** are indicated. For connector overhangs PaqCI sites are used.

Position	Sequence
1_5'Connector_fwd	AACACCTGCATATCTCGAACACGTCTCGNNNN
1_5'Connector_rv	GGAGTGAGATATGCAGGTGTT
2_Promoter_fwd	AACGTCTCGCTCGGGAG
2_Promoter_rv	TACTTGAGGGAGACGAA
3_5'UTR_fwd	AACGTCTCGCTCGTACT
3_5'UTR_rv	AATGTGAGGGAGACGAA
3a_5'UTR_fwd	AACGTCTCGCTCGTACT
3a_5'UTR_rv	CCATTGAGGGAGACGAA
3b_n-tag_fwd	AACGTCTCGCTCGCCATG
3b_n-tag_rv	GGAATGTGAGGGAGACGAA
4_CDS_fwd	AACGTCTCGCTCGAATG
4_CDS_rv	GCTTTGAGGGAGACGAA
5_3'UTR+Terminator_fwd	AACGTCTCGCTCGGCTTAA
5_3'UTR+Terminator_rv	CGCTTGAGGGAGACGAA
5a_c-tag_fwd	AACGTCTCGCTCGGCTTTA

5a_c-tag_rv	GGTATGAGGGAGACGAA
5b_3'UTR+Terminator_fwd	AACGTCTCGCTCGGGTAA
5b_3'UTR+Terminator_rv	CGCTTGAGGGAGACGAA
6_3'Connector_fwd	AACACCTGCATATCTCGCGCT
6_3'Connector_rv	NNNNGGAGACGAGACTGAGATATGCAGGTGTT
7-8_E.coli_ORI+Resistance_fwd	AACGTCTCGCTCGAGAC
7-8_E.coli_ORI+Resistance_rv	AACATGAGGGAGACGAA

Our version of the universally acceptor vector, allows for pink/white selection after cloning and transformation, due to the mScarlet placeholder cassette in the vector. Except of this mScarlet placeholder cassette this vector is sequence identical to the commonly used (in the plant synthetic biology community) Universal acceptor vector (Addgene #68161). Another aspect, which needs to be considered when creating new Level 0 Part is that internal enzyme recognition sites for at least BsaI and BsmBI have to be removed within the part sequence to not interfere with the subsequent cloning process.

Assembly of Level 1 Transcription Units

To construct a single transcriptional unit (Level 1), 5–9 Level 0 parts are assembled in a one-pot reaction using BsaI. The overhangs on these parts adhere to the widely adopted Phytobrick standard, which is also employed in the modular cloning system for nuclear engineering in Chlamydomonas, including the A1–C1 overhangs (Figure 4).

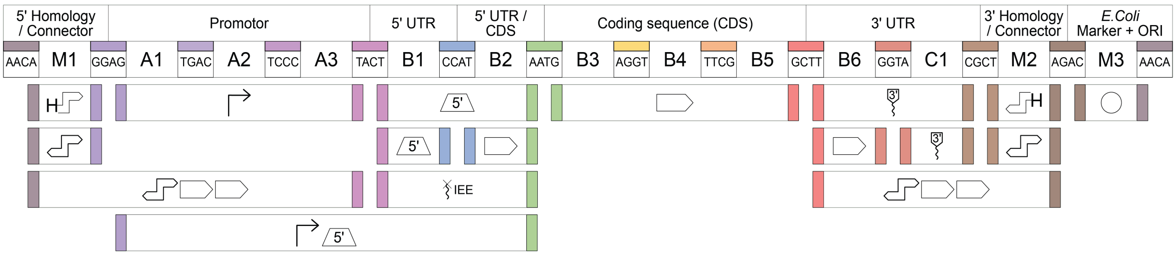

Figure 4 Part types and positioning of the chloroplast modular cloning system
The modular design of the system provides high flexibility by offering 5'UTRs, promoters, IEEs, and tags as separate, independently selectable parts. The chloroplast modular cloning systems adheres to the widely adopted Phytobrick standard, including the A1-C1 overhangs. These overhangs are complemented by two additional overhangs for the connectors/homology parts, as well as the E. coli marker and origin.

Compared to the modular cloning system for nuclear engineering in Chlamydomonas, our system brings several new features, such as the additional connector/homology parts, the possibility of building polycistronic transcription units via IEEs and a few more.

Our system differs from the traditional modular cloning architecture by not using the conventional Level 1 and Level 2 acceptor vectors to dictate the placement of the transcription units in subsequent assembly steps. Instead, this function is performed by connector parts that contain BsmBI sites and specific overhangs to define the arrangement of multigene constructs. The following sections provide a detailed explanation of these homology parts/connectors and other new features.

New in our Chloroplast Modular cloning System:

Chloroplast genome homology parts and connectors

To adapt the Modular Cloning (MoClo) approach for chloroplast engineering, the standard MoClo positions have been extended with additional Connector/Homology positions. These

extensions enable site-directed integration into the plastome and support multigene assembly. Four specific regions of the chloroplast genome are available as homology sites for precise, site-specific integration. For multigene assemblies, the 5' homology region is incorporated into the first transcription unit and the 3' homology region into the last transcription unit. Alternatively, these homology parts can be used within a single transcription unit to integrate a single gene of interest.

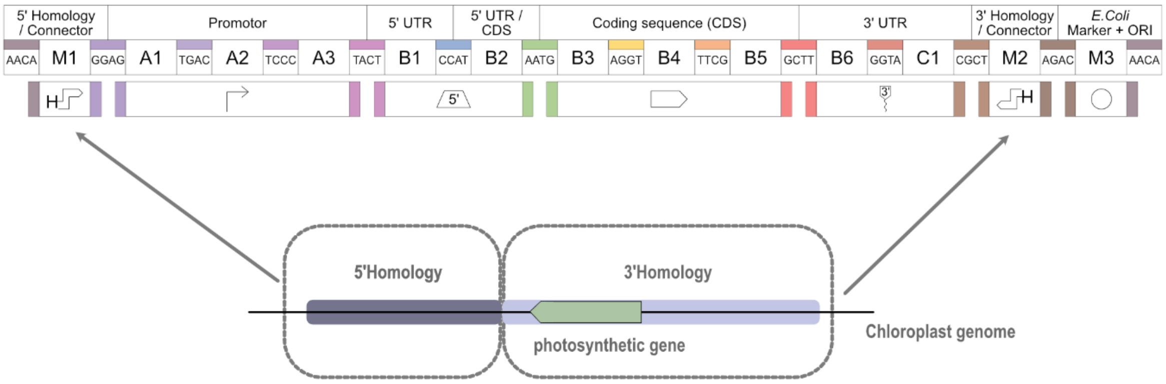

Figure 5 Homology parts of the chloroplast genome of *Chlamydomonas reinhardtii*
Chloroplast regions for four distinct integration sites are provided as homology parts within the extended Modular Cloning framework, supplementing the standard Phytobrick part positions. Notably, one homology part also includes a photosynthetic gene, which can be leveraged for photosynthetic selection strategies.

The same modular cloning position as the homology parts (M1 and M2) are also used for the connector parts. These connector parts are used within a Level 1 assembly to define the order of transcription units within a multigene Level 2 assembly. These parts include BsmBI sites and the different overhangs for the respective position of the transcription unit.
Our system features several types of connector parts—including short, long, and reverse connectors (detailed below). Each type comprises seven distinct connectors corresponding to the seven positions in a Level 2 multigene assembly.

One advantage of connector-based assembly is its increased flexibility: no end linkers are required, and new functions can be easily incorporated into the connector parts. The 5' and 3' connectors are selected independently to construct a Level 1 plasmid, allowing one to seven Level 1 plasmids to be combined into a Level 2 plasmid without needing the end linkers that other modular cloning systems require (Figure 6).
To design and assemble multigene constructs, the rule for selecting connectors is as follows: 5′Con(N) connects to 3′Con(N–1). For example, the 5′ connector of the second transcription unit (5′Connector_2) must connect to the 3′ connector of the first transcription unit (3′Connector_1). Additionally, the total number of transcription units in the final assembly must be considered, since the last unit must end with 3′Homology for the integration in the chloroplast genome and to properly connect to the Level 2 backbone.

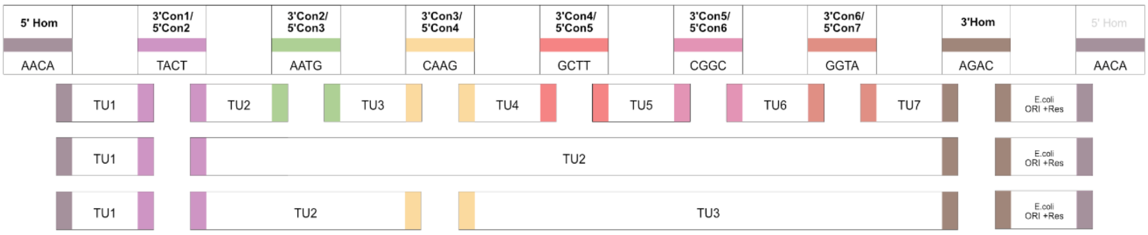

Figure 6 Connector parts define the position of transcription units (TU) within a multigene assembly. The position of the Level 1 transcription units within a multigene Level 2 assembly is dictated by the 4bp BsmBI overhangs withing the connector parts. Our cloning systems allows assembly for up to 7 TU´s but the number of TU´s can be chosen highly flexibly, by the selection of the specific connector parts in the Level 1 assembly. The first TU always includes the 5'Homology and the last TU always includes the 3'homology for the integration into the chloroplast genome

Example for the assembly of a Level 2 Multi-gene constructs

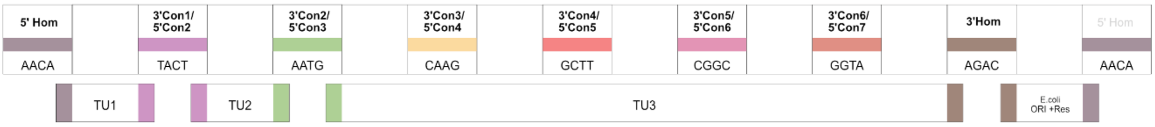

Figure 7 Example of a multigene assembly with three transcription units
The first transcription unit (TU1) uses a 5′ homology part (selected from one of four chloroplast integration sites) combined with 3′ Connector-1. The second transcription unit (TU2) is built with 5′ Connector-2 and 3′ Connector-2. The third transcription unit (TU3) incorporates 5′ Connector-3 paired with the corresponding 3′ homology part matching the chosen 5′ homology site.

An example of assembling a Level 2 multigene construct is as follows:

- **First Transcription Unit:**

  - Use a 5' Homology part for the connector position (selectable from four chloroplast integration sites).
  - Use 3' Connector-1.

- **Second Transcription Unit:**

  - Use 5' Connector-2.
  - Use 3' Connector-2.

- **Third Transcription Unit:**

  - Use 5' Connector-3.
  - Use the corresponding 3' Homology part that matches the chosen 5' Homology site.

These three Level 1 plasmids are subsequently combined with a Level 2 acceptor vector in a BsmBI Golden Gate reaction. The connectors selected during the Level 1 assembly determine the final order of transcription units in the multigene construct (Figure 7).

Additional features: Reversing of Transcription units

Our modular cloning system also includes a set of reverse connectors that allow the orientation of transcription units to be switched (Figure 8). This is particularly important because natural 3′UTRs often lack strong termination signals, leading to read-through transcription. Such read-through can produce antisense RNA that interferes with the expression of neighbouring genes. By inverting the direction of transcription units, this issue can be circumvented. Additionally, homology parts for plastome integration have been designed with an inversion option for all available integration sites as well.

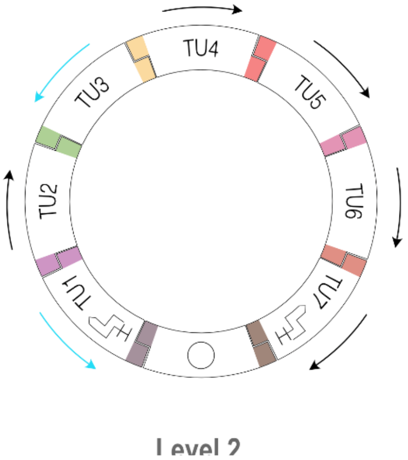

Figure 8 Reverse connectors allow switching of the transcription unit directionality. In this example TU1 and TU3 are in the reverse orientation

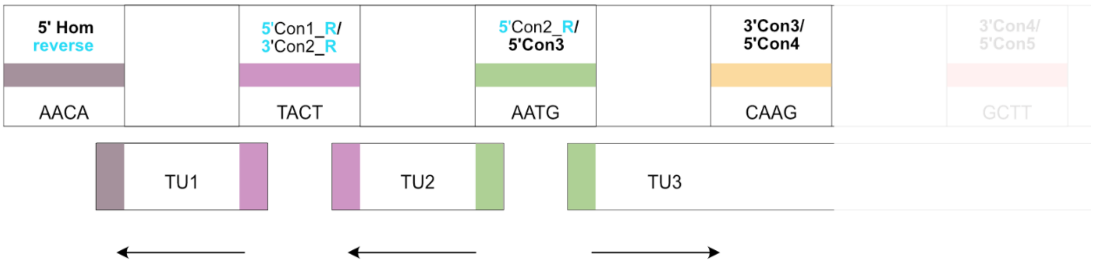

Figure 9 Example of reverse connector usage
For assembling reverse transcription units, reverse connectors and reverse homologies are employed. In the first transcription unit, a 5′ homology part is paired with a reversed 5′Connector-1 (serving as the 3′ connector). The second transcription unit uses a reversed 5′Connector-2 along with a reversed 3′Connector. The third transcription unit, oriented in the forward direction, is constructed using the standard 5′Connector-3 and 3′Connector-3. These three Level 1 transcription units are subsequently combined in a Level 2 assembly.

For reverse transcription unit assembly, the same naming scheme and rules apply, with each connector number corresponding to its position in the multigene assembly (Figure 9) . The only difference is that the inverted versions of the 5′ and 3′ connectors must be used. This approach allows the orientation of up to seven transcription units to be independently selected.

Additional features: Building polycistronic operons via Intercistronic expression elements (IEEs) and operon connectors.

One feature that has not traditionally been incorporated into the Modular Cloning approach is the construction of polycistronic operon structures. Standard Modular Cloning assemblies rely on separate transcription units composed of promoters, 5′UTRs, coding sequences, and 3′UTRs/terminators. To integrate polycistronic design into this framework, a method was needed to “skip” the promoter and terminator positions. This was achieved by extending the connector position to bypass these regions. In this work, 14 novel operon connectors were designed and constructed, enabling the assembly of operons with up to seven genes expressed from a single polycistronic mRNA. These new operon connector parts can be adapted for any Modular Cloning toolkit designed for bacterial chassis, thereby facilitating polycistronic expression.

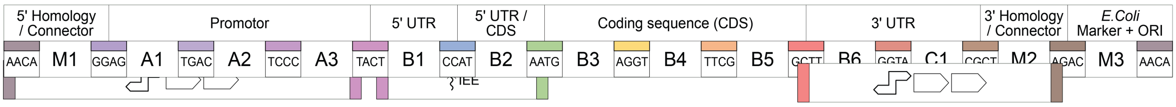

Figure 10 Operon connectors & IEE parts enable the construction of polycistronic multigene assemblies
Operon parts are included in our modular cloning system to “skip” promoter and 3’UTR/Terminator positions. When combined with intercistronic expression elements (IEEs), these parts enable the assembly of polycistronic multigene constructs.

Although polycistronic expression is native to chloroplasts due to their bacterial origin, primary polycistronic transcripts often require post-transcriptional processing into monocistronic fragments. To implement polycistronic gene expression for chloroplast engineering, the necessary intercistronic expression elements (IEEs) were integrated into our system in place of the 5′UTR. Combined with the operon connectors described above, this design allows for the construction of modular polycistronic constructs for chloroplast expression.
For example, a polycistronic construct can be built by designing the first transcription unit to include a promoter and a 3′ operon connector, while the second transcription unit contains a 5′ operon connector, one of 11 distinct IEE parts, and a 3′UTR. This configuration allows two genes of interest to be expressed from a single polycistronic transcript.

Additional features: Placeholder parts
To facilitate the characterization of entire libraries of a specific part type, it would be ideal to pre-build Level 2 assemblies with a placeholder that can later be exchanged with library elements. However, exchangeable parts have not been implemented within the Modular Cloning framework. Therefore we have designed and constructed placeholder parts that can be removed after the assembly process. In standard Golden Gate cloning, the recognition sites are eliminated in the final construct; therefore, these placeholders must be designed to retain BsaI recognition sites after assembly. This was be achieved by incorporating the reverse BsaI recognition sequence.

To simplify the identification of correct assemblies, a GFP cassette—expressed in E. coli—was selected as the placeholder. These placeholder parts have been designed and constructed for most positions defined by the standardized Phytobrick and MoClo syntax, making them universally replaceable in all MoClo toolkits that adhere to this standard.

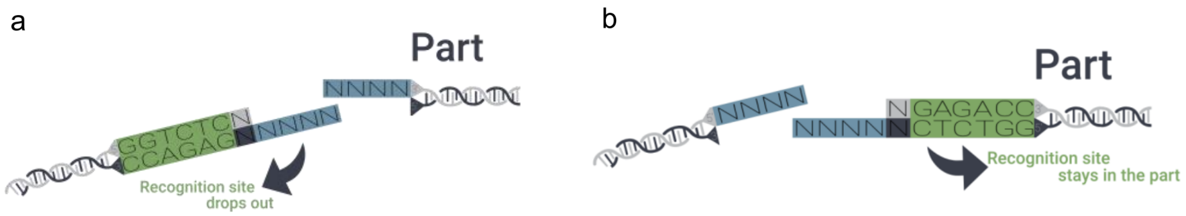

Figure 11 Design and function of modular placeholder parts
BsaI is a Type IIS restriction enzyme that cuts outside of its recognition sequence. In the standard Golden Gate cloning approach (a), the recognition site is removed during assembly. By contrast, using the reverse BsaI recognition sequence (b) allows the site to remain in the final construct. This retained recognition site enables the design of placeholder parts that can be removed in subsequent cloning rounds.

The placeholder parts are treated as regular parts during the Golden Gate assembly, with only a minor protocol adjustment required: the final step in the thermocycler should be a 16°C ligation step. Without this step, the placeholder would be excised from the assembled plasmid. Once assembled, colonies can be identified by green fluorescence due to the GFP cassette. The resulting Level 2 plasmid can then be used for the insertion of part libraries at the placeholder position.

Supplementary Tables

Supplementary Table 1: Mass Spectrometry parameters for CoAs.

Name	Precurser Ion	Product Ion	Collision energy [V]	Fragmentor Voltage [V]	Cell Accelorator Voltage [V]	Dwell time [msec]	Polarity
**CoA**	768.12	428	35	380	5	220	Positive
		261.1	35	380	5	220	Positive
**ACoA**	810.1	428	31	380	5	220	Positive
		302.2	31	380	5	220	Positive

Supplementary Table 2: Mass Spectrometry parameters for organic acids.

Name	Precurser Ion	Product Ion	Collision energy [V]	Fragmentor Voltage [V]	Cell Accelorator Voltage [V]	Dwell time [msec]	Polarity
**Citrate**	191	111.1<br>85.1	11<br>14	380<br>380	5<br>5	20<br>20	Negative
**Alphaketoglutarate**	145.1	101.1<br>57.2	5<br>8	380<br>380	5<br>5	20<br>20	Negative
**Malate**	133.1	115.1<br>71.2	8<br>14	380<br>380	5<br>5	20<br>20	Negative
**Succinate**	117.2	73.2<br>55.1	9<br>15	380<br>380	5<br>5	20<br>20	Negative
**Fumarate**	115.1	71.2<br>27.3	4<br>9	380<br>380	5<br>5	20<br>20	Negative
**Lactate**	89.2	89.2<br>71.3	0<br>10	380<br>380	5<br>5	20<br>20	Negative
**Pyruvate**	87.1	87.1<br>43.1	0<br>4	380<br>380	5<br>5	20<br>20	Negative
**Glycolate**	75.2	75.2<br>47.2	0<br>6	380<br>380	5<br>5	20<br>20	Negative
**Glyoxylate**	73.2	73.2<br>45.2	0<br>7	380<br>380	5<br>5	20<br>20	Negative

Supplementary Table 3: Mass Spectrometry parameters for amino acids.

Compound	Precurser	Product	Dwell time [msec]	Fragmenter Voltage [V]	Collision Energy [V]	Cell Accelerator Voltage [V]	Polarity
**Tryptophane**	205.1	188	20	380	7	5	Positive
		145.9	20	380	17	5	Positive
**Tyrosine**	182.1	165	20	380	6	5	Positive
		136.1	20	380	12	5	Positive
**Arginie**	174.9	116	20	380	12	5	Positive
		70.2	20	380	29	5	Positive
**Phenylalanine**	166.1	120.2	20	380	13	5	Positive
		103.1	20	380	32	5	Positive
**Histidine**	156.1	110.1	20	380	16	5	Positive
		83	20	380	30	5	Positive
**Methionine**	150.1	133	20	380	7	5	Positive
		104	20	380	7	5	Positive
**Glutamate**	148.1	84.1	20	380	17	5	Positive
		56.1	20	380	34	5	Positive
**Glutamine**	147.2	130.1	20	380	8	5	Positive
		84.2	20	380	17	5	Positive
**Lysine**	147.1	130.1	20	380	8	5	Positive
		84.1	20	380	19	5	Positive
**Aspertate**	134.1	88	20	380	9	5	Positive
		74	20	380	14	5	Positive
**Asparagine**	133.	87.1	20	380	17	5	Positive
		74.2	20	380	16	5	Positive
**Isoleucine**	132.1	86.1	20	380	8	5	Positive
		69.1	20	380	18	5	Positive
**Leucine**	132.1	86.1	20	380	8	5	Positive
		30.3	20	380	18	5	Positive
**Threonine**	120.2	74.1	20	380	8	5	Positive
		55.9	20	380	18	5	Positive
**Valine**	118.1	72	20	380	9	5	Positive
		55.1	20	380	23	5	Positive
**Proline**	116	70	20	380	15	5	Positive
		43.3	20	380	35	5	Positive
**Serine**	106.1	60.2	20	380	12	5	Positive
		42.2	20	380	11	5	Positive
**Alanine**	90	44.1	20	380	12	5	Positive
**Glycine**	76.1	30.3	20	380	32	5	Positive
		28.3	20	380	32	5	Positive

Supplementary Table 4: Mass Spectrometry parameters for energy metabolites.

Name	Precurser Ion	Product Ion	Collision energy [V]	Fragmentor Voltage [V]	Cell Accelorator Voltage [V]	Dwell time [msec]	Polarity
**NAD**	664.1	524	18	380	5	50	Positive
		428	26	380	5	50	Positive
**NADH**	666.1	649	17	380	5	50	Positive
		514	23	380	5	50	Positive
**NADP**	744.1	604	20	380	5	50	Positive
		508	32	380	5	50	Positive
**NADPH**	746.1	729	17	380	5	50	Positive
		135.8	40	380	5	50	Positive
**AMP**	346	96	24	380	5	50	Negative
		78.9	30	380	5	50	Negative
**ADP**	425.9	327.8	17	380	5	50	Negative
		133.9	22	380	5	50	Negative
**ATP**	505.9	407.9	21	380	5	50	Negative
		158.8	28	380	5	50	Negative

Supplementary Table 5: Sequences of all the parts of interest.

MoClo part	Part type	Sequence
**pME_Cp_0_1_001_psbH_5'Homology_Cr**	5' Homology	GAATTGTCAATTTTTAATGTACACTTAGTTGAAAGTGCC<br>CCTGTCCCCTTGGCCATATTTAACAGAAGTTATTTATAA<br>CGCAGCTGTTTTTTGGAGTCTATAAATTTATAACATCAG<br>TTACTATGGATTTCCCTTTAGTTTTATGGCCTAGGACGT<br>CCCCTTCCCCTTCGATGCTGGAGGCATCCTTTTACGGGA<br>CAATAAATAAATTTGTTGCCTCGCCTATCGGCTAACAA<br>GTTCCTTCGGAGTATATAAATATAGGATGTTAATACTG<br>CTATAAACTTTAGTTGCCCAATATTTATATTAGGACGCC<br>AGTGGCAGTGGTACCGCCACTGCCTGCTTCGCAGTATA<br>TAAATATAGGCAGTTGGCAGGCAACTGCCACTGACGTC<br>CTATTTTAATACTCCCAAGTTTACTTGCCTAGGCAGTTG<br>GCAGGCAACAAATTTATTTATTGTCCACTAAAATTTATT<br>TGCCCGAAGGGGACGTCCACTAAAATTTATTTACCCGA<br>AGGGGACGTCCTAATATAAATATGGGGATGTCAATGCT<br>CCGTTAGGAAGTAACTAACGTTTTTCAAATAAATTTTAT<br>CCCGGAGGGAAGTAGGCAGTAGCCCGCCACTGTCATC<br>CTTTAAGTGGATCTCTCGTCAGGCAATTTGCTTACACCT<br>TTAAATTAAAAATTAAATTTAAAGAAAAGTGAGCTATT<br>AACGCGTTTATCTTAACG

**pME_Cp_0_1_004_psbA_5'Homology_Cr**	5' Homology	CCTCTGTTGGAGCTTCTCCCCTTCCCCTTACGGGACATC<br>CCCTTCCCCTTACGGGATATTTATATACTCCGAAGGACG<br>TCCCCTTCGGGCAAATAAATTTTAGTGGCAGTTGCAAA<br>GTATTAATATCGTATATAAATATCCTGCCAACTGCCTAG<br>GCAAGTAAACTTAGGGATTTTAATGCTCCGTTAGGACG<br>TCCCCTTCCCCTTCGGGACGTCCCCTTACGGGAATATAA<br>ATATTAGTGGACGTCCCCTTCGGGCAAATGAATTTTAG<br>TGGCAGTTGCCTGCCAACTGCCGATATTTATATACTGC<br>GATAAACTTTAGTTGCCCGAAGGGGTTTACATACAATT<br>TATTTATTGTACCACTGCCACTAATATTTATATTCCCGTA<br>AGGGGACGTCCTCCTTCCCCTTCGGGCAAGTAAACTTA<br>GGAGTATGTAAACCTGCTAGCGCAGCAAATAAATTTTA<br>TTCTAAGTTTACTTGCCCGAAGGGGAAGGAGGAAGCA<br>GGCAGTTGCCTCCTTCCCCTTCCCCTTCGGGACGTCCCC<br>TTCGGGATTTTAATGCTCCGTTAGGAGGCAAATAAATT<br>TTAGTGGCAGTTGCCTGCCAACTGCCTCCTTCGGAGTA<br>TTAAAATAGGACGTCCCCTTACGGGAATATAAATATTA<br>GTGGCAGTTGCCTGCCAACTGCCTCCTTCGGAGTATTA<br>AAATAGGATGTTAATACTGCGGAGCAGGCAGTGGCGG<br>TACCACTGCCACTGGCGTCCTCCTTCGGAGTATGTAAA<br>CATTCTATATTTATATACTGCGATAAACTTTAGTTGCCC<br>GAAGGGGTTTACATACAATTTATTTATTGTACCACTGCC<br>ACTAATATTTATATTCCCGTAAGGGGACGTCCTCCTTCC<br>CCTTCGGGCAAGTAAACTTAGGAGTATGTAAACCTGCT<br>AGCGCAGCAAATAAATTTTATTCTAAGTTTACTTGCCCG<br>AAGGGGAAGGAGGAAGCAGGCAGTGGTGGTACCACT<br>GCCACTAAAATTTATTTACCCGAAGGGGACGTCCTTCC<br>CCTTCCCCTTCGGGACGTCAGTGGCAGTTGCCTGCCAA<br>CTGCCTAATATAAATATTGGGCAAGTAAACTTAGGAGT<br>ATATAAATATAGGATTTTAATACTCCGAAGGAGGCAGT<br>TGGCAGGCAACTGCCACTGACGTCCCGTAAGGGGAAG<br>GGGACAAATTTATTTATTGTCCCGTAAGGGAAAGTCGT<br>GGAGTATTTAATACAGCTAAAAGACTTAAAGAAACTTT<br>AGATTTAGCTATAAAACTTAAAGCGACAGGTACTTCCG<br>AAACGGTGGTTATTCCAGGCCAAACTTATGAAATGCAA<br>GTACCATCAGATATTGCTAGCATGGATACG

**pME_Cp_0_1_005_rbcl_5'Homology_Cr**	5' Homology	GTAAAAACACATAACTCCACGTAAGCGCATTTTCTTACA<br>ATCAAAGAATGCCAATTGTTCTTTGCTTGTTGTAAAAGA<br>AAAACTAGGCTTTTTCTTTGAAAACATTTCAGCATATTC<br>AACAACTTTTATAGATTGGTTTAGCGGATGATGGGACT<br>CGAACCCACAACCTCGAACTTGGAAGGATCGCACTCTA<br>CCGATTGAGTTACATCCGCTTTAGTATGTTACTATTTCT<br>TTTATTATAACTTATAAAATATAATACATAAAGATAAAT<br>TCTATAATAAAAAGCTAAGATTTTATTTTTCTGGCACAT<br>CGTAATTTATAAAGACAGGCAAATTTAAACAAAAGATA<br>ACTTTAGAACTTAATTTTAAAAATGTAAAATGATGTTTA<br>GGTATTTAACCTAAACACCATAAAAATAAAAACGATGT<br>TTATGCTATTCACATAAACATCATGAAAAATAAAAATTA<br>AAGTTTGTCAATAGTATCAAATTCGAATTTAATTTCTTT<br>CCAAACTTCACATGCAGCAGCAAGTTCTGGTGACCATT<br>TACAAGCTGAACGAATTACGTCGCCACCTTCACGAGCA<br>AGGTCACGACCTTCGTTACGAGCTTGAGTACAAGCTTC<br>AAGAGCTACACGGTTAGCTGCAGCACCTGGAGCGTTA<br>CCCCAAGGGTGACCTAGAGTACCACCACCGAACTGAA<br>GACATGCGTCATCACCGAAGATTTCAACTAAAGCTGGC<br>ATGTGCCATACGTGAATACCACCTGAAGCAACTGGCAT<br>AACACCTGGCATTGAACACCAGTCTTGAGTGAAGTAAA<br>TACCACGGCTACGGTCTTTTTCAACGTAGTCATCACGCA<br>TTAAGTCTACGAAACCTAGAGTAACTTCACGTTCACCTT<br>CTAGTTTACCTACAACAGTACCAGAGTGAAGGTGGTCA<br>CCACCAGACATACGAAGAGCTTTAGCAAGAACACGGA<br>AGTGAATACCGTGGTTACGTTGACGGTCAATAACCGCG<br>TGCATAGCACGGTGGATGTGTAGAAGAAGACCGTTGT<br>CACGACAGTAGATAGCTAATGAAGTGTTAGCTGTGAA<br>ACCACCTGTTAAGTAGTCGTGCATAATAATAGGTACAC<br>CTAATTCTTTAGCACATACTGCACGTTTCATCATTTCTTC<br>ACAAGTACCAGCAGTAGCGTTTAAGTAGTGACCTTTAA<br>CTTCACCTGTTTCTGCTTGAGCTTTGTAAATAGCTTCAG<br>CAACGAAAAGGAAACGGTCACGCCAACGCATGAATGG<br>TTGTGAGTTTACGTTTTCGTCGTCTTTAGTAAAGTCAAG<br>ACCACCACGTAAACATTCATAAACTGCACGACCGTAGT<br>TTTTAGCTGAAAGACCTAATTTAGGTTTGATTGTACAAC<br>CTAAAAGACCACGACCATATTTGTTTAATTTGTCACGTT<br>CTACCTGAATACCGTGTGGAGGACCTACGAATGTTTTA<br>ACGTAAGCAGGTGGAATACGAAGGTCTTCAAGACGTA<br>GAGCACGTAAAGCTTTGAAACCGAATACGTTACCTACA<br>ATAGAAGTGAACATGTTAGTTACTGAACCTTCTTCGAA<br>TAAGTCGATTGGGTAAGCTACGTAAGCAATGTATTGGT<br>TGTCTTCACCCGGAACTGGTTCGATATCGTAACAACGA<br>CCTTTGTAACGGTCAAGACTTGTTAAACCGTCAGTCCAT<br>ACTGTAGTCCATGTACCTGTTGAAGATTCAGCAGCTAC<br>AGCAGCACCACATTCTTCAGGTGGAACACCTGGTTGTG<br>GAGTCATACGGAATGCAGCTAAAATATCAGTATCTCTT<br>ACTACGTAATCAGGTGTGTAGTATGTTAAACGGTAGTC<br>TTTTACACCGGCTTTGAATCCAGCACCTGCTTTAGTTTC<br>TGTTTGTGGAACCATTTATATAAATAAATGTAACTTCTT<br>TTGACGATCCTAAAATAATCTGTCCGGAAATATAATTTA<br>AAAATTGTTGAAAAATTTTAAATACATTTAAATATATTA<br>TATATTTAGAAATAAAAAATAATGTAAACTAGCAAATT<br>TATGTACTTGTCTTATCAGTCGAGCATAGAAAGGGCCT<br>TTAGGTATATGTCGGTCGTCTTACGCAGTTTACTTAC

**pME_Cp_0_1_007_petB_5'Homology_Cr**	5' Homology	GATTGACGTTTTGCACTAGCGCCGCCAGATAAATTTTCT<br>TTTTTTACTGTATCTTTTCCTTTTGTTTTTGCGGTGCCAC<br>TTTTAAATAATCCACTTTTTTCCATTTCTTTTTTTTCCAAC<br>TCTTTAGAACGATCTTCCATATGGATATTAATTAATAAA<br>CCACAAATTTGGTTACATAATTCATCATCTAAATATTGC<br>ATTAAAAATACCATACGACGACGGAAAATAAAGTTATA<br>AATATCAGTCCACTGTGCAGGTAATTCTTCACCCCAACA<br>ATAAATAATACGAGGTACTCCAATCGGCATAAATTACT<br>TTGTTAATAAAATGTTGTGTTTTATTAATACTAGTCTAA<br>TATCCTAGTAGATAGAGCTTTTATTTGCACGTAAATAAG<br>CTCTGCGAGTGCTACTGTAAAAATTAAAGTACCGTTTA<br>CAGGCTCCTTTGAAGCAAATAAAAATTTTAAACCAAAA<br>TGGTTTAAAAAATAGATAAATTCAAACAAATATTGGCT<br>CTACGTTAAACTTAAAATGCCTTCGGCCGGATTTGAAC<br>CGGCACGCCTTTCAGCACTGGTTCCTAAAACCAGGATG<br>TCTACCAGTTCCATCACGAAGGCTAAAAAATATTACTTT<br>TATAATATCATACTTAATTTTTTTTGTCTAGTTAAAAACA<br>CTAAATGTGTTAAATGCATACACAATTTTTTGTTTAGAC<br>TAGAGACTTGGGGTTAGTTCCGATCCTTTAACCAGAAT<br>ATAACTATGGTTAATTTATAGCTACCCCTTATCAATGGG<br>CCAACGGGGGATCTGGCAGAAACCGC
**pME_Cp_0_1_010_psbD_5'Homology_Cr**	5' Homology	GGTTTGGAATAATAAAAATCGAAAAAAAGAATTCACTC<br>AAAGAAAAAAACGTTTGACTTTAATTTCGGATAAACTG<br>CATAAAATTTATAGTTTCTACAAAGTTTATCTAGTTTAG<br>GGATATAAATATAGAGCAAATCAATTGAATTGCTATAC<br>CCAAAGAGGCAGAGATGTCCTTAAAAATAAATTAAAGT<br>TTTTTCAGACCTAGCTGATTTAATACATTTTGTGTTCTAT<br>TAGACTTGTTAATGTATTTTTTTTGAGTATCCTTAATAAT<br>ATTAAAATATTATTTTTACACGTTACATATATTAGTATTT<br>TATTTTAACGATAAACGCTTTGTGGTTATTAAAATGGAT<br>TTACCTCTGTTAGGCTATGGAACTTGATAAAACCGTCCC<br>TTAAGGGGATGTCTTACAAGGATTTAGGATCTGCCATT<br>TGTATTAAGAGTATAGATTTTAATAAAAAACCAAAAGA<br>TTTTTAAAAAAGTGGATTGTAAAAATCTTTTTATATGAT<br>AAACCACTTCTGTCTTTTTAGACATAATTGAAACTAAAA<br>AGTAAGTTTTTCTGAAAAAATAAAAAGAACTAAAGCAA<br>AATGCAAATAATTGCCTTATACTGCTTTGATTTTTAAAA<br>GTGCTTATACTTTTCAACTACAGTGTAAAAGTATCTTTT<br>TAAACTATAAAAATATATACGTCTTCGTGTCGCACCCAC<br>TTAACCAAATGAAAATGATACCTAATTGACCAAAGTGT<br>GCACTGAATACTTTTCTAGAAATTTCTTCTAGATCACTT<br>GTATGACTGTCAAAGTCATGAGCATCTGCGTGAAGGTT<br>CCAAATCCAAGTTGTTGTGTTTGGACCTTTTGACAGAGT<br>ACGTGAAAAATGTCCTGGTTTAGTCAGGTAGGATTTGT<br>TTTTTTATTTTTTAATATTTTAAATCCTCCTAAAGAACCG<br>TACATGCGAGTTTTTTCGCATACGGCTCAAGCACTCATG<br>TGATTTTTAGCCCCAAAGGGGCATTTTCTTTTAAATAAT<br>AAAGTAATATAAATTACCAATACTAATTAGTATTGGTA<br>ATTTATATAAAAATATATTATAGAGCGTTACCACGTGGT<br>AATACTTCTTCAGGGAATACTAAACGTTCGTGTGGTTG<br>GTCTTGAGCAGCCATCCAAGCACGAATACCTTCGTTAA<br>GAAGAATGTTTTTAGTGTAGAATGTTTCGAATTCAGGG<br>TCTTCAGCAGCACGAATCTCTTGTGATACGAAGTCGTA<br>AGCACGTAAGTTTAGAGCTAAACCTACAACACCAATAG<br>CACTCATCCAAAGACCAGTTACTGGAACTAATAACATG<br>AAGAAGTGAAGCCAACGTTTGTTAGAGAAAGCAACAC<br>CGAAGATTTGTGACCAGAAACGGTTAGCAGTAACCATA<br>GAGTATGTTTCTTCAGCCTGTGTAGGGTTGAATGCACG<br>GAATGTGTTAGCACCGTCACCGTCTTCGAATAATGTGT<br>TTTCAACAGTAGCACCGTGAATAGCACATAATAAAGCA<br>GCACCTAAAACACCAGCAACACCCATCATGTGGAATGG<br>GTTAAGTGTCCAGTTGTGGAAACCTTGGAAGAATAAAA<br>TGAAACGGAAGATAGCAGCTACACCGAAACTAGGTGC

		AAAGAACCAACCTGATTGACCTAATGGGTAAATTAGGA<br>ATACTGAAACGAATACAGCAATTGGTGCTGAGAAAGC<br>AATTGCGTTGTATGGACGTAAGTTTACTGAACGAGCAA<br>TTTCAAACTGACGAAGCATGAAACCAATTAAACCAAAT<br>GCACCGTGTAAAGCAACGAATGCCCATAAACCACCAAG<br>TTGACACCAACGAGTGAAATCACCTTGAGCTTCTGGAC<br>CCCAAACAAATAGAAGAGAGTGAGCCATACTGTTAGC<br>AGGTGTAGAAACAGCTGCTGTTAAGAAGTTACAACCTT<br>CTAAGTAAGAAGTAGCTAAACCATGCGTATACCATGAA<br>GTAACGAAAGTAGTACCAGTTAACCAACCACCTAATGC<br>AAAGTAAGCACAAGGGAATAGTAATAAACCTGACCAA<br>CCTACGAATACGAAACGGTCTTGACGAAGCCAGTCATC<br>AGCGTCATCGAACCATGTGCGTTTCTCTTGATATGTACC<br>GATCGCAATTGTCATTGCGTGTATCTCCAAAATAAAAA<br>AACAACTCATCGTTACGTTAAATTTATTATTATTTAATTT<br>TAATCATTGTGTATTTAATATTATAACTTATATAAAATA<br>AAATTAAAAATAAGCATTTTTTACACACATATTTTTAAA<br>TAAATCTTTAAACGGGTTATATATAGTTATATATATGGG<br>ACTAGAACTGCTTTGTGCATAGTC
**pME_Cp_0_2_001_16s_Prom_Cr**	Promoter	CAGGCAACAAATTTATTTATTGTCCCGTAAGGGGAAGG<br>GGAAAACAATTATTATTTTACTGCGGAGCAGCTTGTTA<br>TTAGAAATTTTTATTAAAAAAAAAAATAAAAATTTGACAA<br>AAAAAAATAAAAAAGTTAAATTAAAAACACTGGGAAT<br>GTTCTAACAATCATAAAAAAATCAAAAGGGTTTAAAAT<br>CCCGACAAAATTTAAACTTTAAAGAGT
**pME_Cp_0_2_003_psaA2_Prom_Cr**	Promoter	TTTAAAATAAGTAAAAATAATTTGTAAACCAATAAAAA<br>ATATATTTATGGTATAATATAAC
**pME_Cp_0_2_025_psbA_Prom_Nt**	Promoter	GATCTACATACACCTTGGTTGACACGAGTATATAAGTC<br>ATGTTATACTGTTG
**pME_Cp_0_2_035_rrn16_short_Prom_Nt**	Promoter	ATGAGAATGGATAAGAGGCTCGTGGGATTGACGTGAG<br>GGGGCAGGGATGGCTATATTTCTGGGA
**pME_Cp_0_2_036_rrn16_long_Prom_Nt**	Promoter	GCTCCCCCGCCGTCGTTCAATGAGAATGGATAAGAGGC<br>TCGTGGGATTGACGTGAGGGGGCAGGGATGGCTATAT<br>TTCTGGGAGCGAACTCCGGGCGAATACGAAGCGCTTG<br>GATACAGTTGTAGGGAGGGATTT
**pME_Cp_0_2_057_Synthetic_promoter_01**	Promoter	CGAAATATAATTTGACATAAAAACTATATAATTTTAAAT<br>TAAATAC

**pME_Cp_0_2_058_Synthetic_promoter_02**	Promoter	AATAATTTGATTTGACAAAAAATAAGTTACAAGTTAAATTAAAATC
**pME_Cp_0_2_059_Synthetic_promoter_03**	Promoter	TATATTGAATTTTGACATAAATAAGTTATAACAATAAATTAATAAC
**pME_Cp_0_2_060_Synthetic_promoter_04**	Promoter	ATGATTACTATTTGACATTTATAAATTTTATATTTAAATTAGAAAC
**pME_Cp_0_2_061_Synthetic_promoter_05**	Promoter	ATAAACATATTTTGACATAAAATAATATAATATCTAAATTACTAAT
**pME_Cp_0_2_062_Synthetic_promoter_06**	Promoter	CAAGCAATCAATTGACATTATAAATTATATAAATTAAATTAAAAAC
**pME_Cp_0_2_063_Synthetic_promoter_07**	Promoter	CAGATAATAATTTGACAATTTAAAAAAATATATATAAATTCTAATC
**pME_Cp_0_2_064_Synthetic_promoter_08**	Promoter	TCATAAAAGTTTTGACAAAATTGTATTATAAATTTAAATTAATTAG
**pME_Cp_0_2_065_Synthetic_promoter_09**	Promoter	ATAATATATATTTGACATACATTTATTTTTAAGCTAAATTAACATT
**pME_Cp_0_2_066_Synthetic_promoter_10**	Promoter	AATTAATAAATTTGACATTATATTTTACATCTATTAAATTCAGTAC
**pME_Cp_0_2_067_Synthetic_promoter_11**	Promoter	AAAATAAAAATTTGACAAAAAAATAATATTTACATAAATTAAAAAC
**pME_Cp_0_2_068_Synthetic_promoter_12**	Promoter	AAAATAAAAATTTGACATTATTATTTTAAAAAGTTAAATTAAAAAC
**pME_Cp_0_2_069_Synthetic_promoter_13**	Promoter	AAAATAAAAATTTGACAATTTATTAAATTATACTTAAATTAAAAAC
**pME_Cp_0_2_070_Synthetic_promoter_14**	Promoter	AAAATAAAAATTTGACAAAAAAAAAATAAAAAAGTTAAATTTTCATT
**pME_Cp_0_2_071_Synthetic_promoter_15**	Promoter	TATTATTGACAAAAAAAAAATAAAAAAGTTAAATTAAAAAC
**pME_Cp_0_2_072_Synthetic_promoter_16**	Promoter	AATGGCAATTTGACAACAAGCAAGTAAAGGGTTAAATTAAGAAC
**pME_Cp_0_2_073_Synthetic_promoter_17**	Promoter	AAAATAAAAATTTGACAAAAAAATATATTTATGGTAAATTAAAAAC
**pME_Cp_0_2_074_Synthetic_promoter_18**	Promoter	AAAATAAAAATTTGACAACCAATAAAAAATATATTTATGGTAAATTAAAAAC
**pME_Cp_0_2_075_Synthetic_promoter_19**	Promoter	AAAATGTGGGATTGACAAAAAAAAAATAAAAAAGTTAAATTAAAAAC
**pME_Cp_0_2_076_Synthetic_promoter_20**	Promoter	AAAATAAAAATTTGACAAAAAAAAAATAAAAAAGTTATAATAAAAAC
**pME_Cp_0_2_077_Synthetic_promoter_21**	Promoter	ATTTTTATTGACATTTTTTTTATTTTTTCATAAATTTTTTTG
**pME_Cp_0_2_078_Synthetic_promoter_22**	Promoter	TTCAGGTTTTATTGACAAAAAAAAAATAAAAAAGTTAAATTATTCTC
**pME_Cp_0_2_079_rbcL_promoter_Cr**	Promoter	ACAAGTACATAAATTTGCTAGTTTACATTATTTTTTATTTCTAAATATATAATATATTT
**pME_Cp_0_2_080_tRNA_Glu2_promoter_Cr**	Promoter	CAACTAAGTGTACATTAAAAATTGACAATTCAATTTTTTTTAATTATAATATATAT
**pME_Cp_0_2_081_WendyII_promoter _Cr**	Promoter	AATATATTTATTTGACATAAATATATTTATGTGATATAATATATTT
**pME_Cp_0_2_082_pDummy _Cr**	Promoter	CCCCTGGCGCCCCTT

**pME_Cp_0_2_083_Promoter_Placeholder_Cr**	Promoter	GAAAGTGAAACGTGATTTCATGCGTCATTTTGAACATT<br>TTGTAAATCTTATTTAATAATGTGTGCGGCAATTCACAT<br>TTAATTTATGAATGTTTTCTTAACATCGCGGCAACTCAA<br>GAAACGGCAGGTTCGGATCTTAGCTACTAGAGAAAGA<br>GGAGAAATACTAGATGCGTAAAGGCGAAGAGCTGTTC<br>ACTGGTGTCGTCCCTATTCTGGTGGAACTGGATGGTGA<br>TGTCAACGGTCATAAGTTTTCCGTGCGTGGCGAGGGTG<br>AAGGTGACGCAACTAATGGTAAACTGACGCTGAAGTT<br>CATCTGTACTACTGGTAAACTGCCGGTTCCTTGGCCGA<br>CTCTGGTAACGACGCTGACTTATGGTGTTCAGTGCTTT<br>GCTCGTTATCCGGACCATATGAAGCAGCATGACTTCTT<br>CAAGTCCGCCATGCCGGAAGGCTATGTGCAGGAACGC<br>ACGATTTCCTTTAAGGATGACGGCACGTACAAAACGCG<br>TGCGGAAGTGAAATTTGAAGGCGATACCCTGGTAAAC<br>CGCATTGAGCTGAAAGGCATTGACTTTAAAGAGGACG<br>GCAATATCCTGGGCCATAAGCTGGAATACAATTTTAAC<br>AGCCACAATGTTTACATCACCGCCGATAAACAAAAAAA<br>TGGCATTAAAGCGAATTTTAAAATTCGCCACAACGTGG<br>AGGATGGCAGCGTGCAGCTGGCTGATCACTACCAGCA<br>AAACACTCCAATCGGTGATGGTCCTGTTCTGCTGCCAG<br>ACAATCACTATCTGAGCACGCAAAGCGTTCTGTCTAAA<br>GATCCGAACGAGAAACGCGATCATATGGTTCTGCTGG<br>AGTTCGTAACCGCAGCGGGCATCACGCATGGTATGGA<br>TGAACTGTACAAATGACCAGGCATCAAATAAAACGAAA<br>GGCTCAGTCGAAAGACTGGGCCTTTCGTTTTATCTGTT<br>GTTTGTCGGTGAACGCTCTCTACTAGAGTCACACTGGC<br>TCACCTTCGGGTGGGCCTTTCTGCGTTTATA
**pME_Cp_0_3_001_atpA_5'UTR_Cr**	5'UTR	TTTACCTTTTTTTTAATTTGCATGATTTTAATGCTTATGC<br>TATCTTTTTTTATTTAGTCCATAAAACCTTTAAAGGACCTT<br>TTCTTATGGGATATTTATATTTTCCTAACAAAGCAATCG<br>GCGTCATAAACTTTAGTTGCTTACGACGCCTGTGGACG<br>TCCCCCCCTTCCCCTTACGGGCAAGTAAACTTAGGGATT<br>TTAATGCAATAAATAAATTTGTCCTCTTCGGGCAAATGA<br>ATTTTAGTATTTAAATATGACAAGGGTGAACCATTACTT<br>TTGTTAACAAGTGATCTTACCACTCACTATTTTTGTTGA<br>ATTTTAAACTTATTTAAAATTCTCGAGAAAGATTTTAAA<br>AATAAACTTTTTTAATCTTTTATTTATTTTTTCTTTTTT
**pME_Cp_0_3_002_atpA_5'UTR_Cr**	5'UTR	ACTATATAAATACATTTACCTTTTTATAAATACATTTACC<br>TTTTTTTTAATTTGCATGATTTTAATGCTTATGCTATCTT<br>TTTTATTTAGTCCATAAAACCTTTAAAGGACCTTTTCTTA<br>TGGGATATTTATATTTTCCTAACAAAGCAATCGGCGTCA<br>TAAACTTTAGTTGCTTACGACGCCTGTGGACGTCCCCCC<br>CTTCCCCTTACGGGCAAGTAAACTTAGGGATTTTAATG<br>CAATAAATAAATTTGTCCTCTTCGGGCAAATGAATTTTA<br>GTATTTAAATATGACAAGGGTGAACCATTACTTTTGTTA<br>ACAAGTGATCTTACCACTCACTATTTTTGTTGAATTTTA<br>AACTTATTTAAAATTCTCGAGAAAGATTTTAAAAATAAA<br>CTTTTTTAATCTTTTATTTATTTTTTCTTTTTT
**pME_Cp_0_3_003_atpB_5'UTR_Cr**	5'UTR	ATATATATAGTTAAATGAAAAAACTAAAAAATAAGCGT<br>TAGTGAATAATACTTTTTATATATAAATTCTACTATATGT<br>TTGAGCTTCCTTTTATATAAATTTTAAATTTATAACAAGT<br>TACACTCTCAGTTAAAGGTGCCTTTTTGAAAGTAAAGC<br>CACAATGTTTACATACTAGGCAGTGGCGATACCACTGC<br>CACTGGCGTCCTCCTTCGGAGTATGTAAACATGCTAAG<br>TTTACTTGCCCGAAGGGGAAGGAGGAGGTTCTTATTTC<br>AAATTACCTAAGATAAAGTTTCCTTCGGGGTTTATACGT<br>AAGAATTTAAGTAAAATAAGTTAAAATATGA

**pME_Cp_0_3_004_chlL_5'UTR_Cr**	5'UTR	ATATATAAAATAAAAAAAACGTTAGTAATTCAAAAGTTTTAATATTATACAATTGAACTATTATGTATTAAATATAAGAATGTCACCTCTTACCATATTTCTATACTCCAAAGTAACTTTTTACATAAATGTCCCCTCTGGGGCTGCCTCCTTCCCCTTCCCCTTCGGGTATATAAATATAGGGCAAGTAAACTTAGCATAAACTTTAGTTGCCCGAAGGGGTTTACATACTCCGAAGGAGGACAAATTTATTTATTGTGGTACAATAAATAAATTGTATGTAAACCCCTTTCGGGTAACTAAAGTTTATCACGGCAATAAGTTTCTGCTTACGCAGTATTATATCTGACGCAGTATTATATAAGAAGTTGGCAGGATAAAAATGTGTAAGTATGGCAATCTTTTAAAATAGTGTTCAATTCATTTAAGGCAGATAAAAAGAAAAAAGTCCACAGGATTTAATTTTGAATAGTTCTCTATCAAAAAAAAGGTTTGCCGAACA
**pME_Cp_0_3_005_petB_5'UTR_Cr**	5'UTR	ATAACTTTAATTTAAACTTTAAATAATTAAGCTACACATTTTTTTAGTCTTAAGAAAGCCTAATGGTCATGTCACAATCTTATAAAAATTTTAT
**pME_Cp_0_3_006_psaA_5'UTR_Cr**	5'UTR	ATATGATGTAAAAAAAACTATTTGTCTAATTTAATAACCATGCATTTTTTATGAACACATAATAATTAAAAGCGTTGCTAATGGTGTAAATAATGTATTTATTAAATTAAATAATTGTTATTATAAGGAGAAATCC
**pME_Cp_0_3_007_psbA_5'UTR_Cr**	5'UTR	ACCATGCTTTTAATAGAAGCTTGAATTTATAAATTAAAATATTTTTACAATATTTTACGGAGAAATTAAAACTTTAAAAAAATTAACAT
**pME_Cp_0_3_008_psbE_5'UTR_Cr**	5'UTR	ATAATACATTGATTATAAAGCAGACAAATTGTTGAAAAAGCTAAGTAAGCACACTTAGGAGGTATTTTTT
**pME_Cp_0_3_009_psbF_5'UTR_Cr**	5'UTR	ATTATATTTATTTTAAACTAATATTTACATAAATTTTAAACGAGTTAGCTTAATACAAAAGGTAAAATTACCCTATTTATTTTTATTAATT
**pME_Cp_0_3_010_psbK_5'UTR_Cr**	5'UTR	TTTGATTTTAGAAAGAAAAAACGAGCTTTAAGGTGAGCTTATTTTGTTGCGTGTAAATTTTTAAAATCTAAGGTGTATAGACAAAAATCTACATTTTCATATGCTAAAAACATACTCTTTACGGGTACGCGAATGTTAGGTAAATTTTCACAACTAACTCTATGGTTGTGGGAAGAAAACCAAATACATAGAGATATTTTTAAAAAGATATCTCTCACTTTAATAGATTTTATTATAAATACTATCAACAATTTCTTAAACTTTTTAATGAAGGATATTT
**pME_Cp_0_3_011_petA_5'UTR_Cr**	5'UTR	GAGAAGAAAAAAAATAAAATTTAAATAGTAATTATGAATGTAAATTACTTATGCTTACTTTTTCAGCTAGAATAAACTTGTTGAGGCTGCTTAGTTGCACTTTCCTCGTACCCGTATGGGTAGGGGTTTATCCACGAAAAAATTCTATTTTAAAATAGGAGTCCAGTTGAAAAGCAACTGGAATCCCCTTATAGATAAATTAATATCTATTTTAAAATTGAATAGTTTTTATTCTAGTTTCGTTTTAAGATTAATAAAATT
**pME_Cp_0_3_012_petD_5'UTR_Cr**	5'UTR	AAAAATTTTTAGCATGTAAACATTAGAAATACAGCATAATTGGAGTAAAAGAAAAATATTAAACTTTTACATTGAAAAGTTTATGGCGTTTTGGCTTTATAAAATAAAAAACTTTTCGGAACGGCTAAACCATATTTATTATCATTAAAATTTATTTGCCCGAAGGGGACGTATCCGAAATAGAACAAATGCCAAAATCTACTAAATTAGATTAAAATAGTTTTAAAAATGGATAGATTTAAATAAAAAACAGAAGTAAAATGTAATTCTGTCCCTTTTTACAGGGTGGTATCTCTAAAAACCAGGGCTTGCCCAATCAACAATTTAAAGCTTATTTAGTTTTATTGAAAATTAACGGATAAATAAAT

**pME_Cp_0_3_013_psaB_5'UTR_Cr**	5'UTR	CAAATGTTACTCGTGGATGGCTTTGGTATTTAGGCGTAGCATGCAGTAAATATATGGCCAATGGCTCCTCTTTTAGCAAGTTCTCAACTTAAAATGCAATCTCTTAACGAGAATCTAAAGCTCAGAAATATATTTGAATTAAAATTTCCCACAGGATTATGGCGTAGTCATAATATCAACTAAAAAATCTTTTTAAATTTTAAAATTTACTTTTTTACGCTTTTGTATGCAAAGTTTGCTTTGCACCTGAATAGTTTTATTAAATTTTTATTTAATGGTAGTTTAATAGTAGTAATTTACTTCAATTAAACAAAAAAAAATCCTAATTGTTTATCCCTTTAAAAGAGCGCTTAAAGTTTTTTTACTTAGTGAAGTAAAAATACCGCTCCCTTCTGGTATTTTTTCTTTTGATTTAACAATTAGCATTTTAACCTTTTACTTTTCTCTCAGTGTTATACTGCTTAAAAGTTTTTAGGTCATTAGATAATATTTAATAATATTACATATAGGGAGTAAGACAATTTT
**pME_Cp_0_3_014_psbB_5'UTR_Cr**	5'UTR	AATTTAATTTAAAATCTTAAAAAATTTTTTTTAACATAGTTAATTAAATTTTTTAAGATTTTAAATTAATGTAGAAATTAATTTCTTTTTATTTTTATAACCTTGTAATAATTAAGTAAAAAAATCAGTAAAAAAATTTTTT
**pME_Cp_0_3_015_psbC_5'UTR_Cr**	5'UTR	TATTTTAAGTGTTACAAAGAAATTGAATTTAATCTCAAAATACATTTTTGAAATTTTTTTATATTATTTTTTAGTATCTAAAAAAAAAGCATTTGCTATTAGTAGGACAGTTGTCATGTTAATGGAGCTTACTTTACCTTAGTATATAAGGTAATTTAAATATAAAAAGATTTAAAATTATGTTTTTAATTAACAATTAAAAACGATTAAGTTGTTTTAAATCAACTTTATTTAATAAATATGATCTAAATTATTTTTAAAATTACTTTCCAACTGCAATTTTTAGCACAAATAAATTAGATCATATTTAAAATACAAGTTATAAAATTCTATTCTTAAAAACAACATTATTTTTACTCTGTGTTTTAAAAATGTTGGAGATAAGCTTTTGTCCTTAATTCTTTTGGATTAATATGGTACAAGAGGATTTTTGTTGTTAAAGGTTTCACCATATTTATTATTTATATGGGTGGACGTTAAAAATTAAAAATTTTTATTTAAGCAAAACTTTATAGAAATCAAAATAATTTGTACGGAGGTAATGCAAA
**pME_Cp_0_3_016_gene10_5'UTR_T7**	5'UTR	AGACCACAACGGTTTCCCTCTAGAAATAATTTTGTTTAACTTTAAGAAGGAGATATACAT
**pME_Cp_0_3_017_ccsA_5'UTR_Cr**	5'UTR	TTAAGAATTGGTAGTTTTTGAACTAAACAGTGACATAAAACTGGTTAAAAGACATCCCTGTAAGAGAAATGCATATGGTGAATTACACAATAAATTTTAAAAATAAAGCTCTGACAAGCCTCTTCCCCTTTGCGATATACATGCTCTGTTAGGATATAATTCTGCCTAAGTTCCTAACAAAGGGTTTACTTTTTCTCGGGGAATGGTTTTGCTACTTTGTTTTAAATCTTTTTCAAAGAATGTCAATCTCCTATAAAGGGAGGAACATTACTCATGG
**pME_Cp_0_3_018_psaC_5'UTR_Cr**	5'UTR	TGGACCAAAAGCAGTCATTCAAATATAGGTATGAGGAGAGAAATATATGCGCCGCAAGGCGCACTGCCACTGCTTAATATAAATACCTTGTTTAAAGTCGATTCTCAATCTTCTTTTTGATATGGAGATGACATATTTAGCACAACGCAAAAATATAGCATCTTTAAAAATTAACTTAAAATTTAAAT
**pME_Cp_0_3_019_rbcL_5'UTR_Cr**	5'UTR	AAATGTATTTAAAATTTTTCAACAATTTTTAAATTATATTTCCGGACAGATTATTTTAGGATCGTCAAAAGAAGTTACATTTATTTATATAA
**pME_Cp_0_3_020_rpoC1_5'UTR_Cr**	5'UTR	ATAGTGTATGTAATTGCGGATGTATAAATTCACTAAAATGTTATATCAACCCTTAAAGGGCCCCGAAATGGTTCATTATACCCGAAGCGAATTGTAGTTACTACCTGCTACCCTCTTCAGGTATATGTACCAACTGGCGTCCCCTTACAGGTACACAGTAAGTAATAAGTTACTACTTCCTCGAGACAGAACCAATGTCCCCCTTCGGGCTGGCACGGCCATGTTATTTATATATCCAAATGAGGTAGCCTTGTTAGCTATACATAAAATATGGTTTTTTCTGGTTAAAAAAATCTTAAGCTTAATAAAGCTTTATATGTTAGTTCGACTTTGTAGCCCCGCAGTCGT

		TTATGTGTGAGATCTAATGCTTTATCGGGATTAGTTTCA AAGGACTTTACCCACATAGAACTACCATATAAAATTGC ATTTTATTAAACATTAAGCTACAAGAAAATCCTTTTAAT ATTTATT
**pME_Cp_0_3_021_rpoC2_5'UTR_Cr**	5'UTR	TTAAAATTATATAAAAAGCTAGTTATCCTGCTACCTCTA CCTTACCGACTCCTAACAGAATATAAATATACTCCGACG ACGTTTAGTATTATACTTAGAGGCAGTTGAAATCTAGTT CCTCCGGGAGTGCTTGGTAACCAATTTTCAATCAAATTA ACCAAGATAAATATAAAAAATGATTTTAAAACGTTTGA CTTTAATCTTCCATTCGTTAT
**pME_Cp_0_3_023_tufA_5'UTR_Cr**	5'UTR	AACAGAACTACTGTAGTTTTTTAAATTATACCTAAACCT GAAAAATTGGATTATATAGCAATAACACTATTAAGAAC TATATATTAAAATGTCAATACTATCCCGTAGGGACTATG AAACTAAAACACCTATCCGAATGTTAATGCTTAGTGAG CAAAGTATGCAGTATTTATCTACAAAAAAGGAGAAACT CTTACCTCTGGTTAGAGGTTTCTTTTTTAATGTTTGAAG CATTAACAATCTAATTCCAAAAGAAGTGACTAAATAAT AATTTATTAAATAACACAT
**pME_Cp_0_3_024_clpP_5'UTR_Cr**	5'UTR	TTTAAGTTTAACGTAGAGCCAATATTTGTTTGAATTTAT CTATTTTTTAAACCATTTTGGTTTAAAATTTTTATTTGCT TCAAAGGAGCCTGTAAACGGTACTTTAATTTTTACAGT AGCACTCGCAGAGCTTATTTACGTGCAAATAAAAGCTC TATCTACTAGGATATTAGACTAGTATTAATAAAACACA ACATTTTATTAACAAAGTAATTT
**pME_Cp_0_3_025_rpl23_5'UTR_Cr**	5'UTR	AGTTCCCTTTTATTTTAAATAGAAAAAAAAACTTATTAA CGTCTTTTGGAGGATTACACATACTGTAAAAACGCCAA TTGTAGTCAAAATCTAGATTCTGGTTAGGCAATAGAGG ATATTTTCTTTTCCCTGTGGAATCTATAACTATAGCTGCT ATACAGACCCGAAAGAAAGCAATATATACTGGTCAGG AACTAAACTTCTCTGTTCCTAAGAAGTTTACATACCACA AAAAACAACGGTATTACTTGGTTTTAAGTTGTATTTTAA AATAAATGTTTGGTTGATATATAAAAAAAAATTTCACTT TTCTT
**pME_Cp_0_3_026_rpl16_5'UTR_Cr**	5'UTR	GATTAAACTAATTTTACCATAAGTATTCTGTTTTAATAA ATTTATGCCCATGAACGCTGCCAAAGGAGGCAGTGGCT CGCCACTGCCCCCTTACGGGTACATAAATGTCCTAACTT GATATTTATTTACCTGTAAGGGTTAGCCTATAGGCGAG GTAAATAAATTTAAGTCAGCCATAGCTATTCTAGAGTA TAACGTGTACGTATCCTTACGGGTACGTACACGTTATA CTCAGTTAGCAGGGACTTGTTAGCCTATAAGCGAGATA AGTACACTTGGCCAACGGTTTATATTAATATACTCCAGC AGAAGAACAATTAAAGTAAAATCTAAAAATATCTATCT TTTTGCTGAAGAATTGCGGAAAAATCGATAGTATTGTT CATTGTATAAAGTGTACGTACCCGTTAAGGGTACGTAC ACTTTAATGCAAGATAAACAAAAATCAATACATATTACT AGTTACTAGTATAAAGTACAATTGATTTCTGTGTATTTG TAGCTTTTAAATTAAATTTTTAATTAACTGTTACATAAA AATTTAAAATTATAAATAAAAAC

**pME_Cp_0_3_027_rpoB1_5'UTR_Cr**	5'UTR	TTTCACGCACAAGGTGGAAATTGATTGATTAGCTTTAAAATTCAAATTAAAAAAATGGCATAATGGAGAAAATACCTTTCTTTAAAACTAACCTAACAATTAGGTAATTGAACCAGGGTAAGCTTTTTTAGCTAAATCCCTTCAGAAGCTAACATGCTAACTTTTCCCATAAATTACATAACTTTTTGCTGATGGTTTGAGCTAATAGGGCCGTATTCAAGTATAATATTATATTGCCATCGGCTTTTGGGTATAAAATATTACCTTATGCTACCATCCAGCCTTAAAGGGAAGTGTATTTAGCTGCTTTATAGTATATAAATTTAAGTTAACTTACAAGTGAGTTAACATAAGGCCCAGTTGTTTAGCAAACTGCCTCCTTCGGAGTATTAAAATAGGCAGTGGCAATACCACTGCCACTGGCGTCCTAAGTATATAAATATTAAGTGGCGTGCATGTGTCTTAGTTTCCAAGAGTTTACATCCTCTTAAGTTTACTTGCCCGAAGGGGAAGAATGCAGGGTTAGACAAAGAAACATGACTCACTGTCTAT
**pME_Cp_0_3_030_atpH_5'UTR_Cr**	5'UTR	CAACCAAAGAATATAATATTCTTTGGTTGTTATCGATTTTATTGATTCATTTAGGAGGAAATA
**pME_Cp_0_3_031_psbD_5'UTR_Cr**	5'UTR	AATACACAATGATTAAAATTAAATAATAATAAATTTAACGTAACGATGAGTTGTTTTTTTATTTTGGAGATACACGCA
**pME_Cp_0_3_032_rbcL_5'UTR_Nt**	5'UTR	ATGTATTTGGCAAATCAAATACCATGGTCTAATAATCAAACATTCTGATTAGTTGATAATATTAGTATTAGTTGGAAATTTTGTGAAAGATTCCTGTGAAAAGTTTCATTAACACGGAATTCGTGTCGAGTAGACCTTGTTGTTGTGAGAATTCTTAATTCATGAGTTGTAGGGAGGGATTT
**pME_Cp_0_3_033_Synthetic_5'UTR_Sy**	5'UTR	AAATTCGATAGAGATGAAATTGGAGCTCTAGAGAATTCAGTTGTAGGGAGGGATCC
**pME_Cp_0_3_039_atpH_5'UTR_Nt**	5'UTR	GATATTGACTAGTGCTAGTTATATATGAAATGAACTAAAGATATATTTTACTACTCTAGGGGATTCTAATAGACTAGAAGTCCTTCCGGCCCCTTGTGACTGTGAATTGAATGAATAAACGGATGAAATCAAGAAATAATTCAACTAACAGTTCGAACCAAGAAATGGAAGAACGAAAGTCGTATGGGTTCACAAAGACTCTGTGGCTAAAAAGTAAAAAAGATATATCGAAGTAGTTCTGATGATTCAATAATCTTATTACTTCAATCCGAAGTTCTTAGTTACTTCGACTGGATGAGTCCTAGCGAGGGAATAATTAAGTCATAACTCATTGGTTGATTGTATCATTAACCATTTCTTTTTTTTGGTACGAGGAACTTATC
**pME_Cp_0_3_061_gene103longer_5'UTR_T7**	5'UTR	GGCAGACCACAACGGTTTCCCACTAGAAATAATTTTGTTTAACTTTAAGAAGGAGATATACAT
**pME_Cp_0_3_064_psbA_5'UTR_Nt**	5'UTR	AATAAAAAGCCTTCCATTTTCTATTTTGATTTGTAGAAAACTAGTGTGCTTGGGAGTCCCTGATGATTAAATAAACCAAGATTTTACC
**pME_Cp_0_3_065_psbC_5'UTR_Nt**	5'UTR	GTTATTTGTACCAGTAACCGGTTTATGGATGAGTGCTCTTGGAGTAGTCGGTCTAGCCCTGAACCTACGTGCCTATGACTTCGTTTCTCAGGAAATTCGCGCAGCGGAAGATCCTGAATTTGAGACTTTCTACACCAAAAATATTCTCTTAAACGAAGGTATTCGCGCTTGGATGGCGGCTCAAGATCAGCCTCATGAAAACCTTATATTCCCTGAGGAGGTTCTACCAC
**pME_Cp_0_3_091_Synthetic_5'UTR_1**	5'UTR	GTTATTATAAGGAGAAATCC
**pME_Cp_0_3_091_Synthetic_5'UTR_2**	5'UTR	ATATGATGTAGATTCATTTAGGAGGAAATA
**pME_Cp_0_3_091_Synthetic_5'UTR_3**	5'UTR	ATATGATGTAATCAAATAAAACGAAAGGCTCAGTCGAAAGACTGGGCCTTTCGTTTTATGATTCATTTAGGAGGAAATA
**pME_Cp_0_3_091_Synthetic_5'UTR_4**	5'UTR	ATATGATGTAATCAAATAAAACGAAAGGCTCAGTCGAAAGACTGGGCCTTTCGTTTTATGTTATTATAAGGAGAAATCC

**pME_Cp_0_03_092_RBS_dummy**	5'UTR	TGTCAGGATACCCGA
**pME_Cp_0_3_093_RBS_Placeholder**	5'UTR	GAAAGTGAAACGTGATTTCATGCGTCATTTTGAACATT<br>TTGTAAATCTTATTTAATAATGTGTGCGGCAATTCACAT<br>TTAATTTATGAATGTTTTCTTAACATCGCGGCAACTCAA<br>GAAACGGCAGGTTCGGATCTTAGCTACTAGAGAAAGA<br>GGAGAAATACTAGATGCGTAAAGGCGAAGAGCTGTTC<br>ACTGGTGTCGTCCCTATTCTGGTGGAACTGGATGGTGA<br>TGTCAACGGTCATAAGTTTTCCGTGCGTGGCGAGGGTG<br>AAGGTGACGCAACTAATGGTAAACTGACGCTGAAGTT<br>CATCTGTACTACTGGTAAACTGCCGGTTCCTTGGCCGA<br>CTCTGGTAACGACGCTGACTTATGGTGTTCAGTGCTTT<br>GCTCGTTATCCGGACCATATGAAGCAGCATGACTTCTT<br>CAAGTCCGCCATGCCGGAAGGCTATGTGCAGGAACGC<br>ACGATTTCCTTTAAGGATGACGGCACGTACAAAACGCG<br>TGCGGAAGTGAAATTTGAAGGCGATACCCTGGTAAAC<br>CGCATTGAGCTGAAAGGCATTGACTTTAAAGAGGACG<br>GCAATATCCTGGGCCATAAGCTGGAATACAATTTTAAC<br>AGCCACAATGTTTACATCACCGCCGATAAACAAAAAAA<br>TGGCATTAAAGCGAATTTTAAAATTCGCCACAACGTGG<br>AGGATGGCAGCGTGCAGCTGGCTGATCACTACCAGCA<br>AAACACTCCAATCGGTGATGGTCCTGTTCTGCTGCCAG<br>ACAATCACTATCTGAGCACGCAAAGCGTTCTGTCTAAA<br>GATCCGAACGAGAAACGCGATCATATGGTTCTGCTGG<br>AGTTCGTAACCGCAGCGGGCATCACGCATGGTATGGA<br>TGAACTGTACAAATGACCAGGCATCAAATAAAACGAAA<br>GGCTCAGTCGAAAGACTGGGCCTTTCGTTTTATCTGTT<br>GTTTGTCGGTGAACGCTCTCTACTAGAGTCACACTGGC<br>TCACCTTCGGGTGGGCCTTTCTGCGTTTATA
**pME_Cp_0_3IEE_001_atpE-ycf12_IEE_Cr**	IEE	TTTCAACTTATCAACAAATCCCTAATAAGAAAGTAGTAT<br>ATGTTCCTATTAGAGGGTTTGTAAAATTATCTAGAGCTA<br>TTCAAGTCTGATAGAGTTGAACAGTATACTCTTTGGGT<br>AAATACATAGATCTAACATTATTTAAAGTTTATGTTCTT<br>GTTACTTTTTGATTTTGTATATAAAAAAAAATTTTTCAT
**pME_Cp_0_3IEE_003_chlL-rpl36_IEE_Cr**	IEE	TAAGAATAAAGCAGCTTTAAATACTTTCCTGTTTATAAT<br>TTAGGAAATTAAATGGATATTTGTTGAAACTAATCCCC<br>AGTTGGATACCCATTGGTAGTTAATTGCCACTGCCTGCT<br>TCACCTTACAAAATGTATGGACACAAAACGGCTAATAA<br>ATACAGACTCCCGGTGGCATTTGTTGGCTGCTTCGCCC<br>TGAAAGGAGAAAGTGATTTCTTTCCTTATTAGCTAATCT<br>ATTCTTTTTCCTGTTTTGGTAAATAATAGCGTCCTCATAT<br>CCATATCTATAACAAAAAGTTAAATGTTTTAATTTTAAA<br>AGTCTTTTTTACATTAATAAACACTTTTAATTGATGGGA<br>CATCTTTAGTTTTTAAAATAAATAAAGATGCACCCTCAA<br>ACTGTCAGTTCATTAACCCAAATTATTTAATTTGGATGC<br>ATATAAGCGAATTGACTTCAAAACTAATAAAATTTTT
**pME_Cp_0_3IEE_004_petG-rps3_IEE_Cr**	IEE	TATTTATAAACTAAACTACCTCCAATGAAAGAGGTAGT<br>TTATAACCCAATCAAAATGTTTTACCTTTTCTTCTTCTAT<br>GGACGGAATACAAATAACAATTGCATTGTTACTCTCGA<br>CTGGTTTCTAGCTTTACTGTCAGGTGTTTATATTATGCT<br>ACTGTACCTTTTCCCAAGCAAGAATATACTTAAGGTATA<br>GAAATATAAACAAGCATCCCGAAGAAAAAAGGGGTTA<br>TTTTCTATGTTTATTACACTAAAGACATTTGATTTGGTTT<br>AATCTATATAATTGAAAATAGCTTTATTCTTTTATTAAC<br>GTATGGGAACCTTTTACTGTTTAGTTTGTTTTTGCCTATT<br>TGTAAAGGTCTATTTAAATATATCCTTTTTGCTTTGCCG<br>CAACTAAAAAGTTTACTTAGATACTTTCGTAGCTCCATT<br>TACTTGCCGGGGATTTGTTAGCTCTTTTCAAAGAAATG<br>ATTTTTCACGTTGGAAAAATCAACTTTGTATTTAGTACT<br>CTACTAACAAATACTATTTAATTCCTAGTAATGGAGTAG<br>TATTGGATGGTTAATTCTTATTTTAAACAGAGTAAGCAT<br>TACTTACTTGTAATTTTAATTATAATTAAATTTATTTATA

		AACAGGTTCTAAAGAAGCTATAACACACATTGTATTTT<br>ATTTTTTTTAACGTTGTACAAATGTTTAGAATAAAAATA<br>CGTGTTTTGCACCTTGAAATGGCTCCAGTTTTCTAAACT<br>AATGTTTCAAAGGAGATGTGTTCAACATCAATTAGCTT<br>ACTTTAATTTCATTTAAAAAATTTGT
**pME_Cp_0_3IEE_005_psaC-petL_IEE_Cr**	IEE	TTTTTTAATTCACTTGGCGTTTAAAAAACCTTTAAAAAA<br>GGGAGTATCTTGGTGAGATCCTCCATAAATTTTAAATT<br>AGGTAGCCTCTTCCTTTGGGTACATTTATTAATATAAAT<br>GTGCCCAAAGTTCTTAACAAGAAAATATAAATTCCCAT<br>AAACTTTTGGCTTATTTAATTGGAACGTTTACTTTTAAA<br>AACTATATAAAAATTAAACAACTTACAAAGTTGGTTTA<br>AAGTATCTATTTAATGTTTTTCAATCATTTTAATAAATAA<br>ATGACATGCACAAGAACCTTATTAGTTTTAAATCGCAT<br>GTTAGCTTTAAAAGCTAACTTTGTTTTGCACTCTGTGAA<br>ATCTTATTTGAATGTGTCAGGGGTTAACAAGACTCTTCC<br>AGCCAAAGGCAGTGCCGGTGTAAAAAACGGCTTTATTC<br>TATCCCATTTAGGATGCCAATGACAGTTAGAAAGCCTT<br>GATATTCCTAAAGCAGTCAAGCATCACTATTAAATTGTT<br>CACAGTCCGCATATTTTATTTGATCTTTTTGAGAAGATA<br>AAAATTACCTTCCGCTTTTGCAAAAATTTAGCCGAAGA<br>GTTTGATTTAAACTAATTCAGTTAAAAATGATTTTTGAT<br>TTTAATTATATCCATATTTTT
**pME_Cp_0_3IEE_007_psbB-psbT_IEE_Cr**	IEE	GTTTTTCTTTTCTGGCAGTTGGCAGAGAATGTGTTTGTC<br>TCCTTACCGAGTATAAAACCACTCTGCAAGTACAACTG<br>CCACTGTGGTCGCCTTACGTGGTTTAGTTACACGTAAG<br>GCGACGTCCTTTAGGTATTTAAAAGTTTTACTTACAGAA<br>CTTATTTATACATGGAGGGCTCCACAGCAGCAAAGTTG<br>TATAAGTTTAAAAAAGCACTCTCTTTTTGGGACTGCTTA<br>GCAAATTACCTAATTTTACTTTTTAAACCTTTAACACTTT<br>ACTATGATTCTTTTTATAGCCATACACTTGAAATTCTTCA<br>AGTATTTTAAAACATTTTTAAAAATGTTTTAGTGCAATG<br>TTCATTTTAAGGTAATTTCAATAGATACTTTTGCTTTGTA<br>GCTAAAGTATGCATTTATAATTAAACTTAATTCAAAAAA<br>ATCAAAATACATTTTACTTTTT
**pME_Cp_0_3IEE_008_psbE-rps9_IEE_Cr**	IEE	TTAATCTAAAAACTTTTTTTACTTAGAAAATTTTTTCTAA<br>GTAAAAAAAGTACCTTTTCAAACCATCTATATAAATCTT<br>GGCAAAGGCATTTAATTTGGGTCTACCACACATAATGA<br>TTAAAAAAAAACGTAAATCCTAAAGAAAAAGTCCATTGA<br>TGTCAGTTAGCATAACTCACGTACAAGAAATAATAATA<br>TGATAGTAATATAAGATATATATAGGAGTTTCCATAAA<br>GATAAAGAAAGCTTTTTAGTGATTTTAAAAAAATATTG<br>TTCATAAATAAACATGAGCAACTGGCACTAGTCTAGAT<br>GACTAATAGGGTAAAAGATTAGTTCTAATCATTGTGCT<br>TTTAGCGCACTTTCTAGACTTTGTGATATGGTAGGTTTT<br>ACAATGAATATCGTCCATTTTCCTCTTAACAGAATCATA<br>AATCTCAACAGGAAACTAAATTCCCTTGTGTTTATTTGC<br>AAAATATTTTTTATAGTTTATTTTGATTGCTTGTGAGTTT<br>AATTTCAATTTAAATGGTAACATTTATATTTTTGAAAGA<br>CGATTTTTGATAATTTAACGCATGTGCGAATTAAAAATT<br>TGTGACAAGCATTTTCCCGATATGTACAATACATTTGAA<br>ACTCCTATTAAAATTTATTGGCCGAAATCAGTCATATAA<br>CCTAAAAAAATAGGTTACTTACCAGAAGGAGAGCATAAA

		AAATTTTATATGGGAAGTGTAATATGGCTTTAGCAAAAGATGAAAAAATCTCTCATTGTTTTTTCAGTTGCTTTTAGGTATTATGTTGTATTTTAAAAGGGGTGTCGGATGTCTTTTAAAAAATATAGAAATAAAGCAAATTAAAAATTTATTTTTGT
**pME_Cp_0_3IEE_009_psbF-psbL_IEE_Cr**	IEE	GTATTCGTCCCTAAAAGAACGTTAGTACGCTGTAGCGAAATCCTTTCTGGATATTTGTATATTCCAAAGGGCGGCTGTGGCTGTAGATATTTACATACTCCATGCTTTGCCTAGATATTTATATTAGGATTTTAATACTCCCATGCTTTGCATAGATATTTATATTCCCGTAAGGGGATGCCGGAGGCATTGAAGGAGGCAGTTGGCAGGATATTTATATACTCCGAAGGGACTTCTTAGCCGATAGGCGAGGCAACTGCCTCCTTCGGAGTATATAAATATCCACTGGCATCCCGTAAGGGGATGCCGAAGGCAGGCTATATTAATATCAACTGACGTCCTAATTTATGGACTTTTTTAGAACTGCCTGCAGCTTTTGGGCAAGAAAATTGATTACAATAGCCTAACCTTTGAGTCCATCCACAGTAAGGATAGCTTAAAATCCCTAAAGGGATCGTTATTTTACCTAGAACACACTCAAAAGGAAAAGGATATTATGACTTTAATAAATAACCAAAACGTTATTTTTATAGTTATAGACATTAGTTAATGAGTAAAATAATTTATAGGCTATCTTTTTTAATGTAAACTCATTAAATAAGTTAGACCG
**pME_Cp_0_3IEE_010_psbJ-atpI_IEE_Cr**	IEE	TCAAATTTAAATTTTTTGTTTAGGCAGTTGGCTATGCCTCAACTCACTTACGGGATTTACATACTCCGTAAAAAAGATGTTAGTGGCAAGTTACTGCCTATTCGAAAAAATTTGAAGCCCTTATCAAGCTTCCACATAGCGTTTTAGATGGTTTGTTTTCCAACAATCCATCTTTTTATATATTTGTTATGAAAATAGCAGTTTATTTAGACTTTTGCATCAATCCATAGGATTGTATATACCAGATTTTAAACAAATATGTTTTACAATTTTTTCTTGTAACATAACCTTACTTACGTGTCCTCCTTAGGGCTAACAATAATACAAAGCATACCTAATACGGTTAAAGCTTGGCAAACTGTGAGGCTGTTAAACAGCATAAGTTATCCAAAAAAGAGACACTCAAATATGGAGTTCCTAATACTGCTAAAGCTAGAATTGTTCTTGCTTGCAAATATACATCATTTTTGGGAATGCTATAATCTTACCACCTTTTTAAAGGACTCACCTGTTTAATACTGTAATCGCTTTTAGGTAGAGAACTATCTTTGTATTCACCATATTCTTCTGTCACATGCACAGCAATATAATACGTAAGTACAATTGTTTAAAAAAATAGATAATAACTGGTCATTATTTATAGTGGTTATTATTAAAAATATTAATATCTTTCCAATAATTGGTAAGAATTTTATTACATTTTT

**pME_Cp_0_3IEE_013_rpl2-rps19_IEE_Cr**	IEE	CTAATTAAAGATAGGTCAATTAAAACAAGTGGCCTCTT<br>TTGGCTATTTATTTACCCCCTTCCCCTTACGGGACAATA<br>AATAAATTTGTTGGCAGGCAACTGCCTCCTTCCCCTTCG<br>GGCAAGTAAACTTAGAATAAAATTTATTTGCTGCGCTA<br>GCAGGTTTACATACTCCTAAGTTTACTTGCCCGAAGGG<br>GAAGGAGGACGTCCCCTTACGGGAATATAAATATTAGT<br>GGCAGTGGTACAATAAATAAATAGTATATAAATATCGG<br>CAGTTGGCAGGCAACTGCCACTGACGTCCACTAAAATT<br>TATTCTTTCTCGGGGACAATAAATAAATTTGTCCTGTAA<br>AGGGACGTAAAATAGCAGTAAGCATAAGTATGGCCAC<br>TTGCTTAAATTTTACAATATTAAAAAAATTCTAGAAATA<br>ATAAAGTTTTGGTTGATAAATTTTTAACGTTAATTGTTT<br>GTTTAAACTTTATAGATATCGGGACTTAGTAAGTCTAA<br>AGTCGCTAAAAACAACCAGTTTCAGATAAACATTTGTTT<br>CAACTGATTGGTTCGTTTTGTTTATCCTTAGAGTTTATA<br>TATCTTAACTCTATATTGGGTAAACCACTATAATGGTCA<br>TATGTTGGAAAAATTCCAATAAATTTCAATTTAATGTGG<br>AATTTAAAAAGCTCATATGTACTTAAAATAGACAATTGT<br>TAAACATGAATAGAAAATATTACCTACTTTTATTTTTAT<br>AAATACAGCTTTAGCCATTATTATAAAATTCAAAAGTCA<br>TTTTAAAAAATCAA
**pME_Cp_0_3IEE_015_rpl23-rpl2_IEE_Cr**	IEE	TGGCTGATTTAAAATAGGCAGTGGCGGTACCACTGCCA<br>CTGGCGTCCTCTCAAGAACAACAAAAGATATTTTTAAA<br>CTCTAAGTTTATTTGCCCAAAGAGGCAGAGAAACATTG<br>TACCAGAAATGACAGCTTTTGTAGTTTAGGGATATACA<br>TAGCCAAAGCTCTAGGATCTATCCAAAAATCACTGTCA<br>TGCCGCCCCTCAAGGGGAATCAGTAGACTTTACAGTGT<br>ATCCCTTTACCGGTATAGTAATCTTTTGCTTATTTTAGAA<br>GCAAGTATCATATAAATAAAATTGTAAATAAAAAATAT<br>ACTTAATTTTTTAATAAAAAGGGTTATTTTTCTAATAAT<br>AAATCTAGCTATTAAGTGGTAACCTATCTATCGGCTAA<br>ACCCTTTAATATGCAAAGGCCCGTTTAAGGTGCCAATG<br>CGTAGTTGAAAAACACCTTTCCCTTTTCCCCTGCGGGAC<br>GCCAGTAGATAAATATCGGCAGTTGGCAGGCAACTGC<br>CACTGACGTCCCGTAAGGGGAAAGGGACGTCCTGCCA<br>ACTGCTGAGGCAAATGAATTTTAGTGGCAATGTTACCG<br>CCACTGGCGTCCCGTAAGGGGAAAGGGAGGAGCTCGA<br>ACGAGGTTCGTTAGTGGTCCACTTTTAATTGTTTGACTC<br>CTCATGAGAGCTGAAAGACAGGCTGTAAATTTTATTAG<br>TACCTAATCTATAAATAGTAAAACAACTTAAAAACATAA<br>TT
**pME_Cp_0_3IEE_018_rps14-rps7_IEE_Cr**	IEE	TTTCGTTTTGATATCGTAAACTTAGTTATATGCATATCTA<br>TTAAGTAGCGATTTTCAAAGAGGCAGTTGGCAGGACG<br>TCCCCTTACGGGAATATAAATATTAGTGGCAGTGGTAC<br>CGCCACTGCCTATATTTATATACTCCGAAGGAACTTGTT<br>AGCCGATAGGCGAGGCAACAAATTTATTTATTGTATAT<br>AAATATCCACTAAAATTTATTTGCCCGAAGGGGACGTC<br>CTATTAAACCATCACATAACTAAAATTGCTTATTTGGTA<br>TGAAAGTTTGCATCTATTTTAACCATTTAGTAAAAATAA<br>TGATGCTTTTTTAAAATAAAA

**pME_Cp_0_3IEE_019_rps18-rps2_IEE_Cr**	IEE	CAAATCCAGTGTTATAAAAAACTTGCTAAGTACTTAAATCTCAGCGAAAGCTAGATATCGATTTATCTACTAGTATTGTCTTAATAGAAAAAAGTTATTAATAGAAAAGAAAAATATAAAAATCAATAGTGGATTTTGAATAGAAATTGAATGCCTTACAAACTTTTCTGCTTCAGACACATAATAAGTTAGAGATAACCCCTTTATGGCATAAAATGTCTTTGTTATGTGGCAGCCCCGAAAGGGAAGTCTACTAACTCGTTAAAGGCTGCGCTCCCAGATTAATATTCTGCGAAGGATATCGCCTGATAGGTAAATAAATTTTAACTGGCATCCCTTTACGGGATAACATTTATTGGCTGCCTCGCCTATCGGCTAACCACTTCGGGCAAGTCAACTTAGAATAACATTTATTTACTGCGCTAGCAGGTTTACATACTAGGCAGTGGTGGTACCACTGCCACTGGCGTCCTCCTTCGGAGTATGTAAACATTCAATATTTATATTAGGACGTCCCCTTCGGGTAAATAAATTTTAGTGGCAGTTGGCAAGATATTTATATACAATATTAATACTTCGCAACTGCCACTGACGTCCTTCCCCTTCGGGCAAGTAAACTTAGAATAAAATTTATTTGCTGCGTTAGCAGGTTTACATACTCCTAAGTTTACTTGCCTCCTTCGGAGTATATAAATATCCCGAAGGGGAAGGAGGACGTCCCCTTACGGGAATATAAATATTAGTGGCAGTGGTACAATAAATAAATAGTATATAAATATCCCGTAAGGGGAAGGGGACAAATTTATTTATTGTCCTAGGTACATAAATGTTTTAAGCAGTTTAAAGGCCTGACGGGCTCAATTTCT
**pME_Cp_0_3IEE_020_trnF-psaC_IEE_Cr**	IEE	GCCGGGATAGCTCAGTTGGTAGAGCAAAGCATTGAAAATGCTTGGGTCACCGGTTCAAGTCCGGTTCCTGGCAATTCCCTAATGGACCAAAAGCAGTCATTCAAATATAGGTATGAGGAGAGAAATATATGCGCCGCAAGGCGCACTGCCACTGCTTAATATAAATACCTTGTTTAAAGTCGATTCTCAATCTTCTTTTTGATATGGAGATGACATATTTAGCACAACGCAAAAATATAGCATCTTTAAAAATTAACTTAAAATTTAAAT
**pME_Cp_0_3IEE_021_ycf3-rps18_IEE_Cr**	IEE	AAAAGATGTTGACCCTTTTGACTTGAAGTTGCCAGATTGATTTTTACTCTTCTCTAAGCCATATTTTAATACTTTGTAAGGAAAATATAAATACCTATTGATATAAATTAAAACAAATTGCAGCCCCTCAGTGGCAATTTTATTTACCTAGGCCGCCAGCGGCAGTGGTACCACCACTGCCGCTGGTGTTCCTAAGAAGAAAAGGTTTAGATATTTTCCTTTGGGGCTGTACAACAGCAAGATTAATATGCTTTATGGAGAAGCTGTATTCTCAATGGTCTAAGCTGCTTCAAATTTTATAAAAAACTACTTTAGTTACTAAGTAGTATTTCGCAATTTATATTTTAAATTTTTATTTATTT
**pME_Cp_0_3IEE_022_ycf4-ycf3_IEE_Cr**	IEE	AATACTGATTTTAAAATTTTATAGAGATTAAATTAAAAACAAAACGTCTAAACAGATGTTATTAAGCTGCACCTTTGGCGCAGCTTAAAAGGCACGTTGCCTAATATTACTACAGTACAAAAATGTAATTGTTATAGTTATAAGTACTTAGTTTCAATAAATCGCTGATTTTATTTTTA
**pME_Cp_0_3IEE_023_IEE5_v2**	IEE	GACTTGTAAAAAATATGACGTCTTTTTCCTTTGGTTTATAGTATTTGGAAAAGCAAAATGTTTAGACATCTGGTTTAAAGACAGTATACTACTTGATTGCACTTTTGGCCGTATAGTAGATCATAGGTTAAAAGGCTATTTGTACATAAGTAGACTTTTACATTTTAATTTTTTTGTAAGTTTGAATACATTTAGTTAATTAAGTTTTAGATTTTTAAATTAAAAAGCTCCCTTGTATGAGGAAGTAGGTTGTCCCACAGGGATTGCTTAAAATCTAACAAAAATACATTTCCGTTAAATTAAGATGGGTGTAGCATCTCGCTGCTATACTTCACCAAAAGATCTTACTTCCACATACCCTGTTTGTGACCAAGTAAGTGTTTACTTTTAGATTTAACGTGTTTATAACATTTAAGTAATTAT

**pME_Cp_0_3IEE_023_tscA-chlN_IEE_Cr**	IEE	GACTTGTAAAAATATGACGTCTTTTTCCTTTGGTTTATA<br>GTATTTGGAAAAGCAAAATGTTTAGACATCTGGTTTAA<br>AGACAGTATACTACTTGATTGCACTTTTGGCCGTATAGT<br>AGATCATAGGTTAAAAGGCTATTTGTACATAAGTAGAC<br>TTTTACATTTTAATTTTTTTGTAAGTTTGAATACATTTAG<br>TTAATTAAGTTTTAGATTTTTAAATTAAAAAGCTCCCTT<br>GTATGAGGAAGTAGGTTGTCCCACAGGGATTGCTTAA<br>AATCTAACAAAAATACATTTCCGTTAAATTAAGATGGG<br>TGTAGCATCTCGCTGCTATACTTCACCAAAAGATCTTAC<br>TTCCACATACCCTGTTTGTGACCAAGTAAGTGTTTACTT<br>TTAGATTTAACGTGTTTATAACATTTAAGTAATTAT
**pME_Cp_0_3b_003_strep-tag**	N-terminal Tag	ATGTGGAGCCACCCGCAGTTCGAGAAGGGAATG
**pME_Cp_0_3b_024_His-Tag**	N-terminal Tag	CACCATCACCACCATCAT
**pME_Cp_0_3b_025_Hibit-tag**	N-terminal Tag	CCATGGTTTCTGGTTGGCGTTTATTTAAGAAAATTTCTG<br>GAATG
**pME_Cp_0_3b_026_flag-tag**	N-terminal Tag	GATTATAAAGATGACGATGACAAAGGTGACTATAAGG<br>ATGATGATGATAAAGGTGACTACAAAGACGACGATGA<br>TAAA
**pME_Cp_0_3b_027_HA-tag**	N-terminal Tag	TATCCATATGATGTACCAGATTACGCAAAATACCCATAC<br>GATGTTCCTGATTATGCTGGTTATCCTTATGATGTTCCA<br>GATTATGCT
**pME_Cp_0_3b_028_myc-tag**	N-terminal Tag	GAACAAAAACTTATTTCAGAGGAAGATTTAAACGGTGA<br>GCAAAAATTAATTTCTGAGGAAGACTTAAATGGTGAAC<br>AAAAACTGATTTCGGAGGAAGATCT
**pME_Cp_0_4_001_Nanoluc**	CDS	GTTTTCACTTTAGAAGACTTCGTAGGTGACTGGCGTCA<br>AACAGCAGGTTATAATTTAGACCAAGTTTTAGAACAAG<br>GTGGTGTATCAAGTTTATTCCAAAATTTAGGTGTTTCTG<br>TTACTCCAATTCAACGTATCGTATTAAGTGGTGAAAAT<br>GGTCTTAAAATTGACATCCATGTTATTATTCCTTATGAA<br>GGTCTTTCAGGTGACCAAATGGGTCAAATTGAAAAAAT<br>TTTTAAAGTAGTTTATCCTGTAGATGACCATCACTTTAA<br>AGTAATTTTACACTATGGTACTTTAGTAATTGATGGCGT<br>TACACCTAATATGATTGACTACTTTGGTCGTCCTTATGA<br>AGGTATTGCTGTTTTTGATGGTAAAAAAATCACAGTTA<br>CAGGTACATTATGGAATGGTAATAAAATTATTGACGAA<br>CGTTTAATCAATCCTGATGGTTCATTATTATTCCGTGTT<br>ACAATTAATGGTGTTACAGGTTGGCGTCTTTGTGAACG<br>TATCCTTGCT
**pME_Cp_0_4_005_aadA_with_stop_codon**	CDS	GCAATGCGTACTCCAGAAGAACTTAGTAATCTTATTAA<br>AGATTTAATTGAACAATACACTCCAGAAGTGAAAATGT<br>CCATGGCTCGTGAAGCGGTTATCGCCGAAGTATCGACT<br>CAACTATCAGAGGTAGTTGGCGTCATCGAGCGCCATCT<br>CGAACCGACGTTGCTGGCCGTACATTTGTACGGCTCCG<br>CAGTGGATGGCGGCCTGAAGCCACACAGTGATATTGA<br>TTTGCTGGTTACGGTGACCGTAAGGCTTGATGAAACAA<br>CGCGGCGAGCTTTGATCAACGACCTTTTGGAAACTTCG<br>GCTTCCCCTGGAGAGAGCGAGATTCTCCGCGCTGTAGA<br>AGTCACCATTGTTGTGCACGACGACATCATTCCGTGGC<br>GTTATCCAGCTAAGCGCGAACTGCAATTTGGAGAATGG<br>CAGCGCAATGACATTCTTGCAGGTATCTTCGAGCCAGC<br>CACGATCGACATTGATCTGGCTATCTTGCTGACAAAAG<br>CAAGAGAACATAGCGTTGCCTTGGTAGGTCCAGCGGC<br>GGAGGAACTCTTTGATCCGGTTCCTGAACAGGATCTAT<br>TTGAGGCGCTAAATGAAACCTTAACGCTATGGAACTCG<br>CCGCCCGACTGGGCTGGCGATGAGCGAAATGTAGTGC<br>TTACGTTGTCCCGCATTTGGTACAGCGCAGTAACCGGC<br>AAAATCGCGCCGAAGGATGTCGCTGCCGACTGGGCAA<br>TGGAGCGCCTGCCGGCCCAGTATCAGCCCGTCATACTT<br>GAAGCTAGGCAGGCTTATCTTGGACAAGAAGATCGCTT

		GGCCTCGCGCGCAGATCAGTTGGAAGAATTTGTTCACT<br>ACGTGAAAGGCGAGATCACTAAGGTAGTTGGCAAATA<br>A
**pME_Cp_0_4_007_mScarlet-I**	CDS	GTTTCAAAAGGTGAAGCAGTAATTAAAGAATTTATGCG<br>TTTCAAAGTTCATATGGAAGGTTCTATGAATGGTCACG<br>AATTTGAAATTGAAGGTGAAGGTGAAGGTCGTCCATAT<br>GAAGGTACACAAACTGCTAAATTAAAAGTAACAAAAG<br>GTGGTCCATTACCTTTTAGTTGGGATATTTTATCTCCAC<br>AATTTATGTATGGTTCACGTGCATTTATTAAACACCCAG<br>CAGACATTCCAGATTACTATAAACAATCATTCCCAGAA<br>GGTTTCAAATGGGAACGTGTTATGAACTTTGAAGATGG<br>TGGTGCAGTTACTGTAACACAAGATACATCATTAGAAG<br>ATGGTACTTTAATTTATAAAGTTAAATTACGTGGTACAA<br>ATTTTCCACCTGATGGTCCTGTAATGCAAAAAAAAACA<br>ATGGGTTGGGAAGCTTCAACTGAACGTTTATATCCTGA<br>AGATGGTGTATTAAAAGGTGATATTAAAATGGCATTAC<br>GTTTAAAAGATGGTGGTCGTTATTTAGCTGACTTTAAA<br>ACAACTTATAAAGCAAAAAAACCTGTTCAAATGCCTGG<br>TGCTTATAACGTTGATCGTAAATTAGACATTACTTCACA<br>CAATGAAGATTATACAGTAGTTGAACAATATGAACGTA<br>GTGAAGGTCGTCATTCTACAGGTGGTATGGATGAATTA<br>TATAAA
**pME_Cp_0_4_008_tdTomato**	CDS	GTTTCAAAAGGTGAAGAAGTAATTAAAGAATTCATGCG<br>TTTTAAAGTACGTATGGAAGGTTCTATGAATGGTCATG<br>AATTTGAAATTGAAGGTGAAGGTGAAGGTCGTCCATAT<br>GAAGGTACACAAACAGCAAAATTAAAAGTTACTAAAG<br>GTGGTCCATTACCATTCGCTTGGGATATTTTAAGTCCAC<br>AATTTATGTACGGTTCAAAAGCTTATGTAAAACATCCA<br>GCAGACATTCCAGATTATAAAAAATTAAGTTTTCCAGA<br>AGGTTTTAAATGGGAACGTGTAATGAATTTCGAAGATG<br>GTGGTTTAGTTACAGTTACTCAAGACTCAAGTTTACAA<br>GATGGTACATTAATTTATAAAGTTAAAATGCGTGGTAC<br>TAATTTTCCACCTGATGGTCCAGTTATGCAAAAAAAAA<br>CTATGGGTTGGGAAGCAAGTACAGAACGTTTATATCCA<br>CGTGATGGTGTTTTAAAAGGTGAAATTCACCAAGCATT<br>AAAATTAAAAGATGGTGGTCACTATTTAGTTGAATTTA<br>AAACAATTTATATGGCAAAAAAACCTGTACAATTACCT<br>GGTTATTACTATGTTGATACTAAATTAGACATTACTTCT<br>CACAATGAAGATTACACTATTGTAGAACAATATGAACG<br>TAGTGAAGGTCGTCACCATTTATTTTTAGGTCACGGTAC<br>TGGTTCTACAGGTTCAGGTTCATCTGGTACTGCTTCAA<br>GTGAAGATAATAACATGGCAGTAATTAAAGAATTTATG<br>CGTTTTAAAGTACGTATGGAAGGTTCTATGAATGGTCA<br>TGAATTCGAAATTGAAGGTGAAGGTGAAGGTCGTCCA<br>TATGAAGGTACTCAAACTGCAAAATTAAAAGTAACTAA<br>AGGTGGTCCATTACCTTTCGCATGGGATATTTTATCACC<br>ACAATTCATGTATGGTTCTAAAGCTTATGTTAAACATCC<br>AGCTGATATTCCTGATTATAAAAAATTAAGTTTTCCAGA<br>AGGTTTCAAATGGGAACGTGTAATGAATTTTGAAGATG<br>GTGGTTTAGTTACAGTAACTCAAGATTCATCTTTACAAG

		ATGGTACTTTAATTTATAAAGTTAAAATGCGTGGTACA<br>AACTTCCCACCTGATGGTCCTGTTATGCAAAAAAAAAC<br>AATGGGTTGGGAAGCTTCTACTGAACGTTTATACCCAC<br>GTGACGGTGTATTAAAAGGTGAAATTCATCAAGCTTTA<br>AAATTAAAAGATGGTGGTCACTATTTAGTTGAATTTAA<br>AACAATTTACATGGCTAAAAAACCTGTTCAATTACCTG<br>GTTATTACTATGTAGATACAAAATTAGACATTACATCAC<br>ATAATGAAGATTACACAATTGTTGAACAATACGAACGT<br>TCAGAAGGTCGTCACCATTTATTCTTATATGGTATGGAC<br>GAATTATACAAA
**pME_Cp_0_4_009_mVenus**	CDS	GTTTCAAAAGGTGAAGAATTATTCACAGGTGTTGTACC<br>AATTTTAGTTGAATTAGATGGTGATGTTAATGGTCACA<br>AATTCTCAGTATCTGGTGAAGGTGAAGGTGATGCAACA<br>TACGGTAAATTAACTTTAAAATTAATTTGTACTACAGGT<br>AAATTACCAGTTCCTTGGCCAACATTAGTTACTACATTA<br>GGTTACGGTTTACAATGTTTTGCACGTTACCCAGACCAT<br>ATGAAACAACATGACTTTTTCAAAAGTGCTATGCCAGA<br>AGGTTATGTACAAGAACGTACTATTTTTTTCAAAGACG<br>ATGGTAATTATAAAACACGTGCAGAAGTAAAATTCGAA<br>GGTGATACATTAGTTAATCGTATTGAATTAAAAGGTAT<br>TGATTTTAAAGAAGATGGTAATATTTTAGGTCACAAAT<br>TAGAATATAATTACAACTCACATAATGTTTATATTACAG<br>CAGATAAACAAAAAAACGGTATTAAAGCTAATTTTAAA<br>ATTCGTCACAATATTGAAGATGGTGGTGTTCAATTAGC<br>AGATCATTATCAACAAAATACTCCAATTGGTGATGGTC<br>CTGTATTATTACCTGATAACCATTACTTATCATACCAAA<br>GTAAATTATCAAAAGACCCTAATGAAAAACGTGATCAT<br>ATGGTATTATTAGAATTTGTTACAGCAGCTGGTATTACA<br>TTAGGTATGGATGAATTATATAAA
**pME_Cp_0_4_010_mCerulean3**	CDS	ATGGTAAGTAAAGGTGAAGAATTATTTACTGGTGTAGT<br>TCCAATTTTAGTTGAATTAGACGGTGACGTTAATGGTC<br>ATAAATTTTCTGTTTCAGGTGAAGGTGAAGGTGATGCT<br>ACTTACGGTAAATTAACTTTAAAATTTATTTGTACAACT<br>GGTAAATTACCAGTACCTTGGCCTACATTAGTAACTAC<br>ATTAACATGGGGTGTTCAATGTTTTGCACGTTATCCAG<br>ATCACATGAAACAACATGATTTCTTTAAATCAGCTATGC<br>CAGAAGGTTATGTACAAGAACGTACAATTTTCTTTAAA<br>GATGACGGTAACTATAAAACACGTGCAGAAGTTAAATT<br>TGAAGGTGATACATTAGTTAATCGTATTGAATTAAAAG<br>GTATTGATTTCAAAGAAGATGGTAATATTTTAGGTCAT<br>AAATTAGAATACAATGCTATTAGTGATAATGTTTATATT<br>ACTGCTGATAAACAAAAAAACGGTATTAAAGCTAACTT<br>CAAAATTCGTCACAATATTGAAGATGGTTCAGTTCAATT<br>AGCTGATCATTATCAACAAAACACACCAATTGGTGATG<br>GTCCAGTTTTATTACCTGATAATCACTACTTAAGTACAC<br>AATCTAAATTATCTAAAGACCCAAATGAAAAACGTGAC

		CACATGGTTTTATTAGAATTTGTTACAGCAGCAGGTATT<br>ACATTAGGTATGGATGAATTATACAAA
**pME_Cp_0_4_022_aac6-aph2-Tobramycin-marker**	CDS	GAAAACATTGTTGAAAACGAAATTTGTATTCGTACATT<br>AATTGACGATGACTTCCCTTTAATGTTAAAATGGTTAAC<br>TGATGAACGTGTTTTAGAATTCTATGGTGGTCGTGACA<br>AAAAATACACTTTAGAATCTCTTAAAAAACACTATACTG<br>AACCATGGGAAGACGAAGTTTTCCGTGTAATTATTGAA<br>TATAACAACGTTCCAATTGGTTATGGTCAAATTTACAAA<br>ATGTATGACGAATTATACACTGACTACCATTATCCTAAA<br>ACAGACGAAATTGTTTACGGTATGGATCAATTCATTGG<br>TGAACCTAACTACTGGAGTAAAGGTATTGGTACACGTT<br>ACATTAAATTAATTTTCGAATTCCTTAAAAAAGAACGTA<br>ACGCTAACGCAGTTATTTTAGATCCTCATAAAAACAACC<br>CTCGTGCTATTCGTGCATATCAAAAAAGTGGTTTCCGTA<br>TTATTGAAGATTTACCAGAACATGAATTACACGAAGGT<br>AAAAAAGAAGACTGTTACTTAATGGAATACCGTTATGA<br>TGACAACGCTACTAACGTAAAAGCTATGAAATACTTAA<br>TTGAACACTACTTTGATAACTTCAAAGTTGACTCAATTG<br>AAATTATTGGTTCTGGTTATGATTCTGTTGCATACTTAG<br>TAAACAACGAATACATTTTTAAAACTAAATTCTCTACTA<br>ACAAAAAGAAAGGTTACGCAAAAGAAAAAGCTATTTA<br>CAACTTCTTAAACACTAACTTAGAAACAAACGTTAAAAT<br>TCCTAACATTGAATACTCTTACATTTCTGACGAATTATC<br>AATTTTAGGTTACAAAGAAATTAAAGGTACTTTCTTAAC<br>ACCTGAAATTTACTCTACAATGTCTGAAGAAGAACAAA<br>ACTTATTAAAACGTGATATTGCAAGTTTCTTACGTCAAA<br>TGCACGGTTTAGACTACACTGATATTTCTGAATGTACTA<br>TTGATAACAAACAAAACGTATTAGAAGAATATATTTTA<br>TTACGTGAAACTATTTACAACGACTTAACTGATATTGAA<br>AAAGATTACATTGAATCTTTCATGGAACGTTTAAACGC<br>AACAACTGTATTCGAAGGTAAAAAATGTTTATGTCACA<br>ACGACTTCAGTTGTAACCATTTATTATTAGACGGTAACA<br>ACCGTTTAACAGGTATTATTGATTTTGGTGACTCAGGTA<br>TTATTGATGAATATTGTGACTTTATTTACTTATTAGAAG<br>ACTCTGAAGAAGAAATTGGTACTAACTTTGGTGAAGAC<br>ATTTTACGTATGTATGGTAACATTGATATTGAAAAAGC<br>AAAAGAATACCAAGACATTGTTGAAGAATACTATCCAA<br>TTGAAACAATTGTATACGGTATTAAAAACATTAAACAA<br>GAATTCATTGAAAACGGTCGTAAAGAAATTTACAAACG<br>TACATACAAAGAC

**pME_Cp_0_4_023_ptxD-marker**	CDS	TTACCAAAATTAGTAATTACTCACCGTGTTCACGACGAA<br>ATTTTACAATTATTAGCTCCACACTGTGAATTAATGACA<br>AACCAAACTGATTCTACTTTAACACGTGAAGAAATTTTA<br>CGTCGTTGTCGTGACGCACAAGCTATGATGGCTTTCAT<br>GCCAGATCGTGTTGATGCTGATTTCTTACAAGCTTGTCC<br>AGAATTACGTGTAGTTGGTTGTGCTTTAAAAGGTTTCG<br>ATAACTTCGATGTTGATGCTTGTACTGCTCGTGGTGTAT<br>GGTTAACATTCGTACCAGATTTATTAACAGTACCAACA<br>GCTGAATTAGCTATTGGTTTAGCTGTAGGTTTAGGTCG<br>TCACTTACGTGCTGCTGATGCTTTCGTACGTTCAGGTGA<br>ATTCCAAGGTTGGCAACCACAATTCTATGGTACTGGTTT<br>AGATAACGCTACTGTAGGTATTTTAGGTATGGGTGCTA<br>TTGGTTTAGCTATGGCTGACCGTTTACAAGGTTGGGGT<br>GCTACTTTACAATATCACGAAGCTAAAGCTTTAGATACT<br>CAAACAGAACAACGTTTAGGTTTACGTCAAGTAGCTTG<br>TTCAGAATTATTTGCTTCTTCTGATTTCATTTTATTAGCT<br>TTACCATTAAACGCTGATACTCAACACTTAGTAAACGCT<br>GAATTATTAGCTTTAGTTCGTCCAGGTGCTTTATTAGTT<br>AACCCATGTCGTGGTTCAGTAGTAGATGAAGCTGCTGT<br>ATTAGCTGCTTTAGAACGTGGTCAATTAGGTGGTTACG<br>CTGCTGATGTTTTTGAAATGGAAGATTGGGCTCGTGCT<br>GACCGTCCACGTTTAATTGACCCTGCTTTATTAGCTCAC<br>CCAAACACTTTATTTACACCACACATTGGTTCAGCTGTT<br>CGTGCTGTTCGTTTAGAAATTGAACGTTGTGCTGCTCA<br>AAACATTATTCAAGTATTAGCTGGTGCTCGTCCAATTAA<br>CGCTGCTAACCGTTTACCAAAAGCTGAACCTGCTGCTT<br>GTGGTTCTTACCCATACGATGTTCCAGATTACGCT
**pME_Cp_0_4_026_Arg9-marker**	CDS	GCACAATTACAACAACATCAAGGTCGTGCAGCTGCAGC<br>TCAACCTAAACGTGCTCGTCAATTAGCTGTTGCTCCTCG<br>TGCTGCATTAACATTCAACCCTGCTTCATTATTCAAAGG<br>TTTAACTTCAAGTAAACCAACACCTAAAGATGCAGCTTT<br>CACTAAACAAGTTATTGCTGATGAATCTAAATATGTTTT<br>ACAAACATATGGTCGTGCTCCAGTTGTAATTTCTCACG<br>GTAAAGGTGCTAAAATGTGGGACGTAGAAGGTAAAGA<br>ATATATTGATATGGCTGCAGGTATTGCAGTTAATGCAT<br>TAGGTCACTCAGATTCACAATGGTATGCAGCTTTAGTA<br>GAACAAGCTGAAAAATTAGCTCATACATCAAATTTATA<br>CCACACACAACCACAAGTTGAATTAGCAAAACGTTTAG<br>TAGAAAATAGTTTCGCAGACAAAGCTTTCTTTTGTAATA<br>CAGGTACAGAAGCAAATGAAGGTGCTATTAAATTCGCT<br>CGTAAATGGGCTCGTGTACGTGCTGGTATTGACCCATA<br>TGATGGTGGTGCAGTAGCTCCATATGAATTAGTATCAT<br>TTACATCATGTTTTCACGGTCGTACAATGGGTGCTTTAG<br>CATTAACATATAAAGAACAATATAAAACACCATTCTATC<br>CAATGATGCCTGGTCATCAATTAGCAGAATATAATAAC<br>TTAGAAAGTGCAGCTGCAGTTATTAAAAAAGGTAAAAC<br>AGCAGCTGTTTTTGTAGAACCTGTTCAAGGTGAAGGTG<br>GTGTTACACCATCTACACAAGCATTTTTAAAAGGTTTAC<br>GTCAATTATGTGATGAAGCTGGTGCTTTATTAGTATTTG<br>ATGAAGTACAATGTGGTTTAGGTCGTACAGGTAAATTA<br>TGGGGTCACCAATTATTCGGTGTAGAACCAGACATGAT<br>GACATTAGCTAAACCATTAGCTGGTGGTTTACCTATTG<br>GTACAGTTTTATTAAAACAACACGTTGCTGATGTAATG<br>AAACCTGGTGATCACGGTAGTACATTTGCAGGTAATCC<br>TTTAGTATGTCATGTAGCTTGTTCAGTATTTGATATTAT<br>TAACTCACCAGCTTTTTTAGCTGCAGTTGAAGCTAAAG<br>GTGAACGTTTACGTGCTGGTTTACGTCGTACAATGGCT<br>GGTAACCCTCATGTACAAGAAGTTCGTGGTGTTGGTTT<br>ATTAGTTGGTGTACAATTAGATATGATGGCAGGTCCAG<br>TAGTTGATGCAGCTCGTGATATGGGTGTTATGGCAATT<br>ACTGCAGGTAAAGGTGATGTTATTCGTTTAGTTCCACCT

		TTAGTAGTTACAGACGCTGAAATTGATACTGCTTGTGA<br>AGTTTTAGCTGCAGCTTTAAATAAAGTTGCTCCAAAA
**pME_Cp_0_4_033_Firefly-luciferase_v2.0**	CDS	GAAGATGCTAAAAATATTAAAAAAGGTCCAGCTCCATT<br>TTATCCATTAGAAGATGGTACAGCTGGTGAACAATTAC<br>ATAAAGCTATGAAACGTTATGCTTTAGTTCCAGGTACA<br>ATTGCTTTTACAGATGCTCATATTGAAGTTGATATTACA<br>TATGCTGAATATTTTGAAATGTCAGTTCGTTTAGCTGAA<br>GCTATGAAACGTTATGGTTTAAATACAAATCATCGTATT<br>GTTGTTTGTTCAGAAAATTCATTACAATTTTTTATGCCA<br>GTTTTAGGTGCTTTATTTATTGGTGTTGCTGTTGCTCCA<br>GCTAATGATATTTATAATGAACGTGAATTATTAAATTCA<br>ATGGGTATTTCACAACCAACAGTTGTTTTTGTTTCAAAA<br>AAAGGTTTACAAAAAATTTTAAATGTTCAAAAAAAATT<br>ACCAATTATTCAAAAAATTATTATTATGGATTCAAAAAC<br>AGATTATCAAGGTTTTCAATCAATGTATACATTTGTTAC<br>ATCACATTTACCACCAGGTTTTAATGAATATGATTTTGT<br>TCCAGAATCATTTGATCGTGATAAAACAATTGCTTTAAT<br>TATGAATTCATCAGGTTCAACAGGTTTACCAAAAGGTG<br>TTGCTTTACCACATCGTACAGCTTGTGTTCGTTTTTCAC<br>ATGCTCGTGATCCAATTTTTGGTAATCAAATTATTCCAG<br>ATACAGCTATTTTATCAGTTGTTCCATTTCATCATGGTTT<br>TGGTATGTTTACAACATTAGGTTATTTAATTTGTGGTTT<br>TCGTGTTGTTTTAATGTATCGTTTTGAAGAAGAATTATT<br>TTTACGTTCATTACAAGATTATAAAATTCAATCAGCTTT<br>ATTAGTTCCAACATTATTTTCATTTTTTGCTAAATCAACA<br>TTAATTGATAAATATGATTTATCAAATTTACATGAAATT<br>GCTTCAGGTGGTGCTCCATTATCAAAAGAAGTTGGTGA<br>AGCTGTTGCTAAACGTTTTCATTTACCAGGTATTCGTCA<br>AGGTTATGGTTTAACAGAAACAACATCAGCTATTTTAA<br>TTACACCAGAAGGTGATGATAAACCAGGTGCTGTTGGT<br>AAAGTTGTTCCATTTTTTGAAGCTAAAGTTGTTGATTTA<br>GATACAGGTAAAACATTAGGTGTTAATCAACGTGGTGA<br>ATTATGTGTTCGTGGTCCAATGATTATGTCAGGTTATGT<br>TAATAATCCAGAAGCTACAAATGCTTTAATTGATAAAG<br>ATGGTTGGTTACATTCAGGTGATATTGCTTATTGGGAT<br>GAAGATGAACATTTTTTTATTGTTGATCGTTTAAAATCA<br>TTAATTAAATATAAAGGTTATCAAGTTGCTCCAGCTGA<br>ATTAGAATCAATTTTATTACAACATCCAAATATTTTTGA<br>TGCTGGTGTTGCTGGTTTACCAGATGATGATGCTGGTG<br>AATTACCAGCTGCTGTTGTTGTTTTAGAACATGGTAAA

		ACAATGACAGAAAAAGAAATTGTTGATTATGTTGCTTC<br>ACAAGTTACAACAGCTAAAAAATTACGTGGTGGTGTTG<br>TTTTTGTTGATGAAGTTCCAAAAGGTTTAACAGGTAAA<br>TTAGATGCTCGTAAAATTCGTGAAATTTTAATTAAAGCT<br>AAAAAAGGTGGTAAAATTGCTGTT
**pME_Cp_0_4_035_aphA6-Kanamycin-marker**	CDS	ATGACCATGGAATTACCAAATATTATTCAACAATTTATC<br>GGAAACAGCGTTTTAGAGCCAAATAAAATTGGTCAGTC<br>GCCATCGGATGTTTATTCTTTTAATCGAAATAATGAAAC<br>TTTTTTTCTTAAGCGATCTAGCACTTTATATACAGAGAC<br>AACATACAGTGTCTCTCGTGAAGCGAAAATGTTGAGTT<br>GGCTCTCTGAGAAATTAAAGGTGCCTGAACTCATCATG<br>ACTTTTCAGGATGAGCAGTTTGAATTCATGATCACTAA<br>AGCGATCAATGCAAAACCAATTTCAGCGCTTTTTTTAAC<br>AGACCAAGAATTGCTTGCTATCTATAAGGAGGCACTCA<br>ATCTGTTAAATTCAATTGCTATTATTGATTGTCCATTTAT<br>TTCAAACATTGATCATCGGTTAAAAGAGTCAAAATTTTT<br>TATTGATAACCAACTCCTTGACGATATAGATCAAGATG<br>ATTTTGACACTGAATTATGGGGTGACCATAAAACTTAC<br>CTAAGTCTATGGAATGAGTTAACCGAGACTCGTGTTGA<br>AGAAAGATTGGTTTTTTCTCATGGCGATATCACGGATA<br>GTAATATTTTTATAGATAAATTCAATGAAATTTATTTTTT<br>AGATCTTGGTCGTGCTGGGTTAGCAGATGAATTTGTAG<br>ATATATCCTTTGTTGAACGTTGCCTAAGAGAGGATGCA<br>TCGGAGGAAACTGCGAAAATATTTTTAAAGCATTTAAA<br>AAATGATAGACCTGACAAAAGGAATTATTTTTTAAAAC<br>TTGATGAATTGAAT

**pME_Cp_0_4_059__malate_synthase_Cm**	CDS	GGTTCTTTAGGTATGTACTCTGAATCTGCTGTACGTAAA<br>AAATCTTCTCGTGGTTACGATGTACCAGAAGGTGTAGA<br>TATTCGTGGTCGTTACGATGAAGAATTCGCTCGTATTTT<br>AAACAAAGAAGCTTTATTATTCGTAGCTGATTTACAAC<br>GTACTTTCCGTAACCACATTCGTTACTCTATGGAATGTC<br>GTCGTGAAGCTAAACGTCGTTACAACGAAGGTGCTGTA<br>CCAGGTTTCGATCCAGCTACTAAATACATTCGTGAATCT<br>GAATGGACTTGTGCTTCTGTACCACCAGCTGTAGCTGA<br>TCGTCGTGTAGAAATTACTGGTCCAGTAGAACGTAAAA<br>TGATTATTAACGCTTTAAACTCTGGTGCTAAAGTATTCA<br>TGGCTGATTTCGAAGATGCTTTATCTCCAAACTGGGAA<br>AACTTAATGCGTGGTCAAATTAACTTAAAAGATGCTGT<br>AGATGGTACTATTTCTTTCCACGATAAAGCTCGTAACAA<br>AGTATACAAATTAAACGATCAAACTGCTAAATTATTCGT<br>ACGTCCACGTGGTTGGCACTTCGCTGAAGCTCACATTTT<br>CATTGATGGTGAACCAGCTACTGGTTGTTTAGTAGATT<br>TCGGTTTATACTTCTTCCACAACCACGCTAACTTCCGTC<br>GTTCTCAAGGTCAAGGTTCTGGTCCATTCTTCTACTTAC<br>CAAAAATGGAACACTCTCGTGAAGCTAAAATTTGGAAC<br>TCTGTATTCGAACGTGCTGAAAAAATGGCTGGTATTGA<br>ACGTGGTTCTATTCGTGCTACTGTATTAATTGAAACTTT<br>ACCAGCTGTATTCCAAATGGATGAAATTTTATACGAATT<br>ACGTGATCACTCTGTAGGTTTAAACTGTGGTCGTTGGG<br>ATTACATTTTCTCTTACGTAAAAACTTTCCAAGCTCACTT<br>AGATCGTTTATTACCAGATCGTGTACAAGTAGGTATGG<br>CTCAACACTTCATGCGTTCTTACTCTGATTTATTAATTCG<br>TACTTGTCACACTGTAGTATGTCACGTAGGTGGTATGG<br>CTGCTCAAATTCCAATTCGTGATGATCCAAAAGCTAAC<br>GAAATGGCTTTAGAATTAGTACGTAAAGATAAATTACG<br>TGAAGCTAAAGCTGGTCACGATGGTACTTGGGCTGCTC<br>ACCCAGGTTTAATTCCAGCTTGTATGGAAGTATTCACTA<br>ACTCTATGGGTAACGCTCCAAACCAAATTCGTTCTGCTC<br>GTCGTGATGATGCTGCTAACTTAACTGAAGATGATTTA<br>TTACAACAACCACGTGGTGTACGTACTTTAGAAGGTTT<br>ACGTTTAAACACTCGTGTAGGTATTCAATACTTAGCTGC<br>TTGGTTAACTGGTACTGGTTCTGTACCATTATACAACTT<br>AATGGAAGATGCTGCTACTGCTGAAATTTCTCGTGTAC<br>AAAACTGGCAATGGTTAAAATACGGTGTAGAATTAGAT<br>GGTGATGGTTTAGGTGTACGTGTAAACAAAGAATTATT<br>CGCTCGTGTAGTAGAAGAAGAAATGGAACGTATTGAA<br>CGTGAAGTAGGTAAAGAAAAATTCCGTAAAGGTATGT<br>ACAAAGAAGCTTGTAAAATGTTCACTCGTCAATGTACT<br>GCTCCAACTTTAGATGATTTCTTAACTTTAGATGCTTAC<br>AACCACATTGTAATTCACCACCCACGTGAATTA

**pME_Cp_0_4_060_CrGlDh**	CDS	GGTGCTCGTGGTCCAGCTTCTCCATCTTCTTTAGAACAA<br>CAAACTCGTCAAGTAGCTCAAGTAGCTGTACAACAATC<br>TACTCAACAAGCTGTAAAAGTAGTAGTACCAGCTATTA<br>AAGTAGATTTAGTAGGTGCTGTATCTTCTGTATCTGAAT<br>CTGATAAAGTAGAACCAGGTGTATTCAAAAACGTAGAT<br>GGTCACCGTTTCGAAGATGGTCGTTACGCTGCTTTCGT<br>AGAAGAAATTACTAAATTCATTCCAAAAGAACGTCAAT<br>ACTCTGATCCAGTACGTACTTTCGCTTACGGTACTGATG<br>CTTCTTTCTACCGTTTAAACCCAAAATTAGTAGTAAAAG<br>TACACAACGAAGATGAAGTACGTCGTATTATGCCAATT<br>GCTGAACGTTTACAAGTACCAATTACTTTCCGTGCTGCT<br>GGTACTTCTTTATCTGGTCAAGCTATTACTGATTCTGTA<br>TTAATTAAATTATCTCACACTGGTAAAAACTTCCGTAAC<br>TTCACTGTACACGGTGATGGTTCTGTAATTACTGTAGA<br>ACCAGGTTTAATTGGTGGTGAAGTAAACCGTATTTTAG<br>CTGCTCACCAAAAGAAAAACAAATTACCAATTCAATAC<br>AAAATTGGTCCAGATCCATCTTCTATTGATTCTTGTATG<br>ATTGGTGGTATTGTATCTAACAACTCTTCTGGTATGTGT<br>TGTGGTGTATCTCAAAACACTTACCACACTTTAAAAGAT<br>ATGCGTGTAGTATTCGTAGATGGTACTGTATTAGATAC<br>TGCTGATCCAAACTCTTGTACTGCTTTCATGAAATCTCA<br>CCGTTCTTTAGTAGATGGTGTAGTATCTTTAGCTCGTCG<br>TGTACAAGCTGATAAAGAATTAACTGCTTTAATTCGTC<br>GTAAATTCGCTATTAAATGTACTACTGGTTACTCTTTAA<br>ACGCTTTAGTAGATTTCCCAGTAGATAACCCAATTGAA<br>ATTATTAAACACTTAATTATTGGTTCTGAAGGTACTTTA<br>GGTTTCGTATCTCGTGCTACTTACAACACTGTACCAGAA<br>TGGCCAAACAAAGCTTCTGCTTTCATTGTATTCCCAGAT<br>GTACGTGCTGCTTGTACTGGTGCTTCTGTATTACGTAAC<br>GAAACTTCTGTAGATGCTGTAGAATTATTCGATCGTGC<br>TTCTTTACGTGAATGTGAAAACAACGAAGATATGATGC<br>GTTTAGTACCAGATATTAAAGGTTGTGATCCAATGGCT<br>GCTGCTTTATTAATTGAATGTCGTGGTCAAGATGAAGC<br>TGCTTTACAATCTCGTATTGAAGAAGTAGTACGTGTATT<br>AACTGCTGCTGGTTTACCATTCGGTGCTAAAGCTGCTC<br>AACCAATGGCTATTGATGCTTACCCATTCCACCACGATC<br>AGAAAAACGCTAAAGTATTCTGGGATGTACGTCGTGGT<br>TTAATTCCAATTGTAGGTGCTGCTCGTGAACCAGGTAC<br>TTCTATGTTAATTGAAGATGTAGCTTGTCCAGTAGATAA<br>ATTAGCTGATATGATGATTGATTTAATTGATATGTTCCA<br>ACGTCACGGTTACCACGATGCTTCTTGTTTCGGTCACGC<br>TTTAGAAGGTAACTTACACTTAGTATTCTCTCAAGGTTT<br>CCGTAACAAAGAAGAAGTACAACGTTTCTCTGATATGA<br>TGGAAGAAATGTGTCACTTAGTAGCTACTAAACACTCT<br>GGTTCTTTAAAAGGTGAACACGGTACTGGTCGTAACGT<br>AGCTCCATTCGTAGAAATGGAATGGGGTAACAAAGCTT<br>ACGAATTAATGTGGGAATTAAAAGCTTTATTCGATCCA<br>TCTCACACTTTAAACCCAGGTGTAATTTTAAACCGTGAT<br>CAAGATGCTCACATTAAATTCTTAAAACCATCTCCAGCT<br>GCTTCTCCAATTGTAAACCGTTGTATTGAATGTGGTTTC<br>TGTGAATCTAACTGTCCATCTCGTGATATTACTTTAACT<br>CCACGTCAACGTATTTCTGTATACCGTGAAATGTACCGT<br>TTAAAACAATTAGGTCCAGGTGCTTCTGAAGAAGAGAA<br>AAAACAATTAGCTGCTATGTCTTCTTCTTACGCTTACGA<br>TGGTGAACAAACTTGTGCTGCTGATGGTATGTGTCAAG<br>AAAAATGTCCAGTAAAAATTAACACTGGTGATTTAATT<br>AAATCTATGCGTGCTGAACACATGAAAGAAGAAAAAA<br>CTGCTTCTGGTATGGCTGATTGGTTAGCTGCTAACTTCG<br>GTGTAATTAACTCTAACGTACCACGTTTCTTAAACATTG<br>TAAACGCTATGCACTCTGTAGTAGGTTCTGCTCCATTAT<br>CTGCTATTTCTCGTGCTTTAAACGCTGCTACTAACCACT<br>TCGTACCAGTATGGAACCCATACATGCCAAAAGGTGCT<br>GCTCCATTAAAAGTACCAGCTCCACCAGCTCCAGCTGC

		TGCTGAAGCTTCTGGTATTCCACGTAAAGTAGTATACA TGCCATCTTGTGTAACTCGTATGATGGGTCCAGCTGCTT CTGATACTGAAACTGCTGCTGTACACGAAAAAGTAATG TCTTTATTCGGTAAAGCTGGTTACGAAGTAATTATTCCA GAAGGTGTAGCTTCTCAATGTTGTGGTATGATGTTCAA CTCTCGTGGTTTCAAAGATGCTGCTGCTTCTAAAGGTG CTGAATTAGAAGCTGCTTTATTAAAAGCTTCTGATAAC GGTAAAATTCCAATTGTAATTGATACTTCTCCATGTTTA GCTCAAGTAAAATCTCAAATTTCTGAACCATCTTTACGT TTCGCTTTATACGAACCAGTAGAATTCATTCGTCACTTC TTAGTAGATAAATTAGAATGGAAAAAAGTACGTGATCA AGTAGCTATTCACGTACCATGTTCTTCTAAGAAAATGG GTATTGAAGAATCTTTCGCTAAATTAGCTGGTTTATGTG CTAACGAAGTAGTACCATCTGGTATTCCATGTTGTGGT ATGGCTGGTGATCGTGGTATGCGTTTCCCAGAATTAAC TGGTGCTTCTTTACAACACTTAAACTTACCAAAAACTTG TAAAGATGGTTACTCTACTTCTCGTACTTGTGAAATGTC TTTATCTAACCACGCTGGTATTAACTTCCGTGGTTTAGT ATACTTAGTAGATGAAGCTACTGCTCCTAAAAAACAAG CTGCTGCTGCTAAAACTGCT
**pME_Cp_0_5_001_psbA_3'UTR_Cr**	3'UTR	TTTTTTTTTAAACTAAAATAAATCTGGTTAACCATACCT GGTTTATTTTAGTTTATACACACTTTTCATATATATATAC TTAATAGCTACCATAGGCAGTTGGCAGGACGTCCC

**pME_Cp_0_5_005_rrnB_3'UTR_Ec**	3'UTR	GTAGAAACGCAAAAAGGCCATCCGTCAGGATGGCCTTCTGCTTAATTTGATGCCTGGCAGTTTATGGCGGGCGTCCTGCCCGCCACCCTCCGGGCCGTTGCTTCGCAACGTTCAAATCCGCTCCCGGCGGATTTGTCCTACTCAGGAGAGCGTTCACCGACAAACAACAGATAAAACGAAAGGCCCAGTCTTTCGACTGAGCCTTTCGTTTTATTTGATG
**pME_Cp_0_5_006_psbA_3'UTR_Nt**	3'UTR	TCCTGGCCTAGTCTATAGGAGGTTTTGAAAAGAAAGGAGCAATAATCATTTTCTTGTTCTATCAAGAGGGTGCTATTGCTCCTTTCTTTTTTT
**pME_Cp_0_5_007_atpA_3'UTR_Cr**	3'UTR	TTTTAATTAAGTAGGAACTCGGTATATGCTCTTTTGGGGTCTTATTAGCTAGTATTAGTTAACTAACAAAAGATCAATATTTTAGTTTGTTTTATATATTTTATTACTTAAGTAGTAAGGATTTGCATTTAGCAATCTTAAATACTTAAGTAATAATCTATAAATAAAATATATTTTCGCTTTAAAACTTATAAAAATTATTTGCTCGTTATAAGCCTAAAAAAACGTAGGATCTCTACGAGATATTACATTGTTTTTTTCTTTAATTGGCTTTAATATTACTTTGTATATATAAACCAAAGTACTTGTTAATAGTTATTAAATTATATTAACTATACAGTACAAAGAAATTTTTTGCTAAAAAAAGTATGTTAACATTAAAAATTTTTGTTTATACAG
**pME_Cp_0_5_009_rbcl_3'UTR+rbcl-CDS_Cr**	3'UTR	GTACTCAAGCTCGTAACGAAGGTCGTGACCTTGCTCGTGAAGGTGGCGACGTAATTCGTTCAGCTTGTAAATGATCTCCAGAACTTGCTGCTGCATGTGAAGTTTGGAAAGAAATTAAATTCGAATTTGATACTATTGACAAACTTTAATTTTTATTTTTCATGATGTTTATGTGAATAGCATAAACATCGTTTTTATTTTTTATGGTGTTTAGGTTAAATACCTAAACATCATTTTACATTTTTAAAATTAAGTTCTAAAGTTATCTTTTGTTTAAATTTGCCTGTCTTTATAAATTACGATGTGCCAGAAAAATAAAATCTTAGCTTTTTATTATAGAATTTATCTTTATGTATTATATTTTATAAGTTATAATAAAAGAAATAGTAACATACTAAAGCGGATGTA
**pME_Cp_0_5_010_atpB_3'UTR_Cr**	3'UTR	TTTAATTTTTTATGTGTAATTAAAATAAATATTTGGACACCATTAAGTTGTTTTCTTCTTAAAGAGCCAATTTATTTTAATTACAC
**pME_Cp_0_5_011_chlB_3'UTR_Cr**	3'UTR	AAATAATCTAAATTTTCAGGATGAAATACTGCGCTAGCGGGCAGTCACAAACTACTATCTTCGAACGCAG
**pME_Cp_0_5_012_clpP_3'UTR_Cr**	3'UTR	TAATATGGAATTCATCTTTTAGTTGTGTAGCTTGCATAAGATATTACTCTAACTGGTCTATAGCTTGACCGCAGGTTACATATATATTCGTTTTTATATTAAGGGATATCACTACAAG
**pME_Cp_0_5_013_ftsH_3'UTR_Cr**	3'UTR	TAATATTTAAACAGTATTAATAAAACATACGGCAGCTTCTAACTGCACCATGTGAATACTATTTCTTGTGCTAGTTACTCTCACTCCGCTTCGTTATTGTGTAGCCCTGTAAGGAACGTCCAACTAACCATATCTATATCCCTAATAAATTATCTTCCCCGAGAAAGAGTAAAATTAGGGTCTGTAGATATAAGGGCTGCACGATAGCGTAAACAACACCAATAAAAGTAAGCCATTTCATGGTTATACTGCTCATGGACAAAAAAATTGAATAACTTGCAAATTATAATGACGAGGGTTAAGCAATGGTACACTCATGGTGGCAAGTTCGATAAACTTTAGTTACCC

**pME_Cp_0_5_015_petA_3'UTR_Cr**	3'UTR	TATTTAATTTTTTGTAGGGCTGCTGTGCAGCTCCTACAA<br>ATTTTAGTATGTTATTTTTAAAGTTTGATATACTGAAAA<br>CAAAGTTCTACTTGAACGATATTTAGCTTTTAATGCTAT<br>AATATAGCGGACTAAGCCGTTGGCAATTTAGCTGCCAA<br>TTAATTTTATTCGAAGGATGTAAACCTGCTAACGATATT<br>TATATATAAGCATTTTAATACTCCGAGGGAGGCCTCTA<br>ACCTTTAGCAAGTAAGTAAACTTCCCCTTCGGGGCAGC<br>AAGGCAGCAGATTTAAATTCTCCAAAGGAGGCAGTTG<br>ATATCAGTAAACCCCTTCGATGACTCTGGCATTGATGC<br>AAAGCATGGGGAAACTAAAGTTCCTCCACTGCCTCCTT<br>CCCCTTCCCTTTCGGGACGTCCCCTTCCCCTTACGGGCA<br>AGTAAACTTAGGGATTTTAATGCAATAAATAAATTTGT<br>CCCCTTACGGGACGTCAGTGGCAGTTGCGAAGTATTAA<br>TATTGTATATAAATATAGAATGTTTACATACTCCGAAGG<br>AGGACGTCAGTGGCAGTGGTACCGCCACTGCCTATTTT<br>AATACTCCGAAGGAGGCAGTGGTGGTCCCACTGCCACT<br>AAAATTTATTTGC
**pME_Cp_0_5_017_petD_3'UTR_Cr**	3'UTR	TCTAAAATTTTAAATTTCCCTCTAGGGTTGCAATACGAT<br>TTGCAACCCTGAAGGGGGAAAACTGAGTTCTGTCATTT<br>TTTTAAGAACTCCATCAATAAATTGTGTCTTAGCAGATA<br>ATGCGGGTAAATAACACCCTTGTTCATCCTACAAAATA<br>GGAGATTCCATTTAATTTAAACCAAACAAATAACTTAGT<br>TACAAC
**pME_Cp_0_5_018_petL_3'UTR_Cr**	3'UTR	TTTATTAATGAGTAAAACCACGCTAATGGGTTTTTACTA<br>CTACATTTTTTATATATAATTGTAAGAGCACAGCAACGT<br>GCTCTTACATTTTCCGGTTATTTAAGTACCTAACTAAATT<br>CAAATAAATGCGTTAAGTTTGAGACTGCAAAGCTGCCA
**pME_Cp_0_5_019_psaB_3'UTR_Cr**	3'UTR	TGTAATGTACTTTATTTACTACCTCATCAGATCAGCATA<br>TACGTGCGACCCGATGTGGTACTTTTAAATCGGTTAGA<br>CATTTAGT
**pME_Cp_0_5_020_psaC_3'UTR_Cr**	3'UTR	TTTTTTAATTCACTTGGCGTTTAAAAAACCTTTAAAAAA<br>GGGAGTATCTTGGTGAGATCCTCCATAAATTTTAAATT<br>AGGTAGCCTCTTCCTTTGGGTACATTTATTAATATAAAT<br>GTGCCCAAAGTTCTTAACAAGAAAATATAAATTCCCAT<br>AAACTTTTGGCTTATTTAATTGGAAC
**pME_Cp_0_5_021_psbA_3'UTR_Cr**	3'UTR	TTTTTTTTTAAACTAAAATAAATCTGGTTAACCATACCT<br>GGTTTATTTTAGTTTATACACACTTTTCATATATATATAC<br>TTAATAGCTACCATAGGCAGTTGGCAG
**pME_Cp_0_5_022_psbC_3'UTR_Cr**	3'UTR	TTTTTATTATTATTAAAATTGGGTTGTTACCATGTAACA<br>ACCCAATTTTACAACTATCAGATTTGTTATTTACTAACA<br>AACCATTGAAATCGCTCATCTGACACGGAAAATGCCAA<br>TCTTTTTAATAACTTTATAGCATTTTCTA
**pME_Cp_0_5_026_rbcL_3'UTR_Cr**	3'UTR	TTTTTATTTTTCATGATGTTTATGTGAATAGCATAAACAT<br>CGTTTTTATTTTTATGGTGTTTAGGTT
**pME_Cp_0_5_028_rpl36_3'UTR_Cr**	3'UTR	AAATCTACTTTAGGAAGCCCCTTTCCCCTTCCCCTTCGG<br>GACGTCCCCTTCGGGCAAATAAATTTTAGTGGACGTCA<br>GTGGCAGTTGCCTGCCAACTGCCGATATTTATATACTA<br>GGCAGTGGCGGTACCACTGCCACTGACGTCCTAAGTTT<br>ACTTGCCCAATATTTATATTAGGCAGTTGGCAGGCAAC<br>TGCCACTGACGTCCCGAAGGGGAAGGGGAAGGAGGC<br>AGTTGCCTGCCAACTGCCTCCTTACGGAGTATTAAAAT<br>AGGCAGTGGCGGTACCACTGCCACTGACGTCCTAATAT<br>AAATATTAGGGCGTCCCGTAAGAGGACGTCATAGGCA<br>AGTAGACTTAAGGAAGCCAGTTGATATTTATTTACGCC<br>AAAAGGAGGTAAGTAACCTATGTTTCGCTATTTCTGTCT<br>AAATGCGCT

**pME_Cp_0_5_030_rpoB2_3'UTR_Cr**	3'UTR	TTCTATGCATAAATGAAATTATTTTATTATTTTTTTAATCACTTTATGAATCTGGTTCTTTCATTTTTTAGAATCTTGTTAGCAAATTTCTCTACCAGAATGGGATGGCATTGGCAGTTGGAAAACCCGGTACAAGTCAATACATTCTTTAGTTTTTTGCATATTCGAAAGGACGTCCCCTTACGGGACGTCCCTTTTGGGACGCCAGTGGACGTCAGTGGCAGTGGTACCGCCACTGCCTCCTTCCCCTTCCCCTTCGGGACGTCCCCTTACGGGAATATAAATATTAGTGGCAGTTGCCTCCTTCCCTTTCCCCTTCGGGACGTCCCCTTACGGGAATATAAATATTAGTGGCAGTTGCCTCCTTCCCTTTCCCCTTCGGGACGTCCCCTTACGGGAATATAAATATTAGTGGCAGTTGCCTCCTTCCCTTTCCCCTTCGGGACGTCCCCTTACGGGAATATAAATATTAGTGGCAGTTGCCTCCTTCCCCTTCGGGCAAGTAAACTTAGGAGTATATAAATATAGGATGTTAATACTGCGGAGCAGGCAGTGGCGGTACCACTGCCACTAAAATTTATTTGCCCGAAGGGGACGTCCTGCCAACTGCCTAGGCAGCCAATAAATTTTATCGCAGTATATGTATATGCTTCCCCTTCTGGGATAGAAGTAAACATCTCCGAAGGCACGTTCTTTACGGTCTATATATTCTTGCAGTGGCTTGCGACTGCTACTAATGTAATGTTCCCAAGGGGTTTAATCCCGATTTAAG
**pME_Cp_0_5_031_rpoC2_3'UTR_Cr**	3'UTR	AATTCTTTCTTTTTGTTAAAAGATTTCTAAAAGTAAAGTATTAACTTT
**pME_Cp_0_5_032_rps2_3'UTR_Cr**	3'UTR	ATACTAACCATATGGTTTAGTTTTTTTTTAGTTGTCATTCTAGGACGTCCCCTTACGGGAATATAAATATTAGTGGCAGTGGTACCGCCACTGCCTGCTCCGCAGTATTAACATCCTATTTTAATACTCCGAAGGAGGCAGTTGGTTCAGCCTCTGCCATTCAAGTAGATATACCCAACGAGGATATTCTTTTTAATATCCTCGTTGGGTATATCTACTTTTTGATTAGTATTTATATACTTAATATGCAAACTATTCC
**pME_Cp_0_5_034_rps4_3'UTR_Cr**	3'UTR	TTTAATCGGTAAAATTCCCAGGTAGTTGCACCTTTCCCTTCAGGACGTCCCCTTCCCCTTCGGGACAATAAATAAATTTGTTGCCTGCCAACAAATTTATTTATTGTATTAAAATAGAATAAAATTTATTTGCTGCGGTAGCAGGTTTACATACAATTTATTTATTGTACCACTGCCACTGGCGTCCTCCTTCGGAGTATGTAAACATGCTAAGTTTACTTGCCCAATATTTATATTAGGCAGTTGGCAGGCAACTGCCACTAAAATTTATTTACCCGAAGGGGACGTCCCGAAGGGGAAGGGGAAGGAGGACGTCCCCTTACGGGAATATAAATATTAGTGGACGTCAGTGGCGGTACCACTGCCACTGGCGTCCTATATTTATATACTCCTAAGTTTACTTGCCCAATATTTATATACCCGAAGGGGAAGGGGAAGCATATAAATATACTTCGGAGTATATAAATATAGGATGTTAATACTGCGGAGCAGGCAGTTGGCAGGACGTCCCCTTCGGGATTTTAATGCTCCGTTAGGAGGCAAATAAATTTTAGTGGCAGTGGTACCGCCACTGCCTGCTTCCTCCTTCGGAGTATGTAAACCCCTTCGGGCAACTAAAGTTTATCGCAGTATATAAATATAGGCAGTTGGCAGGCAACTGCCACTGACGTCCTATATTTATATACTCCGAAGGAACTTGTTAGCCGATAGGCGAGGCAACTGCCACTAAAATTTATTTGCCCGAAGGGGACGTCCACTAATATTTATATTCCCGTAAGGGGACAAATTTATTTATTGCATTAAAATCCCTAAGTTTACTTGCCTCCTTCGGAGTATATAAATATCCCGTAAGGGGAAGGGGACGTCCTAGTATATAAATATCGGCAGTTGGCAGGCAACAAATTTATTTATTGTCCCGTAAGGGTTTGCAAGAATACTGTAAGGGGATGTCTTTTTAAATTCAATTCAAAAAAATCAAATAAAAACTATTATAATTTTGACCAAATTACCAAATAAATTACCAGATTAAATGTATTTTATTTTCCAAATTATACCAG

**pME_Cp_0_5_035_rps8_3'UTR_Cr**	3'UTR	CAATTGGTAACTTTAGAAAAATTAGAAATAAATCCTATGCTGCAAAATATTCTTTTGCAGTCGCCTTCCAATTAATAAATTATTAAAACCACGTTTTGTAAATCGGTAGGCTAGCAGTTGTTGTTCCCAAATCTCTATTTACTATAAGTAAATTTCAAAATAAATTAATGTAATACCCTCTCCCAAAGTGGTACTATAACTAACTAAATTGACATACTGCTAAATTAATTTAAGTAATATTTACAAGGTCAACTTCTGTCAATAATTAGATTTTAAAATAAGTGTTTTTGGGAGGAAAGGTAAATACATTTCTGGTACCTCTGGTGATGCCATATTAAATATTTTTACAGAGGTAGCCAGTTTTAAATTTATAAAAAA
**pME_Cp_0_5_036_rps11_3'UTR_Cr**	3'UTR	TTTTTAAAAAATTAAAGATATACCAATTTATTTTTTGTATACAAGTTTTATAGTGCAATATTTTTATAAAATATTTCCTTGATTATGCTTGAGTTTTAGTATTTTACCATGCTCATGTCAACCATAAACTTGTATACAAAACATTAACAATTTTTTATTTATTGAAATTGAAATTTATTTCTATTTAAAAATGCAG
**pME_Cp_0_5_038_rps19_3'UTR_Cr**	3'UTR	TTTTTTGTTATTTACTGCTATTTGGTACACCTTAGTTTCCTAAACTAATTTCTATAAACTACTATTCTTTGCAGTTAACCGGAATATAAACATCGACTTTGGGAAACCAGTTGGTAAGAACTTGTTGTCTTGCAGCTCTTTGCGCGCTGCCAGACGGCAAGTTCTTTTCCCTTCGGAAGGCAGCTAATTATGTTTATAGTCTATTTGCAATGCCACTCTGAGTAAATAAATTTCCCCTTTGTGATATTAATATGAGCTGCCACTACCATCCTCTTAGAAGTATATAAATATGCACTGGCATCCTAGAGAAATTAATTACTTTTATAGCTATAGAATGCTTGTTAAGGGATTTACTACTATAAAATTAATGTGCTCCTTGGGGTAAATACACTTAATAAATCCCTTTAGGTATTTAAATAGCCTATTTGGGTAAAGGCTTTTTAAATATGTAATAAATTATATATATTAGTTTTATGTGGTTTTTTATATCTATGGGAACTTTACTTTTTTATACTGTTAAATTCCTAGTTAAGAGTAATAAGAGCC
**pME_Cp_0_5_039_tufA_3'UTR_Cr**	3'UTR	TTTCACATTTAAATTCACTAACGAGCCCTTAACGGACGTCCCCTTTGGGATGCCAAGGCTCTGTTAGGATATAATACTGCGTAAGCAGGAATTTGCTATTTATTTACCCGAAAAGGTTCGACCATAGGTGAGGACAAATTTATTTATTGTGGTACCGCCACTGCCTCCTTCGGAGTATTAAAATCCTATATTTATATTCTCTGAAAAAAGTTCCCTTCGGG
**pME_Cp_0_5_040_ycf1_3'UTR_Cr**	3'UTR	AAATATGCCATATTTTTACTAATTTTATATTCCAAGACATTGTATGCTTATAAATAAAGAAGATGCTTTGCATCTCTAAAGATGAG
**pME_Cp_0_5_041_ycf12_3'UTR_Cr**	3'UTR	TTTAAAATAGCATAGCAATTGCTAGCCTTATAAAATGTATGTACCTGAAAGTGACGTTTAGACTTTCGGGTACATACATACTTCTTAATAACAAAGAAAAATTTTGTTCACAAACACACATTAACACTTATAAATAGCTTACTTTAATCTGCATAGCAGCTTCTTGAACGTATTTATTATGTATTCGAAGTTTATATCTTTATGGTCATATAAAGGCTGTTTGTGACAATCTTTAAAGTTAATGACTAAGAAACAATATTGGAGATCAAC
**pME_Cp_0_5_042_rbcL_3'UTR_Nt**	3'UTR	AAACAGTAGACATTAGCAGATAAATTAGCAGGAAATAAAGAAGGATAAGGAGAAAGAACTCAAGTAATTATCCTTCGTTCTCTTAATTGAATTGCAATTAAACTCGGCCCAATCTTTTACTAAAAGGATTGAGCCGAATA
**pME_Cp_0_5_043_BMV_3'UTR_Bv**	3'UTR	GGTGCCTTTGAGAGTCTACTTTTGCTCTCTTCGGAAGAACCCTTAGGGGTTCGTGCATGGGCTTGCATAGCAAGTCTAGATGCGGGTACCGTACAGTGTTGAAAAACACTGTAAATCTCTAAAAGAAACCA
**pME_Cp_0_5_044_TYMV_3'UTR_Tv**	3'UTR	GTTCTCGATCTTTAAAATCGTTAGCTCGCCAGTTAGCGAGGTCTGTCCCCACACGACAGATAATCGGGTGCAACTCCCGCCCCTTTTCCGAGGGTCATCGGAACC

**pME_Cp_0_5_045_rpoA_3'UTR_Nt**	3'UTR	ATCTATTGGACTTACTTAGTGAAAATAGATTGAAACTCTTTAGCACAATTAATATATTAATATATCATATATTCAAAATAAATTCAGAATTAAATTGAATAGATGTATCTAGGGAGAATTCGCTTTGAAGAAACTATTCCCTAGATACACATGTCGTGTTATTTCACAATTGAATCAATTTAAAAAT
**pME_Cp_0_5_046_petD_3'UTR_Nt**	3'UTR	ATTTTTAAATTGATTCAATTGTGAAATAACACGACATGTGTATCTAGGGAATAGTTTCTTCAAAGCGAATTCTCCCTAGATACATCTATTCAATTTAATTCTGAATTTATTTTGAATATATGATATATTAATATATTAATTGTGCTAAAGAGTTTCAATCTATTTTCACTAAGTAAGTCCAATAGAT
**pME_Cp_0_5_047_TMV_3'UTR_Tv**	3'UTR	ATAAATAACGGATTGTGTCCGTAATCACACGTGGTGCGTACGATAACGCATAGTGTTTTTCCCTCCACTTAAATCGAAGGGTTGTGTCTTGGATCGCGCGGGTCAAATGTATATGGTTCATATACATCCGCAGGCACGTAATAAAGCGAGGGGTTCGAATCCCCCCGTTACCCCCGGTAGGGGCCCA
**pME_Cp_0_5_048_psaC_3'UTR_Nt**	3'UTR	TACGTTCGAGAAAACTCTACTTGAATCCATTTAATTTTTTTTACCGACAAACCTGTGCTCGAAAATCACAATATTTTGAGCACGGGTTTTTATG
**pME_Cp_0_5_049_TDummy**	3'UTR	ACTCAGTTGTAGTAACGAGCGGATAGATTCCCAGACCCACCTTCACGGGCGGTAGCAGGACCTCAATAATAGGATTTTCG
**pME_Cp_0_05_050_Terminator_Placeholder**	3'UTR	GAAAGTGAAACGTGATTTCATGCGTCATTTTGAACATTTTGTAAATCTTATTTAATAATGTGTGCGGCAATTCACATTTAATTTATGAATGTTTTCTTAACATCGCGGCAACTCAAGAAACGGCAGGTTCGGATCTTAGCTACTAGAGAAAGAGGAGAAATACTAGATGCGTAAAGGCGAAGAGCTGTTCACTGGTGTCGTCCCTATTCTGGTGGAACTGGATGGTGATGTCAACGGTCATAAGTTTTCCGTGCGTGGCGAGGGTGAAGGTGACGCAACTAATGGTAAACTGACGCTGAAGTTCATCTGTACTACTGGTAAACTGCCGGTTCCTTGGCCGACTCTGGTAACGACGCTGACTTATGGTGTTCAGTGCTTTGCTCGTTATCCGGACCATATGAAGCAGCATGACTTCTTCAAGTCCGCCATGCCGGAAGGCTATGTGCAGGAACGCACGATTTCCTTTAAGGATGACGGCACGTACAAAACGCGTGCGGAAGTGAAATTTGAAGGCGATACCCTGGTAAACCGCATTGAGCTGAAAGGCATTGACTTTAAAGAGGACGGCAATATCCTGGGCCATAAGCTGGAATACAATTTTAACAGCCACAATGTTTACATCACCGCCGATAAACAAAAAAATGGCATTAAAGCGAATTTTAAAATTCGCCACAACGTGGAGGATGGCAGCGTGCAGCTGGCTGATCACTACCAGCAAAACACTCCAATCGGTGATGGTCCTGTTCTGCTGCCAGACAATCACTATCTGAGCACGCAAAGCGTTCTGTCTAAAGATCCGAACGAGAAACGCGATCATATGGTTCTGCTGGAGTTCGTAACCGCAGCGGGCATCACGCATGGTATGGATGAACTGTACAAATGACCAGGCATCAAATAAAACGAAAGGCTCAGTCGAAAGACTGGGCCTTTCGTTTTATCTGTTGTTTGTCGGTGAACGCTCTCTACTAGAGTCACACTGGCTCACCTTCGGGTGGGCCTTTCTGCGTTTATA
**pME_Cp_0_5a_001_Strep-tag**	C-terminal Tag	GCTTTATGGAGCCACCCGCAGTTCGAGAAGGGGTA
**pME_Cp_0_5a_002_His-tag**	C-terminal Tag	GCTTTACACCATCACCACCATCATGGGTA
**pME_Cp_0_5a_003_Hibit-tag**	C-terminal Tag	GCTTTAGTTTCTGGTTGGCGTTTATTTAAGAAAATTTCTGGGTA
**pME_Cp_0_5a_004_flag-tag**	C-terminal Tag	GATTATAAAGATGACGATGACAAAGGTGACTATAAGGATGATGATGATAAAGGTGACTACAAAGACGACGATGATAAA

**pME_Cp_0_5a_005_HA-tag**	C-terminal Tag	TATCCATATGATGTACCAGATTACGCAAAATACCCATACGATGTTCCTGATTATGCTGGTTATCCTTATGATGTTCCAGATTATGCT
**pME_Cp_0_5a_006_myc-tag**	C-terminal Tag	GAACAAAAACTTATTTCAGAGGAAGATTTAAACGGTGAGCAAAAATTAATTTCTGAGGAAGACTTAAATGGTGAACAAAAACTGATTTCGGAGGAAGATCT
**pME_Cp_0_6_005_psbH_3'Hom_v2_Cr**	3'Homology	TGCCACTGCCGAATATAAATATGGTTGAGTTGCTTAGTTTACCTTAGCGAAAAGAAGACTTAGCAGCTAGCCTTAACAAACAGTTTTATATTTTATGTTTGTGTTAAATAAAATTAAGAAACTTTAGCTAAAGTTTCCCAACTCATAGAAACGTCATCTAAAATTAAAGAACTGTTGTAAATTTCTAAAATGATTAATAAGAATGCTGCAAATAAAAGGATAAATACAGCCATTAAAACAGTTGTACCCCAGCCTGGTAATACTTTACCTGCTTCTGAGTTAAGTGGACGTAATAAAGTACCTAATGGTGTAACTAAACCAGGTTCTTGGAAGTCTGAATTTACTTTTGATGGTTTAGCTTTAGAAGTTCCTGTTGCCATAATTGATTAAATGAATTAAGCGTTATTAGCGCTATTTTATTTACTTTCTGTAAAAAATAAGGAAAATATTCTTCAGTGCATTCCCTCTCAGGATTATAAATACTCTGAGGATAACGTTCTCTCGTCAAGGGGTTGCTTCTTGTGAGTATAGAAACCTACTAGCACAAGAAATAAATTGCATAAAAATGTATTTACCTAGGACCGCAGTAGGCAGTCCCTTTTCCCCTTCAGAACTGCCTGCTTTAAAAGAATGAAAAAACTGCCTTGTCTGGTAAGTAAAACTCTTTAATTACTCACTAAAGACGATCTAGAAGTTCTTTGTTCATTTTTTATTTAATATAATATTTGTTATATAAAAATTAAATAATTTTTAATTAATGTTTAACTTTGTAAGGACAGTTTCAAAGTGACATGAATGGCTACTGCAAAAACGAAGTAAGTTATTCTTTCTCAGGGCAAAATTTTGAGTAGATTAATTTTGTTTAAAAATGTGGGACACAGTCGTCAAGTCTTTTGAACTATCTAAGAGATATGTTGAAAAGAGAATAATTTTATTATTAAATGAGCTATGGAAAGTCCAGCTTTTTTCTTTACCTTTTTTTTATGGTTTCTTCTGTTAAGTGTAACTGGCTATTCAGTTTATGTTAGTTTTGGTCCACCTTCAAAAAAAATTACGTGATCCTTTTGAAGAACACGAAGATTAAACAAGTTAAAAAGTACTATTTTTACAAGTGACTTCGGTGCCTC
**pME_Cp_0_6_006_psbH_5'Hom-reversed_v2_Cr**	5'Homology Reversed	CGTTAAGATAAACGCGTTAATAGCTCACTTTTCTTTAAATTTAATTTTTAATTTAAAGGTGTAAGCAAATTGCCTGACGAGAGATCCACTTAAAGGATGACAGTGGCGGGCTACTGCCTACTTCCCTCCGGGATAAAATTTATTTGAAAAACGTTAGTTACTTCCTAACGGAGCATTGACATCCCCATATTTATATTAGGACGTCCCCTTCGGGTAAATAAATTTTAGTGGACGTCCCCTTCGGGCAAATAAATTTTAGTGGACAATAAATAAATTTGTTGCCTGCCAACTGCCTAGGCAAGTAAACTTGGGAGTATTAAAATAGGACGTCAGTGGCAGTTGCCTGCCAACTGCCTATATTTATATACTGCGAAGCAGGCAGTGGCGGTACCACTGCCACTGGCGTCCTAATATAAATATTGGGCAACTAAAGTTTATAGCAGTATTAACATCCTATATTTATATACTCCGAAGGAACTTGTTAGCCGATAGGCGAGGCAACAAATTTATTTATTGTCCCGTAAAAGGATGCCTCCAGCATCGAAGGGGAAGGGGACGTCCTAGGCCATAAAACTAAAGGGAAATCCATAGTAACTGATGTTATAAATTTATAGACTCCAAAAAACAGCTGCGTTATAAATAACTTCTGTTAAATATGGCCAAGGGGACAGGGGCACTTTCAACTAAGTGTACATTAAAAATTGACAATTC

**pME_Cp_0_6_007_psbA_3'Hom_Cr**	3'Homology	AAAGTATTTGTCGTCTACGATATGTAAATCTGTCGTCTA<br>CGATATGTAAATTTGAGCTCTTATGGCCTCTACATCGAG<br>GTTTATTATCTTACCGAAGGTAAATGCCTTCGTGGATCT<br>TATGGGACGTGATGTGGTCTTCCTGGTTGTTCTATATGT<br>GAATCTGTCGTTTACAATATGTAAATTTGAGCTCTTATG<br>AGCTCTACATCGAGGTTTATTATCTTACCGAAGGTAAAT<br>GCCTTCGTGGATCTTATGGGACGTGATGCGGTCTTCCT<br>GGTTGTTCTATATGTGAATCTGTCGTTTACAATATGTAA<br>ATTTGAGCTCTTATGAGCTCTACATCGAGGTTTATTATC<br>TTACCGAAGGTAAATGCCTTCGTGGATTTTTTGGGACG<br>TGCTGTGGTCTTCCTAGTTGTTCTATAATCACTTCGTGA<br>CAGCCTGTTGGCTTACATTTATATTTATACGCTGTTATA<br>TTTATACGCTGTTAGACAAGTTTAAATACATAAATTTTT<br>ATTAGTCTATCGACCGTTAATTGCTTAACTTACCGAAGG<br>TTAATCGCTTTACTTACCGAAGGTTAATCGCTTTACTTA<br>CCGAAGGTTAATTGCTTAACTTACTGAAGGTTAATCGC<br>TTTACTTACCGAAGGTTAATTGCTTAACTTACTGAAGGT<br>TAATCGCTTTACTTACCGAAGGTTAATCCAATTTTTTTT<br>CGCCATATGTAGACGTTTAATTGCTACAACGTTATTAGC<br>CTTTCGTCGCTATCAAATCGGTTCAGATATATATCACTT<br>TATTCACTTTCGTTTATATTATGGCTGGATTAGGTCTTTT<br>AGTTAATTAAAATTTACATATTTAATGCTATTTATTATTA<br>TTGCAATTGCATTAAATATTTTTTTAAAAAAAATTAATC<br>TTCAGCTATATTAGTAAATAACCCATAAATAGTTTCAAT<br>TGGAATAATTGGAATTGGATATGGACTAGTTTTATTTTC<br>TTCTAATAACTTTAATATCGCTGGA
**pME_Cp_0_6_009_rbcl_3'Hom_Cr**	3'Homology	CAACGAACACGGCTACCTTCAGTAATTTTTAAACCATCA<br>CCTAATAATACCGCACCTACGTTGTTTGCTTCTAAGTTA<br>AGTGCAATACCTAAAGTACCATCTTCGAATTCAAGTAA<br>TTCACCTGACATTGCTTTTTCTAAACCATAAATACGAGC<br>AATACCGTCACCTACTTGGAAAACGATACCGAAATCTA<br>CCATTTTCACTTCTGGAGTGTATTGTTCAATTAAATCTTT<br>AATAAGATTACTAAGTTCTTCTGGAGTACGCATTGCCAT<br>AAAAAAGAAAAAATAAATAAAAGATTAAAAAAGTTTAT<br>TTTTAAAATCTTTCTCGAGAATTTTAAATAAGTTTAAAA<br>TTCAACAAAAATAGTGAGTGGTAAGATCACTTGTTAAC<br>AAAAGTAATGGTTCACCCTTGTCATATTTAAATACTAAA<br>ATTCATTTGCCCGAAGAGGACAAATTTATTTATTGCATT<br>AAAATCCCTAAGTTTACTTGCCCGTAAGGGGAAGGGG<br>GGGACGTCCACAGGCGTCGTAAGCAACTAAAGTTTAT<br>GACGCCGATTGCTTTGTTAGGAAAATATAAATATCCCA<br>TAAGAAAAGGTCCTTTAAAGGTTTTATGGACTAAATAA<br>AAAAGATAGCATAAGCATTAAAATCATGCAAATTAAAA<br>AAAAGGTAAATGTATTTATAAAAAGGTAAATGTATTTA<br>TATAGTATTTATATTATAGCATAATAATAAATATATTTA<br>TAAATTGATTGTTCTTAGAGCTAAAAGAGAAGAACAAT<br>GGGTTTATAGGTATTTTGAGATCCAGTTATAAAAATGA<br>CTTTTGACGTTTATGGTATATAAACACTGCCTCTAATAA<br>AGTCTACTATATTGGAGAGGAGTGAACAGTGGCCTCG<br>CTTATCCCCTGACAGGAATATACATG

**pME_Cp_0_6_013_petB_3'Hom_Cr**	3'Homology	CTTGTCGAAATAAATAGCACACTCTTGCATATCTATATT<br>TTAAGATGTCAGCTTTTTGATTCATTGAGTTGTATATTT<br>TATTTACTTTACTAGGGTGTTTATATATTCCTTTTTGGG<br>GTTGCCCAGATAGTTATATAACCAATCACAACAACAGT<br>AAAAATTGAAGTTTATTTACCCAAAGGGGTGTATCCCC<br>TTTGGGTAAATAAACTTCAATGGCCAACTGCCTTGGAA<br>ACTTACGTAGCAGCCTAAAAAAAGCTAGTCTTATATCC<br>GAAGCAAGTAAAAGAAATGGATATTAAATATTATAGA<br>GGACCTGAAATACCTTGTTTACGAATCATTAAGAAGTG<br>CATTAACATGAAAACAGCTGTTAAAAGTGGTAATACGA<br>AAGTGTGTAAACTGTAGAAACGTGTTAAAGTTGCTTGA<br>CCAACACCAACACCACCACGTAATAACTCAACAATGAA<br>ACCACCAACACCTGGGATTGCATCAGGAACACCTGTTA<br>CAATTTTAACCGCCCAGTAACCAACTTGGTCCCATGGTA<br>ATGAATAACCTGTTACACCAAAAGAAACTGTACATACA<br>GCCATGATTACACCTGTAACCCATGTTAATTCACGTGG<br>ACGTTTGAAACCACCTGTTAAATATACACGGAAAACGT<br>GTAAAACCATCATAAGAACCATCATACTAGCTGACCAA<br>CGGTGAATTGAACGAATTAACCAACCAAAGTTAACATC<br>AGTCATAATGTATTGTACTGATGCGAAAGCTTCTGCTA<br>CTGTTGGACGGTAGTAGAAAGTCATAGCAAAACCAGT<br>AGCTACTTGCACAAGGAAACATGTAAAAGTAATACCAC<br>CAATACAGTAGAAAATATTTACGTGTGGTGGAACATAT<br>TTACTTGTAATATCATCAGCAATTGCTTGAATTTCTAAA<br>CGTTCTTCAAACCAATCGTATACTTTACTCATATAAAAT<br>TTTTATAAGATTGTGACATGACCATTAGGCTTTCTTAAG<br>ACTAAAAAAATGTGTAGCTTAATTATTTAAAGTTTAAAT<br>TAAAGTTATAATATATATTATATATAAAATAAAAAAAAC<br>GTTAGTAATTCAAAAGTTTTAATATTATACAATTGAACT<br>ATTATGTATTAAATATAAGAATGTCACCTCTTACCATAT<br>TTCTATACTCCAAAGTAACTTTTTACATAAATGTCCCCTC<br>TGGGGCTGCCTCCTTCCCCTTCCCCTTCGGGTATATAAA<br>TATAGGGCAAGTAAACTTAGCATAAACTTTAGTTGCCC<br>GAAGGGGTTTACATACTCCGAAGGAGGACAAATTTATT<br>TATTGTGGTACAATAAATAAATTGTATGTAAACCCCTTT<br>CGGGTAACTAAAGTTTATCACGGCAATAAGTTTCTGCT<br>TACGCAGTATTATATCTGACGCAGTATTATATAAGAAG<br>TTGGCAGGATAAAAATGTGTAAGTATGGCAATCTTTTA<br>AAATAGTGTTCAATTCATTTAAGGCAGATAAAAAGAAA<br>AAAGTCCACAGGATTTAATTTTGAATAGTTCTCTATCAA<br>AAAAAGGTTTGCCGAACAATGTTTTTATTCCTGGAGTTT<br>GATTTTATGAAATTAGCTGTTTACGGAAAAGGTGGTAT<br>TGGAAAATCAACGACAAGTTGTAATATTTCGATTGCTTT<br>AGCAAAACGTGGTAAAAAAGTGTTACAAATTGGTTGT<br>GATCCTAAACATGATAGTACTTTTACATTGACAGGGTTT<br>TTAATTCCAACCATTATTGATACATTAAGTTCTAAAGAT<br>TATCATTATGAAGATATTTGGCC

**pME_Cp_0_6_019_psbD_3'Hom_Cr**	3'Homology	CTAAATCTCCAATTTGCAATTTAGAGATATAATTAAAAC<br>TATAAAGTTATTTAAGTTAATTTGTAATCAAATCCAACA<br>CAAAAATGTTTTTATATAGTTAACATGTTAAATTTAACA<br>TATGTTAAACAACTAAAATTCTGTAACAGAGAACAATA<br>AAATAAATGCTAGATTTTGTGTAATGCCGAAGTATATTT<br>ATATACTTCCCTTTCAAAAAAATAAATACTCTTGCCACT<br>AAAATTCATTTGCCTAGGACGTCCCCTTCCCCTTACGGG<br>ATGTTTATATACTAGGACGTCCCCTTCCCCTTACGGGAT<br>ATTTATATACTCCGAAGGACGTCCCCTTCGGGCAAATA<br>AATTTTAGTGGCAGTTGCCTGCCAACTGCCTAGGCAAG<br>TAAACTTAGGGATTTTAATGCAATAAATAAATTTGTCCC<br>CTTACGGGACGTCAGTGGCAGTTGCCTGCCAACTGCCT<br>AATATAAATATTAGTGGATATTTATATACTCCGAAGGA<br>GGCAGTTACCTGCCAACTGCCGAGGCAAATAAATTTTA<br>GTGGCAGTGGTACCGCCACTGCCTGCTCCCTCCTTCCCC<br>TTCGGGCAAGTAAACTTAGCATGTTTACATACTCCGAA<br>GGAGGACAAATTTATTTATTGTGGTACAATAAATAAGT<br>GGTACAATAAATAAATTGTATGTAAACCCCTTCCCCTTC<br>GGGACGTCCCCTTACGGGAATATAAATATTAGTGGCAG<br>TTGCCTGCCAACAAATTTATTTATTGTATTAACATAGGC<br>AGTGGCGGTACCACTGCCACTGGCGTCCTAATATAAAT<br>ATTGGGCAACTAAAGTTTATCGCAGTATTAACATAGGC<br>AGTGGCGGTACCACTGCCACTGGCGTCCTCCTTCGGAG<br>TATGTAAACCTGCTACCGCAGCAAATAAATTTTATTCTA<br>TTTTAATACTACAATATTTAGATTCCCGTTAGGGGATAG<br>GCCAGGCAATTGTCACTGGCGTCATAGTATATCAATAT<br>TGTAACAGATTGACACCCTTTAAGTAAACATTTTTTTTA<br>GGATTCATATGAAATTAAATGGATATTTGGTACATTTA<br>ATTCCACAAAAATGTCCAATACTTAAAATACAAAATTAA<br>AAGTATTAGTTGTAAACTTGACTAACATTTTAAATTTTA<br>AATTTTTTCCTAATTATATATTTTACTTGCAAAATTTATA<br>AAAATTTTATGCATTTTTATATCATAATAATAAAACCTTT<br>ATTCATGGTTTATAATATAATAATTGTG
**pME_Cp_0_6_020_psbA_5'Hom_-_reversed_Cr**	5'Homology Reversed	CGTATCCATGCTAGCAATATCTGATGGTACTTGCATTTC<br>ATAAGTTTGGCCTGGAATAACCACCGTTTCGGAAGTAC<br>CTGTCGCTTTAAGTTTTATAGCTAAATCTAAAGTTTCTTT<br>AAGTCTTTTAGCTGTATTAAATACTCCACGACTTTCCCTT<br>ACGGGACAATAAATAAATTTGTCCCCTTCCCCTTACGG<br>GACGTCAGTGGCAGTTGCCTGCCAACTGCCTCCTTCGG<br>AGTATTAAAATCCTATATTTATATACTCCTAAGTTTACTT<br>GCCCAATATTTATATTAGGCAGTTGGCAGGCAACTGCC<br>ACTGACGTCCCGAAGGGGAAGGGGAAGGACGTCCCCT<br>TCGGGTAAATAAATTTTAGTGGCAGTGGTACCACCACT<br>GCCTGCTTCCTCCTTCCCCTTCGGGCAAGTAAACTTAGA<br>ATAAAATTTATTTGCTGCGCTAGCAGGTTTACATACTCC<br>TAAGTTTACTTGCCCGAAGGGGAAGGAGGACGTCCCC<br>TTACGGGAATATAAATATTAGTGGCAGTGGTACAATAA<br>ATAAATTGTATGTAAACCCCTTCGGGCAACTAAAGTTT<br>ATCGCAGTATATAAATATAGAATGTTTACATACTCCGA<br>AGGAGGACGCCAGTGGCAGTGGTACCGCCACTGCCTG<br>CTCCGCAGTATTAACATCCTATTTTAATACTCCGAAGGA<br>GGCAGTTGGCAGGCAACTGCCACTAATATTTATATTCC<br>CGTAAGGGGACGTCCTATTTTAATACTCCGAAGGAGGC<br>AGTTGGCAGGCAACTGCCACTAAAATTTATTTGCCTCCT<br>AACGGAGCATTAAAATCCCGAAGGGGACGTCCCGAAG<br>GGGAAGGGGAAGGAGGCAACTGCCTGCTTCCTCCTTC<br>CCCTTCGGGCAAGTAAACTTAGAATAAAATTTATTTGCT<br>GCGCTAGCAGGTTTACATACTCCTAAGTTTACTTGCCCG<br>AAGGGGAAGGAGGACGTCCCCTTACGGGAATATAAAT<br>ATTAGTGGCAGTGGTACAATAAATAAATTGTATGTAAA<br>CCCCTTCGGGCAACTAAAGTTTATCGCAGTATATAAAT<br>ATCGGCAGTTGGCAGGCAACTGCCACTAAAATTCATTT<br>GCCCGAAGGGGACGTCCACTAATATTTATATTCCCGTA

		AGGGGACGTCCCGAAGGGGAAGGGGACGTCCTAACG<br>GAGCATTAAAATCCCTAAGTTTACTTGCCTAGGCAGTT<br>GGCAGGATATTTATATACGATATTAATACTTTGCAACTG<br>CCACTAAAATTTATTTGCCCGAAGGGGACGTCCTTCGG<br>AGTATATAAATATCCCGTAAGGGGAAGGGGATGTCCC<br>GTAAGGGGAAGGGGAGAAGCTCCAACAGAGG
**pME_Cp_0_6_021_petB_5'Hom-reversed_Cr**	5'Homology Reversed	GCGGTTTCTGCCAGATCCCCCGTTGGCCCATTGATAAG<br>GGGTAGCTATAAATTAACCATAGTTATATTCTGGTTAA<br>AGGATCGGAACTAACCCCAAGTCTCTAGTCTAAACAAA<br>AAATTGTGTATGCATTTAACACATTTAGTGTTTTTAACT<br>AGACAAAAAAAAATTAAGTATGATATTATAAAAGTAATA<br>TTTTTTAGCCTTCGTGATGGAACTGGTAGACATCCTGGT<br>TTTAGGAACCAGTGCTGAAAGGCGTGCCGGTTCAAATC<br>CGGCCGAAGGCATTTTAAGTTTAACGTAGAGCCAATAT<br>TTGTTTGAATTTATCTATTTTTTAAACCATTTTGGTTTAA<br>AATTTTTATTTGCTTCAAAGGAGCCTGTAAACGGTACTT<br>TAATTTTTACAGTAGCACTCGCAGAGCTTATTTACGTGC<br>AAATAAAAGCTCTATCTACTAGGATATTAGACTAGTATT<br>AATAAAACACAACATTTTATTAACAAAGTAATTTATGCC<br>GATTGGAGTACCTCGTATTATTTATTGTTGGGGTGAAG<br>AATTACCTGCACAGTGGACTGATATTTATAACTTTATTT<br>TCCGTCGTCGTATGGTATTTTTAATGCAATATTTAGATG<br>ATGAATTATGTAACCAAATTTGTGGTTTATTAATTAATA<br>TCCATATGGAAGATCGTTCTAAAGAGTTGGAAAAAAAA<br>GAAATGGAAAAAAGTGGATTATTTAAAAGTGGCACCG<br>CAAAAACAAAAGGAAAAGATACAGTAAAAAAAGAAAA<br>TTTATCTGGCGGCGCTAGTGCAAAACGTCAATC

**pME_Cp_0_6_022_rbcl_5'Hom-reversed_Cr**	5'Homology Reversed	GTAAGTAAACTGCGTAAGACGACCGACATATACCTAAA<br>GGCCCTTTCTATGCTCGACTGATAAGACAAGTACATAA<br>ATTTGCTAGTTTACATTATTTTTTATTTCTAAATATATAA<br>TATATTTAAATGTATTTAAAATTTTTCAACAATTTTTAAA<br>TTATATTTCCGGACAGATTATTTTAGGATCGTCAAAAGA<br>AGTTACATTTATTTATATAAATGGTTCCACAAACAGAAA<br>CTAAAGCAGGTGCTGGATTCAAAGCCGGTGTAAAAGA<br>CTACCGTTTAACATACTACACACCTGATTACGTAGTAAG<br>AGATACTGATATTTTAGCTGCATTCCGTATGACTCCACA<br>ACCAGGTGTTCCACCTGAAGAATGTGGTGCTGCTGTAG<br>CTGCTGAATCTTCAACAGGTACATGGACTACAGTATGG<br>ACTGACGGTTTAACAAGTCTTGACCGTTACAAAGGTCG<br>TTGTTACGATATCGAACCAGTTCCGGGTGAAGACAACC<br>AATACATTGCTTACGTAGCTTACCCAATCGACTTATTCG<br>AAGAAGGTTCAGTAACTAACATGTTCACTTCTATTGTA<br>GGTAACGTATTCGGTTTCAAAGCTTTACGTGCTCTACGT<br>CTTGAAGACCTTCGTATTCCACCTGCTTACGTTAAAACA<br>TTCGTAGGTCCTCCACACGGTATTCAGGTAGAACGTGA<br>CAAATTAAACAAATATGGTCGTGGTCTTTTAGGTTGTA<br>CAATCAAACCTAAATTAGGTCTTTCAGCTAAAAACTAC<br>GGTCGTGCAGTTTATGAATGTTTACGTGGTGGTCTTGA<br>CTTTACTAAAGACGACGAAAACGTAAACTCACAACCAT<br>TCATGCGTTGGCGTGACCGTTTCCTTTTCGTTGCTGAAG<br>CTATTTACAAAGCTCAAGCAGAAACAGGTGAAGTTAAA<br>GGTCACTACTTAAACGCTACTGCTGGTACTTGTGAAGA<br>AATGATGAAACGTGCAGTATGTGCTAAAGAATTAGGT<br>GTACCTATTATTATGCACGACTACTTAACAGGTGGTTTC<br>ACAGCTAACACTTCATTAGCTATCTACTGTCGTGACAAC<br>GGTCTTCTTCTACACATCCACCGTGCTATGCACGCGGTT<br>ATTGACCGTCAACGTAACCACGGTATTCACTTCCGTGTT<br>CTTGCTAAAGCTCTTCGTATGTCTGGTGGTGACCACCTT<br>CACTCTGGTACTGTTGTAGGTAAACTAGAAGGTGAACG<br>TGAAGTTACTCTAGGTTTCGTAGACTTAATGCGTGATG<br>ACTACGTTGAAAAAGACCGTAGCCGTGGTATTTACTTC<br>ACTCAAGACTGGTGTTCAATGCCAGGTGTTATGCCAGT<br>TGCTTCAGGTGGTATTCACGTATGGCACATGCCAGCTT<br>TAGTTGAAATCTTCGGTGATGACGCATGTCTTCAGTTC<br>GGTGGTGGTACTCTAGGTCACCCTTGGGGTAACGCTCC<br>AGGTGCTGCAGCTAACCGTGTAGCTCTTGAAGCTTGTA<br>CTCAAGCTCGTAACGAAGGTCGTGACCTTGCTCGTGAA<br>GGTGGCGACGTAATTCGTTCAGCTTGTAAATGGTCACC<br>AGAACTTGCTGCTGCATGTGAAGTTTGGAAAGAAATTA<br>AATTCGAATTTGATACTATTGACAAACTTTAATTTTTATT<br>TTTCATGATGTTTATGTGAATAGCATAAACATCGTTTTT<br>ATTTTTATGGTGTTTAGGTTAAATACCTAAACATCATTT<br>TACATTTTTAAAATTAAGTTCTAAAGTTATCTTTTGTTTA<br>AATTTGCCTGTCTTTATAAATTACGATGTGCCAGAAAAA<br>TAAAATCTTAGCTTTTTATTATAGAATTTATCTTTATGTA<br>TTATATTTTATAAGTTATAATAAAAGAAATAGTAACATA<br>CTAAAGCGGATGTAACTCAATCGGTAGAGTGCGATCCT<br>TCCAAGTTCGAGGTTGTGGGTTCGAGTCCCATCATCCG<br>CTAAACCAATCTATAAAAGTTGTTGAATATGCTGAAAT<br>GTTTTCAAAGAAAAAGCCTAGTTTTTCTTTTACAACAAG<br>CAAAGAACAATTGGCATTCTTTGATTGTAAGAAAATGC<br>GCTTACGTGGAGTTATGTGTTTTTAC

**pME_Cp_0_6_023_psbD_5'Hom-reversed_Cr**	5'Homology Reversed	CACAAAGCAGTTCTAGTCCCATATATATAACTATATATA<br>ACCCGTTTAAAGATTTATTTAAAAATATGTGTGTAAAAA<br>ATGCTTATTTTTAATTTTATTTTATATAAGTTATAATATT<br>AAATACACAATGATTAAAATTAAATAATAATAAATTTAA<br>CGTAACGATGAGTTGTTTTTTTATTTTGGAGATACACGC<br>AATGACAATTGCGATCGGTACATATCAAGAGAAACGC<br>ACATGGTTCGATGACGCTGATGACTGGCTTCGTCAAGA<br>CCGTTTCGTATTCGTAGGTTGGTCAGGTTTATTACTATT<br>CCCTTGTGCTTACTTTGCATTAGGTGGTTGGTTAACTGG<br>TACTACTTTCGTTACTTCATGGTATACGCATGGTTTAGC<br>TACTTCTTACTTAGAAGGTTGTAACTTCTTAACAGCAGC<br>TGTTTCTACACCTGCTAACAGTATGGCTCACTCTCTTCT<br>ATTTGTTTGGGGTCCAGAAGCTCAAGGTGATTTCACTC<br>GTTGGTGTCAACTTGGTGGTTTATGGGCATTCGTTGCT<br>TTACACGGTGCATTTGGTTTAATTGGTTTCATGCTTCGT<br>CAGTTTGAAATTGCTCGTTCAGTAAACTTACGTCCATAC<br>AACGCAATTGCTTTCTCAGCACCAATTGCTGTATTCGTT<br>TCAGTATTCCTAATTTACCCATTAGGTCAATCAGGTTGG<br>TTCTTTGCACCTAGTTTCGGTGTAGCTGCTATCTTCCGT<br>TTCATTTTATTCTTCCAAGGTTTCCACAACTGGACACTTA<br>ACCCATTCCACATGATGGGTGTTGCTGGTGTTTTAGGT<br>GCTGCTTTATTATGTGCTATTCACGGTGCTACTGTTGAA<br>AACACATTATTCGAAGACGGTGACGGTGCTAACACATT<br>CCGTGCATTCAACCCTACACAGGCTGAAGAAACATACT<br>CTATGGTTACTGCTAACCGTTTCTGGTCACAAATCTTCG<br>GTGTTGCTTTCTCTAACAAACGTTGGCTTCACTTCTTCA<br>TGTTATTAGTTCCAGTAACTGGTCTTTGGATGAGTGCTA<br>TTGGTGTTGTAGGTTTAGCTCTAAACTTACGTGCTTACG<br>ACTTCGTATCACAAGAGATTCGTGCTGCTGAAGACCCT<br>GAATTCGAAACATTCTACACTAAAAACATTCTTCTTAAC<br>GAAGGTATTCGTGCTTGGATGGCTGCTCAAGACCAACC<br>ACACGAACGTTTAGTATTCCCTGAAGAAGTATTACCAC<br>GTGGTAACGCTCTATAATATATTTTTATATAAATTACCA<br>ATACTAATTAGTATTGGTAATTTATATTACTTTATTATTT<br>AAAAGAAAATGCCCCTTTGGGGCTAAAAATCACATGA<br>GTGCTTGAGCCGTATGCGAAAAAACTCGCATGTACGGT<br>TCTTTAGGAGGATTTAAAATATTAAAAAATAAAAAAAC<br>AAATCCTACCTGACTAAACCAGGACATTTTTCACGTACT<br>CTGTCAAAAGGTCCAAACACAACAACTTGGATTTGGAA<br>CCTTCACGCAGATGCTCATGACTTTGACAGTCATACAA<br>GTGATCTAGAAGAAATTTCTAGAAAAGTATTCAGTGCA<br>CACTTTGGTCAATTAGGTATCATTTTCATTTGGTTAAGT<br>GGGTGCGACACGAAGACGTATATATTTTTATAGTTTAA<br>AAAGATACTTTTACACTGTAGTTGAAAAGTATAAGCAC<br>TTTTAAAAATCAAAGCAGTATAAGGCAATTATTTGCATT<br>TTGCTTTAGTTCTTTTTATTTTTTCAGAAAAACTTACTTT<br>TTAGTTTCAATTATGTCTAAAAAGACAGAAGTGGTTTAT<br>CATATAAAAAGATTTTTACAATCCACTTTTTTAAAAATC<br>TTTTGGTTTTTTATTAAAATCTATACTCTTAATACAAATG<br>GCAGATCCTAAATCCTTGTAAGACATCCCCTTAAGGGA<br>CGGTTTTATCAAGTTCCATAGCCTAACAGAGGTAAATC<br>CATTTTAATAACCACAAAGCGTTTATCGTTAAAATAAAA<br>TACTAATATATGTAACGTGTAAAAATAATATTTTAATAT<br>TATTAAGGATACTCAAAAAAAATACATTAACAAGTCTA<br>ATAGAACACAAAATGTATTAAATCAGCTAGGTCTGAAA<br>AAACTTTAATTTATTTTTAAGGACATCTCTGCCTCTTTGG<br>GTATAGCAATTCAATTGATTTGCTCTATATTTATATCCCT<br>AAACTAGATAAACTTTGTAGAAACTATAAATTTTATGCA<br>GTTTATCCGAAATTAAAGTCAAACGTTTTTTTCTTTGAG<br>TGAATTCTTTTTTTCGATTTTTATTATTCCAAACC

Supplementary Table 6: List of primers used in this study.

Primer	Target	Sequence	Purpose
**oRI0025**	MoClo backbone	CGGTTCCTGGCCTTTTGC	Sequencing Lvl1 fw
**oRI0026**	MoClo backbone	GATAGGTGCCTCACTGATTAAG	Sequencing Lvl1 rv
**oRI0027**	MoClo backbone	CTATAAAAATAGGCGTATCACGAGGC	Sequencing Entry vector fwd
**oRI0028**	MoClo backbone	CGAGTCAGTGAGCGAGGAAGC	Sequencing Entry vector rv
**oRI0202**	MoClo backbone	GTGAGCTATTAACGCGTTTATCTTAAC	Sequencing Lvl2 fw
**oRI0203**	MoClo backbone	GTAAACTAAGCAACTCAACCATATTTATATTC	Sequencing Lvl2 rv
**oRI0343**	Nanoluciferase	CCAACCTGTAACACCATTAATTG	cPCR to confirm integration of Nanoluc rv
**oRI0642**	psbH locus	CTCTAGACGATGGGGGC	cPCR to confirm integration in psbH locus fw
**oRI0643**	psbN locus	GTTTATATGCTCGACAAAATGACTTTC	cPCR to confirm integration in psbN locus rv
**oRI0733**	Glycolate dehydrogenase	GGTCTCGTTGCGGGACGTCCTGCCAAC	cPCR to confirm Glycolate dehydrogenase integration fw
**oRI0734**	Glycolate dehydrogenase	GGTCTCGCGCTGCAGGCAACAAATTTATTTATTGTC	cPCR to confirm Glycolate dehydrogenase integration rv
**oRI0738**	Malate synthase	GGTCTCGAGCGGGGTAACTAAAGTTTATC	cPCR to confirm Malate synthase integration fw
**oRI0739**	Malate synthase	GGTCTCGCATTCGCTAATGGGAGAATATATTTATTTGAC	cPCR to confirm Malate synthase integration rv
**oRI0736**	Kanamycin	GGTCTCGGTTGATGCGAGTGATTTTGATG	cPCR to confirm Kanamycin integration fw
**oRI0737**	Kanamycin	GGTCTCGCAACCAAACCGTTATTCATTCG	cPCR to confirm Kanamycin integration rv
**oRI0745**	Tobramycin	CATTAAAGGGAAGTCATCGTC	cPCR to confirm Tobramycin integration fw
**oRI0746**	Tobramycin	CATTGAAAACGGTCGTAAAG	cPCR to confirm Tobramycin integration rv
**aadA_rv_57_54**	Spectinomycin	CAGATCAGTTGGAAGAATTTG	cPCR to confirm Spectinomycin integration rv
**aadA_rv_63_60**	Spectinomycin	CATACTTGAAGCTAGGCAGGC	cPCR to confirm Spectinomycin integration rv
**Nanoluc fwd_61_59**	Nanoluciferase	CCTGATGGTTCATTATTATTCCGTG	cPCR to confirm integration of Nanoluc fw
**Nanoluc_fwd_58_55**	Nanoluciferase	CAATTAATGGTGTTACAGGTTG	cPCR to confirm integration of Nanoluc fw
**Nanoluc_fwd_62_59**	Nanoluciferase	GTTACAGGTTGGCGTCTTTG	cPCR to confirm integration of Nanoluc fw
**petB_fwd_59_56**	petB locus	ACAGGTGATTTTCTTCAATACC	cPCR to confirm integration in petB locus for Homoplasmy fw
**petB_fwd_63_60**	petB locus	GTTCCAGTTTAACCATTCCGTTG	cPCR to confirm integration in petB locus for Homoplasmy fw
**petB_rv_57_55**	petB locus	ACCTCCGTAAATAACATCTTC	cPCR to confirm integration in petB locus for Homoplasmy rv
**petB_rv_60_57**	petB locus	CAATCTACACCCCCATAACC	cPCR to confirm integration in petB locus for Homoplasmy rv
**petB_rv_63_60**	petB locus	CACAATCTACACCCCCATAACC	cPCR to confirm integration in petB locus for Homoplasmy rv
**psbD_fwd_59_56**	psbD locus	AGGCGATTTGTTTCACTTAAG	cPCR to confirm integration in psbD locus for Homoplasmy fw
**psbD_fwd_62_59**	psbD locus	CCGTGATACATCTTCGAAAGTAAG	cPCR to confirm integration in psbD locus for Homoplasmy fw
**psbD_rv_57_54**	psbD locus	CTTGTTTTTCGGGTATTGAG	cPCR to confirm integration in psbD locus for Homoplasmy rv
**psbD_rv_63_60**	psbD locus	TTCCCGATACTTTGTCTGCTTG	cPCR to confirm integration in psbD locus for Homoplasmy rv

**psbH_cPCR_hom_fwd_2**	psbH locus	CTTTAAGTGGATCTCTCGTC	cPCR to confirm integration in psbH locus for Homoplasmy fw
**psbH_cPCR_Hom_fwd_3**	psbH locus	AAGTGGATCTCTCGTCAG	cPCR to confirm integration in psbH locus for Homoplasmy fw
**psbH_cPCR_homology_fwd**	psbH locus	GAAAAGTGAGCTATTAACGC	cPCR to confirm integration in psbH locus for Homoplasmy fw
**psbH_fwd_60_57**	psbH locus	CATTGCGAAAATACTGGTGC	cPCR to confirm integration in psbH locus for Homoplasmy fw
**psbH_fwd_60_57_2**	psbH locus	GCTAGTCATTGCGAAAATACTG	cPCR to confirm integration in psbH locus for Homoplasmy fw
**psbH_rv_58_56**	psbH locus	GTTTATATGCTCGACAAAATGAC	cPCR to confirm integration in psbH locus for Homoplasmy rv
**psbH_rv_59_57**	psbH locus	ATGCTCGACAAAATGACTTTC	cPCR to confirm integration in psbH locus for Homoplasmy rv
**rbcl_fwd_57_55**	rbcL locus	GTCTAACCGATTTTATAATGCG	cPCR to confirm integration in rbcL locus for Homoplasmy fw
**rbcl_fwd_59_57**	rbcL locus	GTTAACAAGTTATTCCCACTGTG	cPCR to confirm integration in rbcL locus for Homoplasmy fw
**rbcl_rv_57_55**	rbcL locus	GTCAACTACACGACCTAAG	cPCR to confirm integration in rbcL locus for Homoplasmy rv
**rbcl_rv_60_57**	rbcL locus	CAGGAATTTCAGCGATTTTACC	cPCR to confirm integration in rbcL locus for Homoplasmy rv
**tobra_rv_56_58**	Tobramycin	GGTGACTCAGGTATTATTGATG	cPCR to confirm integration of Tobramycin rv
**tobra_rv_57_55**	Tobramycin	TTGGTGACTCAGGTATTATTG	cPCR to confirm integration of Tobramycin rv
**tobra_rv_58_56_2**	Tobramycin	CAAGAATTCATTGAAAACGGTC	cPCR to confirm integration of Tobramycin rv
**tobra_tmv_rv_59_56**	Tobramycin	CGCTTAAATAAATAACGGATTGTG	cPCR to confirm integration of Tobramycin rv
**petB_fwd_5'Hom**	petB locus	GTTAGTTCCGATCCTTTAACC	cPCR to confirm integration in petB locus for Homoplasmy fw
**psbD_fwd_5'Hom**	psbD locus	GTCATTGCGTGTATCTCC	cPCR to confirm integration in psbD locus for Homoplasmy fw
**psbH_rv_58_56**	psbH locus	GTTTATATGCTCGACAAAATGAC	cPCR to confirm integration in psbH locus for Homoplasmy fw
**rbcl_fwd_5'Hom**	rbcL locus	CTTTAGGTATATGTCGGTCG	cPCR to confirm integration in rbcL locus for Homoplasmy fw
**WendyI_fwd**	WendyI locus	GATCAGTTCAGTTGTGTTAG	cPCR to confirm the lack of integration in the WendyI locus fw
**WendyI_fwd2**	WendyI locus	TTTATGGCGTCTGAGTTG	cPCR to confirm the lack of integration in the WendyI locus fw
**WendyI_rv**	WendyI locus	GTAAACAGATAGGAATGGAC	cPCR to confirm the lack of integration in the WendyI locus rv
**WendyI_rv2**	WendyI locus	CTAACACAACTGAACTGATC	cPCR to confirm the lack of integration in the WendyI locus rv
**WendyII_fwd**	WendyII locus	CAAATGCGTTAAGACTTCC	cPCR to confirm the lack of integration in the WendyII locus fw
**WendyII_fwd_2**	WendyII locus	CCTTAAAGGGTGAATGTATATAC	cPCR to confirm the lack of integration in the WendyII locus fw
**WendyII_rv**	WendyII locus	CCAGTATTTTCGCAATGAC	cPCR to confirm the lack of integration in the WendyII locus rv
**WendyII_rv_2**	WendyII locus	GGAAGTCTTAACGCATTTG	cPCR to confirm the lack of integration in the WendyII locus rv
**HomPCR_Confirm_01**	psbH locus fwd	GAATTCGAATCCGCGTTTTC	cPCR to assess the homoplasmic state of engineered strains in psbH locus
**HomPCR_Confirm_02**	psbH locus rv	TAACTAGGGTTCTCAGAGGC	cPCR to assess the homoplasmic state of engineered strains in psbH locus
**HomPCR_Confirm_03**	psbH locus left flank fwd	GAATTCGAATCCGCGTTTTC	cPCR to confirm integration of the left flank of genetic constructs in psbH locus
**HomPCR_Confirm_04**	psbH locus left flank rv	GGCTTATCTTGGACAAGAAGAAG	cPCR to confirm integration of the left flank of genetic constructs in psbH locus
**HomPCR_Conf**	psbH locus	GTAGTGAAGGTCGTCATTCTAC	cPCR to confirm integration of the

**irm_05**	right flank mScarlet-I fw		right flank of the mScarlet-I construct in psbH locus
**HomPCR_Confirm_06**	psbH locus right flank mScarlet-I rv	TAACTAGGGTTCTCAGAGGC	cPCR to confirm integration of the right flank of the mScarlet-I construct in psbH locus
**HomPCR_Confirm_07**	psbH locus right flank Nanoluc fw	CATATATATATACTTAATAGCTACCATAGGC	cPCR to confirm integration of the right flank of the Nanoluc construct in psbH locus
**HomPCR_Confirm_08**	psbH locus right flank Nanoluc rv	TAACTAGGGTTCTCAGAGGC	cPCR to confirm integration of the right flank of the Nanoluc construct in psbH locus

Supplementary Table 7: List of Genbank Accession code per construct.

**Plasmid Name**	**Genbank Accession code**
**pME_Cp_UAV_sfGFP**	PX069915
**pME_G_E_004_UAV_mScarlet-I**	PX069916
**pME_G_E_007_UAV_sfGFP_PaqCI**	PX069914
**pME_Cp_0_1_001_psbH_5'Hom_Cr**	PX069917
**pME_Cp_0_1_004_psbA_5'Hom_Cr**	PX069918
**pME_Cp_0_1_005_rbcl_5'Hom_Cr**	PX069919
**pME_Cp_0_1_007_petB_5'Hom_Cr**	PX069920
**pME_Cp_0_1_010_psbD_5'Hom_Cr**	PX069921
**pME_G_0_1_003_5C1RLF**	PX069922
**pME_G_0_1_005_5C1RSF**	PX069923
**pME_G_0_1_006_5C1SR**	PX069924
**pME_G_0_1_007_5C2LF**	PX069925
**pME_G_0_1_008_5C2SF**	PX069926
**pME_G_0_1_009_5C2SR**	PX069927
**pME_G_0_1_010_5C3LF**	PX069928
**pME_G_0_1_011_5C3SF**	PX069929
**pME_G_0_1_012_5C3SR**	PX069930
**pME_G_0_1_013_5C5LF**	PX069931
**pME_G_0_1_014_5C5SF**	PX069932
**pME_G_0_1_015_5C5SR**	PX069933
**pME_G_0_1_016_5C7LF**	PX069934
**pME_G_0_1_017_5C7SF**	PX069935
**pME_G_0_1_019_5C7OSR**	PX069936
**pME_G_0_1_022_5C4SF**	PX069937
**pME_G_0_1_023_5C6SF**	PX069938
**pME_G_0_1-2_001_OC5'1**	PX171811
**pME_G_0_1-2_002_OC5'2**	PX171812
**pME_G_0_1-2_003_OC5'3**	PX171813
**pME_G_0_1-2_004_OC5'4**	PX171814
**pME_G_0_1-2_005_OC5'5**	PX171815

**pME_G_0_1-2_006_OC5'6**	PX171816
**pME_G_0_1-2_007_OC5'7**	PX171817
**pME_B_0_2_020_Pdummy**	PX171457
**pME_Cp_0_2_001_rrn16_prom_Cr**	PX171467
**pME_Cp_0_2_003_psaA2_prom_Cr**	PX171468
**pME_Cp_0_2_004_rrn16_prom_short_Cr**	PX171469
**pME_Cp_0_2_057_Synthetic_promoter_01**	PX171470
**pME_Cp_0_2_058_Synthetic_promoter_02**	PX171471
**pME_Cp_0_2_059_Synthetic_promoter_03**	PX171472
**pME_Cp_0_2_060_Synthetic_promoter_04**	PX171473
**pME_Cp_0_2_061_Synthetic_promoter_05**	PX171474
**pME_Cp_0_2_062_Synthetic_promoter_06**	PX171475
**pME_Cp_0_2_063_Synthetic_promoter_07**	PX171476
**pME_Cp_0_2_064_Synthetic_promoter_08**	PX171477
**pME_Cp_0_2_065_Synthetic_promoter_09**	PX171478
**pME_Cp_0_2_066_Synthetic_promoter_10**	PX171479
**pME_Cp_0_2_067_Synthetic_promoter_11**	PX171480
**pME_Cp_0_2_068_Synthetic_promoter_12**	PX171481
**pME_Cp_0_2_069_Synthetic_promoter_13**	PX171482
**pME_Cp_0_2_070_Synthetic_promoter_14**	PX171483
**pME_Cp_0_2_071_Synthetic_promoter_15**	PX171484
**pME_Cp_0_2_072_Synthetic_promoter_16**	PX171485
**pME_Cp_0_2_073_Synthetic_promoter_17**	PX171486
**pME_Cp_0_2_074_Synthetic_promoter_18**	PX171487
**pME_Cp_0_2_075_Synthetic_promoter_19**	PX171488
**pME_Cp_0_2_076_Synthetic_promoter_20**	PX171489
**pME_Cp_0_2_077_Synthetic_promoter_21**	PX171490
**pME_Cp_0_2_078_Synthetic_promoter_22**	PX171491
**pME_Cp_0_2_079_rbcL_promoter_Cr**	PX171492
**pME_Cp_0_2_080_tRNA_Glu2_promoter_Cr**	PX171493
**pME_Cp_0_2_081_WendyII_promoter__Cr**	PX171494
**pME_G_0_2_001_Promoter_placeholder**	PX171819
**pME_Cp_0_2-3_001_psbD_Prom_5UTR_Cr**	PX171460
**pME_Cp_0_2-3_002_rbcL_Prom_5UTR_Cr**	PX171461
**pME_Cp_0_2-3_003_atpA_Prom_5UTR_Cr**	PX171462
**pME_Cp_0_2-3_004_psbA_Prom_5UTR_Cr**	PX171463
**pME_G_0_2-3_005_Placeholder**	PX171818
**pME_Cp_0_2-3a_002_psbD_Prom_5'UTR_Cr**	PX171464
**pME_Cp_0_2-3a_003_rbcL_Prom_5'UTR_Cr**	PX171465
**pME_Cp_0_2-3a_004_atpA_Prom_5UTR_Cr**	PX171466
**pME_B_0_3_009_5'UTR_dummy**	PX171458
**pME_Cp_0_3_001_atpA_5'UTR_Cr**	PX171495
**pME_Cp_0_3_002_atpA_5'UTR_Cr**	PX171496
**pME_Cp_0_3_003_atpB_5'UTR_Cr**	PX171497

**pME_Cp_0_3_004_chlL_5'UTR_Cr**	PX171498
**pME_Cp_0_3_005_petB_5'UTR_Cr**	PX171499
**pME_Cp_0_3_006_psaA_5'UTR_Cr**	PX171500
**pME_Cp_0_3_007_psbA_5'UTR_Cr**	PX171501
**pME_Cp_0_3_008_psbE_5'UTR_Cr**	PX171502
**pME_Cp_0_3_009_psbF_5'UTR_Cr**	PX171503
**pME_Cp_0_3_010_psbK_5'UTR_Cr**	PX171504
**pME_Cp_0_3_011_petA_5'UTR_Cr**	PX171505
**pME_Cp_0_3_012_petD_5'UTR_Cr**	PX171506
**pME_Cp_0_3_013_psaB_5'UTR_Cr**	PX171507
**pME_Cp_0_3_014_psbB_5'UTR_Cr**	PX171508
**pME_Cp_0_3_015_psbC_5'UTR_Cr**	PX171509
**pME_Cp_0_3_016_gene10_5'UTR_T7**	PX171510
**pME_Cp_0_3_017_ccsA_5'UTR_Cr**	PX171511
**pME_Cp_0_3_018_psaC_5'UTR_Cr**	PX171512
**pME_Cp_0_3_019_rbcL_5'UTR_Cr**	PX171513
**pME_Cp_0_3_020_rpoC1_5'UTR_Cr**	PX171514
**pME_Cp_0_3_021_rpoC2_5'UTR_Cr**	PX171515
**pME_Cp_0_3_023_tufA_5'UTR_Cr**	PX171516
**pME_Cp_0_3_024_clpP_5'UTR_Cr**	PX171517
**pME_Cp_0_3_025_rpl23_5'UTR_Cr**	PX171518
**pME_Cp_0_3_026_rpl16_5'UTR_Cr**	PX171519
**pME_Cp_0_3_027_rpoB1_5'UTR_Cr**	PX171520
**pME_Cp_0_3_030_atpH_5'UTR_Cr**	PX171521
**pME_Cp_0_3_031_psbD_5'UTR_Cr**	PX171522
**pME_Cp_0_3_032_rbcL_5'UTR-full_Nt**	PX171523
**pME_Cp_0_3_033_Synthetic_5'UTR_1_Sy**	PX171524
**pME_Cp_0_3_039_atpH_5'UTR_Nt**	PX171525
**pME_Cp_0_3_061_gene10v2_5'UTR_T7**	PX171526
**pME_Cp_0_3_064_psbA_5'UTR_Nt**	PX171527
**pME_Cp_0_3_065_psbC_5'UTR_Nt**	PX171528
**pME_Cp_0_3_091_Synthetic_5'UTR_2_Sy**	PX171529
**pME_Cp_0_3_092_Synthetic_5'UTR_3_Sy**	PX171530
**pME_Cp_0_3_093_Synthetic_5'UTR_4_Sy**	PX171531
**pME_Cp_0_3_094_Synthetic_5'UTR_5_Sy**	PX171532
**pME_G_0_3_001_5'UTR_Placeholder**	PX171820
**pME_Cp_0_3a_001_gene10_5'UTR_T7**	PX171533
**pME_Cp_0_3a_002_petA_5'UTR_Cr**	PX171534
**pME_Cp_0_3a_003_psaA_5'UTR_Cr**	PX171535
**pME_Cp_0_3a_004_psbE_5'UTR_Cr**	PX171536
**pME_Cp_0_3a_005_rbcl_5'UTR_Cr**	PX171537
**pME_Cp_0_3a_006_atpE-ycf12_IEE_Cr**	PX171538
**pME_Cp_0_3a_007_rps14-rps7_IEE_Cr**	PX171539
**pME_Cp_0_3a_008_psbE-rps9_IEE_Cr**	PX171540

**pME_Cp_0_3a_009_ycf4-ycf3_IEE_Cr**	PX171541
**pME_Cp_0_3a_010_tscA-chlN_IEE_Cr**	PX171542
**pME_Cp_0_3a_011_psbC_5'UTR_Cr**	PX171543
**pME_G_0_3a_001_Placeholder**	PX171821
**pME_Cp_0_3b_024_His-Tag**	PX171544
**pME_Cp_0_3b_025_Hibit-tag**	PX171545
**pME_Cp_0_3b_026_Flag-tag**	PX171546
**pME_Cp_0_3b_027_HA-tag**	PX171547
**pME_Cp_0_3b_028_myc-tag**	PX171548
**pME_Cp_0_3b_029_mScarlet-I**	PX171549
**pME_Cp_0_3b_030_mCerulean**	PX171550
**pME_Cp_0_3b_031_mVenus**	PX171551
**pME_G_0_3b_0002_Placeholder**	PX171822
**pME_Cp_0_3IEE_001_atpE-ycf12_IEE_Cr**	PX171552
**pME_Cp_0_3IEE_003_chlL-rpl36_IEE_Cr**	PX171553
**pME_Cp_0_3IEE_004_petG-rps3_IEE_Cr**	PX171554
**pME_Cp_0_3IEE_005_psaC-petL_IEE_Cr**	PX171555
**pME_Cp_0_3IEE_007_psbB-psbT_IEE_Cr**	PX171556
**pME_Cp_0_3IEE_008_psbE-rps9_IEE_Cr**	PX171557
**pME_Cp_0_3IEE_009_psbF-psbL_IEE_Cr**	PX171558
**pME_Cp_0_3IEE_010_psbJ-atpI_IEE_Cr**	PX171559
**pME_Cp_0_3IEE_013_rpl2-rps19_IEE_Cr**	PX171560
**pME_Cp_0_3IEE_015_rpl23-rpl2_IEE_Cr**	PX171561
**pME_Cp_0_3IEE_018_rps14-rps7_IEE_Cr**	PX171562
**pME_Cp_0_3IEE_019_rps18-rps2_IEE_Cr**	PX171563
**pME_Cp_0_3IEE_020_trnF-psaC_IEE_Cr**	PX171564
**pME_Cp_0_3IEE_021_ycf3-rps18_IEE_Cr**	PX171565
**pME_Cp_0_3IEE_022_ycf4-ycf3_IEE_Cr**	PX171566
**pME_Cp_0_3IEE_023_tscA-chlN_IEE5_Cr**	PX171567
**pME_Cp_0_4_001_Nanoluc**	PX171568
**pME_Cp_0_4_005_aadA**	PX171569
**pME_Cp_0_4_006_mScarlet**	PX171570
**pME_Cp_0_4_007_mScarlet-I**	PX171571
**pME_Cp_0_4_008_tdTomato**	PX171572
**pME_Cp_0_4_009_mVenus**	PX171573
**pME_Cp_0_4_010_mCerulean3**	PX171574
**pME_Cp_0_4_011_iLov**	PX171575
**pME_Cp_0_4_012_FlucS284T**	PX171576
**pME_Cp_0_4_022_aac6-aph2-marker**	PX171577
**pME_Cp_0_4_023_ptxD-marker**	PX171578
**pME_Cp_0_4_024_CrCD-negative-marker**	PX171579
**pME_Cp_0_4_026_Arg9-marker**	PX171580
**pME_Cp_0_4_033_Firefly-luciferase_v2**	PX171581
**pME_Cp_0_4_034_aadA**	PX171582

**pME_Cp_0_4_035_aphA6**	PX171583
**pME_G_0_4_001_CDS_Placeholder**	PX171823
**pME_B_0_5_012_3'UTR_dummy**	PX171459
**pME_Cp_0_5_001_psbA_3'UTR_Cr**	PX171584
**pME_Cp_0_5_005_rrnB_3'UTR_Ec**	PX171585
**pME_Cp_0_5_006_psbA_3'UTR_Nt**	PX171586
**pME_Cp_0_5_007_atpA_3'UTR_Cr**	PX171587
**pME_Cp_0_5_009_rbcl_3'UTR_rbcl-CDS_Cr**	PX171588
**pME_Cp_0_5_010_atpB_3'UTR_Cr**	PX171589
**pME_Cp_0_5_011_chlB_3'UTR_Cr**	PX171590
**pME_Cp_0_5_012_clpP_3'UTR_Cr**	PX171591
**pME_Cp_0_5_013_ftsH_3'UTR_Cr**	PX171592
**pME_Cp_0_5_015_petA_3'UTR_Cr**	PX171593
**pME_Cp_0_5_017_petD_3'UTR_Cr**	PX171594
**pME_Cp_0_5_018_petL_3'UTR_Cr**	PX171595
**pME_Cp_0_5_019_psaB_3'UTR_Cr**	PX171596
**pME_Cp_0_5_020_psaC_3'UTR_Cr**	PX171597
**pME_Cp_0_5_021_psbA_3'UTR_Cr**	PX171598
**pME_Cp_0_5_022_psbC_3'UTR_Cr**	PX171599
**pME_Cp_0_5_026_rbcL_3'UTR_Cr**	PX171600
**pME_Cp_0_5_028_rpl36_3'UTR_Cr**	PX171601
**pME_Cp_0_5_030_rpoB2_3'UTR_Cr**	PX171602
**pME_Cp_0_5_031_rpoC2_3'UTR_Cr**	PX171603
**pME_Cp_0_5_032_rps2_3'UTR_Cr**	PX171604
**pME_Cp_0_5_034_rps4_3'UTR_Cr**	PX171605
**pME_Cp_0_5_035_rps8_3'UTR_Cr**	PX171606
**pME_Cp_0_5_036_rps11_3'UTR_Cr**	PX171607
**pME_Cp_0_5_038_rps19_3'UTR_Cr**	PX171608
**pME_Cp_0_5_039_tufA_3'UTR_Cr**	PX171609
**pME_Cp_0_5_040_ycf1_3'UTR_Cr**	PX171610
**pME_Cp_0_5_041_ycf12_3'UTR_Cr**	PX171611
**pME_Cp_0_5_042_rbcL_3'UTR_Nt**	PX171612
**pME_Cp_0_5_043_BMV_3'UTR_Bv**	PX171613
**pME_Cp_0_5_044_TYMV_3'UTR_Tv**	PX171614
**pME_Cp_0_5_045_rpoA_3'UTR_Nt**	PX171615
**pME_Cp_0_5_046_petD_3'UTR_Nt**	PX171616
**pME_Cp_0_5_047_TMV_3'UTR_Tv**	PX171617
**pME_Cp_0_5_048_psaC_3'UTR_Nt**	PX171618
**pME_G_0_5_001_3'UTR_Placeholder**	PX171831
**pME_G_0_5-6_006_OC3'1**	PX171824
**pME_G_0_5-6_007_OC3'2**	PX171825
**pME_G_0_5-6_008_OC3'3**	PX171826
**pME_G_0_5-6_009_OC3'4**	PX171827
**pME_G_0_5-6_010_OC3'5**	PX171828

**pME_G_0_5-6_011_OC3'6**	PX171829
**pME_G_0_5-6_012_OC3'7**	PX171830
**pME_Cp_0_5a_002_His-tag**	PX171619
**pME_Cp_0_5a_003_Hibit-tag**	PX171620
**pME_Cp_0_5a_004_Flag-tag**	PX171621
**pME_Cp_0_5a_005_HA-tag**	PX171622
**pME_Cp_0_5a_006_myc-tag**	PX171623
**pME_Cp_0_5a_007_mScarlet-I**	PX171624
**pME_Cp_0_5b_001_psbA_3'UTR_Cr**	PX171625
**pME_Cp_0_5b_002_atpB_3'UTR_Cr**	PX171626
**pME_Cp_0_5b_003_petL_3'UTR_Cr**	PX171627
**pME_Cp_0_5b_004_psbA_3'UTR_Nt**	PX171628
**pME_Cp_0_5b_005_TMV_3'UTR_Tv**	PX171629
**pME_Cp_0_5b_006_ftsH_3'UTR_Cr**	PX171630
**pME_Cp_0_6_005_psbH_3'Hom_Cr**	PX171631
**pME_Cp_0_6_006_psbH_5'Hom_rev_Cr**	PX171632
**pME_Cp_0_6_007_psbA_3'Hom_Cr**	PX171633
**pME_Cp_0_6_009_rbcL_3'Hom_Cr**	PX171634
**pME_Cp_0_6_013_petB_3'Hom_Cr**	PX171635
**pME_Cp_0_6_019_psbD_3'Hom_Cr**	PX171636
**pME_Cp_0_6_020_psbA_5'Hom_rev_Cr**	PX171637
**pME_Cp_0_6_021_petB_5'Hom_rev_Cr**	PX171638
**pME_Cp_0_6_022_rbcL_5'Hom_rev_Cr**	PX171639
**pME_Cp_0_6_023_psbD_5'Hom_rev_Cr**	PX171640
**pME_G_0_6_004_3C1LR**	PX171832
**pME_G_0_6_006_3C1SR**	PX171833
**pME_G_0_6_008_3C2LR**	PX171834
**pME_G_0_6_009_3C2SR**	PX171835
**pME_G_0_6_011_3C3LR**	PX171836
**pME_G_0_6_012_3C3SR**	PX171837
**pME_G_0_6_014_3C5LR**	PX171838
**pME_G_0_6_015_3C5SR**	PX171839
**pME_G_0_6_018_3C7LR**	PX171840
**pME_G_0_6_019_3C7SR**	PX171841
**pME_G_0_6_021_3C1SF**	PX171842
**pME_G_0_6_022_3C2SF**	PX171843
**pME_G_0_6_023_3C3SF**	PX171844
**pME_G_0_6_024_3C4SF**	PX171845
**pME_G_0_6_025_3C5SF**	PX171846
**pME_G_0_6_026_3C6SF**	PX171847
**pME_G_0_6_027_3C7SF**	PX171848
**pME_Cp_0_7-8_003_Amp_ColE1_mScarlet**	PX171641
**pME_Cp_0_7-8_004_Amp_ColE1_sfGFP**	PX171642
**pME_Cp_0_7-8_005_Kan_ColE1_mScarlet**	PX171643

**pME_Cp_0_7-8_006_Kan_ColE1_sfGFP**	PX171644
**pME_Cp_2_001_Promoter_PH_Meas_construct**	PX171645
**pME_Cp_2_002_5'UTR_PH_Meas_construct**	PX171646
**pME_Cp_2_003_3'UTR_PH_Meas_construct**	PX171647
**pME_Cp_2_005_atpA_5'UTR_Cr_Meas_construct**	PX171648
**pME_Cp_2_006_atpB_5'UTR_Cr_Meas_construct**	PX171649
**pME_Cp_2_007_chlL_5'UTR_Cr_Meas_construct**	PX171650
**pME_Cp_2_008_petB_5'UTR_Cr_Meas_construct**	PX171651
**pME_Cp_2_009_psaA_5'UTR_Cr_Meas_construct**	PX171652
**pME_Cp_2_010_psbA_5'UTR_Cr_Meas_construct**	PX171653
**pME_Cp_2_011_psbE_5'UTR_Cr_Meas_construct**	PX171654
**pME_Cp_2_012_psbF_5'UTR_Cr_Meas_construct**	PX171655
**pME_Cp_2_013_psbK_5'UTR_Cr_Meas_construct**	PX171656
**pME_Cp_2_014_petA_5'UTR_Meas_construct**	PX171657
**pME_Cp_2_015_petD_5'UTR_Meas_construct**	PX171658
**pME_Cp_2_016_psaB_5'UTR_Cr_Meas_construct**	PX171659
**pME_Cp_2_017_psbB_5'UTR_Cr_Meas_construct**	PX171660
**pME_Cp_2_018_psbC_5'UTR_Cr_Meas_construct**	PX171661
**pME_Cp_2_019_gene10_5'UTR_T7_Meas_construct**	PX171662
**pME_Cp_2_020_ccsA_5'UTR_Cr_Meas_construct**	PX171663
**pME_Cp_2_021_psaC_5'UTR_Cr_Meas_construct**	PX171664
**pME_Cp_2_022_rbcL_5'UTR_Cr_Meas_construct**	PX171665
**pME_Cp_2_023_rpoC1_5'UTR_Cr_Meas_construct**	PX171666
**pME_Cp_2_024_rpoC2_5'UTR_Cr_Meas_construct**	PX171667
**pME_Cp_2_025_tufA_5'UTR_Cr_Meas_construct**	PX171668
**pME_Cp_2_026_clpP_5'UTR_Cr_Meas_construct**	PX171669
**pME_Cp_2_027_rpl23_5'UTR_Cr_Meas_construct**	PX171670
**pME_Cp_2_028_rpl16_5'UTR_Cr_Meas_construct**	PX171671
**pME_Cp_2_029_rpoB1_5'UTR_Cr_Meas_construct**	PX171672
**pME_Cp_2_030_atpH_5'UTR_Cr_Meas_construct**	PX171673
**pME_Cp_2_031_psbD_5'UTR_Cr_Meas_construct**	PX171674
**pME_Cp_2_032_rbcL_5'UTR_Nt_Meas_construct**	PX171675
**pME_Cp_2_033_Synthetic_5'UTR_Meas_construct**	PX171676
**pME_Cp_2_034_atpH_5'UTR_Nt_Meas_construct**	PX171677
**pME_Cp_2_035_gene10_5'UTR_v2_T7_Meas_construct**	PX171678
**pME_Cp_2_036_psbA_5'UTR_Nt_Measurment_construct**	PX171679
**pME_Cp_2_037_psbC_5'UTR_Nt_Meas_construct**	PX171680
**pME_Cp_2_038_5'UTR_Dummy_Meas_construct**	PX171681
**pME_Cp_2_039_psbA_3'UTR_Cr_Meas_construct**	PX171682
**pME_Cp_2_040_rrnB_3'UTR_Ec_Meas_construct**	PX171683
**pME_Cp_2_041_psbA_3'UTR_Nt_Meas_construct**	PX171684
**pME_Cp_2_042_atpA_3'UTR_Cr_Meas_construct**	PX171685
**pME_Cp_2_043_rbcL_3'UTR_CDS_Cr_Meas_construct**	PX171686
**pME_Cp_2_044_atpB_3'UTR_Cr_Meas_construct**	PX171687

**pME_Cp_2_045_chlB_3'UTR_Cr_Meas_construct**	PX171688
**pME_Cp_2_046_clpP_3'UTR_Cr_Meas_construct**	PX171689
**pME_Cp_2_047_ftsH_3'UTR_Cr_Meas_construct**	PX171690
**pME_Cp_2_048_petA_3'UTR_Cr_Meas_construct**	PX171691
**pME_Cp_2_049_petD_3'UTR_Cr_Meas_construct**	PX171692
**pME_Cp_2_050_petL_3'UTR_Cr_Meas_construct**	PX171693
**pME_Cp_2_051_psaB_3'UTR_Cr_Meas_construct**	PX171694
**pME_Cp_2_052_psaC_3'UTR_Cr_Meas_construct**	PX171695
**pME_Cp_2_053_psbA_3'UTR_Cr_Meas_construct**	PX171696
**pME_Cp_2_054_psbC_3'UTR_Cr_Meas_construct**	PX171697
**pME_Cp_2_055_rbcL_3'UTR_Cr_Meas_construct**	PX171698
**pME_Cp_2_056_rpl36_3'UTR_Cr_Meas_construct**	PX171699
**pME_Cp_2_057_rpoB2_3'UTR_Cr_Meas_construct**	PX171700
**pME_Cp_2_058_rpoC2_3'UTR_Cr_Meas_construct**	PX171701
**pME_Cp_2_059_rps_3'UTR_Cr_Meas_construct**	PX171702
**pME_Cp_2_060_rps2_3'UTR_Cr_Meas_construct**	PX171703
**pME_Cp_2_061_rps8_3'UTR_Cr_Meas_construct**	PX171704
**pME_Cp_2_062_rps11_3'UTR_Cr_Meas_construct**	PX171705
**pME_Cp_2_063_rps19_3'UTR_Cr_Meas_construct**	PX171706
**pME_Cp_2_064_tufA_3'UTR_Cr_Meas_construct**	PX171707
**pME_Cp_2_065_ycf1_3'UTR_Cr_Meas_construct**	PX171708
**pME_Cp_2_066_ycf12_3'UTR_Cr_Meas_construct**	PX171709
**pME_Cp_2_067_rbcL_3'UTR_Nt_Meas_construct**	PX171710
**pME_Cp_2_068_BMV_3'UTR_Meas_construct**	PX171711
**pME_Cp_2_069_TYMV_3'UTR_Meas_construct**	PX171712
**pME_Cp_2_070_rpoA_3'UTR_Nt_Meas_construct**	PX171713
**pME_Cp_2_071_petD_3'UTR_Cr_Meas_construct**	PX171714
**pME_Cp_2_072_TMV_3'UTR_Meas_construct**	PX171715
**pME_Cp_2_073_psaC_3'UTR_Nt_Meas_construct**	PX171716
**pME_Cp_2_074_3'UTR_Dummy_Meas_construct**	PX171717
**pME_Cp_2_097_mScarlet-I_Meas_construct**	PX171718
**pME_Cp_2_098_CDS_PH_Meas_construct**	PX171719
**pME_Cp_2_100_tdTomato_Meas_construct**	PX171720
**pME_Cp_2_101_mVenus_Meas_construct**	PX171721
**pME_Cp_2_120_Fluc_Meas_construct**	PX171722
**pME_Cp_2_121_Syn_Prom_01_Meas_construct**	PX171723
**pME_Cp_2_122_Syn_Prom_02_Meas_construct**	PX171724
**pME_Cp_2_123_Syn_Prom_03_Meas_construct**	PX171725
**pME_Cp_2_124_Syn_Prom_04_Meas_construct**	PX171726
**pME_Cp_2_125_Syn_Prom_05_Meas_construct**	PX171727
**pME_Cp_2_126_Syn_Prom_06_Meas_construct**	PX171728
**pME_Cp_2_127_Syn_Prom_07_Meas_construct**	PX171729
**pME_Cp_2_128_Syn_Prom_08_Meas_construct**	PX171730
**pME_Cp_2_129_Syn_Prom_09_Meas_construct**	PX171731

**pME_Cp_2_130_Syn_Prom_10_Meas_construct**	PX171732
**pME_Cp_2_131_Syn_Prom_11_Meas_construct**	PX171733
**pME_Cp_2_132_Syn_Prom_12_Meas_construct**	PX171734
**pME_Cp_2_133_Syn_Prom_13_Meas_construct**	PX171735
**pME_Cp_2_134_Syn_Prom_14_Meas_construct**	PX171736
**pME_Cp_2_135_Syn_Prom_15_Meas_construct**	PX171737
**pME_Cp_2_136_Syn_Prom_16_Meas_construct**	PX171738
**pME_Cp_2_137_Syn_Prom_17_Meas_construct**	PX171739
**pME_Cp_2_138_Syn_Prom_18_Meas_construct**	PX171740
**pME_Cp_2_139_Syn_Prom_19_Meas_construct**	PX171741
**pME_Cp_2_140_Syn_Prom_20_Meas_construct**	PX171742
**pME_Cp_2_141_Syn_Prom_21_Meas_construct**	PX171743
**pME_Cp_2_142_Syn_Prom_22_Meas_construct**	PX171744
**pME_Cp_2_143_rbcL_Prom_Cr_Meas_construct**	PX171745
**pME_Cp_2_144_trnA_Glu2_Prom_Cr_Meas_construct**	PX171746
**pME_Cp_2_145_WendyII_Prom_Cr_Meas_construct**	PX171747
**pME_Cp_2_146_IEE_Meas_construct**	PX171748
**pME_Cp_2_148_Prom_Dummy_Meas_construct**	PX171749
**pME_Cp_2_151_5'UTR_Dummy_IEE_Meas_construct**	PX171750
**pME_Cp_2_152_atpE-ycf12_IEE_Cr_IEE_Meas_construct**	PX171751
**pME_Cp_2_153_chlL-rpl36_IEE_Cr_IEE_Meas_construct**	PX171752
**pME_Cp_2_154_petG-rps3_IEE_Cr_IEE_Meas_construct**	PX171753
**pME_Cp_2_155_psbB-psbT_IEE_Cr_IEE_Meas_construct**	PX171754
**pME_Cp_2_156_psbE-rps9_IEE_Cr_IEE_Meas_construct**	PX171755
**pME_Cp_2_157_psbF-psbL_IEE_Cr_IEE_Meas_construct**	PX171756
**pME_Cp_2_158_psbJ-atpI_IEE_Cr_IEE_Meas_construct**	PX171757
**pME_Cp_2_159_rpl2-rps19_IEE_Cr_IEE_Meas_construct**	PX171758
**pME_Cp_2_160_rpl23-rpl2_IEE_Cr_IEE_Meas_construct**	PX171759
**pME_Cp_2_161_rps14-rps7_IEE_Cr_IEE_Meas_construct**	PX171760
**pME_Cp_2_162_rps18-rps2_IEE_Cr_IEE_Meas_construct**	PX171761
**pME_Cp_2_163_trnF-psaC_IEE_Cr_IEE_Meas_construct**	PX171762
**pME_Cp_2_164_ycf3-rps18_IEE_Cr_IEE_Meas_construct**	PX171763
**pME_Cp_2_165_ycf4-ycf3_IEE_Cr_IEE_Meas_construct**	PX171764
**pME_Cp_2_166_tscA-chlN_IEE5_Cr_IEE_Meas_construct**	PX171765
**pME_Cp_2_185_mScarlet-I_His-tag**	PX171766
**pME_Cp_2_186_mScarlet-I_Hibit-tag**	PX171767
**pME_Cp_2_187_mScarlet-I_Flag-tag**	PX171768
**pME_Cp_2_188_mScarlet-I_HA-tag**	PX171769
**pME_Cp_2_189__mScarlet-I_c-Myc-tag**	PX171770
**pME_Cp_2_295_Tobra_test_construct**	PX171771
**pME_Cp_2_296_Arg_test_construct**	PX171772
**pME_Cp_2_297_PtxD_test_construct**	PX171773
**pME_Cp_2_298_mCerulean_Meas_construct**	PX171774
**pME_Cp_2_299_Psyn15_5'psbF_3'rps8_psbH_locus**	PX171775

**pME_Cp_2_300_Psyn22_5'rpoC1_3'petD_psbH_locus**	PX171776
**pME_Cp_2_301_Psyn9_5'rbcL_3'petA_psbH_locus**	PX171777
**pME_Cp_2_302_Psyn5_5'psbD_3'petL_psbH_locus**	PX171778
**pME_Cp_2_303_PrbcL_5'psaC_3'rps2_psbH_locus**	PX171779
**pME_Cp_2_304_PpsaA2_5'psaB_3'ycf12_psbH_locus**	PX171780
**pME_Cp_2_305_Psyn17_5'clpP_3'TMV_psbH_locus**	PX171781
**pME_Cp_2_306_Psyn2_5'psbE_3'atpB_psbH_locus**	PX171782
**pME_Cp_2_307_PGlu2_5'psbK_3'tufA_psbH_locus**	PX171783
**pME_Cp_2_308_PWendyII_5'psbC_3'ftsH_psbH_locus**	PX171784
**pME_Cp_2_309_Prrn16_5'psaA_3'psbA_psbH_locus**	PX171785
**pME_Cp_2_310_Psyn7_5'clpP_3'rps11_psbH_locus**	PX171786
**pME_Cp_2_311_Psyn9_5'clpP_3'rps11_psbH_locus**	PX171787
**pME_Cp_2_312_Prrn16_5'clpP_3'rps11_psbH_locus**	PX171788
**pME_Cp_2_312_Psyn7_5'clpP_3'rrnB_psbH_locus**	PX171789
**pME_Cp_2_313_Psyn7_5'petD_3'rrnB_psbH_locus**	PX171790
**pME_Cp_2_314_Psyn7_5'psaA_3'rrnB_psbH_locus**	PX171791
**pME_Cp_2_315_Prrn16_5'clpP_3'rrnB_psbH_locus**	PX171792
**pME_Cp_2_316_Prrn16_5'clpP_3'psbA_psbH_locus**	PX171793
**pME_Cp_2_317_PWendyII_5'clpP_3'petA_psbH_locus**	PX171794
**pME_Cp_2_318_Psyn9_5'psbC_3'petA_psbH_locus**	PX171795
**pME_Cp_2_319_PWendyII_5'psbC_3'rps11_psbH_locus**	PX171796
**pME_Cp_2_320_PWendyII_5'psbC_3'petA_psbH_locus**	PX171797
**pME_Cp_2_321_PWendyII_5'clpP_3'petA_rbcL_locus**	PX171798
**pME_Cp_2_322_Psyn9_5'psbC_3'petA_rbcL_locus**	PX171799
**pME_Cp_2_323_PWendyII_5'psbC_3'rps11_rbcL_locus**	PX171800
**pME_Cp_2_324_PWendyII_5'psbC_3'petA_rbcL_locus**	PX171801
**pME_Cp_2_325_PWendyII_5'clpP_3'petA_psbD_locus**	PX171802
**pME_Cp_2_326_Psyn9_5'psbC_3'petA_psbD_locus**	PX171803
**pME_Cp_2_327_PWendyII_5'psbC_3'rps11_psbD_locus**	PX171804
**pME_Cp_2_328_PWendyII_5'psbC_3'petA_psbD_locus**	PX171805
**pME_Cp_2_329_PWendyII_5'clpP_3'petA_petB_locus**	PX171806
**pME_Cp_2_330_Psyn9_5'psbC_3'petA_petB_locus**	PX171807
**pME_Cp_2_332_PWendyII_5'psbC_3'petA_petB_locus**	PX171808
**pME_Cp_2_333_PWendyII_5'psbC_3'rps11_petB_locus**	PX171809
**pME_Cp_2_334_South_Pathway**	PX171810

References

1. Wannathong, T., Waterhouse, J. C., Young, R. E. B., Economou, C. K. & Purton, S. New tools for chloroplast genetic engineering allow the synthesis of human growth hormone in the green alga Chlamydomonas reinhardtii. *Appl. Microbiol. Biotechnol.* **100**, 5467–5477 (2016).
2. Cavaiuolo, M., Kuras, R., Wollman, F., Choquet, Y. & Vallon, O. Small RNA profiling in Chlamydomonas: insights into chloroplast RNA metabolism. *Nucleic Acids Res.* **45**, 10783–10799 (2017).
3. Gallaher, S. D. *et al.* High-throughput sequencing of the chloroplast and mitochondrion of Chlamydomonas reinhardtii to generate improved de novo assemblies, analyze expression patterns and transcript speciation, and evaluate diversity among laboratory strains and wild isolates. *Plant J.* **93**, 545–565 (2018).
