# Supplementary material for: A modular high-throughput approach for advancing synthetic biology in the chloroplast of Chlamydomonas
Source: Nat Plants. 2025 Nov 3;11(11):2332–49. doi: 10.1038/s41477-025-02126-2 (PMC12626891; doi:10.1038/s41477-025-02126-2)
Supplement: Supplementary file 2 — Reporting Summary [file 41477_2025_2126_MOESM2_ESM.pdf]

nature portfolio

Corresponding author(s): Tobias Jürgen Erb, Felix Willmund, René Inckemann

Last updated by author(s): Aug 4, 2025

Reporting Summary

Nature Portfolio wishes to improve the reproducibility of the work that we publish. This form provides structure for consistency and transparency in reporting. For further information on Nature Portfolio policies, see our Editorial Policies and the Editorial Policy Checklist.

Statistics

For all statistical analyses, confirm that the following items are present in the figure legend, table legend, main text, or Methods section.

n/a	Confirmed	
☐	☒	The exact sample size (*n*) for each experimental group/condition, given as a discrete number and unit of measurement
☐	☒	A statement on whether measurements were taken from distinct samples or whether the same sample was measured repeatedly
☐	☒	The statistical test(s) used AND whether they are one- or two-sided<br>*Only common tests should be described solely by name; describe more complex techniques in the Methods section.*
☒	☐	A description of all covariates tested
☒	☐	A description of any assumptions or corrections, such as tests of normality and adjustment for multiple comparisons
☐	☒	A full description of the statistical parameters including central tendency (e.g. means) or other basic estimates (e.g. regression coefficient) AND variation (e.g. standard deviation) or associated estimates of uncertainty (e.g. confidence intervals)
☐	☒	For null hypothesis testing, the test statistic (e.g. *F*, *t*, *r*) with confidence intervals, effect sizes, degrees of freedom and *P* value noted<br>*Give P values as exact values whenever suitable.*
☒	☐	For Bayesian analysis, information on the choice of priors and Markov chain Monte Carlo settings
☒	☐	For hierarchical and complex designs, identification of the appropriate level for tests and full reporting of outcomes
☒	☐	Estimates of effect sizes (e.g. Cohen's *d*, Pearson's *r*), indicating how they were calculated

Our web collection on statistics for biologists contains articles on many of the points above.

Software and code

Policy information about availability of computer code

Data collection	Collection was conducted with Microsoft Excel 2016 (16.0.5443.1000), Masshunter Workstation LC/MS Data Acquisition (10.1.48), Tecan i-control (3.9.1.0), PhenoBooth (2.22.0607.1), SONY SH800S Cell Sorter Software (2.1.6), Spectroquant (S-ID 7087).
Data analysis	Analysis was conducted with Python (3.10.5), Pandas library (1.4.3), Plotly library (5.9.0), FlowCal library (1.3.0), Scipy library (1.8.1), GraphPad Prism (10.1.0 (316)), DIANN (1.8), SafeQuant (2.4.2), Masshunter Workstation for QQQ (10.0), Geneious Prime (2023.1.2), Fiji (2.16). Custom codes used for data analysis have been deposited on Github (https://github.com/ChlamyMarburg/ChloroplastTools).

For manuscripts utilizing custom algorithms or software that are central to the research but not yet described in published literature, software must be made available to editors and reviewers. We strongly encourage code deposition in a community repository (e.g. GitHub). See the Nature Portfolio guidelines for submitting code & software for further information.

Data

Policy information about availability of data

All manuscripts must include a data availability statement. This statement should provide the following information, where applicable:

- Accession codes, unique identifiers, or web links for publicly available datasets
- A description of any restrictions on data availability
- For clinical datasets or third party data, please ensure that the statement adheres to our policy

Raw mass spectrometry data, as well as annotated plasmid maps will be submitted to the "Edmund" Max Planck Society open-source database, as well as deposited

in GenBank. The mass spectrometry proteomics data have been deposited to the ProteomeXchange Consortium via the PRIDE [1] partner repository with the dataset identifier PXD051642.

Research involving human participants, their data, or biological material

Policy information about studies with human participants or human data. See also policy information about sex, gender (identity/presentation), and sexual orientation and race, ethnicity and racism.

Reporting on sex and gender	Does not apply.
Reporting on race, ethnicity, or other socially relevant groupings	Does not apply.
Population characteristics	Does not apply.
Recruitment	Does not apply.
Ethics oversight	Does not apply.

Note that full information on the approval of the study protocol must also be provided in the manuscript.

Field-specific reporting

Please select the one below that is the best fit for your research. If you are not sure, read the appropriate sections before making your selection.

☒ Life sciences ☐ Behavioural & social sciences ☐ Ecological, evolutionary & environmental sciences

For a reference copy of the document with all sections, see nature.com/documents/nr-reporting-summary-flat.pdf

Life sciences study design

All studies must disclose on these points even when the disclosure is negative.

Sample size	Sample size was determined based on the amount of genetic elements (e.g. Promoters, 5'UTRs, 3'UTRs, IEEs) that were designed, assembled and successfully transformed in the chloroplast of Chlamydomonas reinhardtii.
Data exclusions	Biological replicates were excluded if genetic confirmation of the replicates was negative, as they would not represent the behavior of genetic parts.
Replication	Figures containing fluorescent or luminescence reporter measurements (mScarlet-I, Nanoluc) were plotted using 5 biological replicates and each in 3 technical replicates (Fig2, Fig3, Fig4a, Fig5).<br>For the library experiment in Fig4c, due to the nature of the experiment, each variant is represented with one biological replicate.<br>The growth experiments in Fig6 and Supp. Fig3 were carried out twice and were reproducible. Proteomics and metabolomics analyses in Fig6 were tested in three biological replicates.
Randomization	Randomization does not apply to the study.
Blinding	Blinding does not apply to the study.

Reporting for specific materials, systems and methods

We require information from authors about some types of materials, experimental systems and methods used in many studies. Here, indicate whether each material, system or method listed is relevant to your study. If you are not sure if a list item applies to your research, read the appropriate section before selecting a response.

Materials & experimental systems

n/a	Involved in the study	
☐	☒	Antibodies
☒	☐	Eukaryotic cell lines
☒	☐	Palaeontology and archaeology
☒	☐	Animals and other organisms
☒	☐	Clinical data
☒	☐	Dual use research of concern
☐	☒	Plants

Methods

n/a	Involved in the study	
☒	☐	ChIP-seq
☐	☒	Flow cytometry
☒	☐	MRI-based neuroimaging

Antibodies

Antibodies used	The antibodies used in Fig2 are commercially available : HA tag mouse monoclonal antibody (H3663, multiple lots, Sigma-Aldrich), FLAG mouse monoclonal antibody (F3165, multiple lots, Sigma-Aldrich), and Myc mouse monoclonal antibody (13-2500, multiple lots, Invitrogen), anti mCherry (own production), anti rabbit secondary antibody (T6778, lot 092M4751V, Sigma-Aldrich), anti mouse secondary antibody (Covance Art No.: MMS-150P, Lot No: E10032BF).
Validation	All commercial antibodies used in the study passed validation. Own antibody was tested against their epitope and against background signal in samples without the epitope.

Plants

Seed stocks	chlamydomonas reinhardtii strains: cc-125 , cc-5797 , sourced from https://www.chlamycollection.org/
Novel plant genotypes	In this study 3,156 individual transplastomic strains were generated by biolistic chloroplast transformation by using hundreds of different constructs. The process is described in detail in the method section. For each genetic construct 16 biological replicates were selected (if enough transformants available) and maintained
Authentication	All transplastomic strains have been genetically confirmed via colony PCR (which are also shown in the supplementary information). The individual knock-out of cc-5797 were confirmed by colony PCR and sequencing (supplementary figure 2).

Flow Cytometry

Plots

Confirm that:

☒ The axis labels state the marker and fluorochrome used (e.g. CD4-FITC).

☒ The axis scales are clearly visible. Include numbers along axes only for bottom left plot of group (a 'group' is an analysis of identical markers).

☒ All plots are contour plots with outliers or pseudocolor plots.

☒ A numerical value for number of cells or percentage (with statistics) is provided.

Methodology

Sample preparation	WT and mScarlet-I Chlamydomonas reinhardtii expressing strains underwent a two-day pre-culture and a two-day culture in TAP medium, with spectinomycin (100 µg/mL) added for transformants. Samples were diluted tenfold in TAP for optimal sorting and used for flow cytometry.
Instrument	SONY SH800S Cell Sorter.
Software	SONY SH800S Cell Sorter Software (2.1.6), Python FlowCal library (1.3.0)
Cell population abundance	No sorting was performed using the Flow Cytometer in this study.
Gating strategy	Initial gates for analysis were set based on the FSC and Chlorophyll channel (as indicated in supplementary text 3) of the Flow Cytometer. Cells displaying FSC values higher than 1.10^4, and chlorophyll between 1.10^5 - 5.10^6 were selected for comparison of Wild-Type and transformants in their mScarlet-I fluorescence. The rationale used for the gating was to select for cells that contained high chlorophyll fluorescence confirming the integrity of the cells, and to exclude any cell particles for the analysis.

☐ Tick this box to confirm that a figure exemplifying the gating strategy is provided in the Supplementary Information.
