# Supplementary material for: A modular high-throughput approach for advancing synthetic biology in the chloroplast of Chlamydomonas
Source: Nat Plants. 2025 Nov 3;11(11):2332–49. doi: 10.1038/s41477-025-02126-2 (PMC12626891; doi:10.1038/s41477-025-02126-2)

ED Figure 2: Unprocessed Agarose gel, Tobramycin cPCR confirming integration and homoplasmy, shown in panel 2c.

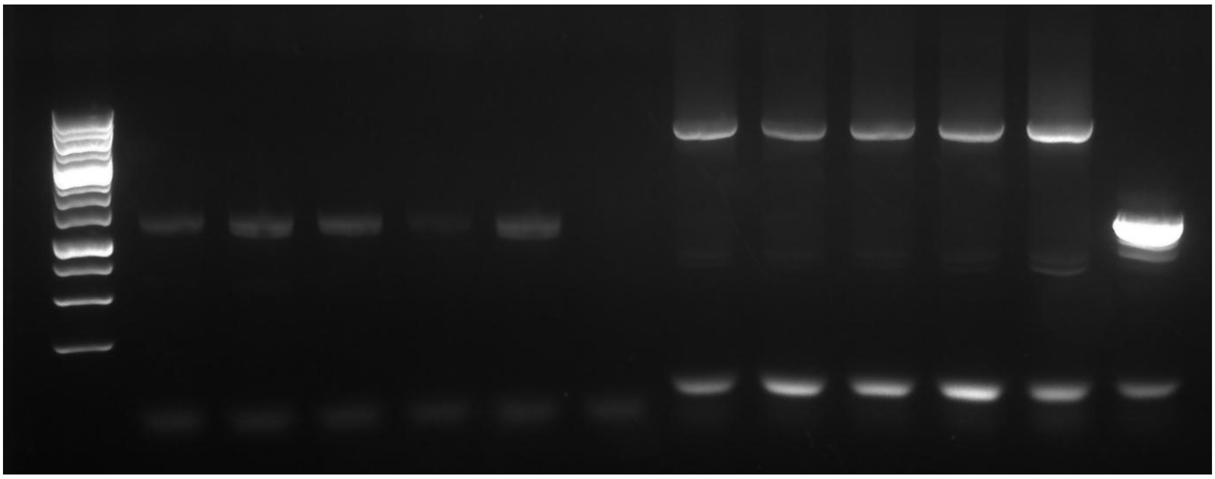

ED Figure 2: Unprocessed plate pictures, Kanamycin marker transformation plates shown in panel 2d.

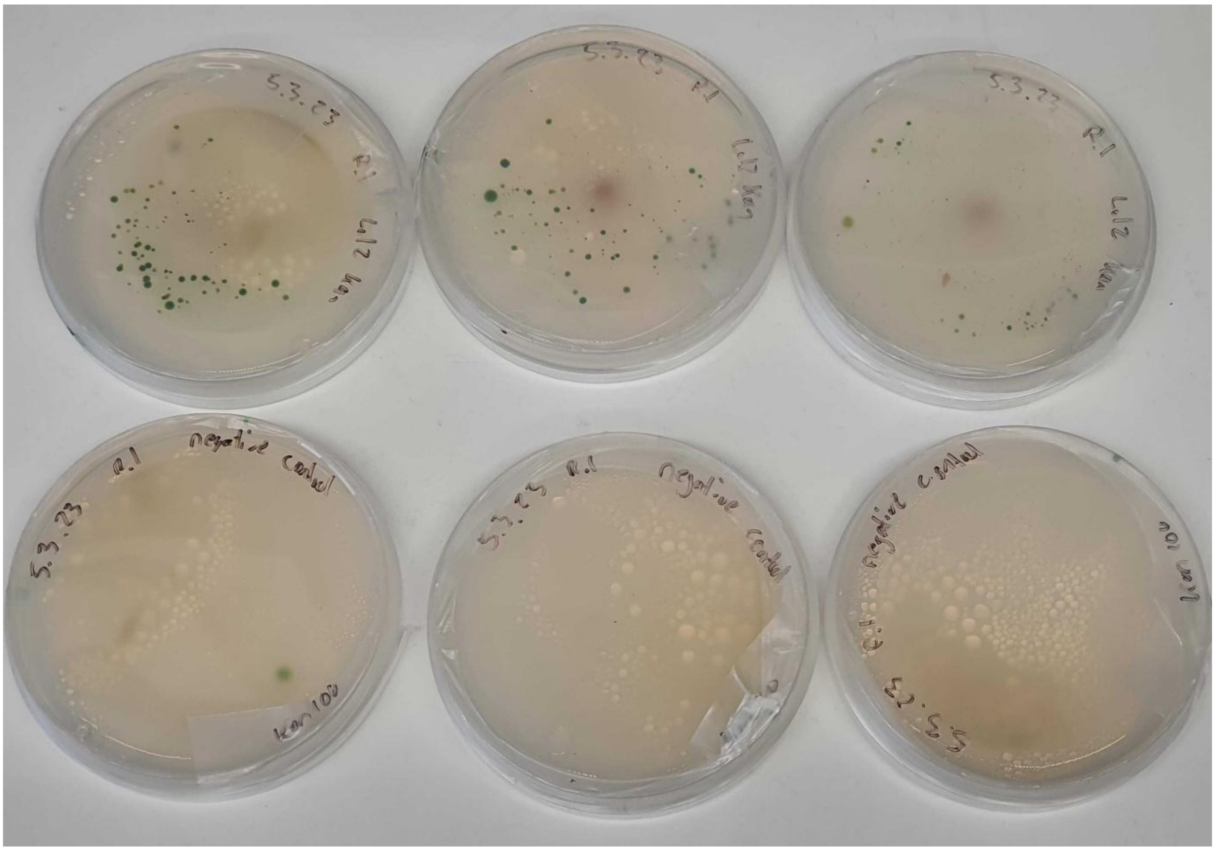

ED Figure 2: Unprocessed western blots and Ponceau , for His WB with anti-His antibody (a) shown in the black rectangle, anti-scarlet-I antibody (b), and ponceau (c) shown in a black rectangle, all shown in panel 2e.

A

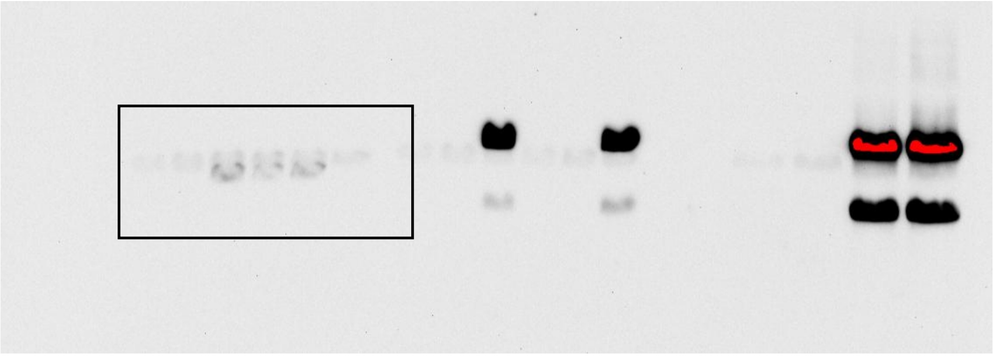

B

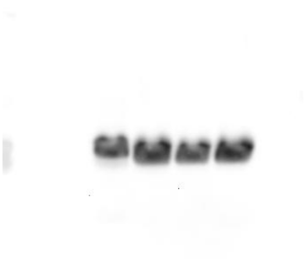

C

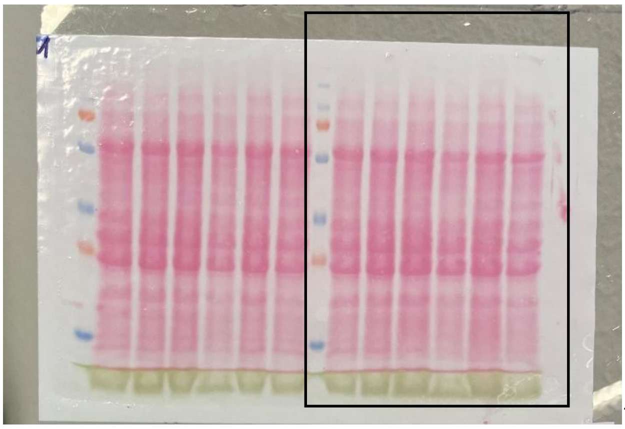

*

ED Figure 2: Unprocessed western blots and Ponceau, for Strep WB with anti-scarlet-I antibody (A) shown in the black rectangle, and ponceau (B) also shown in a black rectangle, all shown in panel 2e.

A

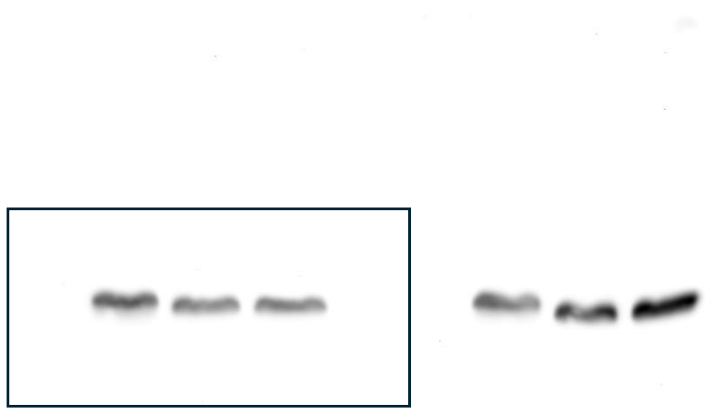

B

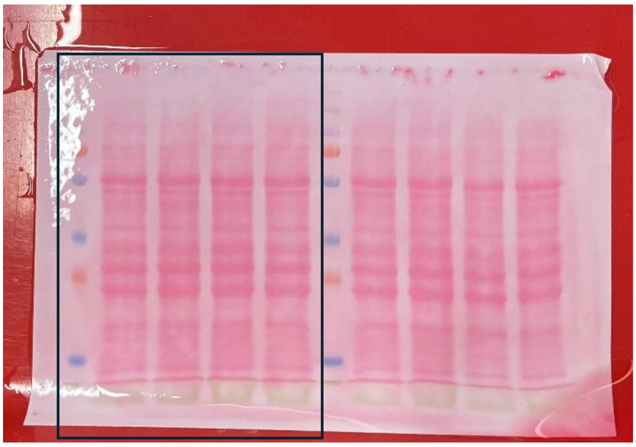

ED Figure 2: Unprocessed western blots and Ponceau, for Strep WB with anti-scarlet-I antibody (A) shown in the black rectangle, and ponceau (B), all shown in panel 2e.

A

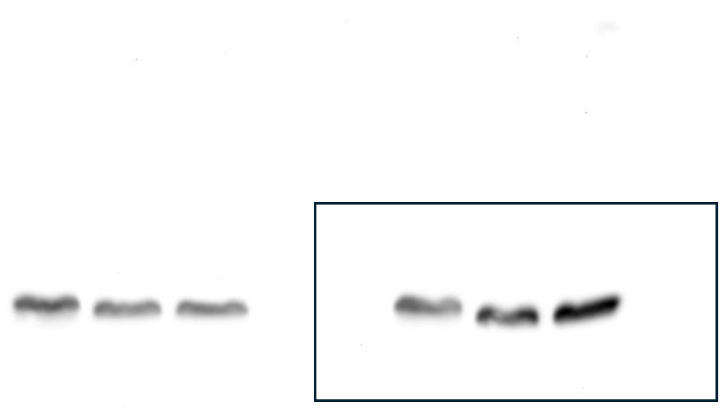

B

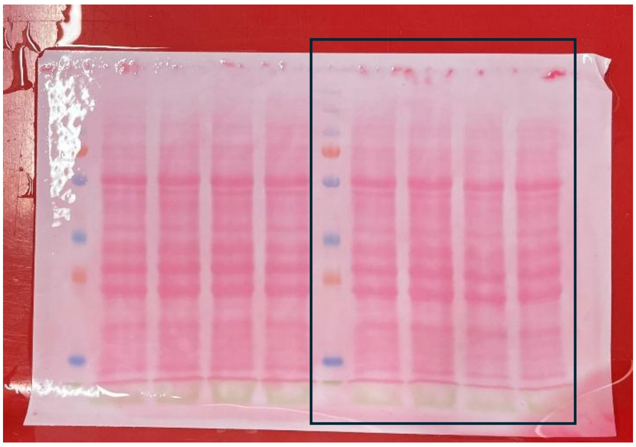

Supplement: Supplementary file 3 — Unprocessed images of DNA and protein gels for Fig. 2 and Extended Data Fig. 2, and statistical source data for Figs. 2–6 and Extended Data Figs. 2–5. [file 41477_2025_2126_MOESM3_ESM.zip › Inckemann_Source_data/Inckemann_Source_Data_ED2.pdf]
