# Supplementary material for: A modular high-throughput approach for advancing synthetic biology in the chloroplast of Chlamydomonas
Source: Nat Plants. 2025 Nov 3;11(11):2332–49. doi: 10.1038/s41477-025-02126-2 (PMC12626891; doi:10.1038/s41477-025-02126-2)

ED Figure 4: Unprocessed Agarose gel, Genotyping of transplastomic strains. Gel depiction of cPCR amplification for the NanoLuc reporter gene across transplastomic strains shown in Figure 5c, integration in RbcL locus.

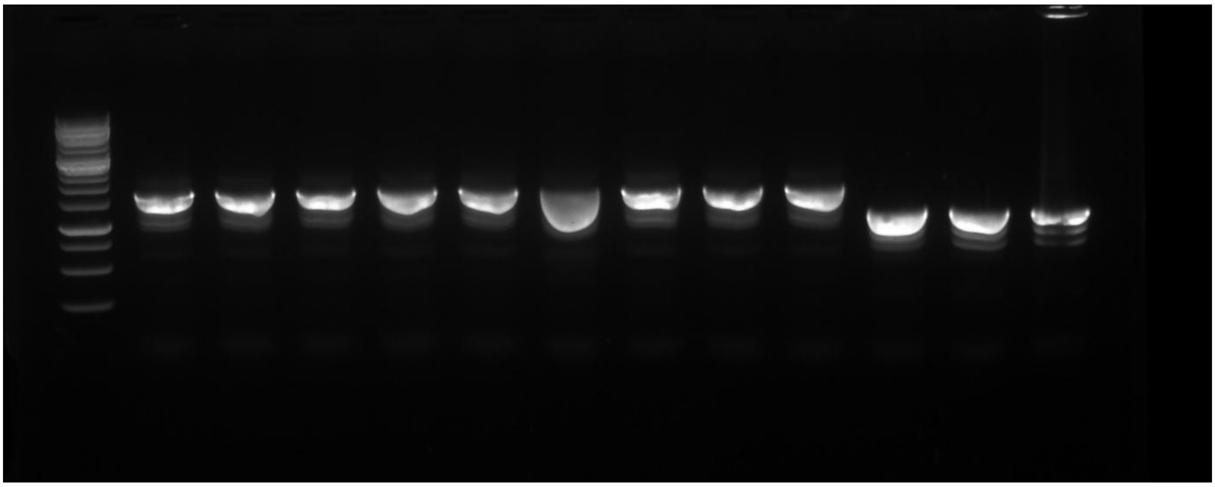

ED Figure 4: Unprocessed Agarose gel, Genotyping of transplastomic strains. Gel depiction of cPCR amplification for the NanoLuc reporter gene across transplastomic strains shown in Figure 5c, integration in psbD locus.

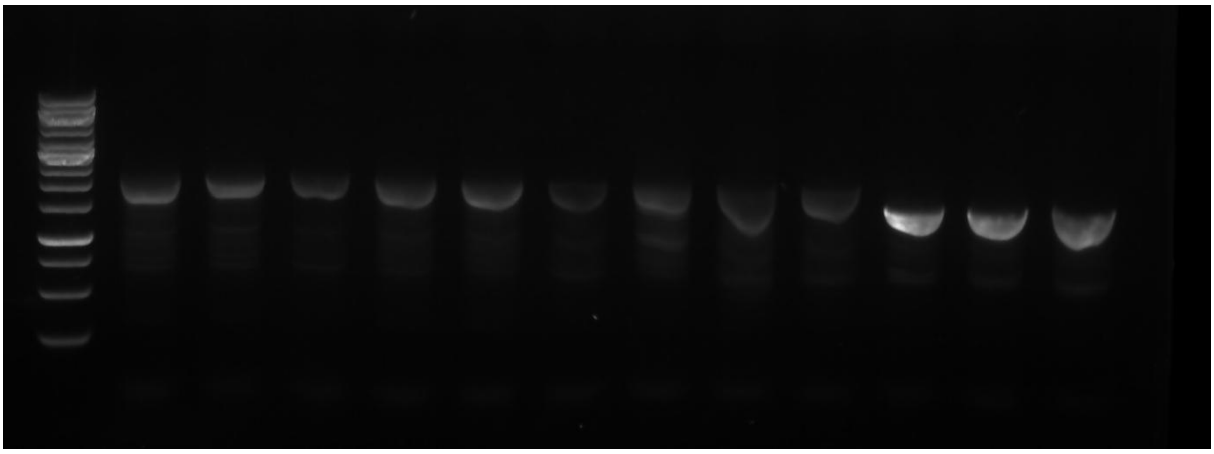

ED Figure 4: Unprocessed Agarose gel, Genotyping of transplastomic strains. Gel depiction of cPCR amplification for the NanoLuc reporter gene across transplastomic strains shown in Figure 5c, integration in psbH locus.

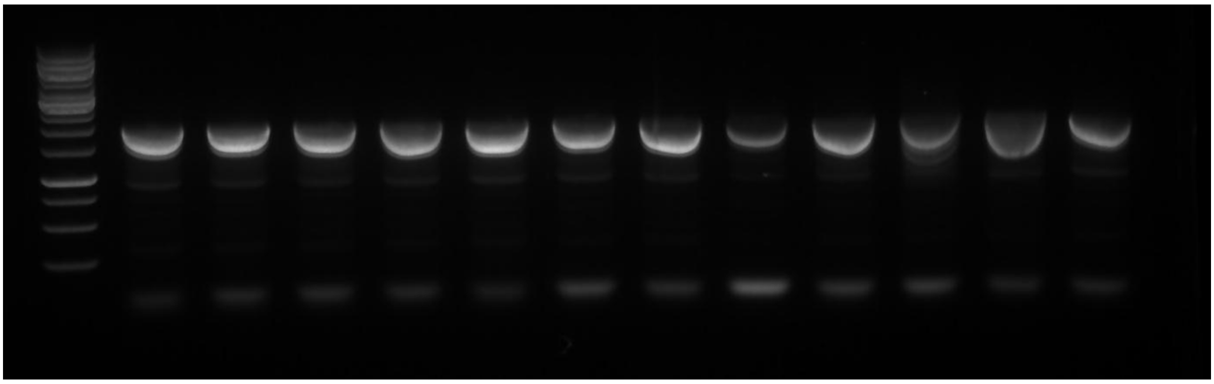

ED Figure 4: Unprocessed Agarose gel, Genotyping of transplastomic strains. Gel depiction of cPCR amplification for the NanoLuc reporter gene across transplastomic strains shown in Figure 5c, integration in petB locus.

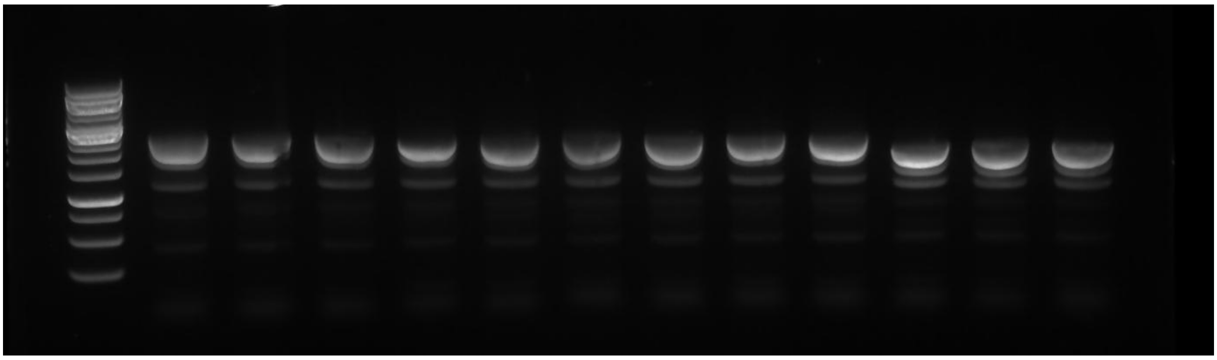

ED Figure 4: Unprocessed Agarose gel, Genotyping of transplastomic strains. Gel depiction of cPCR amplification for the NanoLuc reporter gene across transplastomic strains shown in Figure 5c, WT controls.

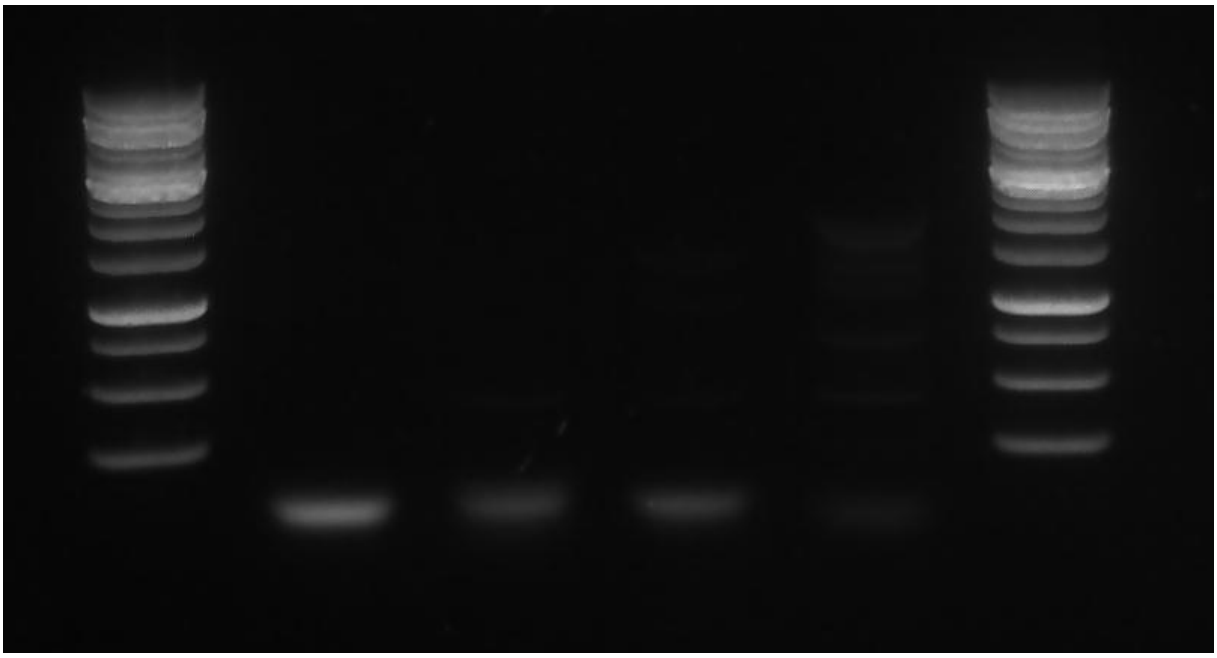

Supplement: Supplementary file 3 — Unprocessed images of DNA and protein gels for Fig. 2 and Extended Data Fig. 2, and statistical source data for Figs. 2–6 and Extended Data Figs. 2–5. [file 41477_2025_2126_MOESM3_ESM.zip › Inckemann_Source_data/Inckemann_Source_Data_ED4.pdf]
