# Supplementary material for: A modular high-throughput approach for advancing synthetic biology in the chloroplast of Chlamydomonas
Source: Nat Plants. 2025 Nov 3;11(11):2332–49. doi: 10.1038/s41477-025-02126-2 (PMC12626891; doi:10.1038/s41477-025-02126-2)

Figure 2G: Unprocessed Western blots and Ponceau, for myc WB with anti-myc antibody (a), anti-scarlet-I antibody (b), and ponceau (c).

A

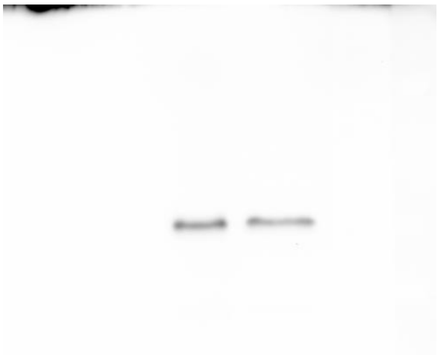

B

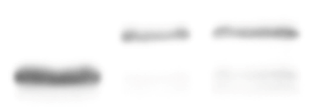

C

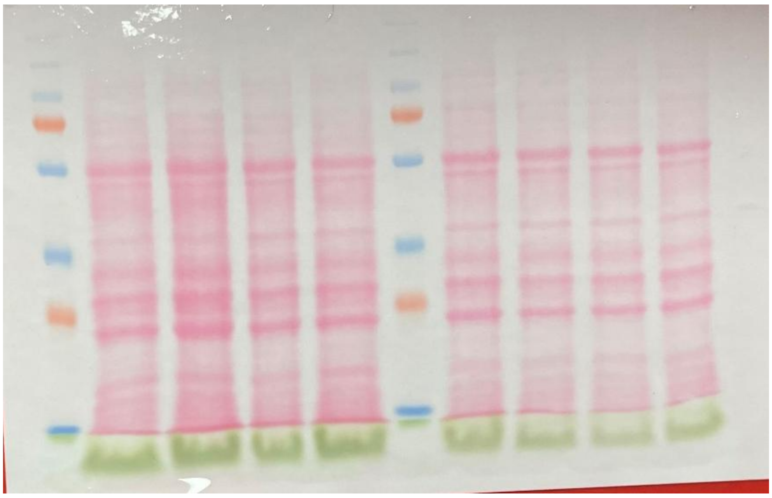

Figure 2G: Unprocessed Western blots and Ponceau, for FLAG WB with anti-FLAG antibody (a), anti-scarlet-I antibody (b), and ponceau (c).

A

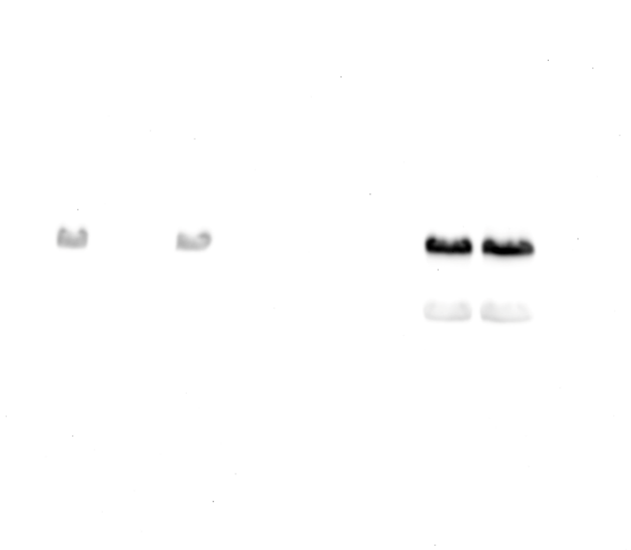

B

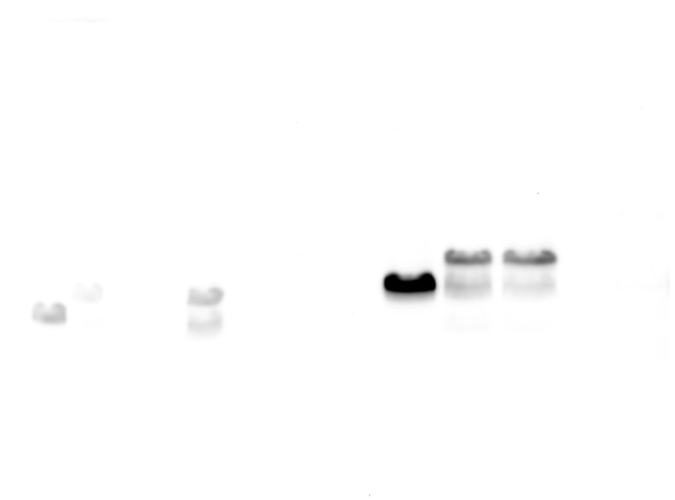

C

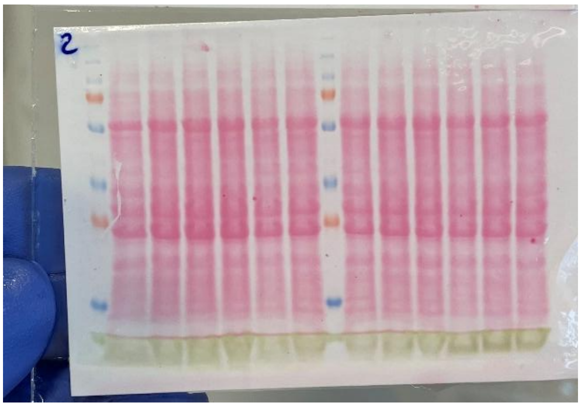

Figure 2G: Unprocessed Western blots and Ponceau, for FLAG WB with anti-FLAG antibody (a), anti-scarlet-I antibody (b), and ponceau (c).

A

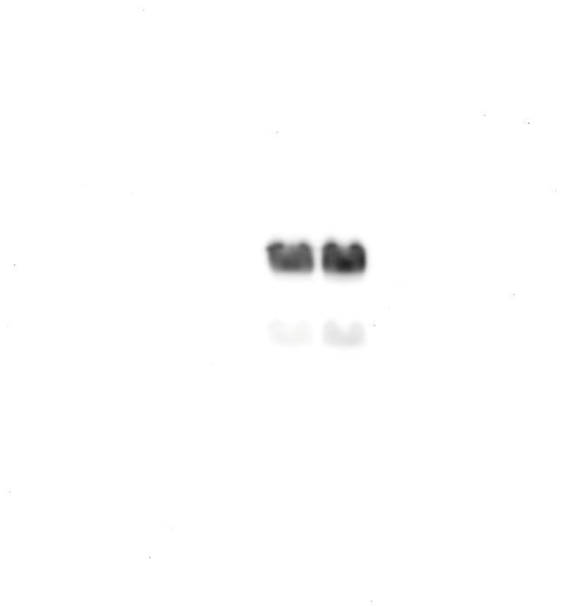

B

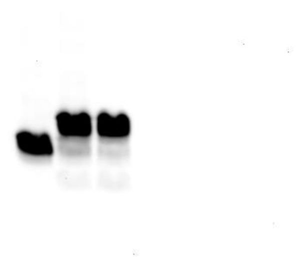

C

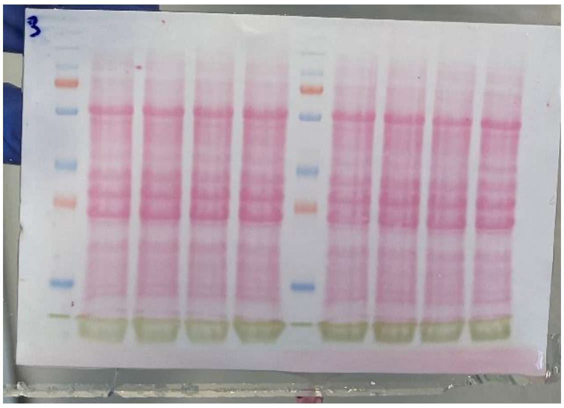

Figure 2B: Unprocessed plate pictures, Arginine marker transformation plates shown in panel 2B.

<

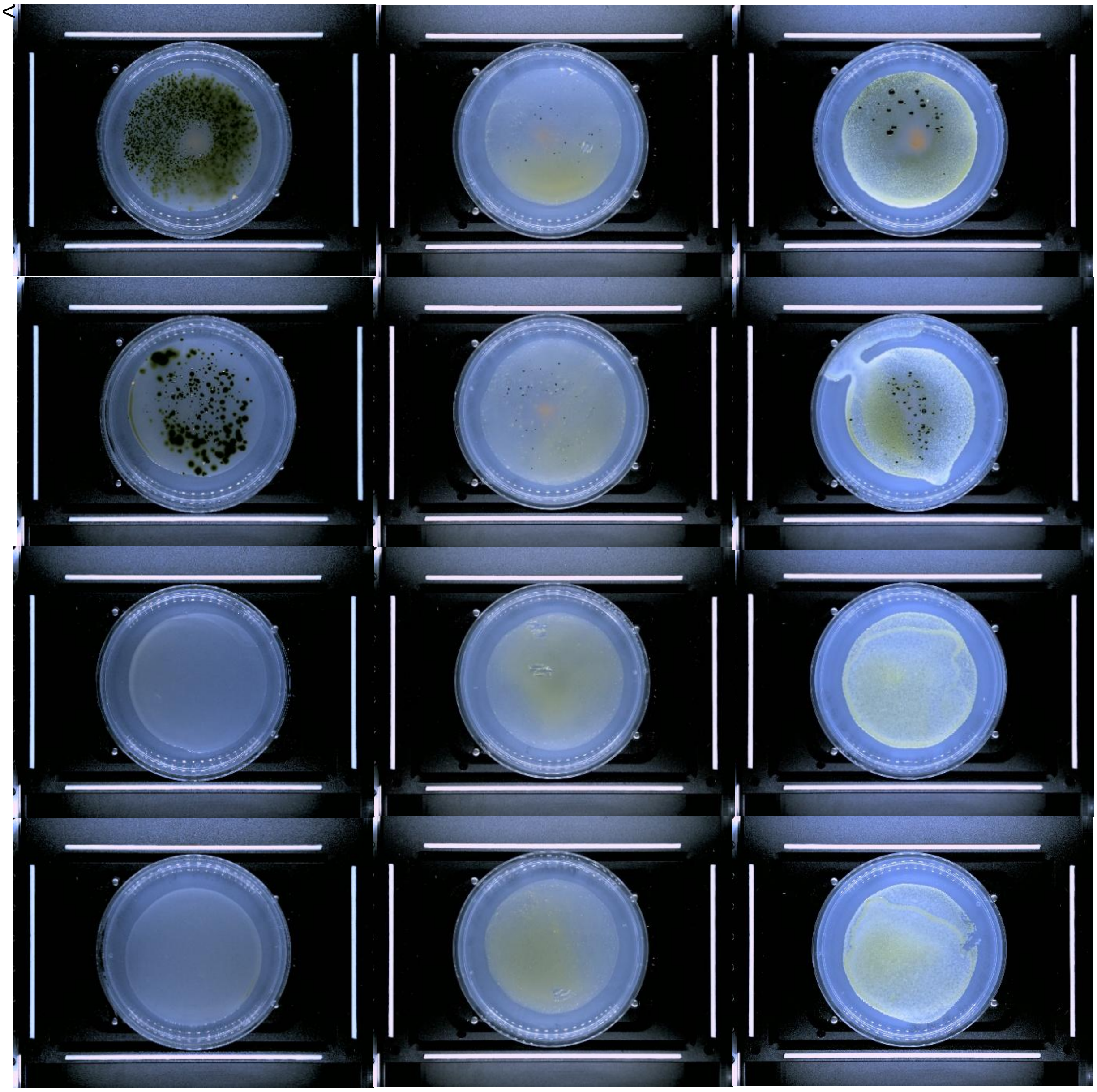

Supplement: Supplementary file 3 — Unprocessed images of DNA and protein gels for Fig. 2 and Extended Data Fig. 2, and statistical source data for Figs. 2–6 and Extended Data Figs. 2–5. [file 41477_2025_2126_MOESM3_ESM.zip › Inckemann_Source_data/Inckemann_Source_Data_Fig.2.pdf]
